# Supplementary material for: Enhancing Pediatric Extracorporeal Membrane Oxygenation Education Through Process-Oriented Guided Inquiry Learning Sessions for Fellows and Advanced Practice Providers
Source: MedEdPORTAL. 2026 May 12;22:11600. doi: 10.15766/mep_2374-8265.11600 (PMC13161199; doi:10.15766/mep_2374-8265.11600)
Supplement: Supplementary file 1 — VA-ECMO Learner Handout.docxVV-ECMO Learner Handout.docxVA-ECMO Facilitator Guide.docxVV-ECMO Facilitator Guide.docxVA-ECMO Slides.pptxVV-ECMO Slides.pptxVA-ECMO Presurvey.docxVV-ECMO Presurvey.docxVA-ECMO Postsurvey.docxVV-ECMO Postsurvey.docx [file mep_2374-8265.11600-s001.zip › A. VA-ECMO Learner Handout.docx]

# **Low Flow on VA-ECMO**

## **This handout is used by learners during the VA-ECMO POGIL session and consists of prompts and questions to guide discussion, clinical reasoning, and reflection.**

## **Objectives**

1. Generate a differential diagnosis for low flow on VA-ECMO
2. Identify the clinical signs and parameters indicative of elevated afterload and low preload on VA-ECMO

**Case 1**

Alexa Carter is a 10-year-old female (weight 45 kg) who is admitted to the PICU for cardiac failure secondary to Rhinovirus/Enterovirus myocarditis. She ultimately required cannulation to VA-ECMO after epinephrine, norepinephrine, and vasopressin failed to improve her hemodynamic status. Echocardiogram demonstrated an ejection fraction of 11% with a rising lactate level.

- - What would be your cannulation strategy?

- - What are the risks and benefits of that cannulation strategy? (Fill in the table)

| **VA ECMO Cannulation** | **Benefits** | **Risks** |
| --- | --- | --- |
| Cervical cannulation |  |  |
| Femoral Cannulation |  |  |
| Central Cannulation |  |  |

She was electively cannulated to VA-ECMO with a 23 F right femoral venous and a 15 F right femoral arterial cannula. A distal limb reperfusion catheter was also placed in the cath lab.

| **Vitals** | T: 37C; HR: 111 bpm; BP: 101/92 mmHg; RR: 10 breaths/min; O_2_Sat: 94%; Cerebral NIRS: 55%; CVP: 9 mmHg |
| --- | --- |
| **Pressors** | Norepinephrine: 0.3 mcg/kg/min; Epinephrine: 0.2 mcg/kg/min; Vasopressin at 0.0008 U/kg/min |
| **ECMO**  **Circuit** | Flows: 84 mL/kg/min, Pin: -59 mmHg, Pout: 270 mmHg |
| **Ventilator Settings** | PC/PS Mode: PIP 20 cmH_2_O, PEEP 10 cmH_2_O, Rate 10, FiO_2_ 0.3 |
| **Pertinent Labs** | SvO_2_ 57%, Lactate 2.1, 7.29/71/43/22 |

Thirty minutes after VA ECMO support was initiated, the patient was repositioned. The ECMO circuit starts alarming, and you notice the following:

| **Vitals** | T: 37C; HR: 132 bpm; BP: 122/116 mmHg; RR: 10 breaths/min; O_2_Sat: 95%; Cerebral NIRS: 35% |
| --- | --- |
| **Pressors** | Norepinephrine: 0.3 mcg/kg/min; Epinephrine: 0.2 mcg/kg/min; Vasopressin at 0.0008 U/kg/min |
| **ECMO**  **Circuit** | Flows: 47 mL/kg/min, Pin: -40 mmHg and Pout: 290 mmHg, stable transmembrane gradient |
| **Pertinent Labs** | SvO_2_ 41%, Lactate 4.5, 7.21/88/44/19 |

- - What could the drop in her mixed venous saturation, oxygenation, and NIRS suggest in her case?

Remember: DO_2_ = CO x CaO_2_

CO~ VA ECMO Flows: Increase the flow.

O2 Saturation: Ensure adequate gas exchange.

Hemoglobin: Transfuse

- - What is the most likely etiology of the inadequate oxygen delivery in this case?

- - The inlet and outlet pressures have increased but the transmembrane pressure is stable. What does that indicate?


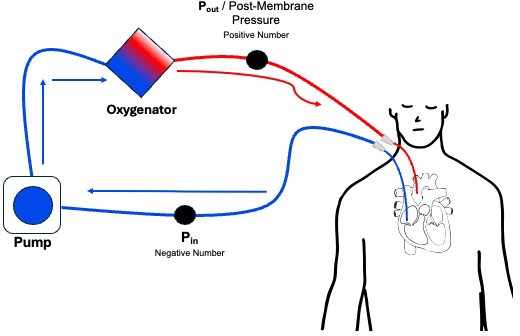
Author Owned Image

*In a low-flow state, always check the circuit pressures. They can help you pinpoint the problem.*

| **Change in Flow Rate** | **Pump Speed** | **Pump Inlet Pressure (Pin)** | **Post-membrane Pressure (Pout)** | **Cause** |
| --- | --- | --- | --- | --- |
| Decreased | Decreased | Increased/ more positive | Decreased | Pump Speed Reduced |
| Decreased | Same | Decreased/ more negative | Decreased | Preload is decreased (CVP decreased) |
| Decreased | Same | Increased/ more positive | Decreased | Resistance is building in artificial lungs (i.e. Clots) |
| Decreased | Same | Increased/ more positive | Increased | Afterload is increased (patient MAP or Cannula resistance) |

An echocardiography is performed and shows severely diminished left ventricular function, 15%; diminished right ventricular wall function, mild mitral and tricuspid regurgitation. The tip of the venous cannula is at the right atrial/ superior vena cava junction, and the infusion port is positioned in the right atrium.

- - Knowing that your physical exam has not changed, what is the most likely etiology of the increased afterload in this patient? How would you intervene?

As pressors are being weaned, her cyanosis and hypoxemia and subsequently her ECMO flows start improving. Her vitals improved: HR 98 bpm, BP 123/107, O_2_Sat 94% and SvO_2_ 60%. She is eventually weaned off pressors but remains hypertensive.

- - What is your next step?

## **Case 2**

Your next-door patient, Lenny Lewis, is a 6-month-old female, 7kg, who was cervically cannulated 5 hours ago to VA-ECMO after bradycardic arrest in the setting of ventricular dysfunction secondary to cardiomyopathy. She received 25 minutes of cardiopulmonary resuscitation prior to cannulation to VA-ECMO. Several hours later, she continues to demonstrate persistent (albeit better) lactic acidosis and widened arteriovenous difference of oxygen content (or AVDO_2_).

| **Vitals** | T: 37C; HR: 151 bpm; BP: 40/32 mmHg; RR: 12 breaths/min; O_2_Sat: 95%; Cerebral NIRS: 57%; Renal NIRS: 55% CVP: 12 mmHg |
| --- | --- |
| **Drips** | Milrinone: 0.3 mcg/kg/min, Heparin 15u/kg/hr, Fentanyl 1.2 mcg/kg/hr |
| **ECMO Circuit** | Flows: 64 mL/kg/min, Pin and Pout higher than expected |
| **Ventilator Settings** | SIMV PC/PS mode: PIP 24 cmH_2_O, PEEP 8 cmH_2_O, Rate 12, FiO_2_ 0.4 |
| **Physical Exam** | Intubated, sedated, no murmur, crackles bilaterally, extremities cool distally and warm centrally, capillary refill ~ 3-4 seconds. |
| **Pertinent Labs** | Normal BMP. Hb 11 g/dL, aPTT 60s, INR 1.8, Patient gas: 7.38/38/100/23 |

- What does a widened AVDO_2_ indicate? How do you calculate it?
- Is the oxygen delivery adequate in this case? How can you tell?
- What is the most likely etiology of the low-flow state?
- What is leading to excessive afterload in her case?
- What would be your next intervention?

The Chest X-ray shows that the cannulas are in an appropriate and stable position, with stable mild to moderate pulmonary edema and stable moderate cardiomegaly. Her echo is also stable. The surgical team comes to the bedside and recognizes that the arterial cannula is too small. They would like to upsize it.

- - What should you be planning for prior to the cannula switch?

## **Case 3**

Jack Hunter is a 5-year-old who was admitted two weeks ago with ARDS and septic shock and was eventually cannulated to femoral VA-ECMO. This morning, the team has started titrating his heparin infusion up due to an increasing amount of fibrin in his circuit. Over the last 4 hours, he developed progressive hypotension, tachycardia, and abdominal distention. You are called to the bedside because the ECMO specialist is having trouble maintaining the flow.

| **Vitals** | T: 37C; HR: 165 bpm; BP: 60/50 mmHg; RR: 10 breaths/min; O_2_Sat: 95%; Cerebral NIRS: 50%; CVP: 2 mmHg |
| --- | --- |
| **Drips** | Fentanyl 2 mcg/kg/hr, Precedex 0.7 mcg/kg/hr, Heparin 45u/kg/hr |
| **ECMO Circuit** | Flows: 61 mL/kg/min, Adequate RPM, Pin dropped from -60 to -82 and Pout is decreased. |
| **Ventilator Settings** | PC Mode, PIP 20 cmH_2_O, PEEP 10 cmH_2_O, Rate 10, FiO_2_ 0.3, Tidal Volume 4.5mL/kg |
| **Physical Exam** | Intubated, sedated, tachycardic, decreased breaths sounds, minimal chest rise, abdomen distended and taught, extremities cool distally, capillary refill 4 seconds. |
| **Pertinent Labs** | Normal electrolytes, Cr 0.6, BUN 25, Hb 5.4 g/dL, aPTT 75s, Anti-Xa: 0.67 IU/mL, INR 2.7, ABG 7.25/50/45/16 |

- Is the oxygen delivery adequate in this case?
  - What is the most likely etiology of the low-flow state?

You noticed worsening abdominal distention, decreased urine output, and a bladder pressure (when paralyzed) of 22 mmHg. Bright red blood comes out of his NG tube, and the ECMO specialist informs you that there was a transient improvement in pump flows and inlet pressures with prior volume administration.

- - What is your next intervention?
