# Supplementary material for: Enhancing Pediatric Extracorporeal Membrane Oxygenation Education Through Process-Oriented Guided Inquiry Learning Sessions for Fellows and Advanced Practice Providers
Source: MedEdPORTAL. 2026 May 12;22:11600. doi: 10.15766/mep_2374-8265.11600 (PMC13161199; doi:10.15766/mep_2374-8265.11600)
Supplement: Supplementary file 1 — VA-ECMO Learner Handout.docxVV-ECMO Learner Handout.docxVA-ECMO Facilitator Guide.docxVV-ECMO Facilitator Guide.docxVA-ECMO Slides.pptxVV-ECMO Slides.pptxVA-ECMO Presurvey.docxVV-ECMO Presurvey.docxVA-ECMO Postsurvey.docxVV-ECMO Postsurvey.docx [file mep_2374-8265.11600-s001.zip › B. VV-ECMO Learner Handout.docx]

**Hypoxemia on VV-ECMO**

**This handout is used by learners during the VV-ECMO POGIL session and consists of prompts and questions to guide discussion, clinical reasoning, and reflection.**

**Objectives**

1. Generate a differential diagnosis for hypoxemia in patients on VV-ECMO
2. Identify clinical signs and parameters indicative of oxygenator failure and decreased preload as an etiology of hypoxemia on VV-ECMO.
3. Identify clinical signs and parameters indicative of recirculation on VV-ECMO and generate a differential diagnosis for recirculation.

**Case 1**

Rebecca Nelson is a 14-year-old 50 kg female who was intubated for acute hypoxemic respiratory failure secondary to trauma from an automobile vs pedestrian accident with blunt force trauma to the chest. She required placement of bilateral chest tubes secondary to air leak and pulmonary contusions. She eventually developed ARDS with worsening hypoxemia and acidosis; it was determined that she necessitated elective cannulation to VV-ECMO.

- What are the options for VV-ECMO cannulation? And what would be the best strategy for this patient?

| **Cannulation strategy** | **Location** | **Benefits** | **Risks** |
| --- | --- | --- | --- |
| Cervical with a single dual lumen cannula (Bicaval) |  |  |  |
| Multisite: Cervical-Femoral (Typically) |  |  |  |

She was cannulated to VV-ECMO with a single 27 French dual lumen cannula 72 hours ago. There was significant bleeding from chest tubes initially, but it has decreased, and no evidence of ongoing air leak. Below are the pertinent numbers shortly after cannulation:

| **Vitals** | T: 36.5C; HR: 96 bpm; BP: 105/50 mmHg; RR: 20 breaths/min; O_2_Sat: 89%; SvO_2_: 65%; CVP: 8 mmHg |
| --- | --- |
| **Infusions** | Fentanyl 2mcg/kg/hour; Midazolam 0.1mg/kg/hr, Heparin 22units/kg/hr |
| **ECMO Circuit** | Flows: 50 mL/kg/min, Pin: -51 mmHg, Pout: 155 mmHg, FiO_2_: 1.0, Sweep: 4.5L/min |
| **Ventilator Settings** | PRVC Mode: TV:8mL/kg, PIP 38 cmH_2_O, PEEP 14 cmH_2_O, Rate 20, FiO_2_ 0.75 |
| **Pertinent Labs** | WBC 7x10^3^/mL; Hb 8g/dL, Plt 94x10^3^/mL; Electrolytes: within normal limits, Anti-Xa 0.5 IU/mL; INR 2; aPTT: 65; Patient ABG: 7.23/65/45/20; Lactate 3.2 mmol/L; Post-oxygenator ABG: 7.3/53/350/18 |

- What should be your next step? And why?

The ventilator settings are adjusted. Over the next two days, some bleeding has been noted at the cannulation site, central venous line site, and chest tube. Fibrin stranding and a clot were noticed in the ECMO circuit at the connectors. The patient requires more frequent transfusions of blood products and platelets. The bedside nurses are mainly concerned of her oxygen saturation that has been down trending the last six hours and is now 76%. Her SvO_2_ has also dropped to 55%. Her Hb is now 8.5 g/dL.

- What is your differential diagnosis for hypoxemia in a patient on VV-ECMO?

The ECMO specialist has updated you, indicating that the patient's oxygen saturation continues to decrease, now in the low 70s range, despite increasing the ECMO flows. Additionally, she has observed an upward trend in the patient's lactate level, which is now at 5.3 mmol/L, and a decrease in the patient’s SvO_2_ to 43%.

- Is the patient’s oxygen delivery adequate?

DO_2_ = CO x CaO_2_

- What would be the next step in attempting to determine the etiology of the patient's hypoxemia?

You are informed that the ECMO flows have begun to decline. Additionally, you are told that the transmembrane gradient is elevated, and the post-oxygenator gas is 7.29/68/85/21.

- What is the most likely cause of the patient’s hypoxemia in this case?
- What other signs would you expect to see in the ECMO circuit if the cause of hypoxemia was oxygenator failure?
- What should be your next step, and how should you plan for it?

**Case 2**

Virginia Baker is a 2-year-old, 12 kg female, admitted with respiratory failure secondary to Rhinovirus/Enterovirus, with secondary Streptococcus Pneumoniae ARDS. She failed conventional and HFOV mechanical ventilation and was placed on VV ECMO with a 16 F dual lumen cannula 1 hour ago. She has been transitioned from the oscillator to rest ventilator settings. Patient was stable in the cath lab with oxygen saturations of 89%. On arrival to the PICU:

| **Vitals** | T: 36.5C; HR: 135 bpm; BP: 95/52 mmHg; RR: 10 breaths/min; O_2_Sat: 86%; SvO_2_: 77%, CVP 11 mmHg |
| --- | --- |
| **Infusions/**  **Medications** | Fentanyl 2mcg/kg/hour; Midazolam 0.1mg/kg/hour, Heparin 25units/kg/hr |
| **ECMO Circuit** | Flows: 80 mL/kg/min, Pin: -30 mmHg, Pout: 145mmHg, FiO_2_: 1.0, Sweep 3.5 L/min |
| **Ventilator Settings** | SIMC-PC Mode: PIP 20 cmH_2_O, PEEP 10 cmH_2_O, Rate 10, FiO_2_ 0.4 |
| **Physical Exam** | Intubated, awake, pupils equal and sluggish, Diffuse crackles bilaterally, Abdomen soft, cap refill 2 sec |
| **Pertinent Labs** | WBC 5x10^3^/mL; Hb 10g/dL, Plt 206x10^3^/mL; Electrolytes: within normal limits; Cr: 0.16 mg/dL; aPTT 72s, INT 1.3 |


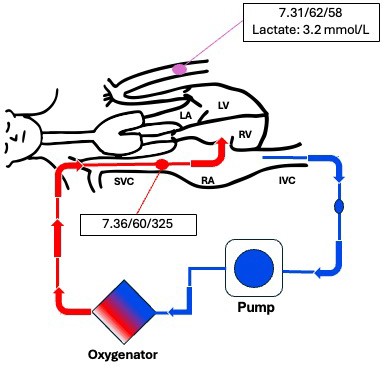


Author Owned Image

Shortly after arrival to the PICU, her Fentanyl drip was switched to Morphine drip, and she was started on a Cisatracurium drip due to agitation. You are called to the bedside because her saturation has dropped to 71% despite increasing the ECMO flow.

| **Vitals** | T: 37.7C; HR: 150 bpm; BP: 85/44 mmHg; RR: 10 breaths/min; O_2_Sat: 71%; SvO_2_: 90% |
| --- | --- |
| **Infusions/**  **Medications** | Morphine 0.1mg/kg/hour; Midazolam 0.1mg/kg/hour, Cisatracurium: 0.12 mg/kg/hour, Heparin 25units/kg/hour |
| **ECMO Circuit** | Flows: 100 mL/kg/min, Pin: -55mmHg, Pout: 145mmHg, FiO_2_: 1.0 |
| **Ventilator Settings** | SIMC-PC Mode: PIP 20 cmH_2_O, PEEP 10 cmH_2_O, Rate 10, FiO_2_ 0.4 |
| **Pertinent Labs** | Patient ABG:7.21/88/44/19; Lactate 4.1 mmol/L; Post-oxygenator ABG: 7.36/58/350/21 |

- Are the circuit flows and oxygenators the source of the patient’s hypoxemia?
- Is the patient’s oxygen delivery adequate?

Below are your new blood gases:

Author Owned Image

- What do you suspect as a possible etiology of your patient’s hypoxemia?
- What is your next step?

The ECMO specialist notes that there is no color differential between the two cannulas. The patient’s CXR is significant for bilateral lung consolidation, with the left lung more affected than the right. The endotracheal tube and ECMO cannula are in good position. Her echocardiography showed normal left ventricular function with an ejection fraction of 62%. The cannula position shows the tip in the IVC, and the return jet is turbulent and directed toward the atrial wall.

- Why are the turbulence of the jet and its location near the cannula tip a problem?
- What is your plan of action?

**Case 3**

Rosa Franklin is a 3-year-old, 15 kg female with subacute autoimmune hepatitis, admitted with acute respiratory failure secondary to influenza A pneumonia. She was cannulated to VV ECMO with a 19 French dual lumen cannula for persistent hypoxemia. Her cannulation was notable for initial bleeding at the cannulation site that resolved with plasma and platelet infusions in the first 24 hours.

This is day 2 of VV ECMO (hour 36). Overnight, she was difficult to sedate but now seems to have adequate analgesia and sedation on morphine and Precedex infusions, respectively. Her transaminases, coagulation studies, and ammonia are elevated. The ECMO specialist notifies you that she is starting to ‘ooze significantly’ from a variety of sites.

| **Vitals** | T: 36.5C; HR: 150 bpm; BP: 85/32 mmHg; RR: 10 breaths/min; O_2_Sat: 85%; SvO_2_: 45%, CVP 10 mmHg |
| --- | --- |
| **Infusions/**  **Medications** | Morphine 1mg/kg/hour; Dexmedetomidine 0.5 mcg/kg/hour, Bivalirudin 0.35mg/kg/hour |
| **ECMO Circuit** | Flows: 80 mL/kg/min, Pin: -30 mmHg, Pout: 175mmHg, FiO_2_: 1.0, Sweep 2L/min |
| **Ventilator Settings** | SIMC-PC Mode: PIP 20 cmH_2_O, PEEP 10 cmH_2_O, Rate 10, FiO_2_ 0.3 |
| **Physical Exam** | Intubated, sedated, thin, pupils equal and sluggish, sinus tachycardia, fine coarse breath sounds bilaterally, Abdomen distended, Liver 6 cm below RCM and firm, cap refill 2 sec, oozing from CVC, art line and cannula sites |
| **Pertinent Labs** | WBC 12x10^3^/mL; Hb 7.5 g/dL, Plt 116x10^3^/mL; Electrolytes: within normal limits; Cr: 0.14 mg/dL; AST 2600 U/L; ALT 2855 U/L; Alb 2.8 g/dL; total Bili 4 mg/dL; aPTT 120s; INR 2.4; Pump ABG: 7.45/35/483/24; Patient ABG: 7.35/45/40/24, Lactate: 4.1 |

- Is the patient’s oxygen delivery adequate?
- How would you approach the patient’s hypoxemia?

The CXR shows diffuse bilateral infiltrates with a paucity of aeration. No pneumothorax, but she has now developed a right-sided large effusion. No change in cannula position. The ECMO specialist informs you that after increasing the ECMO flows to 100mL/kg/min, the patient’s SaO_2_ dropped to 75%, and the patient’s SvO_2_ increased to 75%.

- What does that intervention suggest?

The patient's nurse is concerned that the dressing on her neck is soaked with blood. She also mentioned that the patient's numbers are changing rapidly and drastically, and they are having a harder time maintaining the ECMO flow:

| **Vitals** | T: 36.5C; HR: 160 bpm; BP: 65/34 mmHg; RR: 10 breaths/min; O_2_Sat: 79%; SvO_2_: 60%, CVP 4 mmHg |
| --- | --- |
| **ECMO Circuit** | Flows: 60 mL/kg/min, Pin: -60 mmHg, Pout: 165mmHg, FiO_2_: 1.0 |
| **Physical Exam** | Cooler extremities with a capillary refill of 6 seconds, few disrupted sutures are noted at the cannula site with ongoing bleeding, mottled throughout |

- What is the most likely etiology of the patient’s hypoxemia? What would be your next steps?

Below are some critical lab results: hemoglobin 5, platelet count 60K, INR 3. Point of care arterial blood gas: 7.11/68/35/14, lactate 5.1, ionized Calcium 1.1.

- What would be your plan of action?
