# Supplementary material for: Enhancing Pediatric Extracorporeal Membrane Oxygenation Education Through Process-Oriented Guided Inquiry Learning Sessions for Fellows and Advanced Practice Providers
Source: MedEdPORTAL. 2026 May 12;22:11600. doi: 10.15766/mep_2374-8265.11600 (PMC13161199; doi:10.15766/mep_2374-8265.11600)
Supplement: Supplementary file 1 — VA-ECMO Learner Handout.docxVV-ECMO Learner Handout.docxVA-ECMO Facilitator Guide.docxVV-ECMO Facilitator Guide.docxVA-ECMO Slides.pptxVV-ECMO Slides.pptxVA-ECMO Presurvey.docxVV-ECMO Presurvey.docxVA-ECMO Postsurvey.docxVV-ECMO Postsurvey.docx [file mep_2374-8265.11600-s001.zip › C. VA-ECMO Facilitator Guide.docx]

# **Low Flow on VA-ECMO**

| **Facilitator Actions** | **Time** | **Slides** | **Materials** | **Notes** |
| --- | --- | --- | --- | --- |
| Introduction and session setup:   - Explain POGIL format - Review objectives - Divide learners into groups of 2–5 - Distribute learner handouts. - Complete Pre-survey if able | 5 min | 1–3 | Learner Handout, Facilitator Guide, PowerPoint, Pre-survey |  |
| Case 1 introduction:   - Summarize patient presentation and prompt learners to identify key clinical problems. | 2 min | 4 | PowerPoint | - What problem is VA-ECMO intended to solve here? |
| Cannulation strategy discussion:   - Have groups select a VA-ECMO cannulation strategy and discuss risks and benefits. | 7 min | 5–7 | Learner Handout table |  |
| Review vitals and ECMO data:   - Allow learners to identify concerning parameters. | 3 min | 8–11 | PowerPoint | - Which value concerns you most, and why? - Emphasize trends over isolated numbers |
| Oxygen delivery review:   - Discuss SvO₂, lactate, NIRS - Review DO₂ = CO × CaO₂. | 3 min | 12 | Optional whiteboard | - Difficult concept: Total CO on VA-ECMO = ECMO flow + native CO. |
| Differential diagnosis of low flow:   - Guide learners to consider preload, afterload, and circuit-related causes. | 5 min | 13–15 | Low Flow Diagram (Slides and facilitator handout) | - Is this preload, afterload, or circuit related? - What data argue against hypovolemia? |
| Circuit pressure interpretation:   - Review Pin, Pout, and transmembrane pressure patterns using the figure. - Review how pressures are affected by various problems | 5 min | 17 | Circuit pressure figure and table |  |
| Apply findings to Case 1:   - Identify increased afterload and discuss pressor weaning and SVR reduction. | 3 min | 18–19 | PowerPoint | - Difficult concept: High afterload limits flow despite adequate RPMs. |
| Framework summary for Case 1:   - Reinforce structured approach to low flow on VA-ECMO. | 2 min | 20 | PowerPoint | - Important point: Always check circuit pressures before adjusting pump speed. |
| Case 2 application:   - Learners apply the same low-flow framework to a new scenario with minimal facilitator guidance. | 12 min | 21–29 | Learner Handout, slides | - Using the same framework, what is different here? - Allow learners to struggle productively; intervene sparingly. |
| Case 3 application:   - Learners again apply the framework, focusing on preload limitation and hemorrhage. | 8 min | 29–34 | Learner Handout, slides | - Difficult concept: Hemorrhage and abdominal compartment syndrome impair venous return. |
| Wrap-up and post-session tasks:   - Review key take-home points - Complete post-survey. | 5 min | 35–37 | Slides, handouts, Post-survey | Review the framework:   - DO_2_ inadequate? - Is it a preload or afterload issue? - Narrow it down to a specific problem |

**Introduction: 5 min/Slides 1-3**

**Introduce the session and its objectives. The first 5 minutes can also be used to complete the pre-survey:**

***This session follows the POGIL (Process-Oriented Guided Inquiry Learning) format, in which learners work in small groups to build their own understanding of concepts through guided inquiry. You will be divided into groups of 2-5 people. The session consists of three cases. In the first case, we will introduce a framework or approach to the problem of low flow on VA-ECMO. In the following two cases, you will apply this approach to assess and manage related issues. I will guide you through the process.***

## **Objectives**

1. Generate a differential diagnosis for low flow on VA-ECMO
2. Identify the clinical signs and parameters indicative of elevated afterload and low preload on VA-ECMO

**Case 1: 30 min/ Slides 4-20**

Alexa Carter is a 10-year-old female (weight 45 kg) who is admitted to the PICU for cardiac failure secondary to Rhinovirus/Enterovirus myocarditis. She ultimately required cannulation to VA-ECMO after epinephrine, norepinephrine, and vasopressin failed to improve her hemodynamic status. Echocardiogram demonstrated an ejection fraction of 11% with a rising lactate level.

**7 min/Slides 5-7: *We encourage taking time to review cannulation strategies, including their risks and benefits. This provides a strong introduction and/or recap, as cannulation strategy is a critical consideration in patient management.***

- - What would be your cannulation strategy?

Femoral or cervical cannulation

- - What are the risks and benefits of that cannulation strategy? (Fill in the table)

| **VA ECMO Cannulation** | **Benefits** | **Risks** |
| --- | --- | --- |
| Cervical cannulation | - Seldinger or cut down Larger vessels for flow Easier for early mobilization | - Need retrograde coronary flow Risk for ischemic stroke Permanent vessel damage Poor LV decompression |
| Femoral Cannulation | - Seldinger or cut down Easier during compressions | - Flow limitations in children Vessels too small if <20-25 kg Poor LV decompression - Poor mobility - Competing CO and ECMO flow Limb ischemia |
| Central Cannulation | - High flows - Better venous drainage and cardiac decompression | - Sternotomy - High risk for bleeding Mediastinitis - Less mobile (with open chest) |

**3 min/ Slides 8-11- *Allow learners a couple of minutes to review the values of both sets of vitals and highlight any that raise concern.***

She was electively cannulated to VA-ECMO with a 23 F right femoral venous and a 15 F right femoral arterial cannula. A distal limb reperfusion catheter was also placed in the cath lab.

| **Vitals** | T: 37C; HR: 111 bpm; BP: 101/92 mmHg; RR: 10 breaths/min; O_2_Sat: 94%; Cerebral NIRS: 55%; CVP: 9 mmHg |
| --- | --- |
| **Pressors** | Norepinephrine: 0.3 mcg/kg/min; Epinephrine: 0.2 mcg/kg/min; Vasopressin at 0.0008 U/kg/min |
| **ECMO**  **Circuit** | Flows: 84 mL/kg/min, Pin: -59 mmHg, Pout: 270 mmHg |
| **Ventilator Settings** | PC/PS Mode: PIP 20 cmH_2_O, PEEP 10 cmH_2_O, Rate 10, FiO_2_ 0.3 |
| **Pertinent Labs** | SvO_2_ 57%, Lactate 2.1, 7.29/71/43/22 |

Thirty minutes after VA ECMO support was initiated, the patient was repositioned. The ECMO circuit starts alarming, and you notice the following:

| **Vitals** | T: 37C; HR: 132 bpm; BP: 122/116 mmHg; RR: 10 breaths/min; O_2_Sat: 95%; Cerebral NIRS: 35% |
| --- | --- |
| **Pressors** | Norepinephrine: 0.3 mcg/kg/min; Epinephrine: 0.2 mcg/kg/min; Vasopressin at 0.0008 U/kg/min |
| **ECMO**  **Circuit** | Flows: 47 mL/kg/min, Pin: -40 mmHg and Pout: 290 mmHg, stable transmembrane gradient |
| **Pertinent Labs** | SvO_2_ 41%, Lactate 4.5, 7.21/88/44/19 |

- - What could the drop in her mixed venous saturation, oxygenation, and NIRS suggest in her case? **(3 min/ Slide 12 - *Spend a minute or so reviewing the oxygen delivery formula and how it applies to a patient on VA-ECMO: the ECMO flow plus the native cardiac output (if present) is equal to the patient’s total cardiac output***.

A decrease in SvO_2_, especially with an increase in lactate, suggests inadequate oxygen delivery.

Remember: DO_2_ = CO x CaO_2_

CO~ VA ECMO Flows: Increase the flow.

O2 Saturation: Ensure adequate gas exchange.

Hemoglobin: Transfuse

- - What is the most likely etiology of the inadequate oxygen delivery in this case? **(5 min/ Slides 13-15)**

In her case, it is likely due to suboptimal ECMO flows in the setting of worsening hypertension. Our patient’s CVP and high BP are not suggestive of inadequate preload. Could attempt to increase RPMs to improve her flow. However, increasing RPM at low ECMO flow rates may lead to shearing and hemolysis.

- - The inlet and outlet pressures have increased but the transmembrane pressure is stable. What does that indicate? **(1 min/ Slide 16)**

Increase afterload in the patient. An unchanged transmembrane pressure indicates no change in the oxygenator.

**5 min/ Slide 17: *Take a moment to review the figure with the learners, followed by the table below. The figure introduces the different circuit pressures to help learners become familiar with them. The table illustrates how various problems affect these pressures.***


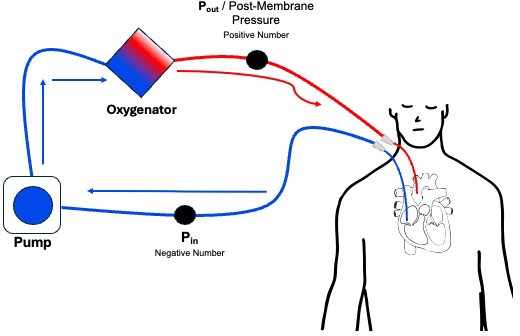
Author Owned Image

*In a low-flow state, always check the circuit pressures. They can help you pinpoint the problem.*

| **Change in Flow Rate** | **Pump Speed** | **Pump Inlet Pressure (Pin)** | **Post-membrane Pressure (Pout)** | **Cause** |
| --- | --- | --- | --- | --- |
| Decreased | Decreased | Increased/ more positive | Decreased | Pump Speed Reduced |
| Decreased | Same | Decreased/ more negative | Decreased | Preload is decreased (CVP decreased) |
| Decreased | Same | Increased/ more positive | Decreased | Resistance is building in artificial lungs (i.e. Clots) |
| Decreased | Same | Increased/ more positive | Increased | Afterload is increased (patient MAP or Cannula resistance) |

An echocardiography is performed and shows severely diminished left ventricular function, 15%; diminished right ventricular wall function, mild mitral and tricuspid regurgitation. The tip of the venous cannula is at the right atrial/ superior vena cava junction, and the infusion port is positioned in the right atrium.

- - Knowing that your physical exam has not changed, what is the most likely etiology of the increased afterload in this patient? How would you intervene? **(2 min / Slide 18)**

Based upon the elevation of arterial circuit pressures and stable transmembrane gradient, the afterload seems to be secondary to patient hypertension. You should start weaning her pressors while trying to gather additional information. Would start weaning vasopressin and norepinephrine infusions to decrease SVR. Would not turn off Epi completely.

Her echo showed that the cannulas are in good position and there is no evidence of thrombus in the cannulas.

As pressors are being weaned, her cyanosis and hypoxemia and subsequently her ECMO flows start improving. Her vitals improved: HR 98 bpm, BP 123/107, O_2_Sat 94% and SvO_2_ 60%. She is eventually weaned off pressors but remains hypertensive.

- - What is your next step? **(1 min / Slide 19 – Point out the importance of SVR reduction agents)**

Would consider starting an SVR reduction agent (eg, Milrinone vs. Nicardipine/Nipride), especially if her hypertension continues to interfere with her ECMO flows. In her case, we should consider starting Milrinone and Nicardipine.

**3 min / Slide 20 – Review the framework:**

## **Case 2:** **12 min/Slides 21-29**

**2 min/ Slide 22- *Allow learners a couple of minutes to review the values of both sets of vitals and highlight any that raise concern.***

Your next-door patient, Lenny Lewis, is a 6-month-old female, 7kg, who was cervically cannulated 5 hours ago to VA-ECMO after bradycardic arrest in the setting of ventricular dysfunction secondary to cardiomyopathy. She received 25 minutes of cardiopulmonary resuscitation prior to cannulation to VA-ECMO. Several hours later, she continues to demonstrate persistent (albeit better) lactic acidosis and widened arteriovenous difference of oxygen content (or AVDO_2_).

| **Vitals** | T: 37C; HR: 151 bpm; BP: 40/32 mmHg; RR: 12 breaths/min; O_2_Sat: 95%; Cerebral NIRS: 57%; Renal NIRS: 55% CVP: 12 mmHg |
| --- | --- |
| **Drips** | Milrinone: 0.3 mcg/kg/min, Heparin 15u/kg/hr, Fentanyl 1.2 mcg/kg/hr |
| **ECMO Circuit** | Flows: 64 mL/kg/min, Pin and Pout higher than expected |
| **Ventilator Settings** | SIMV PC/PS mode: PIP 24 cmH_2_O, PEEP 8 cmH_2_O, Rate 12, FiO_2_ 0.4 |
| **Physical Exam** | Intubated, sedated, no murmur, crackles bilaterally, extremities cool distally and warm centrally, capillary refill ~ 3-4 seconds. |
| **Pertinent Labs** | Normal BMP. Hb 11 g/dL, aPTT 60s, INR 1.8, Patient gas: 7.38/38/100/23 |

- What does a widened AVDO_2_ indicate? How do you calculate it? (**4 min/ Slide 23- Fundamental *concept)***

AVDO_2_, the arteriovenous difference of oxygen content, is the difference between CaO_2_ (arterial oxygen content) and CvO_2._ (venous oxygen content). According to the Fick equation, oxygen consumption is directly proportional to the patient’s metabolic rate and energy expenditure. For simplicity, an approximate systemic AVDO_2_ can be calculated as the difference between SaO_2_ and SvO_2_. A typical range of AVDO_2_ is 4-8 O_2_/dL when using Fick or approximately 20-25% when calculating using SaO_2_ and SvO_2_. Organ-specific oxygen extraction can also be calculated, but is not routinely used in clinical practice. Below is the approximate normal oxygen consumption of different organs:

| Brain | Heart | Liver | Kidney | Skeletal Muscles |
| --- | --- | --- | --- | --- |
| 18% | 11% | 20% | 7% | 20% |

A wide AVDO_2_ suggests a higher O_2_ extraction due to a decrease in DO_2_ or an increase in VO_2_.

A narrow or lower AVDO_2_ indicates a decreased O_2_ extraction.

A balance between DO_2_ and VO_2_ achieves adequate tissue oxygenation.

- Is the oxygen delivery adequate in this case? How can you tell? (**1 min/ Slide 24)**

No, oxygen delivery is suboptimal in this case.

DO_2_ = CO x CaO_2_

- - - CaO_2_ is likely normal in her case: Hb, PaO_2,_ and O_2_ Sat are all normal
    - CO ~ VA-ECMO flow is low
    - The ultimate measure of adequate tissue O_2_ delivery is markers of tissue perfusion, e.g., acidosis, lactate, widened AVDO_2_, and low NIRS.
- What is the most likely etiology of the low-flow state? (**2 min/ Slide 25)**

Her pump flows are inadequate, which is likely the cause of her hypotension. Again, this could be due to:

1. Inadequate preload: however, her CVP is within normal limits, and in a hypovolemic state, we would expect her Pin to be excessively negative and P out to be low.
2. Inadequate RPMs: There is no specific number to aim for but if preload or afterload are not adequate you will not get increasing flow from increasing RPMs (venous pressure will drop/circuit chatter if preload low, increasing Pin/Pout pressures without increasing flow if afterload high). That indicates that pump RPMs are not the issue.
3. A high afterload state, the most likely etiology of her decreased flows, could explain the elevated circuit pressures.

- What is leading to excessive afterload in her case? (**1 min/ Slide 26)**

Her increased afterload is likely due to an issue with the arterial cannula (No seizures, well sedated, increased both Pin and Pout with a stable transmembrane pressure makes it less likely to be due to clots in the oxygenator, she is hypotensive rather than hypertensive). Another hint is that she has had inadequate flows and persistent high circuit pressures with no improvement since cannulation.

- What would be your next intervention? (**1 min/ Slide 27)**

Obtain a chest x-ray and an echo. Would consider activating the surgical team for the cannula issue (obstruction vs. malposition vs. too small)

The Chest X-ray shows that the cannulas are in an appropriate and stable position, with stable mild to moderate pulmonary edema and stable moderate cardiomegaly. Her echo is also stable. The surgical team comes to the bedside and recognizes that the arterial cannula is too small. They would like to upsize it.

- - What should you be planning for prior to the cannula switch? (**1 min/ Slide 28)**

You should plan how to support the patient’s circulation during arterial cannula change, including the use of inotropic support and the possible need for cardiopulmonary resuscitation.

## **Case 3: 8 min/ Slides 29-34**

**2 min/ Slide 22- *Allow learners a couple of minutes to review the values of both sets of vitals and highlight any that raise concern.***

Jack Hunter is a 5-year-old who was admitted two weeks ago with ARDS and septic shock and was eventually cannulated to femoral VA-ECMO. This morning, the team has started titrating his heparin infusion up due to an increasing amount of fibrin in his circuit. Over the last 4 hours, he developed progressive hypotension, tachycardia, and abdominal distention. You are called to the bedside because the ECMO specialist is having trouble maintaining the flow.

| **Vitals** | T: 37C; HR: 165 bpm; BP: 60/50 mmHg; RR: 10 breaths/min; O_2_Sat: 95%; Cerebral NIRS: 50%; CVP: 2 mmHg |
| --- | --- |
| **Drips** | Fentanyl 2 mcg/kg/hr, Precedex 0.7 mcg/kg/hr, Heparin 45u/kg/hr |
| **ECMO Circuit** | Flows: 61 mL/kg/min, Adequate RPM, Pin dropped from -60 to -82 and Pout is decreased. |
| **Ventilator Settings** | PC Mode, PIP 20 cmH_2_O, PEEP 10 cmH_2_O, Rate 10, FiO_2_ 0.3, Tidal Volume 4.5mL/kg |
| **Physical Exam** | Intubated, sedated, tachycardic, decreased breaths sounds, minimal chest rise, abdomen distended and taught, extremities cool distally, capillary refill 4 seconds. |
| **Pertinent Labs** | Normal electrolytes, Cr 0.6, BUN 25, Hb 5.4 g/dL, aPTT 75s, Anti-Xa: 0.67 IU/mL, INR 2.7, ABG 7.25/50/45/16 |

- Is the oxygen delivery adequate in this case? **(2 min/ Slide 32)**

The hypotension, narrow pulse pressure, low SvO_2,_ and poor perfusion on exam are indicative of inadequate oxygen delivery and tissue perfusion.

DO_2_ = CO x CaO_2_

- - - Hb is low, likely due to intra-abdominal and maybe pulmonary hemorrhage in the setting of anticoagulation.
    - PaO_2_ = 45 mmHg, which is low
    - CO ~ VA-ECMO suboptimal
  - What is the most likely etiology of the low-flow state? **(2 min/ Slide 33)**
    The low flow state is likely due to inadequate preload: CVP is low, Pin is more negative, and Pout is also lower. The drop in hemoglobin in the setting of anticoagulation, with abdominal distention and minimal chest rise/decrease breath sounds, is likely suggestive of a hemorrhage.

Abdominal compartment syndrome should be on the differential in this case, where you would expect to see impaired venous return with very low pump flow and low to stable CVP. You would also notice worsening of the abdominal exam and a decrease to no urine output.

You noticed worsening abdominal distention, decreased urine output, and a bladder pressure (when paralyzed) of 22 mmHg. Bright red blood comes out of his NG tube, and the ECMO specialist informs you that there was a transient improvement in pump flows and inlet pressures with prior volume administration.

- - What is your next intervention? **(2 min/ Slide 34)**

Your patient on ECMO is bleeding. Consider:

- - - Transfusion of PRBCs, platelets, and FFPs, and you might still need to give fluid boluses.
    - Urgent surgery consult for abdominal decompression
    - Decreasing/ discontinuing heparin infusion
    - Increasing platelet transfusion threshold
    - Frequent coagulation studies

**Wrap up: 5 min/Slides 35-37:**

***Review the framework.***

***Ask if there are any questions, and allow learners to spend the final five minutes completing the post-survey***
