# Supplementary material for: Enhancing Pediatric Extracorporeal Membrane Oxygenation Education Through Process-Oriented Guided Inquiry Learning Sessions for Fellows and Advanced Practice Providers
Source: MedEdPORTAL. 2026 May 12;22:11600. doi: 10.15766/mep_2374-8265.11600 (PMC13161199; doi:10.15766/mep_2374-8265.11600)
Supplement: Supplementary file 1 — VA-ECMO Learner Handout.docxVV-ECMO Learner Handout.docxVA-ECMO Facilitator Guide.docxVV-ECMO Facilitator Guide.docxVA-ECMO Slides.pptxVV-ECMO Slides.pptxVA-ECMO Presurvey.docxVV-ECMO Presurvey.docxVA-ECMO Postsurvey.docxVV-ECMO Postsurvey.docx [file mep_2374-8265.11600-s001.zip › D. VV-ECMO Facilitator Guide.docx]

**Hypoxemia on VV-ECMO**

| Facilitator Actions | Time | Slides | Materials | Notes |
| --- | --- | --- | --- | --- |
| Introduction:   - Explain POGIL format - Review objectives - Divide learners into groups of 2–5 - Distribute handouts. | 5 min | 1–3 | Handouts, slides, pre-survey |  |
| Case 1 introduction:   - Summarize clinical context and indication for VV-ECMO. | 1. min | 4–5 | Slides | - What problem is VV-ECMO intended to solve in this patient? |
| VV-ECMO cannulation strategies discussion. | 5 min | 6–7 | Handout table | - What are the trade-offs of a dual-lumen cannula? - How might cannula choice affect recirculation? |
| Review initial vitals, ventilator, and ECMO parameters. | 3 min | 8 | Slides | - Focus on trends not isolated numbers |
| Ventilator management on VV-ECMO | 3 min | 8 | Slides | - Emphasize minimizing ventilator-induced lung injury |
| Differential diagnosis of hypoxemia on VV-ECMO. | 7 min | 9–11 | Slides |  |
| Oxygen delivery review and assessment of adequacy. | 4 min | 12 | Whiteboard optional | - Difficult concept: On VV-ECMO, cardiac output ≠ ECMO flow. - Why can a high SvO₂ be misleading? |
| Diagnostic approach to worsening hypoxemia. | 3 min | 12–13 | Slides | - Walk through determinants sequentially |
| Identification of oxygenator failure and planning for circuit intervention. | 4 min | 14–15 | Slides | - Take-home: Rising transmembrane pressure with poor post-oxygenator gases suggests oxygenator failure. |
| Case 2 application: Recirculation on VV-ECMO.   - Review the definition of recirculation - Review parameters indicative of recirculation - Review the differential for recirculation - Review the recirculation fraction | 15 min | 16–30 | Handouts, slides, whiteboard | - It could be helpful to draw two circuits on a whiteboard or show pictures of recirculation - Suspect recirculation when SaO₂ decreases as ECMO flow increases, with paradoxically high SvO₂ and preserved post-oxygenator PaO₂. - Key numbers to follow: SaO₂, SvO₂ from drainage cannula, post-oxygenator PaO₂, ECMO flow trends, lactate. - Teaching point: High SvO₂ does not equal adequate oxygen delivery. |
| Case 3 application: Bleeding and preload limitation. | 10 min | 31–39 | Handouts, slides | - Teaching point: anemia and hypovolemia worsen hypoxemia despite adequate circuit function. |
| Wrap-up and post-session review. | 5 min | 40–41 | Post-survey, Slides | Key take-home points:   - Focus on oxygen delivery, not saturation alone. - Review the diagram of differential diagnosis of hypoxemia on VV-ECMO |

**Introduction: 5 min/Slides 1-3**

**Introduce the session and its objectives. The first 5 minutes can also be used to complete the pre-survey:**

***This session follows the POGIL format, or Process Oriented Guided Inquiry Learning – which is when learners work in small groups to build their own understanding of concepts through guided inquiries. You will be divided into groups of 2-5 people. The session consists of three cases. In the first case, we will introduce a framework or approach to the problem, which is Hypoxemia on VV-ECMO. In the next two cases, you will apply this approach to assess and manage related issues. I will guide you through the process.***

**Objectives**

1. Generate a differential diagnosis for hypoxemia in patients on VV-ECMO
2. Identify clinical signs and parameters indicative of oxygenator failure and decreased preload as an etiology of hypoxemia on VV-ECMO.
3. Identify clinical signs and parameters indicative of recirculation on VV-ECMO and generate a differential diagnosis for recirculation.

**Case 1: 25 min/ Slides 4-15**

Rebecca Nelson is a 14-year-old 50 kg female who was intubated for acute hypoxemic respiratory failure secondary to trauma from an automobile vs pedestrian accident with blunt force trauma to the chest. She required placement of bilateral chest tubes secondary to air leak and pulmonary contusions. She eventually developed ARDS with worsening hypoxemia and acidosis; it was determined that she necessitated elective cannulation to VV-ECMO.

- What are the options for VV-ECMO cannulation? And what would be the best strategy for this patient? **(5 min/Slides 6-7)**

| **Cannulation strategy** | **Location** | **Benefits** | **Risks** |
| --- | --- | --- | --- |
| Cervical with a single dual lumen cannula (Bicaval) | - Typically, Right IJ - Inflow ports: in the IVC and SVC. (Or only in the SVC, not available at our institution) - Outflow port: Right Atrium directed towards the tricuspid valve. | - One cannulation site makes patient mobilization easier - Vessel damage, infections or bleeding at the site limited to one site | - Size limitations: can affect flow rates and gas exchange efficiency - Recirculation - Need to be placed under fluoroscopy/echo guidance due to the risk of RA injury or perforation. - Cannula malposition |
| Multisite: Cervical-Femoral (Typically) | - Femoral cannula extending to the IVC - Right IJ re-infusion cannula in SVC | - Higher flow rates | - Highest rate of recirculation - Requires more time - Patient discomfort/limited mobilization - Greater risk of bleeding, infection |

She was cannulated to VV-ECMO with a single 27 French dual lumen cannula 72 hours ago. There was significant bleeding from chest tubes initially, but it has decreased, and no evidence of ongoing air leak. Below are the pertinent numbers shortly after cannulation:

| **Vitals** | T: 36.5C; HR: 96 bpm; BP: 105/50 mmHg; RR: 20 breaths/min; O_2_Sat: 89%; SvO_2_: 65%; CVP: 8 mmHg |
| --- | --- |
| **Infusions** | Fentanyl 2mcg/kg/hour; Midazolam 0.1mg/kg/hr, Heparin 22units/kg/hr |
| **ECMO Circuit** | Flows: 50 mL/kg/min, Pin: -51 mmHg, Pout: 155 mmHg, FiO_2_: 1.0, Sweep: 4.5L/min |
| **Ventilator Settings** | PRVC Mode: TV:8mL/kg, PIP 38 cmH_2_O, PEEP 14 cmH_2_O, Rate 20, FiO_2_ 0.75 |
| **Pertinent Labs** | WBC 7x10^3^/mL; Hb 8g/dL, Plt 94x10^3^/mL; Electrolytes: within normal limits, Anti-Xa 0.5 IU/mL; INR 2; aPTT: 65; Patient ABG: 7.23/65/45/20; Lactate 3.2 mmol/L; Post-oxygenator ABG: 7.3/53/350/18 |

- What should be your next step? And why? **(3 min/ Slide 8)**

Adjust the ventilator settings to the rest settings to minimize the risk of ventilator-induced lung injury and to optimize gas exchange. There is no consensus on what the ideal rest or baseline settings of a patient on VV-ECMO should be, but literature talks about using a similar strategy to a patient with ARDS:

1. Ventilator mode: Pressure control *to limit the variability of peak pressures that could occur with volume control ventilation and enable accurate documentation of peak pressures.*
2. PEEP: Moderate to high PEEP strategy to recruit the airways and promote clearance. Start at 10–15 cmH₂O and titrate based on the clinical picture, taking care to avoid overdistention and prevent airway collapse.
3. RR: 5-15 breaths/min – Since VV ECMO is providing full ventilatory support, the patient’s ventilation can be reduced accordingly.
4. Goal tidal volume: Target 4-6 mL/kg to maintain driving pressure below or equal to 15 cmH2O, and the plateau pressure below 25 cmH2O
5. PIP: ≤ 25 cmH2O
6. FiO2: < 0.5, to mitigate oxygen toxicity and decrease mortality

The underlying pathology must be considered in determining how best to support the underlying lung pathology. Please refer to the sheet for additional information.

The ventilator settings are adjusted. Over the next two days, some bleeding has been noted at the cannulation site, central venous line site, and chest tube. Fibrin stranding and a clot were noticed in the ECMO circuit at the connectors. The patient requires more frequent transfusions of blood products and platelets. The bedside nurses are mainly concerned of her oxygen saturation that has been down trending the last six hours and is now 76%. Her SvO_2_ has also dropped to 55%. Her Hb is now 8.5 g/dL.

- What is your differential diagnosis for hypoxemia in a patient on VV-ECMO? **(7min/ Slide 9-11)**

Overall, we can say that there are four key determinants that affect pulmonary arterial oxygen saturation: 1) ECLS flow, 2) recirculation, 3) native cardiac output, and 4) systemic venous saturation.

***We encourage you to take some time to review these slides and the next two figures, as they will help learners approach the upcoming cases and ensure they get the most out of them.***

The ECMO specialist has updated you, indicating that the patient's oxygen saturation continues to decrease, now in the low 70s range, despite increasing the ECMO flows. Additionally, she has observed an upward trend in the patient's lactate level, which is now at 5.3 mmol/L, and a decrease in the patient’s SvO_2_ to 43%.

- Is the patient’s oxygen delivery adequate? **(4 min/Slide 12 -*Spend a minute or so reviewing the oxygen delivery formula and how it applies to a patient on ECMO: on VV-ECMO, unlike VA-ECMO, the ECMO flow is not equal to the patient’s cardiac output; rather, cardiac output equals native venous return + VV-ECMO flow)***

DO2 = CO x CaO2

Hemoglobin

O2 Saturation

CO= VV ECMO Flow

+ Native venous return

A decrease of the SvO2 and an increase lactate despite increasing the ECMO flows suggests inadequate oxygen delivery.

- What would be the next step in attempting to determine the etiology of the patient's hypoxemia? **(3 min/Slides 12-13)**

Ask for a post-oxygenator PaO2 measurement and check for clots in the oxygenator. You could also assess the transmembrane pressure, which is still done in some institutions. These steps are usually done automatically by an ECMO specialist or the bedside perfusionist.

You are informed that the ECMO flows have begun to decline. Additionally, you are told that the transmembrane gradient is elevated, and the post-oxygenator gas is 7.29/68/85/21.

- What is the most likely cause of the patient’s hypoxemia in this case? **(1 min/ Slide 14)**

Her hypoxemia is likely due to a circuit problem, specifically oxygenator failure. Notable signs indicating impending oxygenator failure in her case include an increase in transmembrane pressure, worsening oxygenation or ventilation observed on post-oxygenator blood gases, abnormalities in coagulation studies, and the patient's coagulopathy possibly due to clot burden in the oxygenator, necessitating multiple transfusions.

- What other signs would you expect to see in the ECMO circuit if the cause of hypoxemia was oxygenator failure?

Another indicator would be the need for increased sweep flow or ECMO circuit/Ventilator FiO2 to maintain blood gases.

- What should be your next step, and how should you plan for it? **(2 min/ Slide 15)**

Change the whole circuit, which would require more time vs. changing only the oxygenator.

1. You should inform all the teams involved (ECMO, ICU, Cath lab)
2. Think of a different ventilation strategy during that time off
3. Optimize your sedation
4. You have to move infusions from the circuit to the patient’s side – especially vasoactive (+/- sedation) as the patient will be off infusions for ~ 1min
5. Add inotrope and have emergency medication to prepare for acute right heart failure if lung disease is severe enough

**Case 2: 15 min/ Slides 16-30**

**2 min/ Slides 17-19- *Allow learners a couple of minutes to review the values of both sets of vitals and highlight any that raise concern.***

Virginia Baker is a 2-year-old, 12 kg female, admitted with respiratory failure secondary to Rhinovirus/Enterovirus, with secondary Streptococcus Pneumoniae ARDS. She failed conventional and HFOV mechanical ventilation and was placed on VV ECMO with a 16 F dual lumen cannula 1 hour ago. She has been transitioned from the oscillator to rest ventilator settings. Patient was stable in the cath lab with oxygen saturations of 89%. On arrival to the PICU:

| **Vitals** | T: 36.5C; HR: 135 bpm; BP: 95/52 mmHg; RR: 10 breaths/min; O_2_Sat: 86%; SvO_2_: 77%, CVP 11 mmHg |
| --- | --- |
| **Infusions/**  **Medications** | Fentanyl 2mcg/kg/hour; Midazolam 0.1mg/kg/hour, Heparin 25units/kg/hr |
| **ECMO Circuit** | Flows: 80 mL/kg/min, Pin: -30 mmHg, Pout: 145mmHg, FiO_2_: 1.0, Sweep 3.5 L/min |
| **Ventilator Settings** | SIMC-PC Mode: PIP 20 cmH_2_O, PEEP 10 cmH_2_O, Rate 10, FiO_2_ 0.4 |
| **Physical Exam** | Intubated, awake, pupils equal and sluggish, Diffuse crackles bilaterally, Abdomen soft, cap refill 2 sec |
| **Pertinent Labs** | WBC 5x10^3^/mL; Hb 10g/dL, Plt 206x10^3^/mL; Electrolytes: within normal limits; Cr: 0.16 mg/dL; aPTT 72s, INT 1.3 |


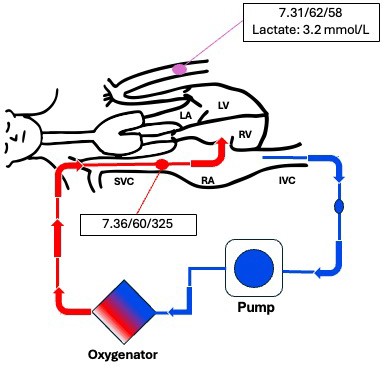


Author Owned Image

Shortly after arrival to the PICU, her Fentanyl drip was switched to Morphine drip, and she was started on a Cisatracurium drip due to agitation. You are called to the bedside because her saturation has dropped to 71% despite increasing the ECMO flow.

| **Vitals** | T: 37.7C; HR: 150 bpm; BP: 85/44 mmHg; RR: 10 breaths/min; O_2_Sat: 71%; SvO_2_: 90% |
| --- | --- |
| **Infusions/**  **Medications** | Morphine 0.1mg/kg/hour; Midazolam 0.1mg/kg/hour, Cisatracurium: 0.12 mg/kg/hour, Heparin 25units/kg/hour |
| **ECMO Circuit** | Flows: 100 mL/kg/min, Pin: -55mmHg, Pout: 145mmHg, FiO_2_: 1.0 |
| **Ventilator Settings** | SIMC-PC Mode: PIP 20 cmH_2_O, PEEP 10 cmH_2_O, Rate 10, FiO_2_ 0.4 |
| **Pertinent Labs** | Patient ABG:7.21/88/44/19; Lactate 4.1 mmol/L; Post-oxygenator ABG: 7.36/58/350/21 |

- Are the circuit flows and oxygenators the source of the patient’s hypoxemia? **(1 min/ Slide 20)**

No, the post-oxygenator gas is unchanged with a PaO2 of 350 mmHg, indicating a healthy oxygenator. The flows do not seem to have improved the O2 saturations. On the contrary, we noticed that with increasing flows from 80 to 100 mL/kg/min, we observed a decrease in the oxygen saturation from 86% to 71%, and an increase in mixed venous oxygen saturation from 77% to 90%. The patient also became more tachycardic and hypotensive.

- Is the patient’s oxygen delivery adequate? **(1 min/ Slide 21)**

Despite the high SvO2, the patient’s oxygen saturation is lower, and her blood pressure is also down-trending, likely due to decreased preload from recirculation. The low BP might lead to inadequate systemic oxygen delivery through inadequate perfusion pressure, which is likely leading to anaerobic metabolism, as indicated by a rising lactate level. Therefore, the oxygen delivery is suboptimal in this case. The high SvO2 is misleading and suggests that a significant portion of oxygenated blood is being recirculated rather than delivering oxygen to tissues.

Below are your new blood gases:

Author Owned Image

- What do you suspect as a possible etiology of your patient’s hypoxemia? **(7 min/ Slide 22-27- *Take some time to review recirculation, including its definition, the recirculation fraction, and the differential diagnosis, as it is an important complication of VV-ECMO.)***

The most likely cause of the patient’s hypoxemia is recirculation.

The high PaO2 and SvO2 values from the drainage cannula indicate that a significant amount of oxygenated blood is being recirculated back into the ECMO circuit without adequately perfusing the systemic circulation. Normally, this blood should have lower oxygen content, reflecting tissue oxygen extraction. The lower PaO2 and SaO2 in the patient’s arterial blood gas compared to the return cannula indicate that the systemic oxygen delivery is likely inadequate (depends on the demand). This reflects poor oxygenation at the tissue level, despite the high SvO2 in the drainage cannula. The oxygenated blood is inefficiently delivered to the patient's systemic circulation, leading to poor tissue oxygenation and anaerobic metabolism, as indicated by the rising lactate levels. The post-oxygenator blood gas shows expected values with a high PaO2 and SaO2, indicating that the oxygenator is functioning well and adequately oxygenating the blood.

- What is your next step? **(1 min/ Slide 28- *It would be helpful to display the figure illustrating the differential diagnosis of recirculation while answering this question, so learners can visualize the rationale behind the recommended steps.)***
- Decrease ECMO flows to see if recirculation decreases and systemic oxygen delivery improves
- Obtain a CXR and an echo - reposition cannula if needed
- Administer volume
- Right heart dysfunction can decrease forward flow through RV and increase recirculation
- can consider RV support with inotropy and pulmonary vasodilation
- Decrease metabolic demand - Increase sedation and consider neuromuscular blockade if not already done
- Support the patient: increase ventilator settings, FiO2 to ventilator/ circuit - in general these patients have sick lungs so cannot rely on this; want to prioritize optimizing ECMO flow and O2 delivery from ECMO

The ECMO specialist notes that there is no color differential between the two cannulas. The patient’s CXR is significant for bilateral lung consolidation, with the left lung more affected than the right. The endotracheal tube and ECMO cannula are in good position. Her echocardiography showed normal left ventricular function with an ejection fraction of 62%. The cannula position shows the tip in the IVC, and the return jet is turbulent and directed toward the atrial wall.

- Why are the turbulence of the jet and its location near the cannula tip a problem? **(2 min/ Slide 29 - *It would be helpful to pull up either the slides or an image of a bicaval cannula to show learners how the cannula sits within the atrium and where it should be positioned.)***

The return turbulent flow and its proximity to the cannula tip can exacerbate recirculation by creating a zone of low-pressure or stagnant flow around the cannula, where recirculation is more likely to occur. Addressing turbulent flow and optimizing cannula positioning are essential not only to mitigate recirculation and maximize the efficacy of ECMO but also to minimize complications such as hemolysis, thrombosis, inefficient oxygenation, and cannula wear and damage.

Ideally, the cannula should be positioned with its tip in the IVC and outflow port directed at the tricuspid valve

- What is your plan of action? **(1 min/ Slide 30)**
  - Consult the Cath team for cannula repositioning.
  - Plan for additional sedation and volume

It is unlikely in this case, given the normal cardiac function described above, but keep in mind that some patients might need additional inotropic support. Conversion to VA-ECMO might be needed if the patient requires more flow than can be delivered without significant recirculation, persistent desaturation, poor cardiac function refractory to vasopressors, or increasing lactate and metabolic acidosis.

**Case 3: 10 min/ Slides 31-39**

**2 min/ Slides 32-33: *Allow learners a couple of minutes to review the values of both sets of vitals and highlight any that raise concern.***

Rosa Franklin is a 3-year-old, 15 kg female with subacute autoimmune hepatitis, admitted with acute respiratory failure secondary to influenza A pneumonia. She was cannulated to VV ECMO with a 19 French dual lumen cannula for persistent hypoxemia. Her cannulation was notable for initial bleeding at the cannulation site that resolved with plasma and platelet infusions in the first 24 hours.

This is day 2 of VV ECMO (hour 36). Overnight, she was difficult to sedate but now seems to have adequate analgesia and sedation on morphine and Precedex infusions, respectively. Her transaminases, coagulation studies, and ammonia are elevated. The ECMO specialist notifies you that she is starting to ‘ooze significantly’ from a variety of sites.

| **Vitals** | T: 36.5C; HR: 150 bpm; BP: 85/32 mmHg; RR: 10 breaths/min; O_2_Sat: 85%; SvO_2_: 45%, CVP 10 mmHg |
| --- | --- |
| **Infusions/**  **Medications** | Morphine 1mg/kg/hour; Dexmedetomidine 0.5 mcg/kg/hour, Bivalirudin 0.35mg/kg/hour |
| **ECMO Circuit** | Flows: 80 mL/kg/min, Pin: -30 mmHg, Pout: 175mmHg, FiO_2_: 1.0, Sweep 2L/min |
| **Ventilator Settings** | SIMC-PC Mode: PIP 20 cmH_2_O, PEEP 10 cmH_2_O, Rate 10, FiO_2_ 0.3 |
| **Physical Exam** | Intubated, sedated, thin, pupils equal and sluggish, sinus tachycardia, fine coarse breath sounds bilaterally, Abdomen distended, Liver 6 cm below RCM and firm, cap refill 2 sec, oozing from CVC, art line and cannula sites |
| **Pertinent Labs** | WBC 12x10^3^/mL; Hb 7.5 g/dL, Plt 116x10^3^/mL; Electrolytes: within normal limits; Cr: 0.14 mg/dL; AST 2600 U/L; ALT 2855 U/L; Alb 2.8 g/dL; total Bili 4 mg/dL; aPTT 120s; INR 2.4; Pump ABG: 7.45/35/483/24; Patient ABG: 7.35/45/40/24, Lactate: 4.1 |

- Is the patient’s oxygen delivery adequate? **(1 min/ Slide 34)**

Given the hypotension, tachycardia, low SaO2, low SvO2, (with an AVDO2 40%), elevated lactate, and anemia, the patient's oxygen delivery is inadequate. The body is showing signs of

compensatory mechanisms (tachycardia), poor perfusion (hypotension), and a shift to anaerobic metabolism (elevated lactate), all of which point to compromised oxygen delivery.

- How would you approach the patient’s hypoxemia? **(3 min/ Slide 35- *It may be helpful to refer learners to the diagram outlining the diagnostic workup of hypoxemia at the end of their handout, to ensure they understand each step and are able to answer the question.)***
- You could try increasing the ECMO flows to check if it increases your SaO2.
- Your post-oxygenator ABG demonstrates a PaO2 of 483 mmHg, suggestive of a healthy oxygenator.
- Obtain a CXR and/or echo to check your cannula position.
- The patient’s clinical picture is not suggestive of either low or high native cardiac output leading to hypoxemia.
- The patient is anemic, which could be causing low oxygen delivery and thus low SvO2 and overall hypoxemia.

The CXR shows diffuse bilateral infiltrates with a paucity of aeration. No pneumothorax, but she has now developed a right-sided large effusion. No change in cannula position. The ECMO specialist informs you that after increasing the ECMO flows to 100mL/kg/min, the patient’s SaO_2_ dropped to 75%, and the patient’s SvO_2_ increased to 75%.

- What does that intervention suggest? **(1 min/ Slide 36)**

Increasing the flows further lead to recirculation. You should ask to decrease the flow back to 80mL/kg/min to minimize recirculation.

The patient's nurse is concerned that the dressing on her neck is soaked with blood. She also mentioned that the patient's numbers are changing rapidly and drastically, and they are having a harder time maintaining the ECMO flow:

| **Vitals** | T: 36.5C; HR: 160 bpm; BP: 65/34 mmHg; RR: 10 breaths/min; O_2_Sat: 79%; SvO_2_: 60%, CVP 4 mmHg |
| --- | --- |
| **ECMO Circuit** | Flows: 60 mL/kg/min, Pin: -60 mmHg, Pout: 165mmHg, FiO_2_: 1.0 |
| **Physical Exam** | Cooler extremities with a capillary refill of 6 seconds, few disrupted sutures are noted at the cannula site with ongoing bleeding, mottled throughout |

- What is the most likely etiology of the patient’s hypoxemia? What would be your next steps? **(2 min/ Slide 38)**

Inadequate preload, likely secondary to anemia from significant bleeding

1. Fluid resuscitation and Administer blood products in the meantime, and if the bleeding is excessive, consider activating the mass transfusion protocol. Your patient has ongoing liver dysfunction and is at high risk for ongoing bleeding. Administer volume rapidly through the circuit, patient lines or both.
2. Obtain an echocardiogram to evaluate your cannula’s position upon noticing loose sutures at the cannula site.
3. Repeat CBC, coagulation studies, ABG, and lactate.
4. Pause anticoagulation

Below are some critical lab results: hemoglobin 5, platelet count 60K, INR 3. Point of care arterial blood gas: 7.11/68/35/14, lactate 5.1, ionized Calcium 1.1.

- What would be your plan of action? **(1 min/ Slide 39)**
- MTP
- Increase ventilator settings/FiO2 and you could also bag mask ventilate the patient in the meantime.
- Initiate pressors if needed.
- Notify the surgeons and/or Cath lab of the significant bleeding at the cannulation site
- Discuss right sided effusion drainage – Less likely if the patient is still on ECMO as it can exacerbate the bleeding. Would most likely allow it to tamponade on its own
- Consider administering calcium if multiple blood products were given
- Inform the hepatology team

**Wrap up: 5 min/Slides 40-41:**

***Ask if there are any questions, and allow learners to spend the final five minutes completing the post-survey***
