# Supplementary material for: Enhancing Pediatric Extracorporeal Membrane Oxygenation Education Through Process-Oriented Guided Inquiry Learning Sessions for Fellows and Advanced Practice Providers
Source: MedEdPORTAL. 2026 May 12;22:11600. doi: 10.15766/mep_2374-8265.11600 (PMC13161199; doi:10.15766/mep_2374-8265.11600)
Supplement: Supplementary file 1 — VA-ECMO Learner Handout.docxVV-ECMO Learner Handout.docxVA-ECMO Facilitator Guide.docxVV-ECMO Facilitator Guide.docxVA-ECMO Slides.pptxVV-ECMO Slides.pptxVA-ECMO Presurvey.docxVV-ECMO Presurvey.docxVA-ECMO Postsurvey.docxVV-ECMO Postsurvey.docx [file mep_2374-8265.11600-s001.zip › E. VA-ECMO Slides.pptx]

## Slide 1
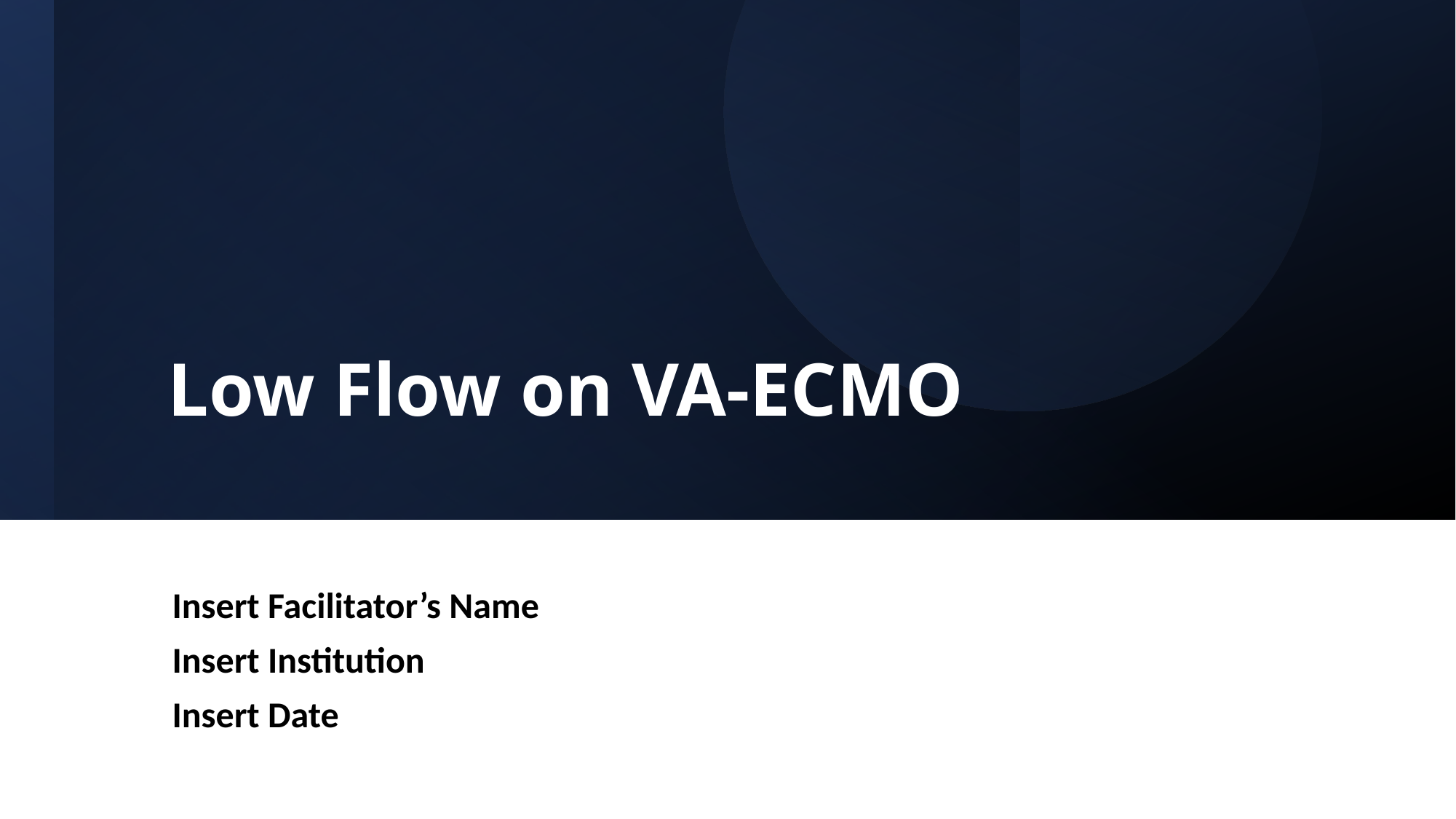

# Low Flow on VA-ECMO
Insert Facilitator’s Name
Insert Institution
Insert Date

## Slide 2
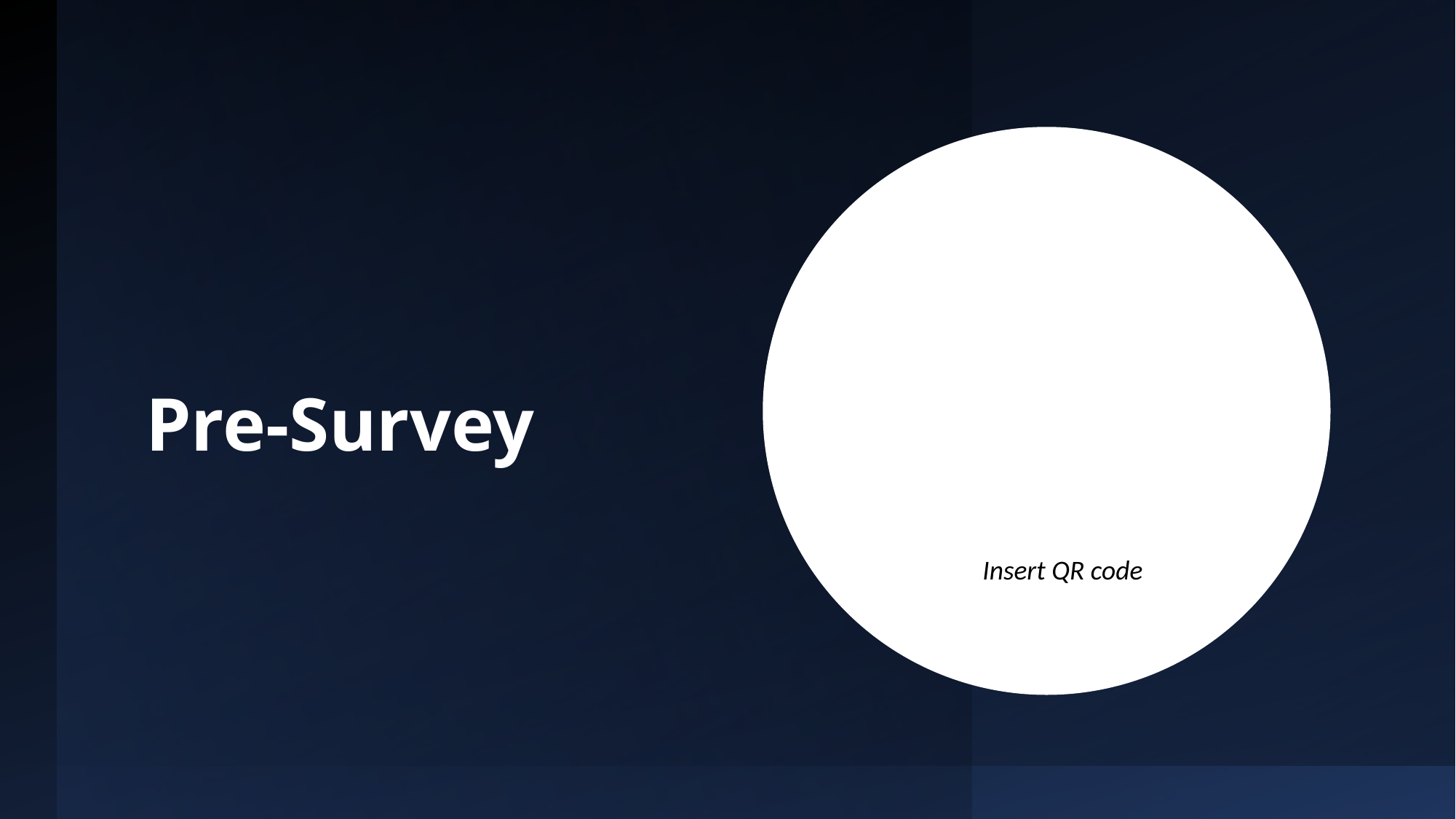

# Pre-Survey
Insert QR code

## Slide 3
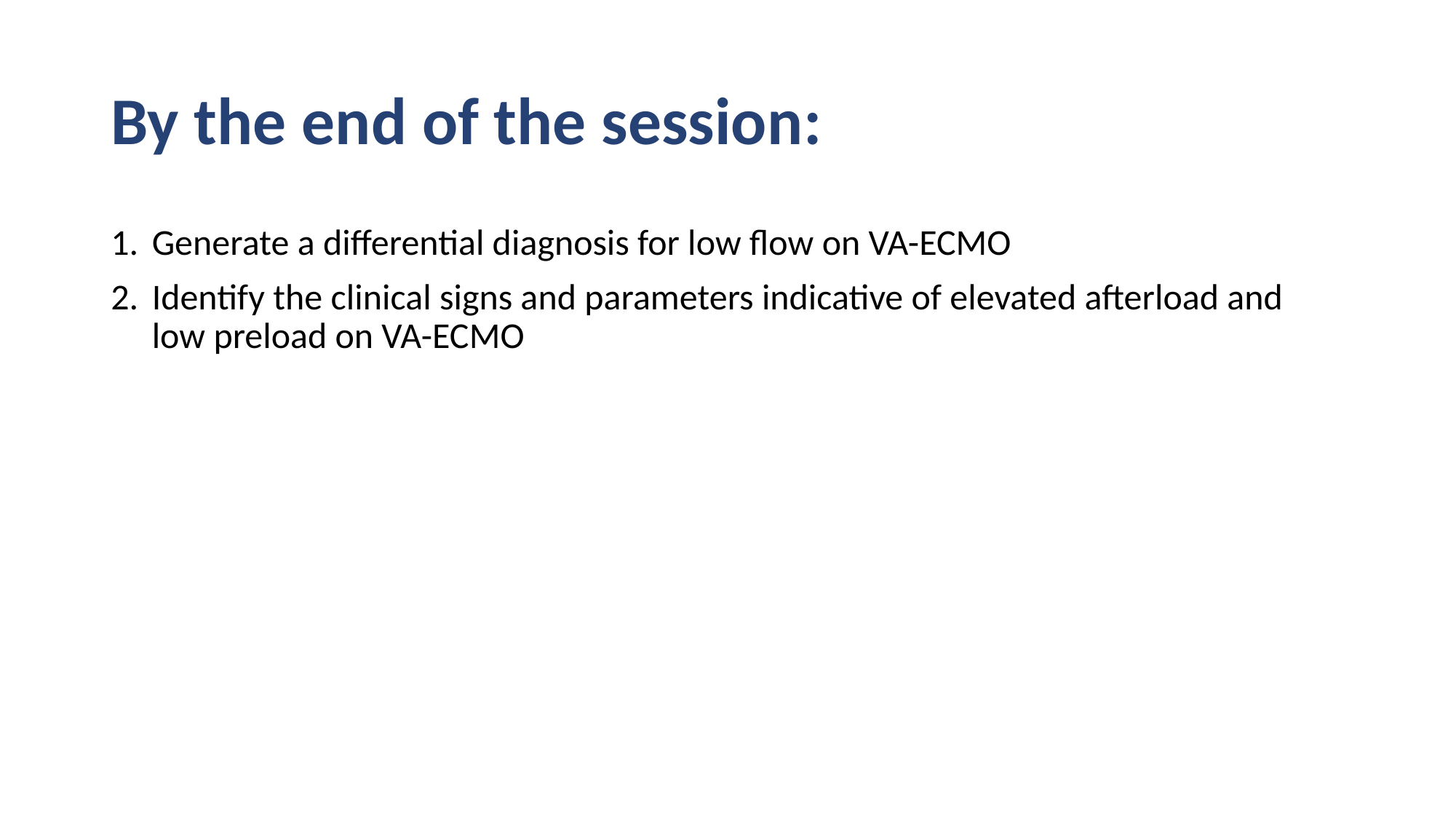

# By the end of the session:
Generate a differential diagnosis for low flow on VA-ECMO
Identify the clinical signs and parameters indicative of elevated afterload and low preload on VA-ECMO

## Slide 4
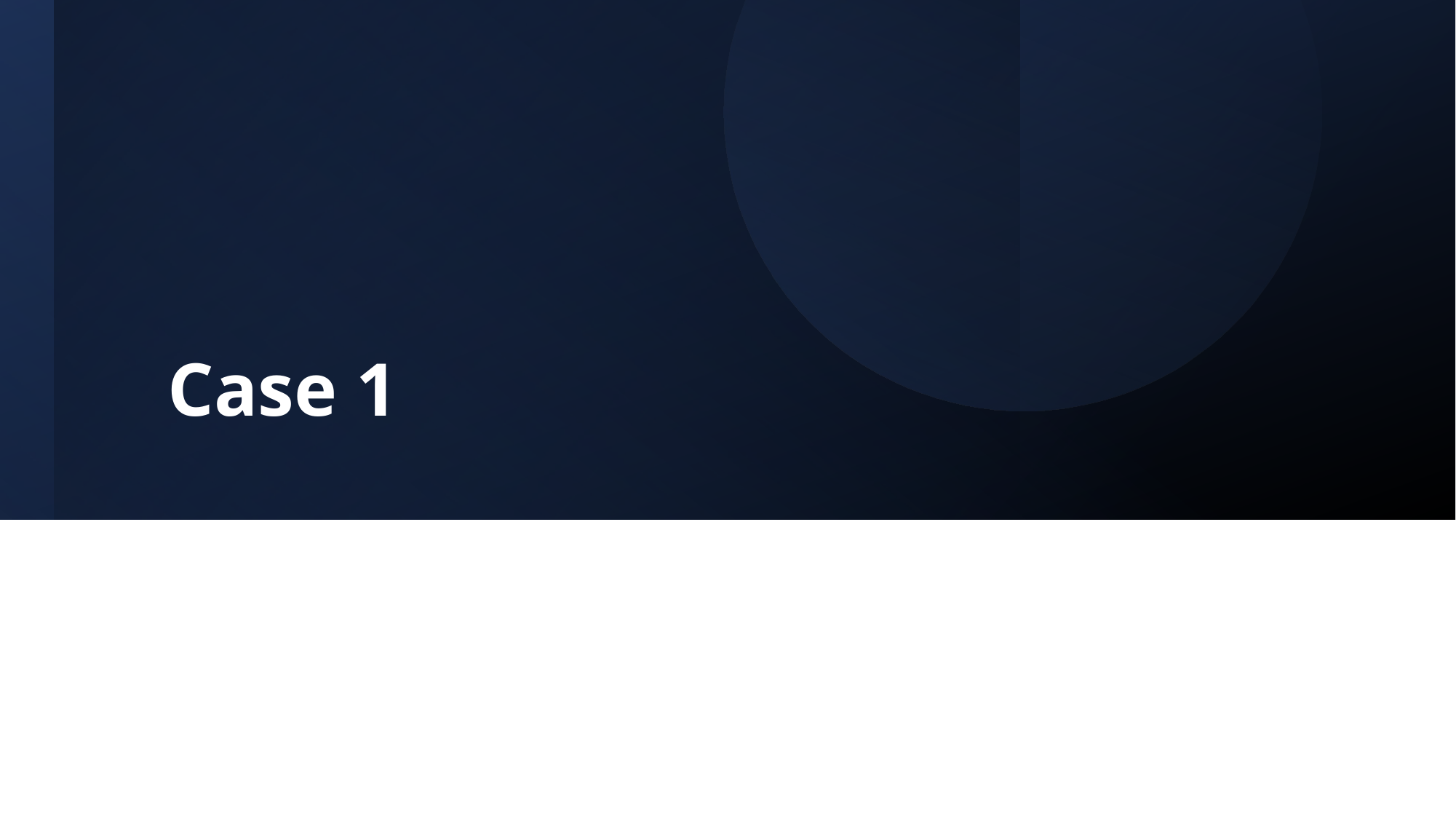

# Case 1

## Slide 5
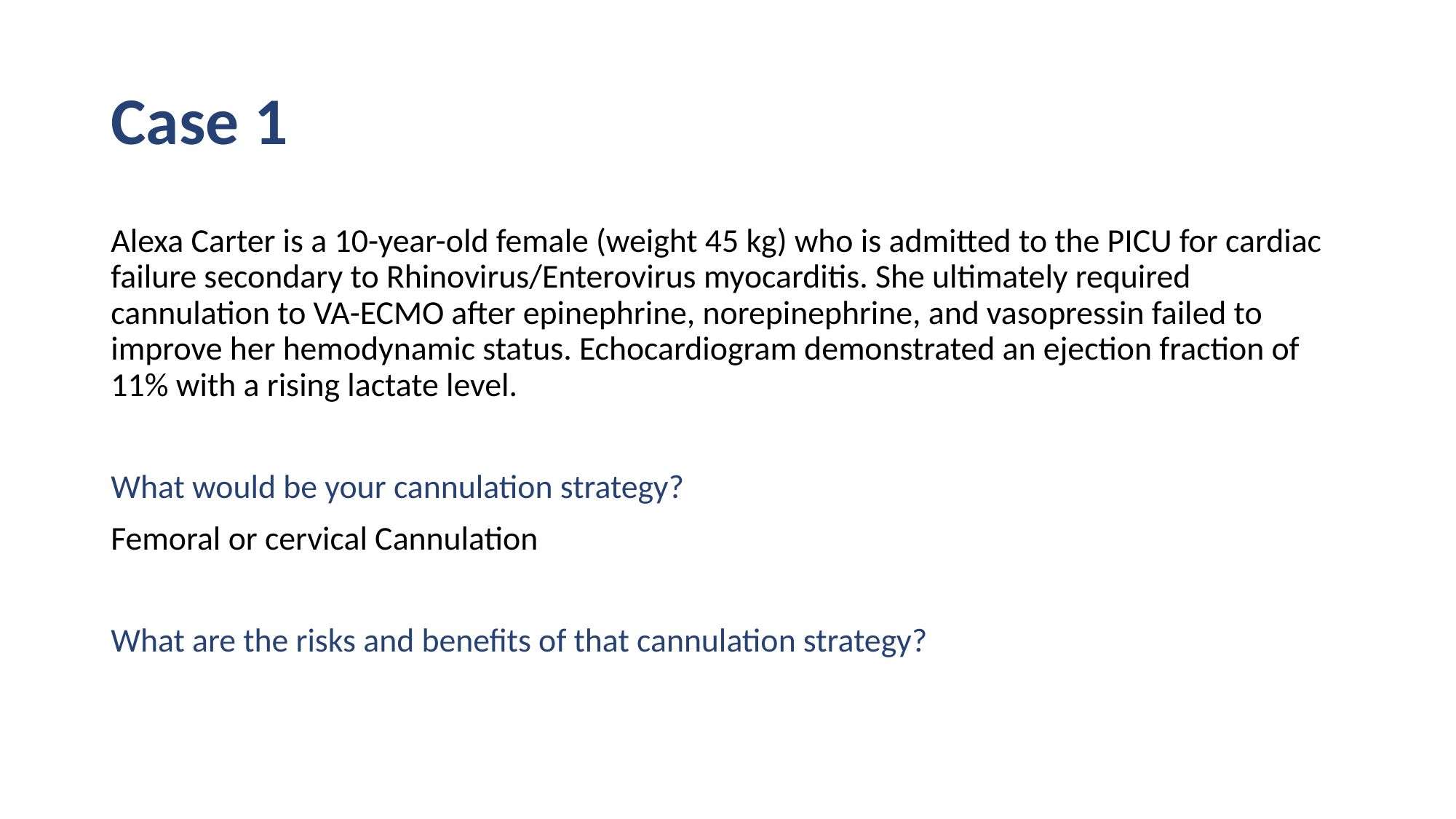

# Case 1
Alexa Carter is a 10-year-old female (weight 45 kg) who is admitted to the PICU for cardiac failure secondary to Rhinovirus/Enterovirus myocarditis. She ultimately required cannulation to VA-ECMO after epinephrine, norepinephrine, and vasopressin failed to improve her hemodynamic status. Echocardiogram demonstrated an ejection fraction of 11% with a rising lactate level.
What would be your cannulation strategy?
Femoral or cervical Cannulation
What are the risks and benefits of that cannulation strategy?

## Slide 6
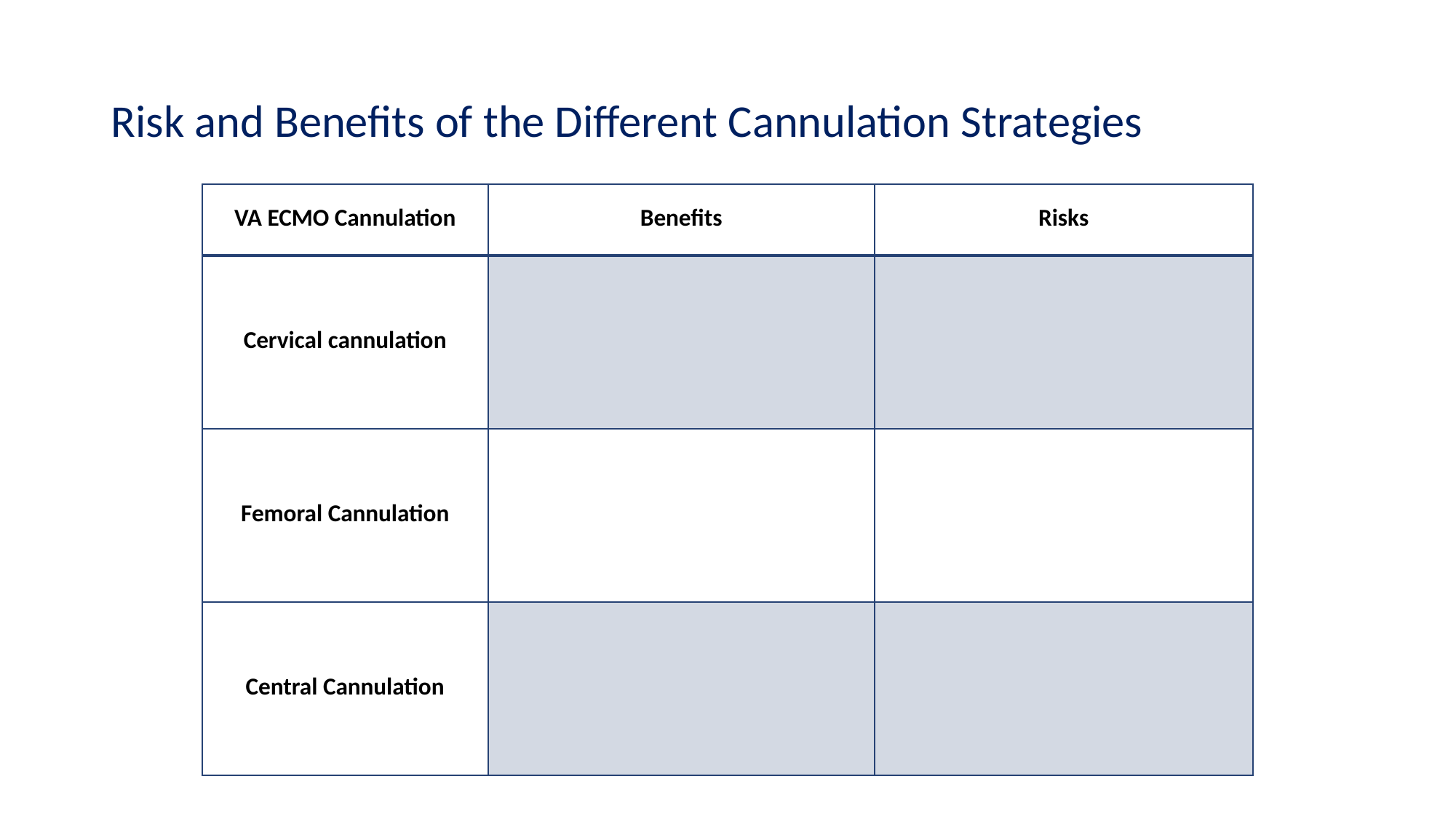

# Risk and Benefits of the Different Cannulation Strategies
| VA ECMO Cannulation | Benefits | Risks |
| --- | --- | --- |
| Cervical cannulation | | |
| Femoral Cannulation | | |
| Central Cannulation | | |

## Slide 7
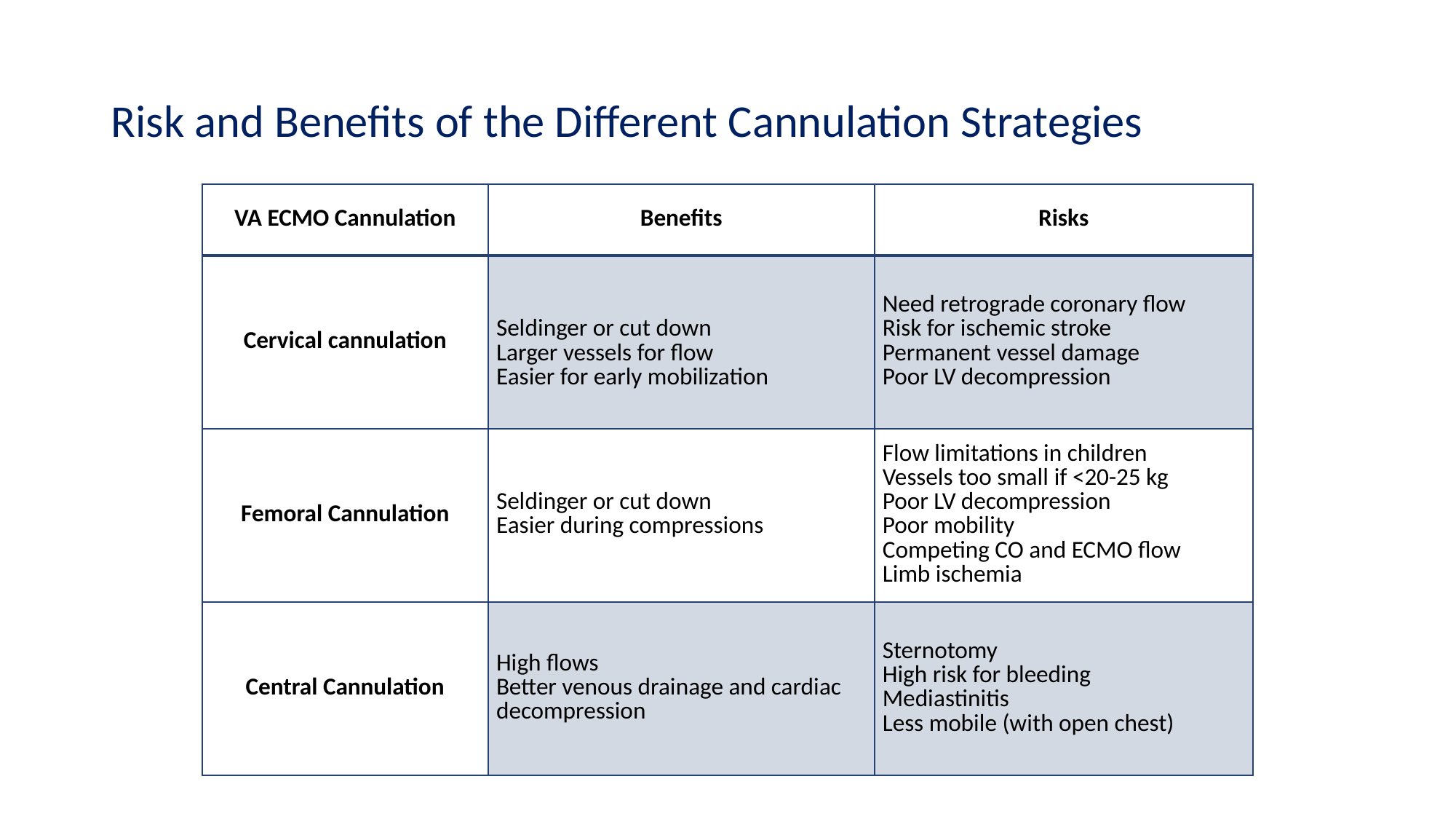

# Risk and Benefits of the Different Cannulation Strategies
| VA ECMO Cannulation | Benefits | Risks |
| --- | --- | --- |
| Cervical cannulation | Seldinger or cut down ​Larger vessels for flow Easier for early mobilization | Need retrograde coronary flow  ​ Risk for ischemic stroke Permanent vessel damage Poor LV decompression |
| Femoral Cannulation | Seldinger or cut down​ Easier during compressions | Flow limitations in children​ Vessels too small if <20-25 kg Poor LV decompression​ Poor mobility​ Competing CO and ECMO flow​ Limb ischemia |
| Central Cannulation | High flows​ Better venous drainage and cardiac decompression | Sternotomy​ High risk for bleeding​ Mediastinitis​ Less mobile (with open chest) |

## Slide 8
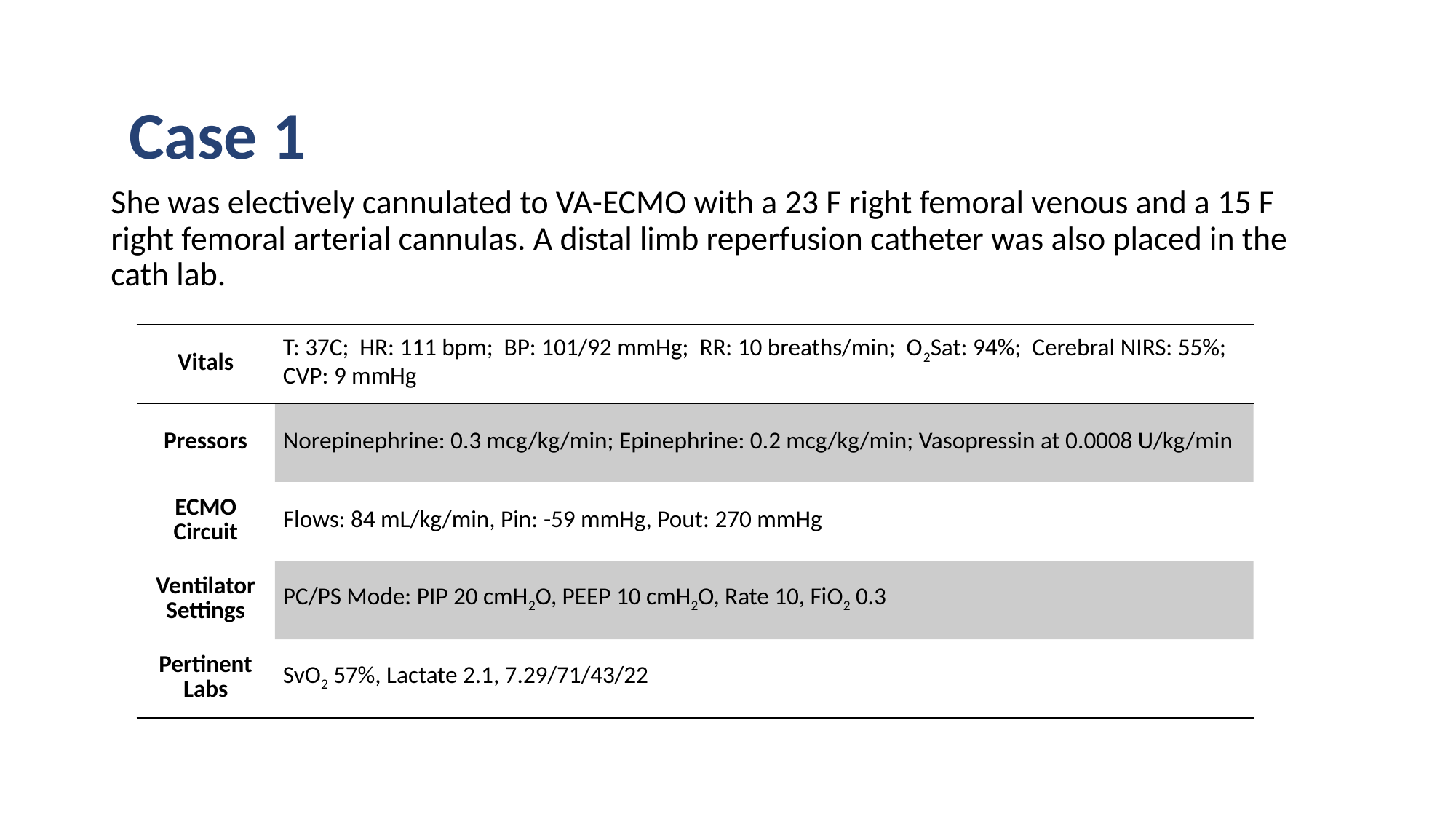

Case 1
She was electively cannulated to VA-ECMO with a 23 F right femoral venous and a 15 F right femoral arterial cannulas. A distal limb reperfusion catheter was also placed in the cath lab.
| Vitals | T: 37C; HR: 111 bpm; BP: 101/92 mmHg; RR: 10 breaths/min; O2Sat: 94%; Cerebral NIRS: 55%; CVP: 9 mmHg |
| --- | --- |
| Pressors | Norepinephrine: 0.3 mcg/kg/min; Epinephrine: 0.2 mcg/kg/min; Vasopressin at 0.0008 U/kg/min |
| ECMO Circuit | Flows: 84 mL/kg/min, Pin: -59 mmHg, Pout: 270 mmHg |
| Ventilator Settings | PC/PS Mode: PIP 20 cmH2O, PEEP 10 cmH2O, Rate 10, FiO2 0.3 |
| Pertinent Labs | SvO2 57%, Lactate 2.1, 7.29/71/43/22 |

## Slide 9
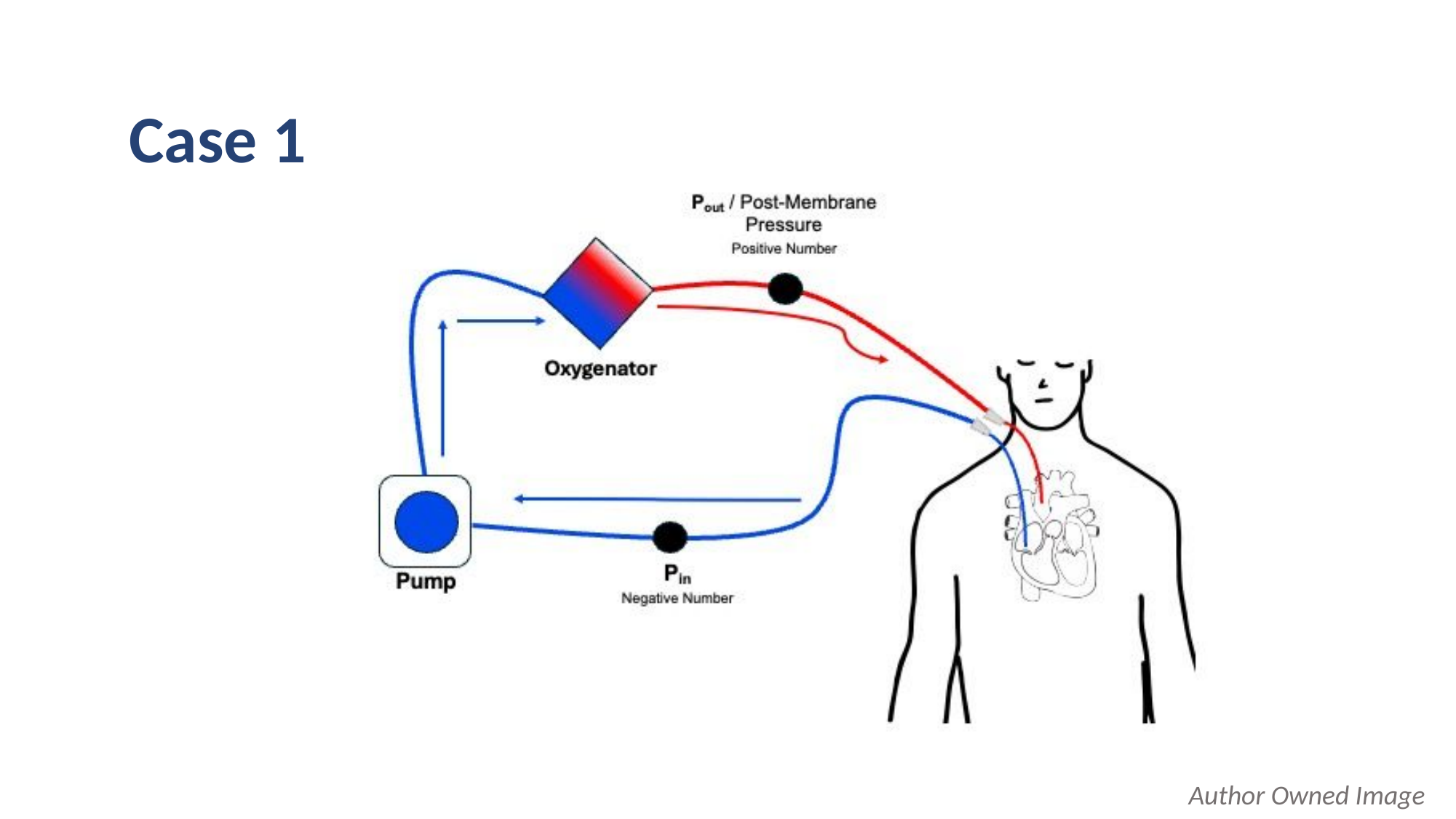

Case 1
Author Owned Image

## Slide 10
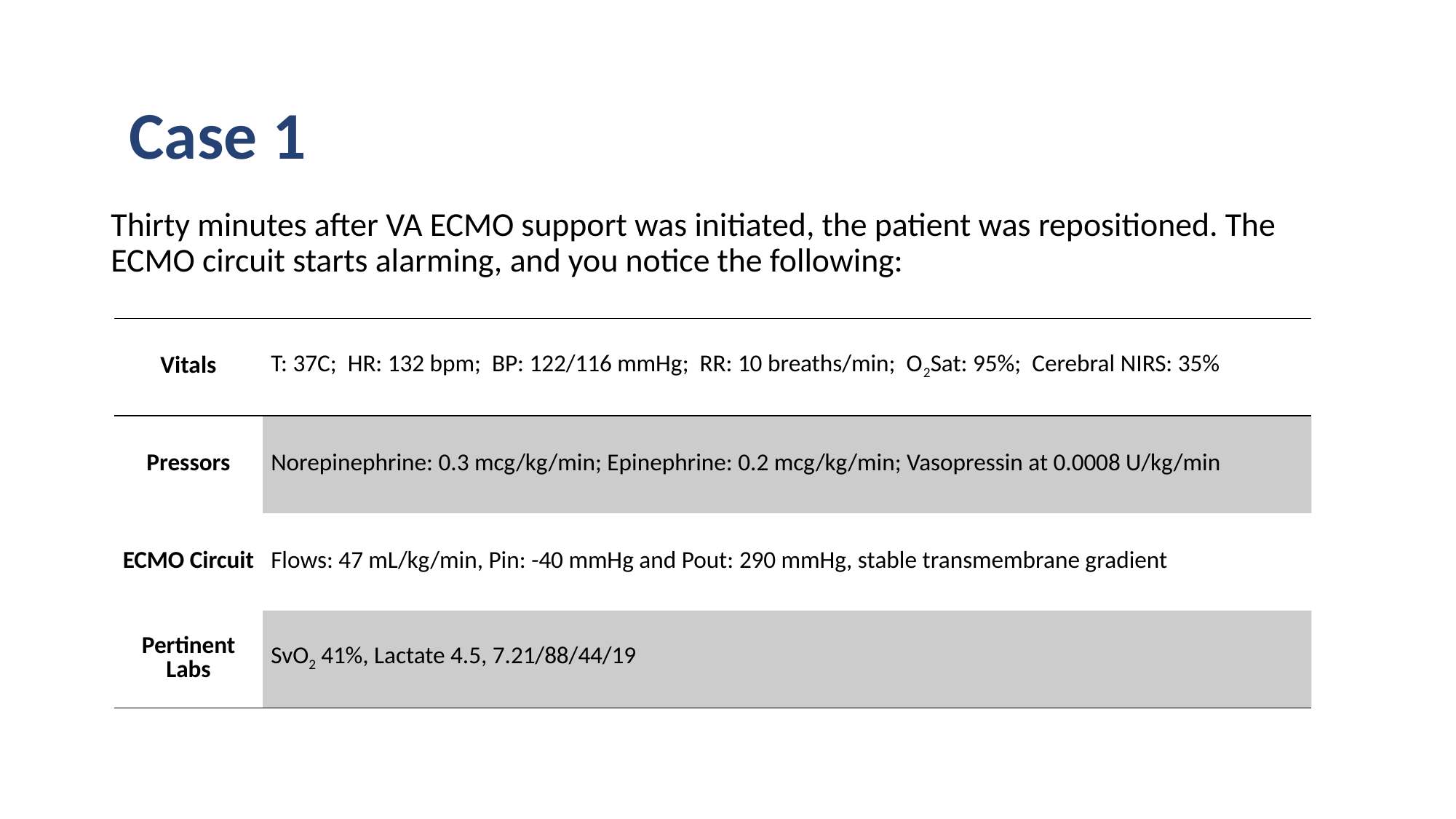

Case 1
Thirty minutes after VA ECMO support was initiated, the patient was repositioned. The ECMO circuit starts alarming, and you notice the following:
| Vitals | T: 37C; HR: 132 bpm; BP: 122/116 mmHg; RR: 10 breaths/min; O2Sat: 95%; Cerebral NIRS: 35% |
| --- | --- |
| Pressors | Norepinephrine: 0.3 mcg/kg/min; Epinephrine: 0.2 mcg/kg/min; Vasopressin at 0.0008 U/kg/min |
| ECMO Circuit | Flows: 47 mL/kg/min, Pin: -40 mmHg and Pout: 290 mmHg, stable transmembrane gradient |
| Pertinent Labs | SvO2 41%, Lactate 4.5, 7.21/88/44/19 |

## Slide 11
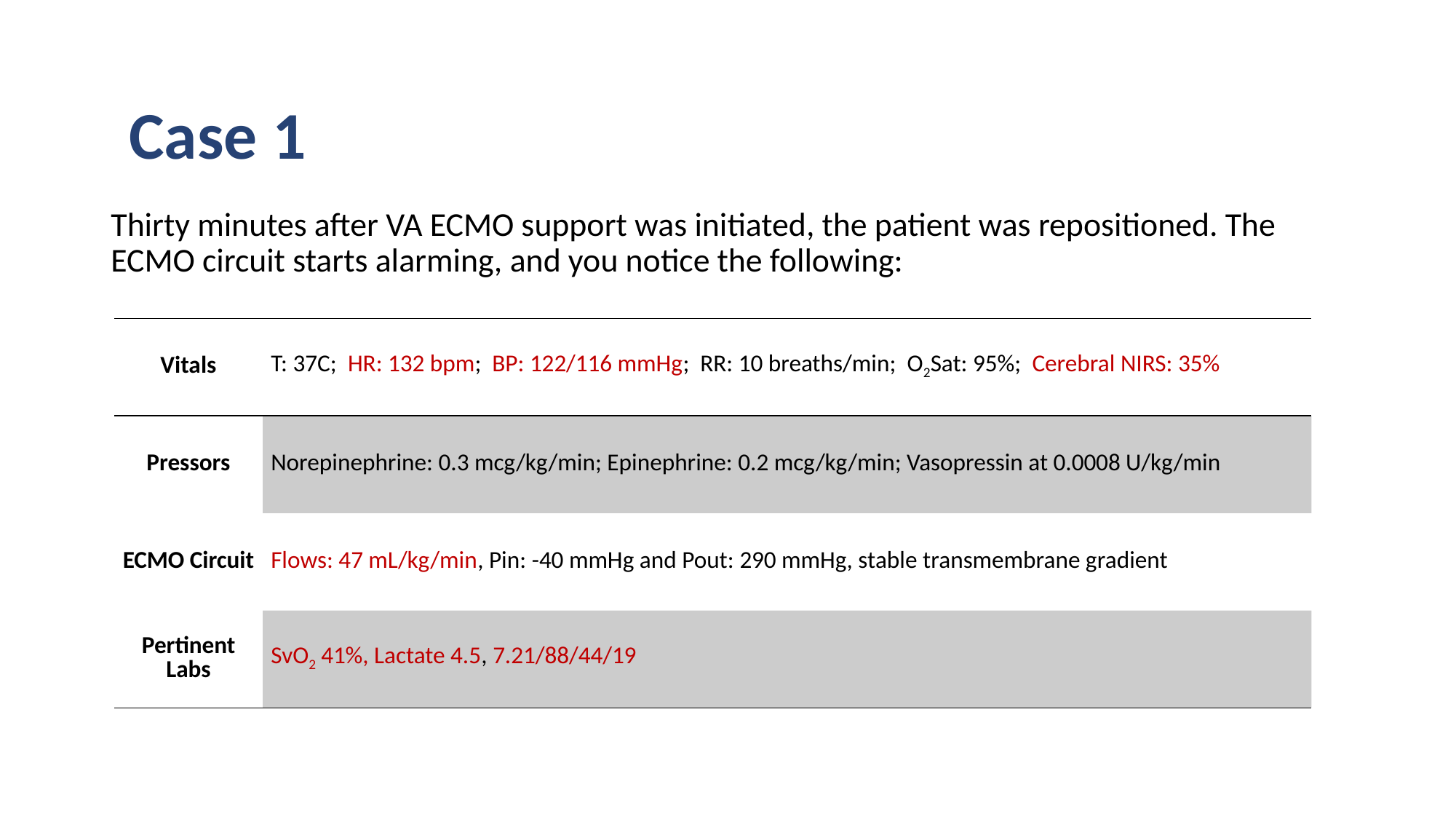

Case 1
Thirty minutes after VA ECMO support was initiated, the patient was repositioned. The ECMO circuit starts alarming, and you notice the following:
| Vitals | T: 37C; HR: 132 bpm; BP: 122/116 mmHg; RR: 10 breaths/min; O2Sat: 95%; Cerebral NIRS: 35% |
| --- | --- |
| Pressors | Norepinephrine: 0.3 mcg/kg/min; Epinephrine: 0.2 mcg/kg/min; Vasopressin at 0.0008 U/kg/min |
| ECMO Circuit | Flows: 47 mL/kg/min, Pin: -40 mmHg and Pout: 290 mmHg, stable transmembrane gradient |
| Pertinent Labs | SvO2 41%, Lactate 4.5, 7.21/88/44/19 |

## Slide 12
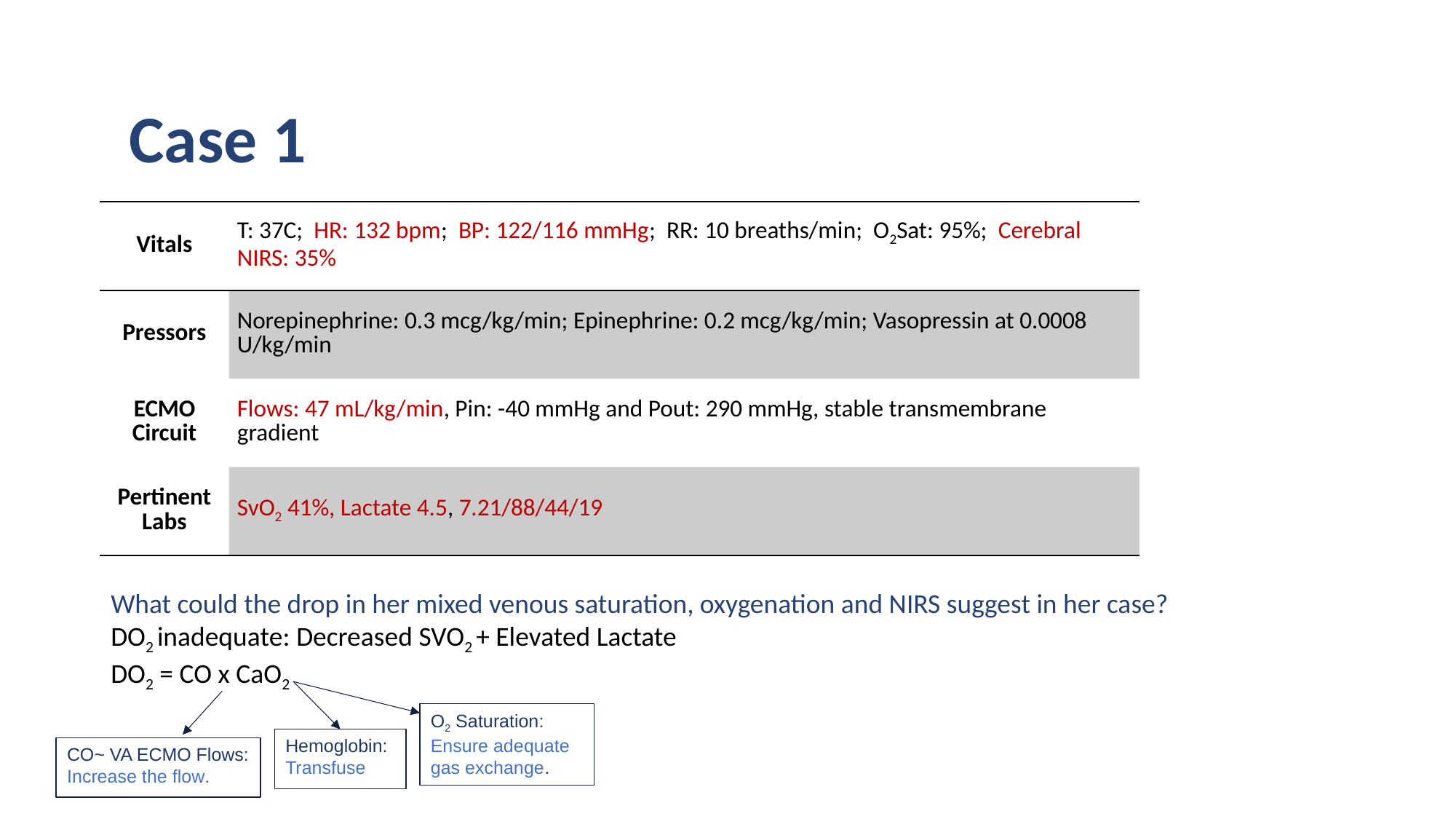

Case 1
| Vitals | T: 37C; HR: 132 bpm; BP: 122/116 mmHg; RR: 10 breaths/min; O2Sat: 95%; Cerebral NIRS: 35% |
| --- | --- |
| Pressors | Norepinephrine: 0.3 mcg/kg/min; Epinephrine: 0.2 mcg/kg/min; Vasopressin at 0.0008 U/kg/min |
| ECMO Circuit | Flows: 47 mL/kg/min, Pin: -40 mmHg and Pout: 290 mmHg, stable transmembrane gradient |
| Pertinent Labs | SvO2 41%, Lactate 4.5, 7.21/88/44/19 |
What could the drop in her mixed venous saturation, oxygenation and NIRS suggest in her case?
DO2 inadequate: Decreased SVO2 + Elevated Lactate
DO2 = CO x CaO2
O2 Saturation:
Ensure adequate gas exchange.
Hemoglobin:
Transfuse
CO~ VA ECMO Flows:
Increase the flow.

## Slide 13
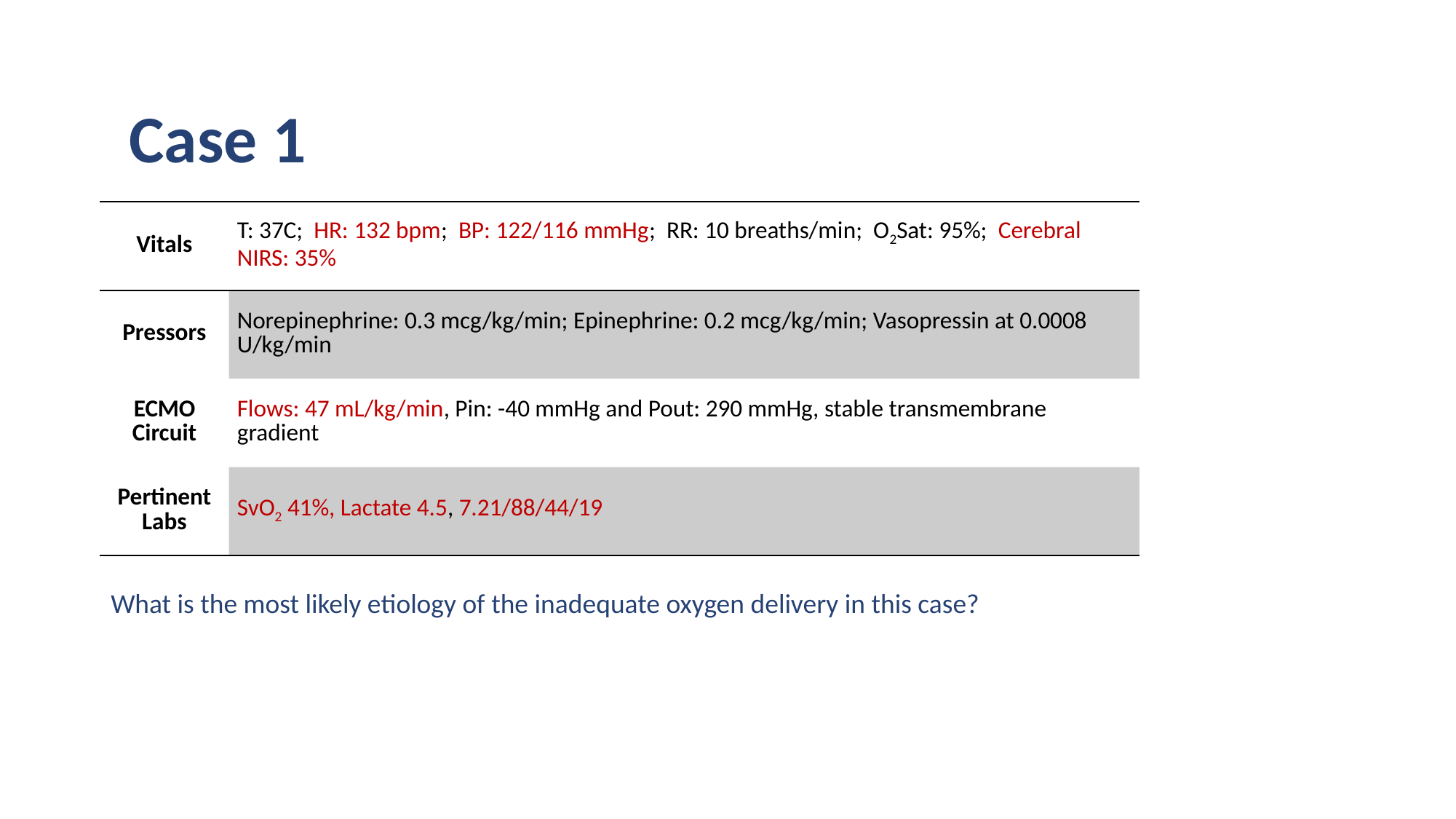

Case 1
| Vitals | T: 37C; HR: 132 bpm; BP: 122/116 mmHg; RR: 10 breaths/min; O2Sat: 95%; Cerebral NIRS: 35% |
| --- | --- |
| Pressors | Norepinephrine: 0.3 mcg/kg/min; Epinephrine: 0.2 mcg/kg/min; Vasopressin at 0.0008 U/kg/min |
| ECMO Circuit | Flows: 47 mL/kg/min, Pin: -40 mmHg and Pout: 290 mmHg, stable transmembrane gradient |
| Pertinent Labs | SvO2 41%, Lactate 4.5, 7.21/88/44/19 |
What is the most likely etiology of the inadequate oxygen delivery in this case?

## Slide 14
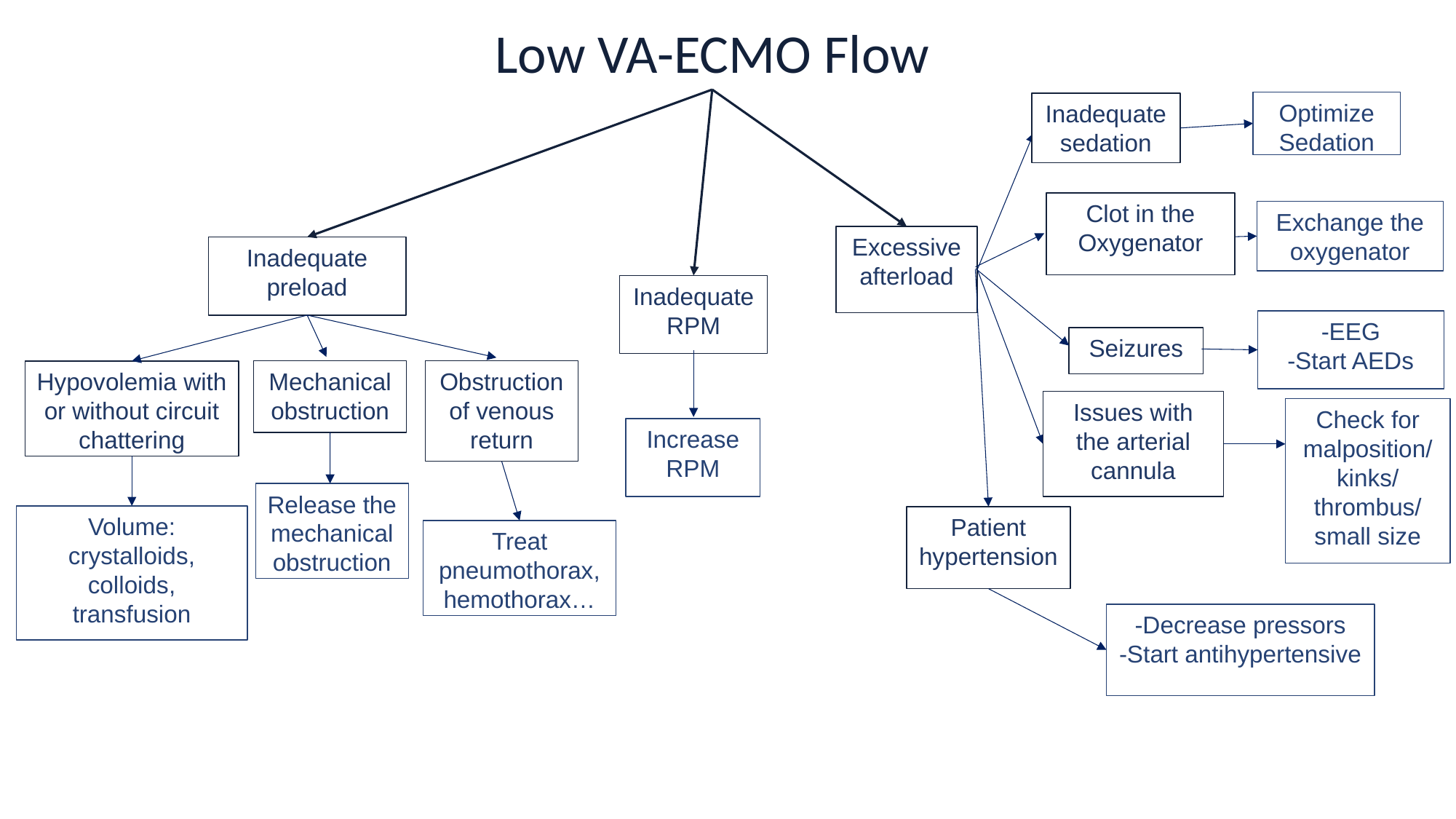

Low VA-ECMO Flow
Optimize Sedation
Inadequate sedation
Clot in the Oxygenator
Exchange the oxygenator
Excessive afterload
Inadequate preload
Inadequate RPM
-EEG
-Start AEDs
Seizures
Obstruction of venous return
Mechanical obstruction
Hypovolemia with or without circuit chattering
Issues with the arterial cannula
Check for malposition/ kinks/ thrombus/ small size
Increase RPM
Release the mechanical obstruction
Volume: crystalloids, colloids, transfusion
Patient hypertension
Treat pneumothorax, hemothorax…
-Decrease pressors
-Start antihypertensive

## Slide 15
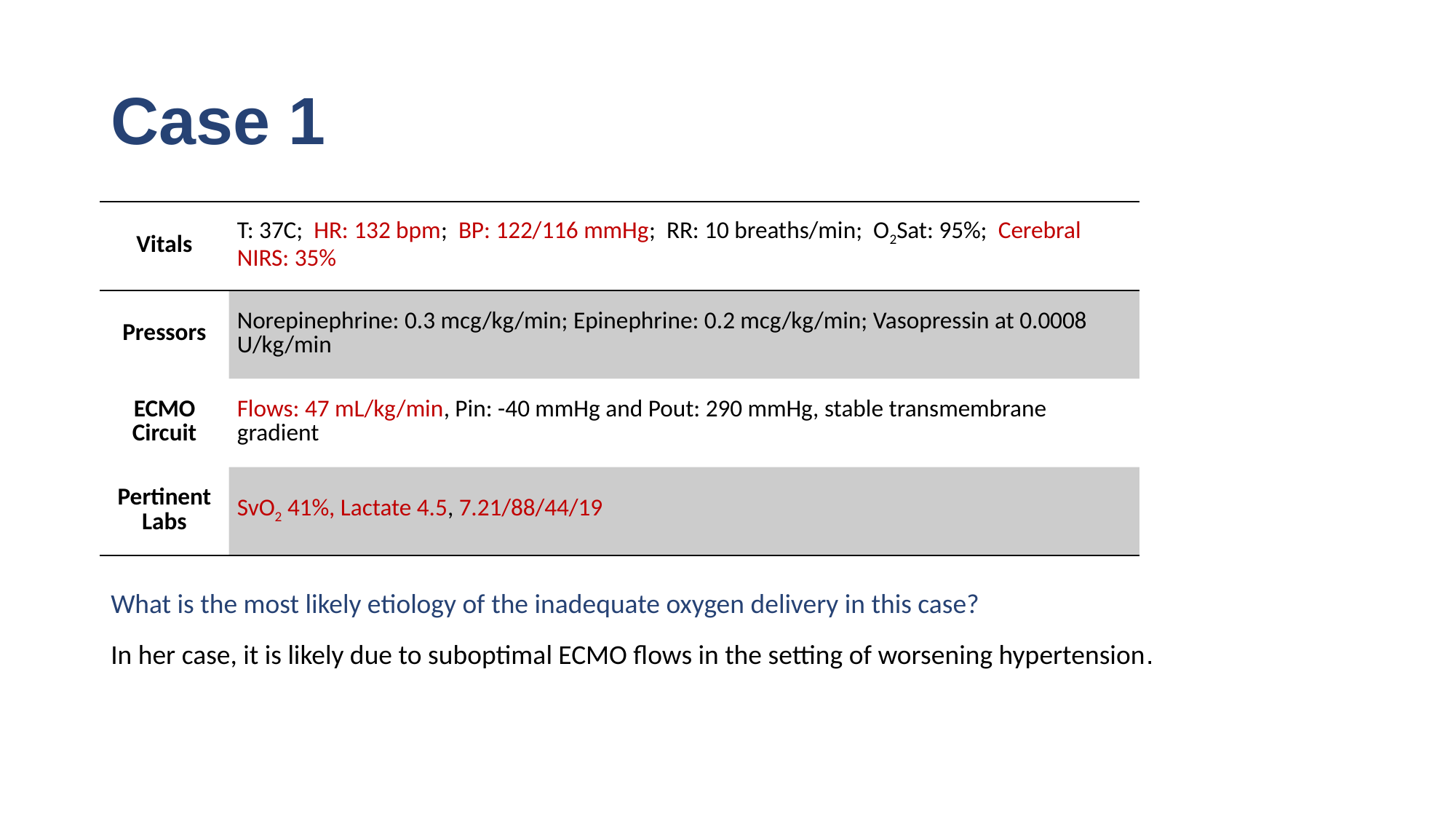

# Case 1
| Vitals | T: 37C; HR: 132 bpm; BP: 122/116 mmHg; RR: 10 breaths/min; O2Sat: 95%; Cerebral NIRS: 35% |
| --- | --- |
| Pressors | Norepinephrine: 0.3 mcg/kg/min; Epinephrine: 0.2 mcg/kg/min; Vasopressin at 0.0008 U/kg/min |
| ECMO Circuit | Flows: 47 mL/kg/min, Pin: -40 mmHg and Pout: 290 mmHg, stable transmembrane gradient |
| Pertinent Labs | SvO2 41%, Lactate 4.5, 7.21/88/44/19 |
What is the most likely etiology of the inadequate oxygen delivery in this case?
In her case, it is likely due to suboptimal ECMO flows in the setting of worsening hypertension.

## Slide 16
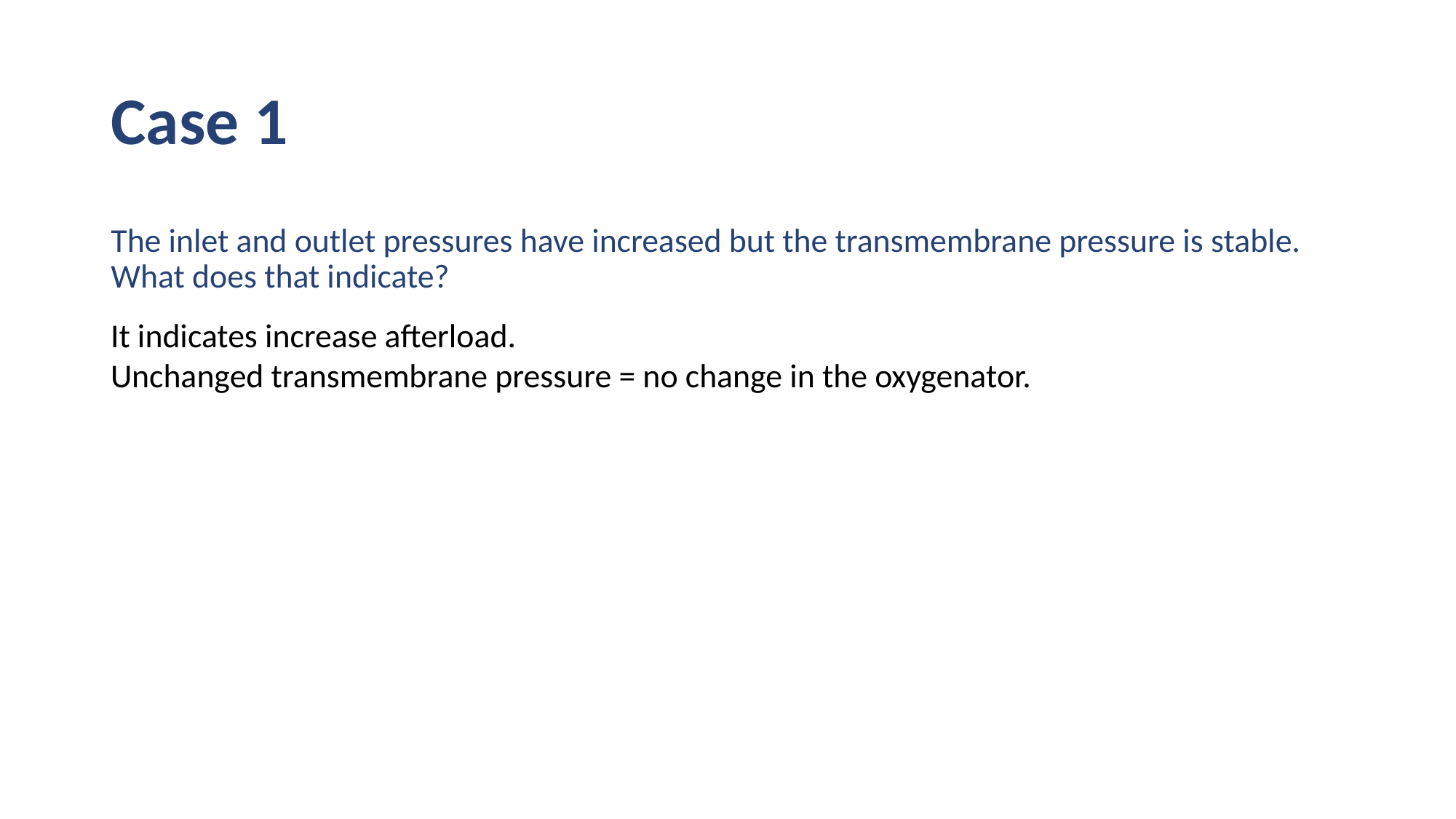

# Case 1
The inlet and outlet pressures have increased but the transmembrane pressure is stable. What does that indicate?
It indicates increase afterload.
Unchanged transmembrane pressure = no change in the oxygenator.

## Slide 17
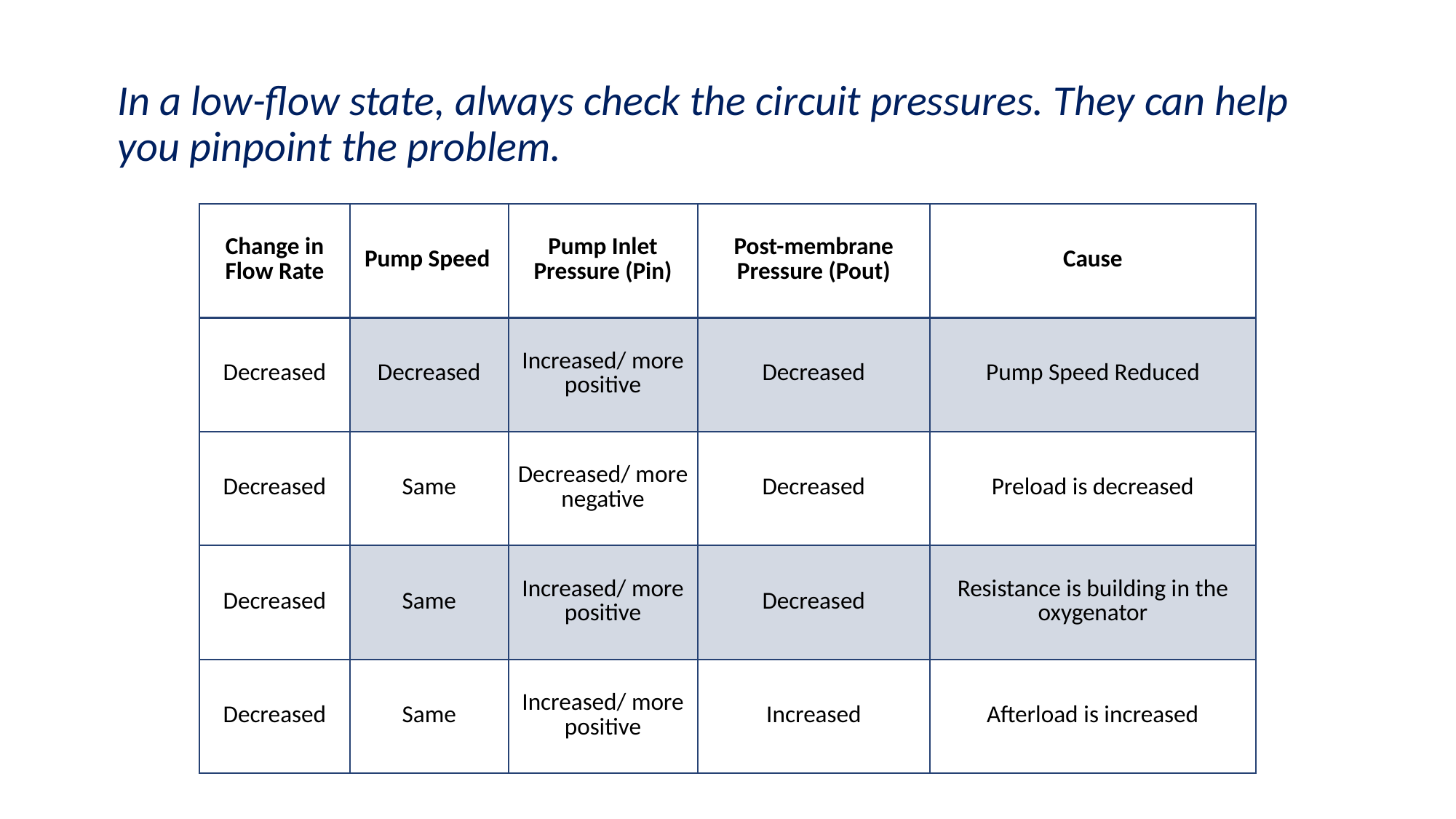

# In a low-flow state, always check the circuit pressures. They can help you pinpoint the problem.
| Change in Flow Rate | Pump Speed | Pump Inlet Pressure (Pin) | Post-membrane Pressure (Pout) | Cause |
| --- | --- | --- | --- | --- |
| Decreased | Decreased | Increased/ more positive | Decreased | Pump Speed Reduced |
| Decreased | Same | Decreased/ more negative | Decreased | Preload is decreased |
| Decreased | Same | Increased/ more positive | Decreased | Resistance is building in the oxygenator |
| Decreased | Same | Increased/ more positive | Increased | Afterload is increased |

## Slide 18
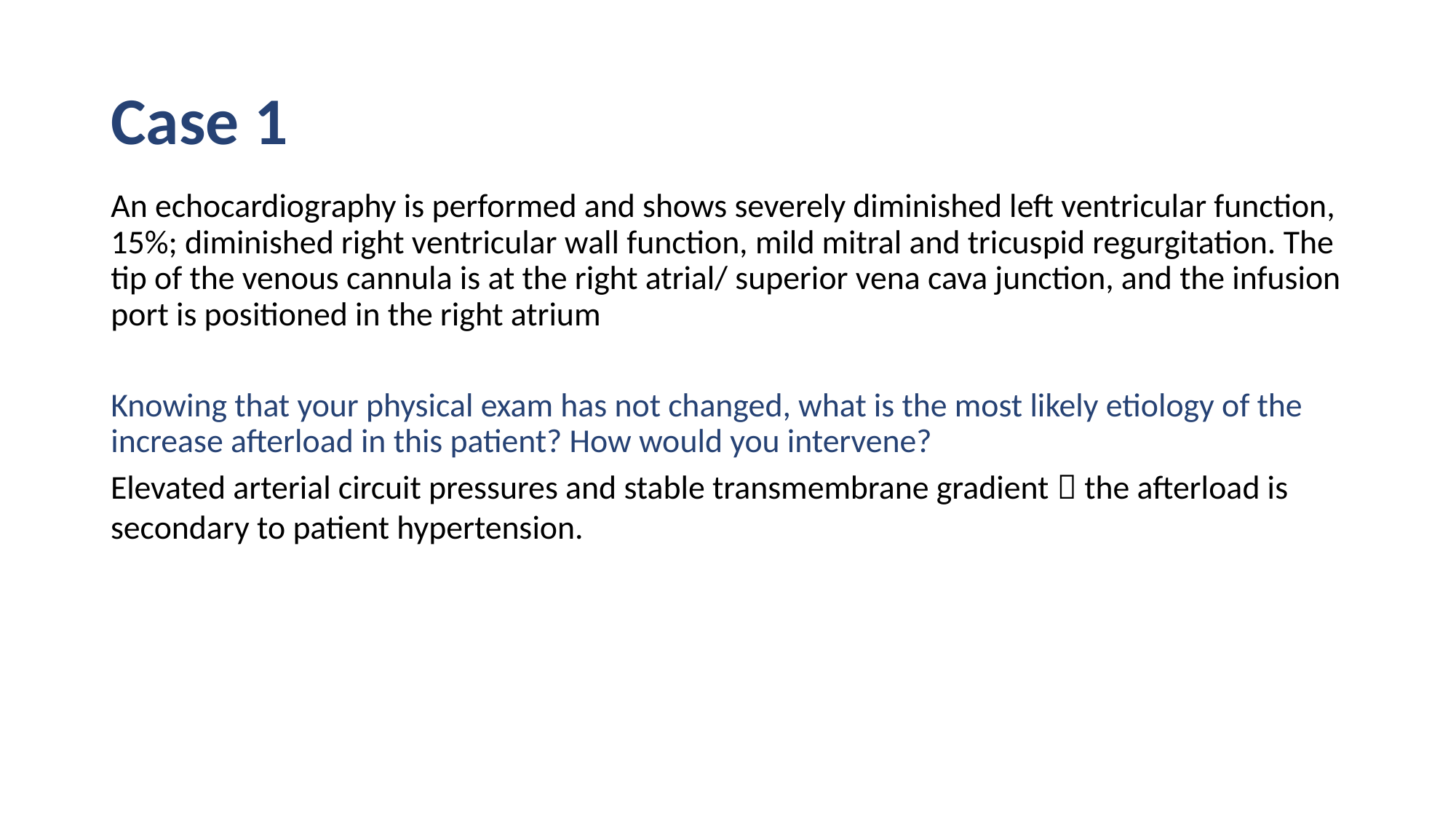

# Case 1
An echocardiography is performed and shows severely diminished left ventricular function, 15%; diminished right ventricular wall function, mild mitral and tricuspid regurgitation. The tip of the venous cannula is at the right atrial/ superior vena cava junction, and the infusion port is positioned in the right atrium
Knowing that your physical exam has not changed, what is the most likely etiology of the increase afterload in this patient? How would you intervene?
Elevated arterial circuit pressures and stable transmembrane gradient  the afterload is secondary to patient hypertension.

## Slide 19
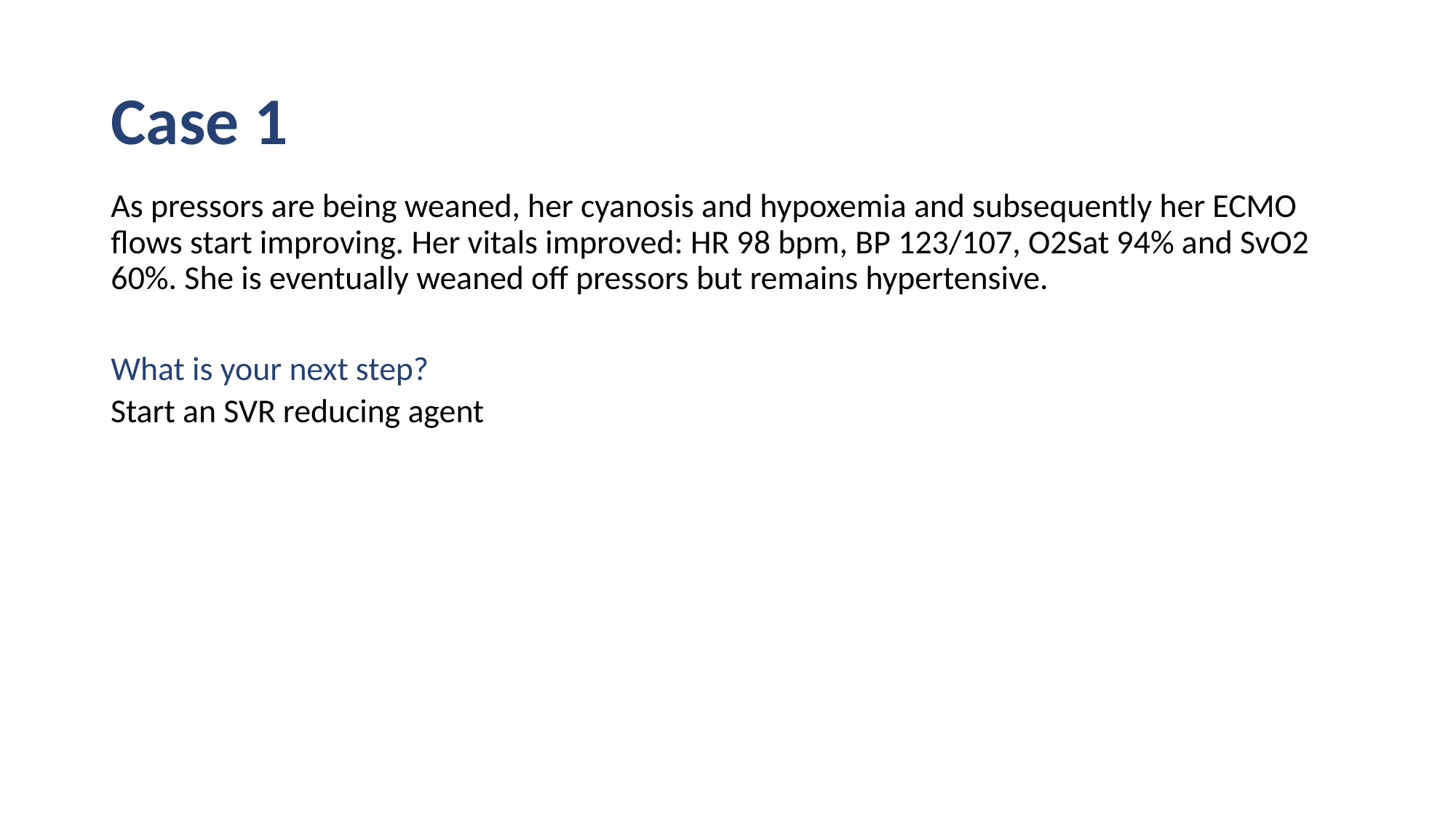

# Case 1
As pressors are being weaned, her cyanosis and hypoxemia and subsequently her ECMO flows start improving. Her vitals improved: HR 98 bpm, BP 123/107, O2Sat 94% and SvO2 60%. She is eventually weaned off pressors but remains hypertensive.
What is your next step?
Start an SVR reducing agent

## Slide 20
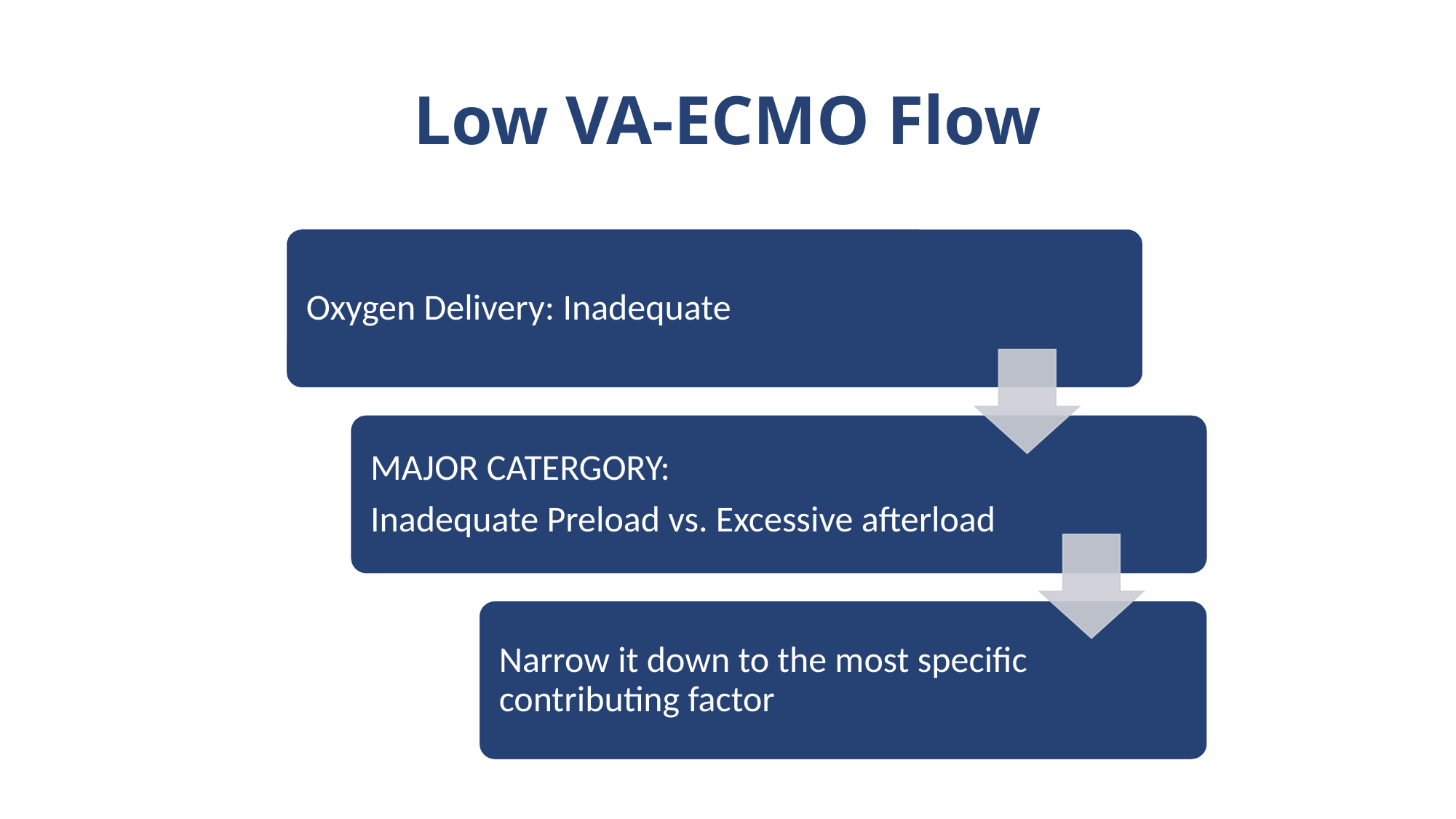

# Low VA-ECMO Flow

## Slide 21
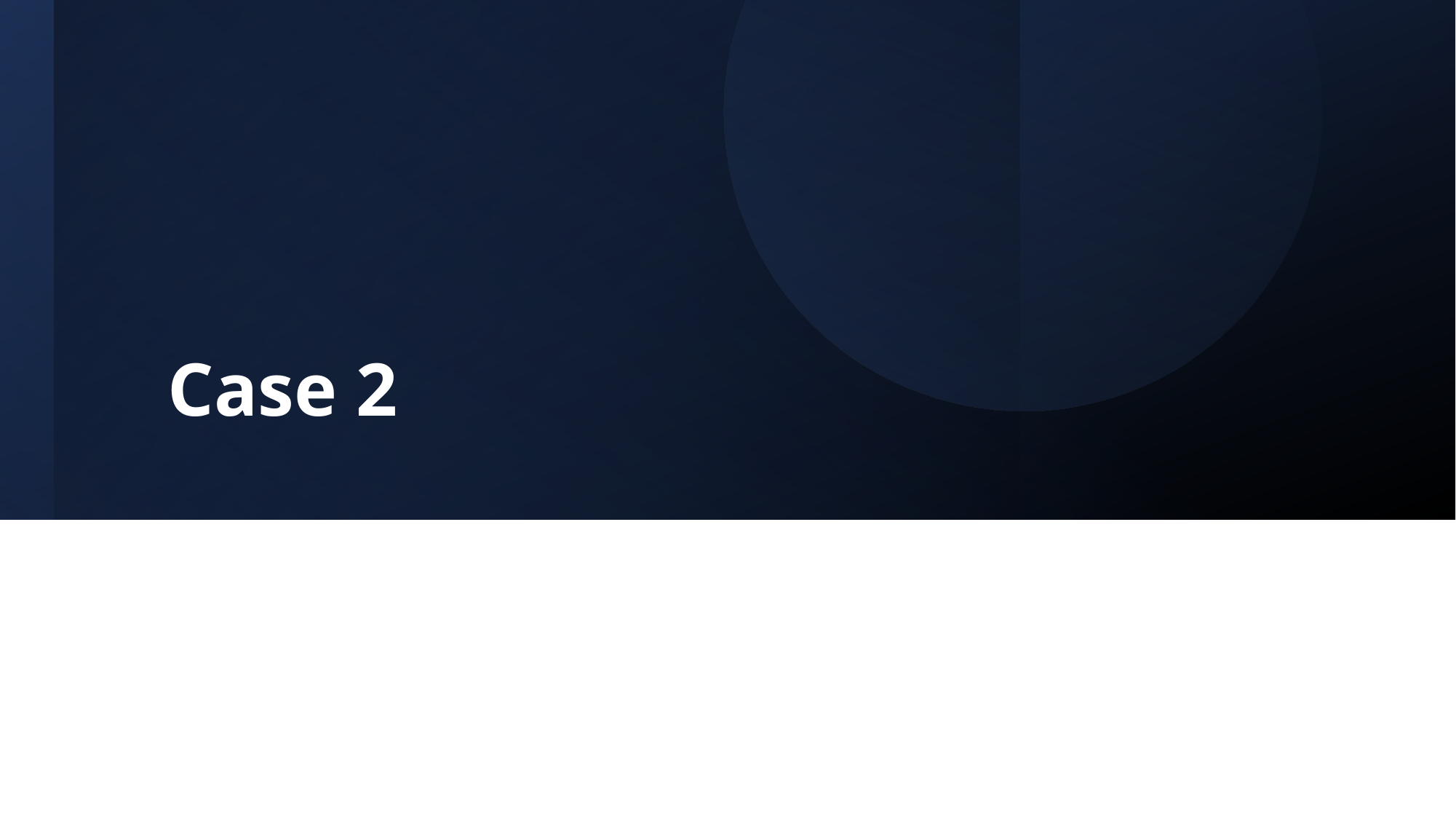

# Case 2

## Slide 22
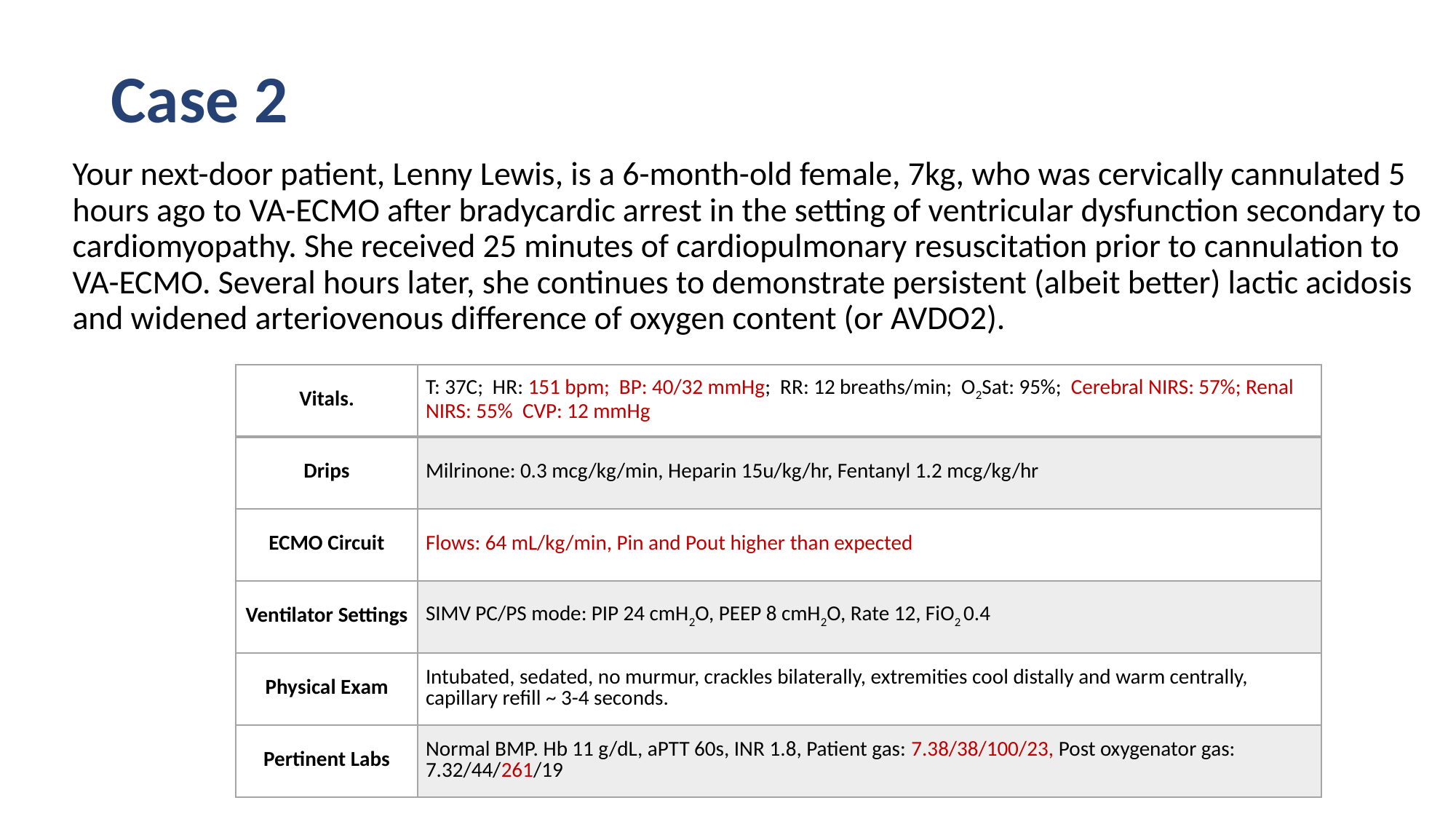

Case 2
Your next-door patient, Lenny Lewis, is a 6-month-old female, 7kg, who was cervically cannulated 5 hours ago to VA-ECMO after bradycardic arrest in the setting of ventricular dysfunction secondary to cardiomyopathy. She received 25 minutes of cardiopulmonary resuscitation prior to cannulation to VA-ECMO. Several hours later, she continues to demonstrate persistent (albeit better) lactic acidosis and widened arteriovenous difference of oxygen content (or AVDO2).
| Vitals. | T: 37C; HR: 151 bpm; BP: 40/32 mmHg; RR: 12 breaths/min; O2Sat: 95%; Cerebral NIRS: 57%; Renal NIRS: 55% CVP: 12 mmHg |
| --- | --- |
| Drips | Milrinone: 0.3 mcg/kg/min, Heparin 15u/kg/hr, Fentanyl 1.2 mcg/kg/hr |
| ECMO Circuit | Flows: 64 mL/kg/min, Pin and Pout higher than expected |
| Ventilator Settings | SIMV PC/PS mode: PIP 24 cmH2O, PEEP 8 cmH2O, Rate 12, FiO2 0.4 |
| Physical Exam | Intubated, sedated, no murmur, crackles bilaterally, extremities cool distally and warm centrally, capillary refill ~ 3-4 seconds. |
| Pertinent Labs | Normal BMP. Hb 11 g/dL, aPTT 60s, INR 1.8, Patient gas: 7.38/38/100/23, Post oxygenator gas: 7.32/44/261/19 |

## Slide 23
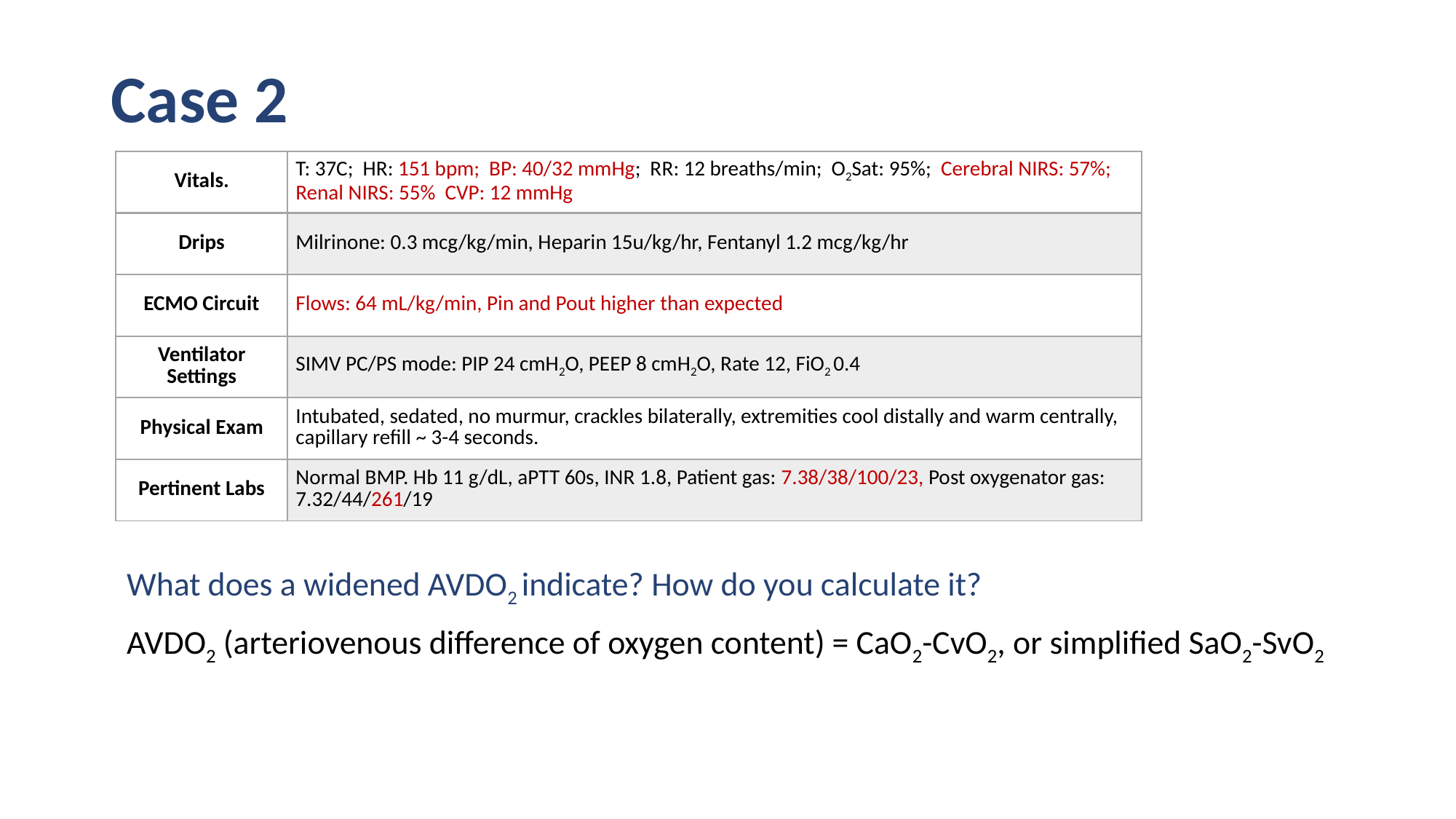

Case 2
| Vitals. | T: 37C; HR: 151 bpm; BP: 40/32 mmHg; RR: 12 breaths/min; O2Sat: 95%; Cerebral NIRS: 57%; Renal NIRS: 55% CVP: 12 mmHg |
| --- | --- |
| Drips | Milrinone: 0.3 mcg/kg/min, Heparin 15u/kg/hr, Fentanyl 1.2 mcg/kg/hr |
| ECMO Circuit | Flows: 64 mL/kg/min, Pin and Pout higher than expected |
| Ventilator Settings | SIMV PC/PS mode: PIP 24 cmH2O, PEEP 8 cmH2O, Rate 12, FiO2 0.4 |
| Physical Exam | Intubated, sedated, no murmur, crackles bilaterally, extremities cool distally and warm centrally, capillary refill ~ 3-4 seconds. |
| Pertinent Labs | Normal BMP. Hb 11 g/dL, aPTT 60s, INR 1.8, Patient gas: 7.38/38/100/23, Post oxygenator gas: 7.32/44/261/19 |
What does a widened AVDO2 indicate? How do you calculate it?
AVDO2 (arteriovenous difference of oxygen content) = CaO2-CvO2, or simplified SaO2-SvO2

## Slide 24
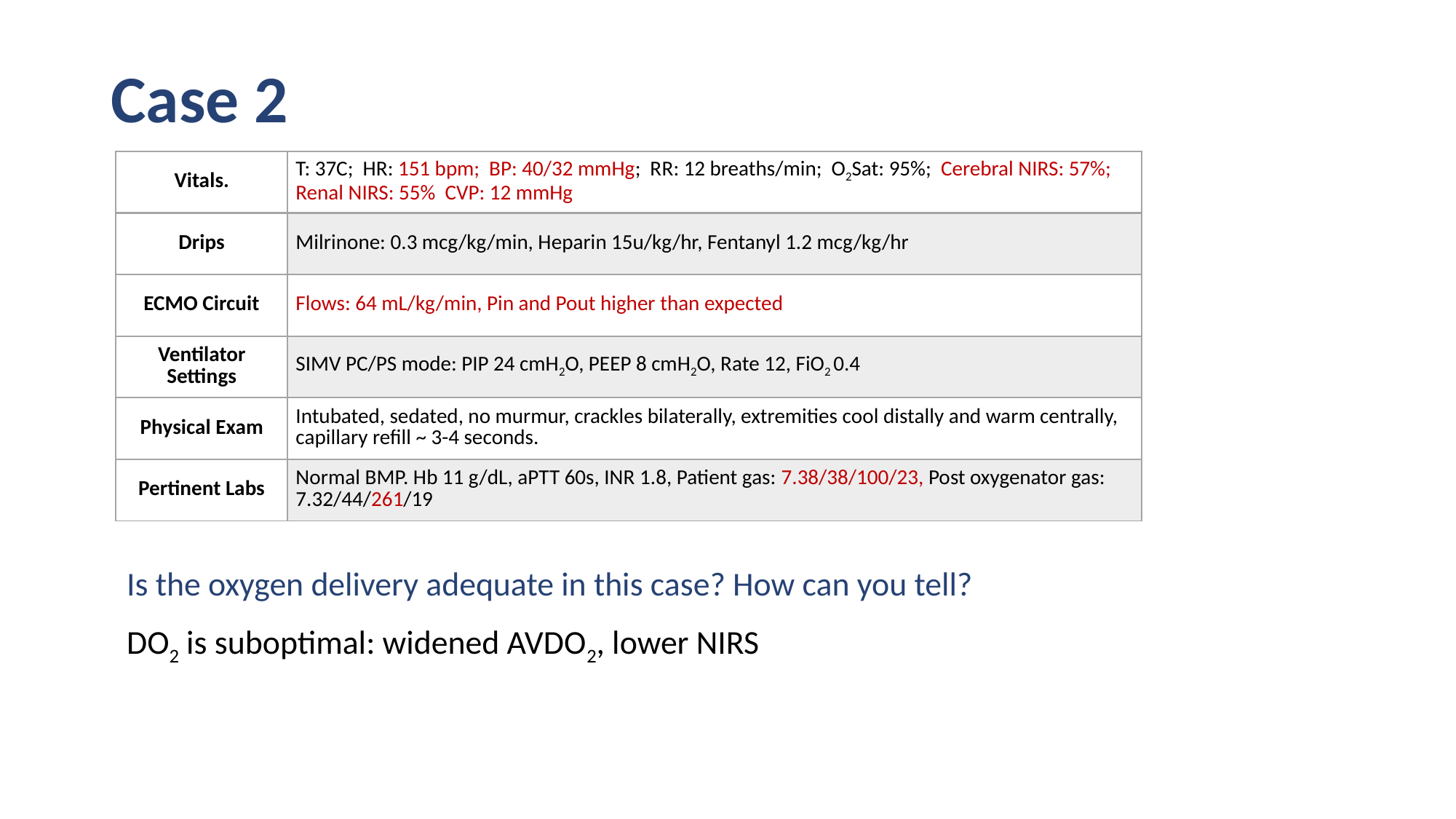

Case 2
| Vitals. | T: 37C; HR: 151 bpm; BP: 40/32 mmHg; RR: 12 breaths/min; O2Sat: 95%; Cerebral NIRS: 57%; Renal NIRS: 55% CVP: 12 mmHg |
| --- | --- |
| Drips | Milrinone: 0.3 mcg/kg/min, Heparin 15u/kg/hr, Fentanyl 1.2 mcg/kg/hr |
| ECMO Circuit | Flows: 64 mL/kg/min, Pin and Pout higher than expected |
| Ventilator Settings | SIMV PC/PS mode: PIP 24 cmH2O, PEEP 8 cmH2O, Rate 12, FiO2 0.4 |
| Physical Exam | Intubated, sedated, no murmur, crackles bilaterally, extremities cool distally and warm centrally, capillary refill ~ 3-4 seconds. |
| Pertinent Labs | Normal BMP. Hb 11 g/dL, aPTT 60s, INR 1.8, Patient gas: 7.38/38/100/23, Post oxygenator gas: 7.32/44/261/19 |
Is the oxygen delivery adequate in this case? How can you tell?
DO2 is suboptimal: widened AVDO2, lower NIRS

## Slide 25
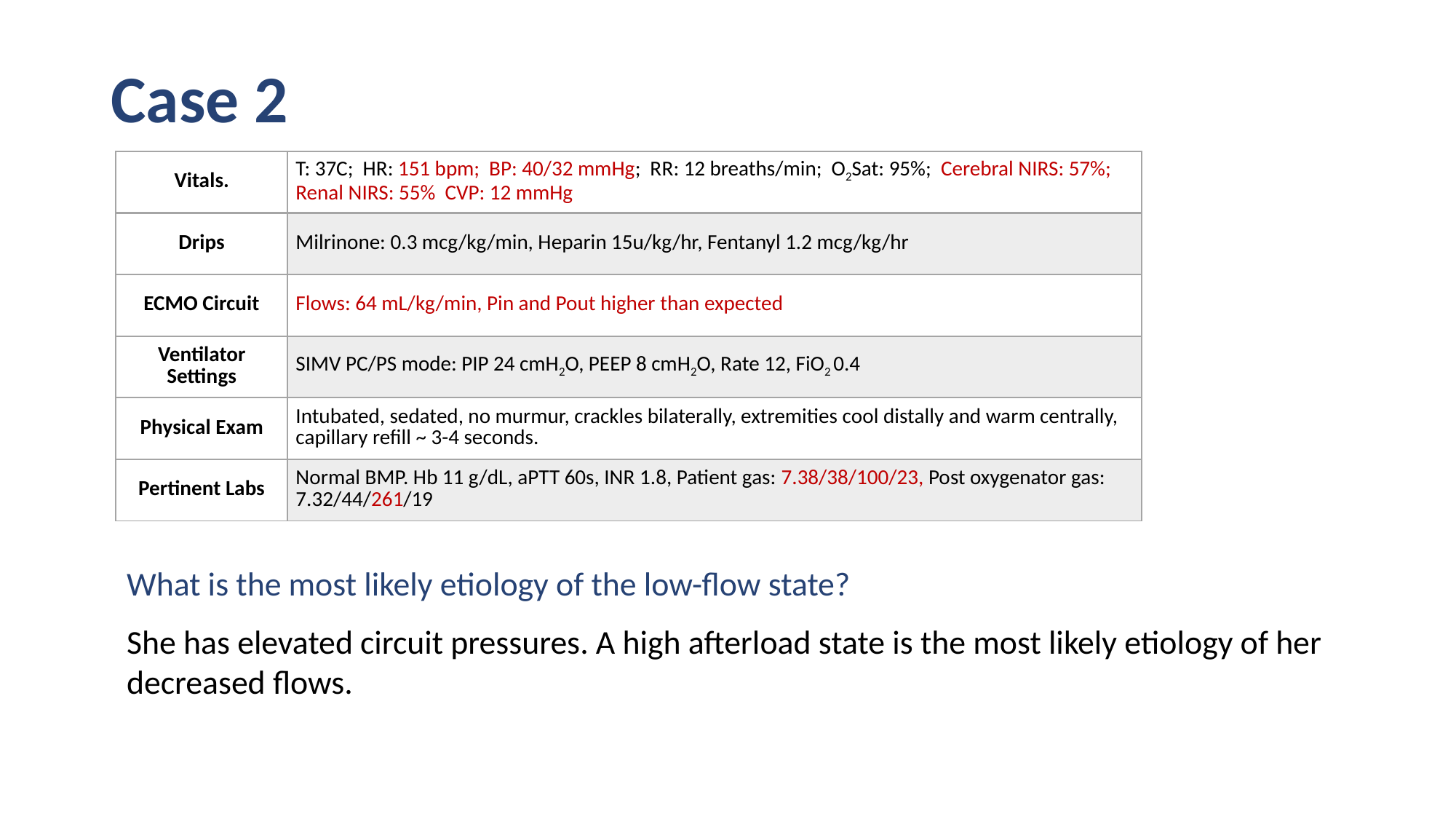

Case 2
| Vitals. | T: 37C; HR: 151 bpm; BP: 40/32 mmHg; RR: 12 breaths/min; O2Sat: 95%; Cerebral NIRS: 57%; Renal NIRS: 55% CVP: 12 mmHg |
| --- | --- |
| Drips | Milrinone: 0.3 mcg/kg/min, Heparin 15u/kg/hr, Fentanyl 1.2 mcg/kg/hr |
| ECMO Circuit | Flows: 64 mL/kg/min, Pin and Pout higher than expected |
| Ventilator Settings | SIMV PC/PS mode: PIP 24 cmH2O, PEEP 8 cmH2O, Rate 12, FiO2 0.4 |
| Physical Exam | Intubated, sedated, no murmur, crackles bilaterally, extremities cool distally and warm centrally, capillary refill ~ 3-4 seconds. |
| Pertinent Labs | Normal BMP. Hb 11 g/dL, aPTT 60s, INR 1.8, Patient gas: 7.38/38/100/23, Post oxygenator gas: 7.32/44/261/19 |
What is the most likely etiology of the low-flow state?
She has elevated circuit pressures. A high afterload state is the most likely etiology of her decreased flows.

## Slide 26
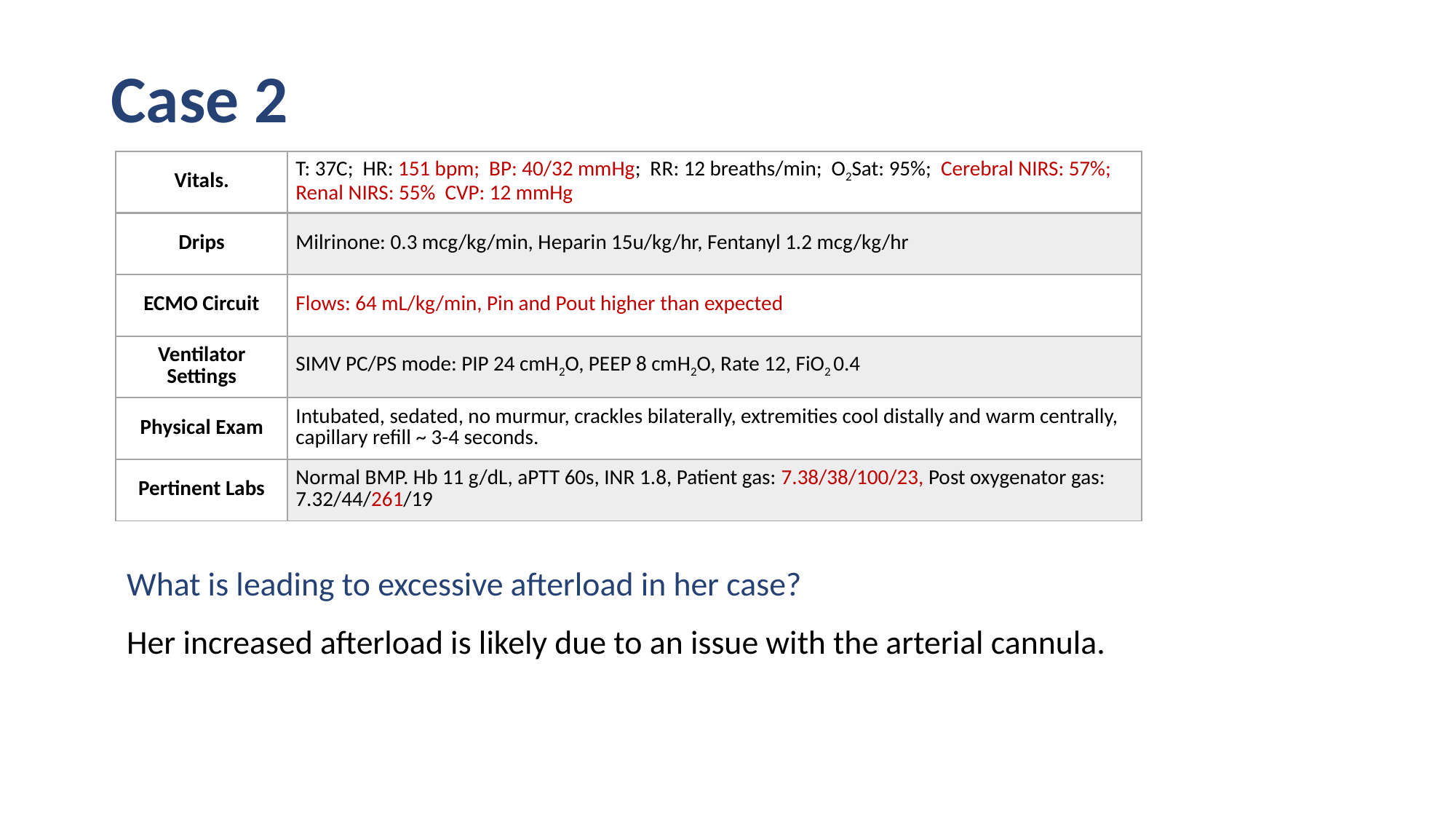

Case 2
| Vitals. | T: 37C; HR: 151 bpm; BP: 40/32 mmHg; RR: 12 breaths/min; O2Sat: 95%; Cerebral NIRS: 57%; Renal NIRS: 55% CVP: 12 mmHg |
| --- | --- |
| Drips | Milrinone: 0.3 mcg/kg/min, Heparin 15u/kg/hr, Fentanyl 1.2 mcg/kg/hr |
| ECMO Circuit | Flows: 64 mL/kg/min, Pin and Pout higher than expected |
| Ventilator Settings | SIMV PC/PS mode: PIP 24 cmH2O, PEEP 8 cmH2O, Rate 12, FiO2 0.4 |
| Physical Exam | Intubated, sedated, no murmur, crackles bilaterally, extremities cool distally and warm centrally, capillary refill ~ 3-4 seconds. |
| Pertinent Labs | Normal BMP. Hb 11 g/dL, aPTT 60s, INR 1.8, Patient gas: 7.38/38/100/23, Post oxygenator gas: 7.32/44/261/19 |
What is leading to excessive afterload in her case?
Her increased afterload is likely due to an issue with the arterial cannula.

## Slide 27
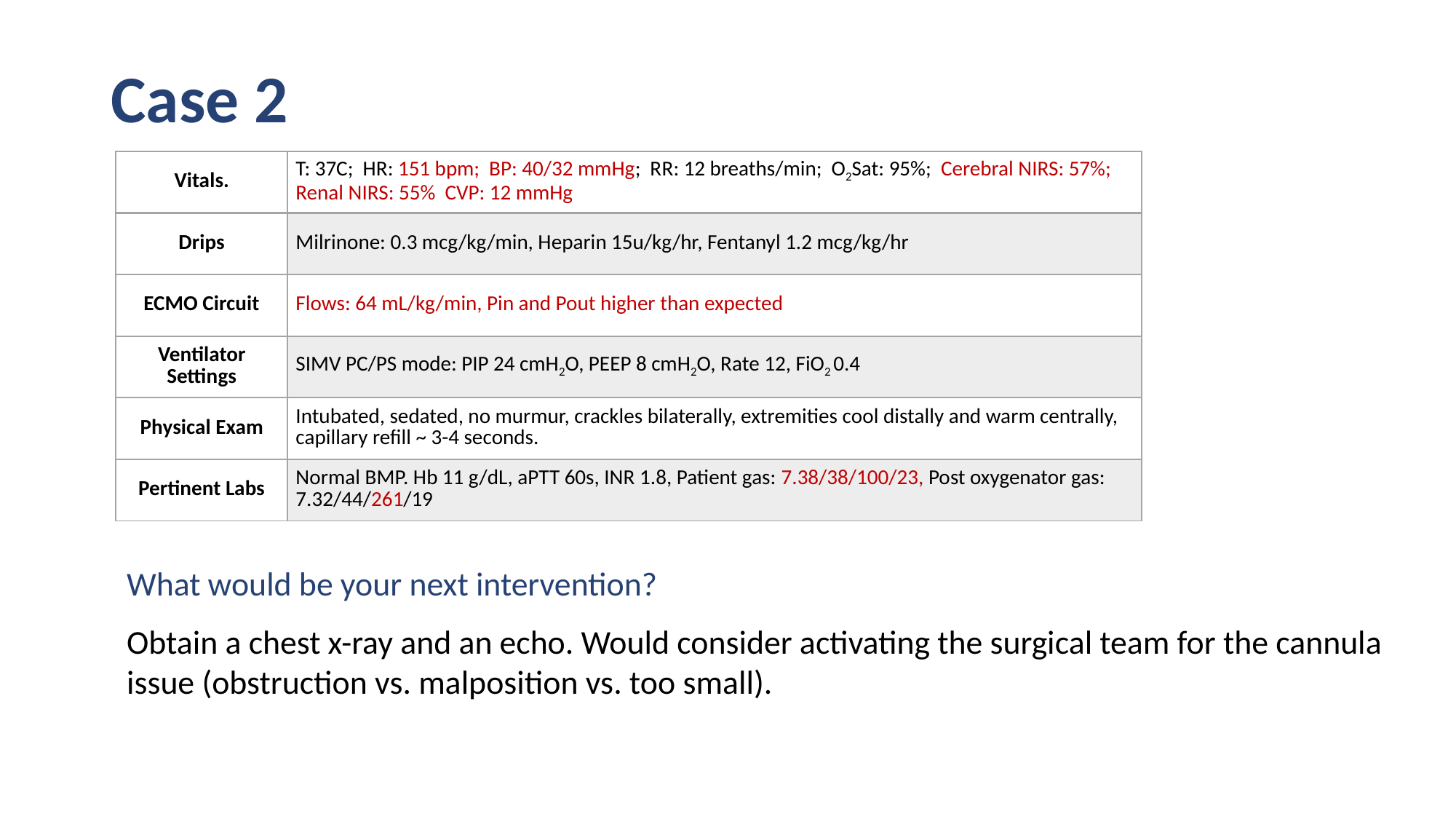

Case 2
| Vitals. | T: 37C; HR: 151 bpm; BP: 40/32 mmHg; RR: 12 breaths/min; O2Sat: 95%; Cerebral NIRS: 57%; Renal NIRS: 55% CVP: 12 mmHg |
| --- | --- |
| Drips | Milrinone: 0.3 mcg/kg/min, Heparin 15u/kg/hr, Fentanyl 1.2 mcg/kg/hr |
| ECMO Circuit | Flows: 64 mL/kg/min, Pin and Pout higher than expected |
| Ventilator Settings | SIMV PC/PS mode: PIP 24 cmH2O, PEEP 8 cmH2O, Rate 12, FiO2 0.4 |
| Physical Exam | Intubated, sedated, no murmur, crackles bilaterally, extremities cool distally and warm centrally, capillary refill ~ 3-4 seconds. |
| Pertinent Labs | Normal BMP. Hb 11 g/dL, aPTT 60s, INR 1.8, Patient gas: 7.38/38/100/23, Post oxygenator gas: 7.32/44/261/19 |
What would be your next intervention?
Obtain a chest x-ray and an echo. Would consider activating the surgical team for the cannula issue (obstruction vs. malposition vs. too small).

## Slide 28
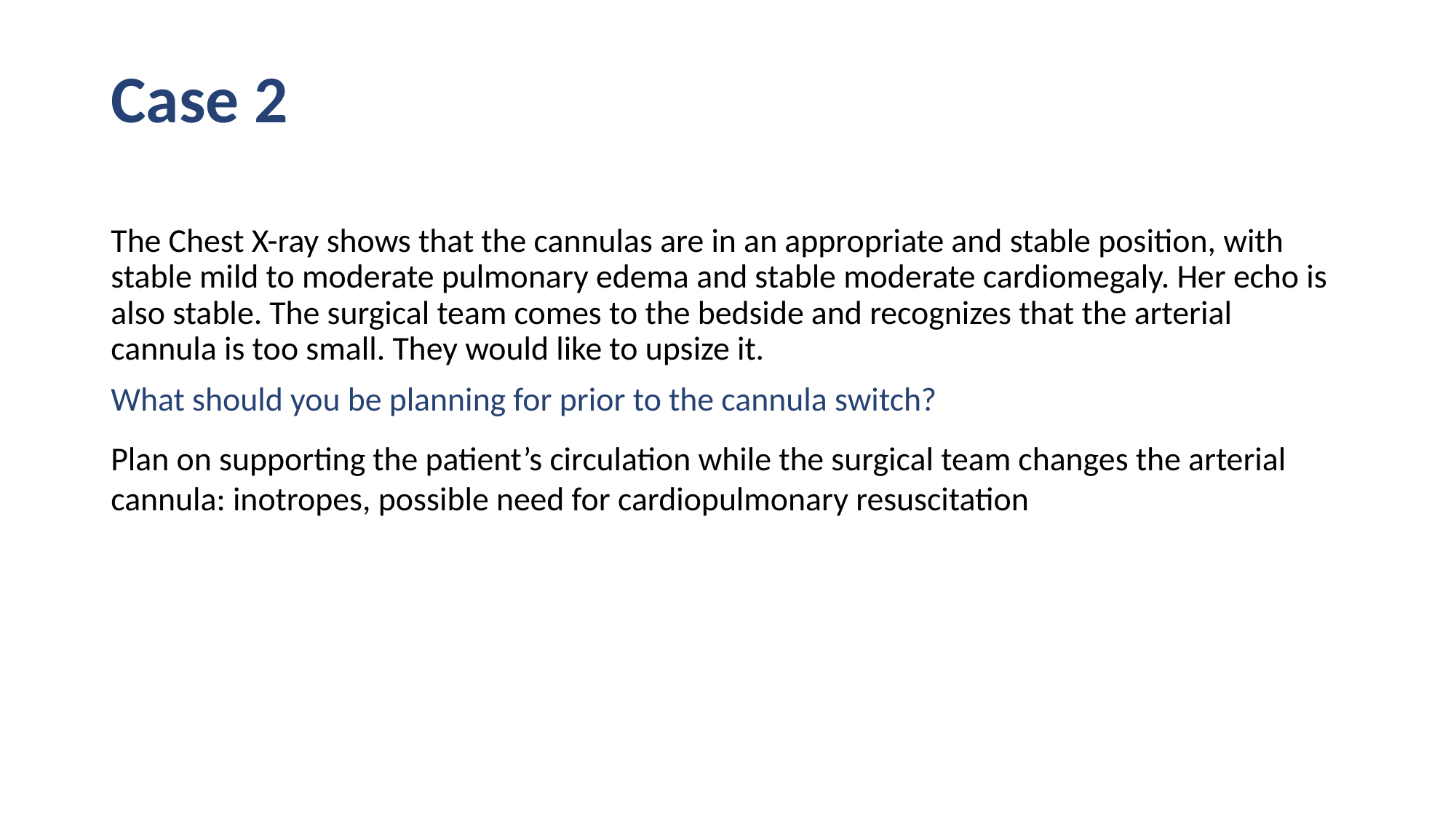

Case 2
The Chest X-ray shows that the cannulas are in an appropriate and stable position, with stable mild to moderate pulmonary edema and stable moderate cardiomegaly. Her echo is also stable. The surgical team comes to the bedside and recognizes that the arterial cannula is too small. They would like to upsize it.
What should you be planning for prior to the cannula switch?
Plan on supporting the patient’s circulation while the surgical team changes the arterial cannula: inotropes, possible need for cardiopulmonary resuscitation

## Slide 29
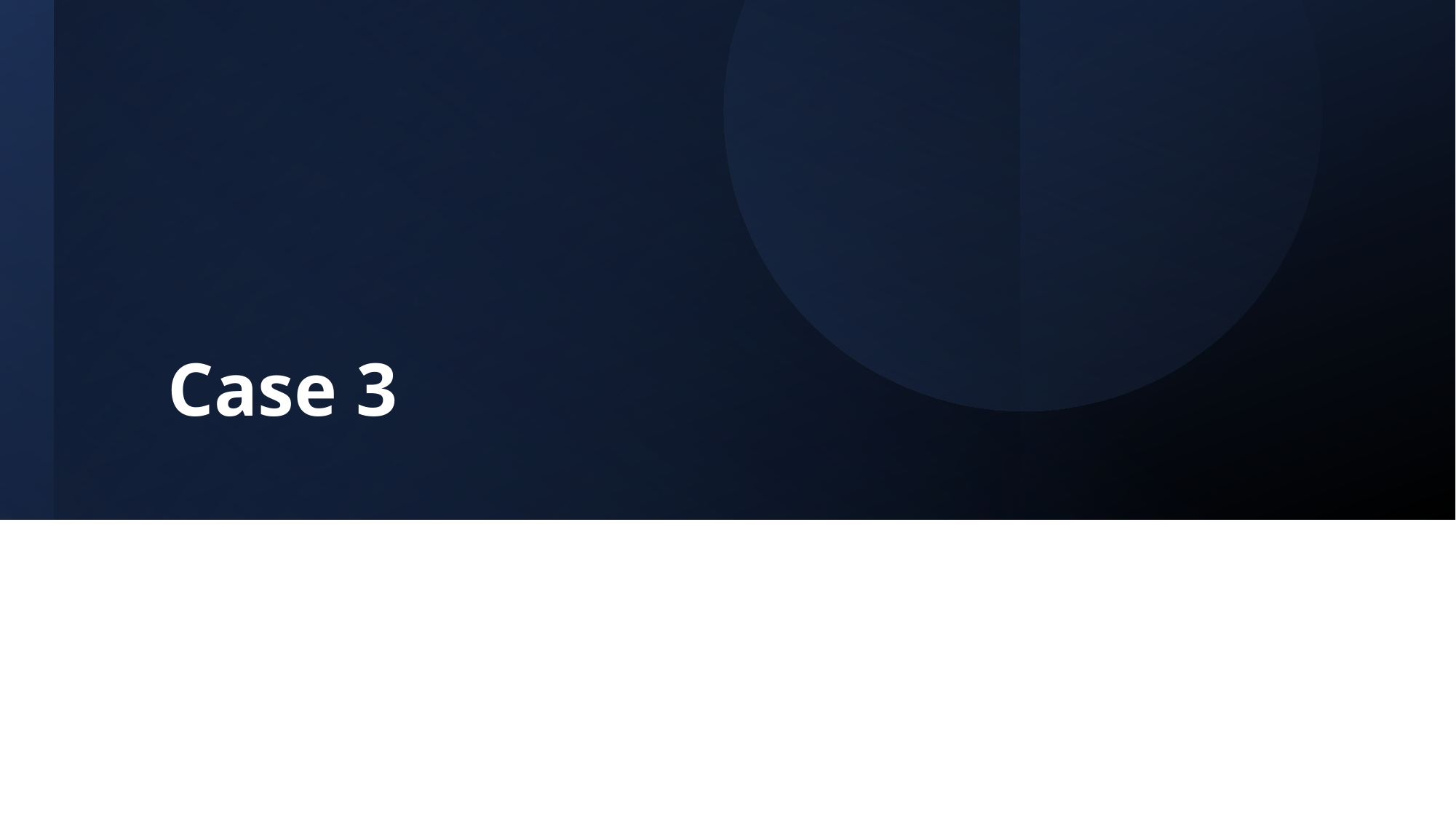

# Case 3

## Slide 30
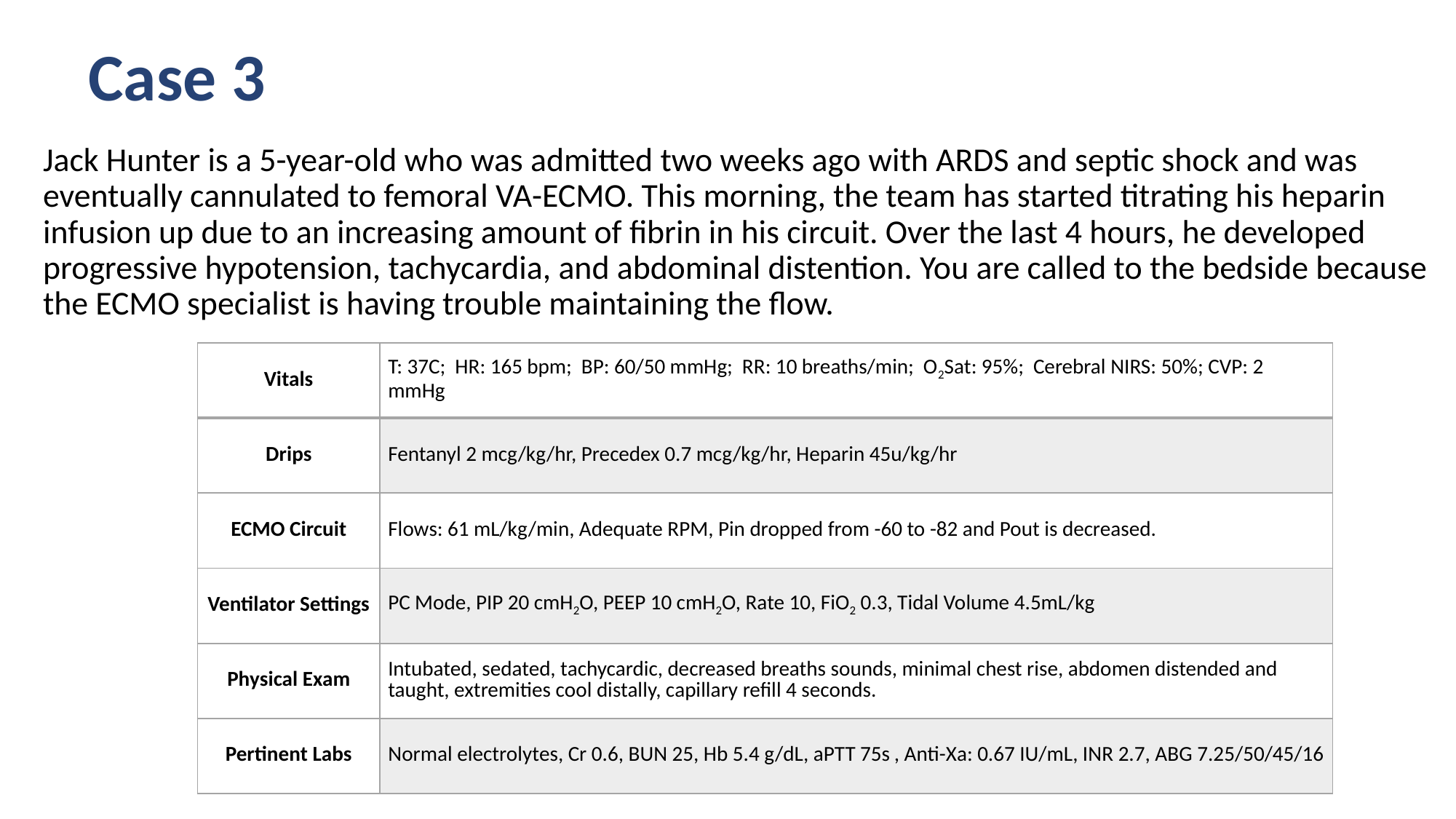

# Case 3
Jack Hunter is a 5-year-old who was admitted two weeks ago with ARDS and septic shock and was eventually cannulated to femoral VA-ECMO. This morning, the team has started titrating his heparin infusion up due to an increasing amount of fibrin in his circuit. Over the last 4 hours, he developed progressive hypotension, tachycardia, and abdominal distention. You are called to the bedside because the ECMO specialist is having trouble maintaining the flow.
| Vitals | T: 37C; HR: 165 bpm; BP: 60/50 mmHg; RR: 10 breaths/min; O2Sat: 95%; Cerebral NIRS: 50%; CVP: 2 mmHg |
| --- | --- |
| Drips | Fentanyl 2 mcg/kg/hr, Precedex 0.7 mcg/kg/hr, Heparin 45u/kg/hr |
| ECMO Circuit | Flows: 61 mL/kg/min, Adequate RPM, Pin dropped from -60 to -82 and Pout is decreased. |
| Ventilator Settings | PC Mode, PIP 20 cmH2O, PEEP 10 cmH2O, Rate 10, FiO2 0.3, Tidal Volume 4.5mL/kg |
| Physical Exam | Intubated, sedated, tachycardic, decreased breaths sounds, minimal chest rise, abdomen distended and taught, extremities cool distally, capillary refill 4 seconds. |
| Pertinent Labs | Normal electrolytes, Cr 0.6, BUN 25, Hb 5.4 g/dL, aPTT 75s , Anti-Xa: 0.67 IU/mL, INR 2.7, ABG 7.25/50/45/16 |

## Slide 31
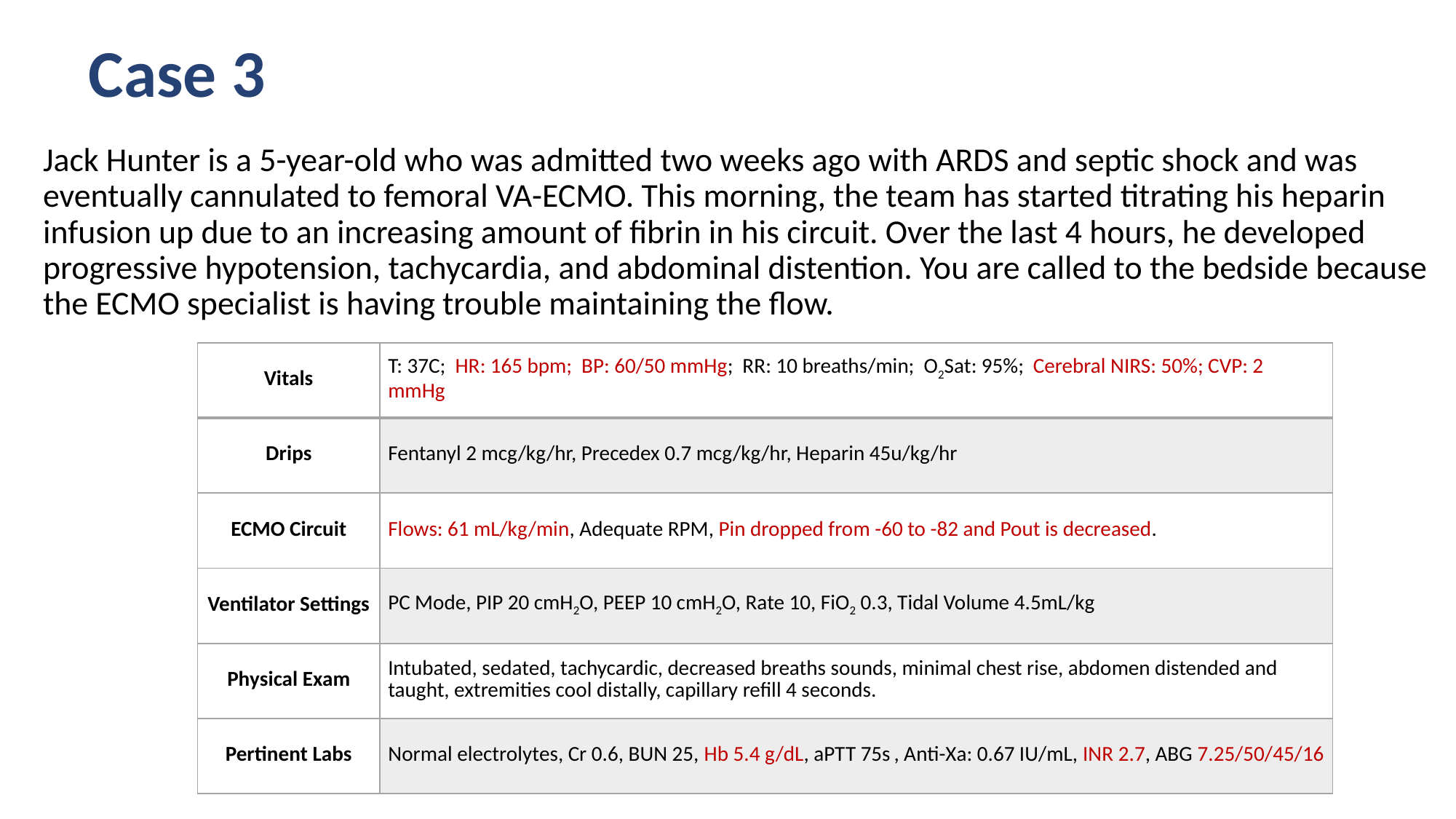

Case 3
Jack Hunter is a 5-year-old who was admitted two weeks ago with ARDS and septic shock and was eventually cannulated to femoral VA-ECMO. This morning, the team has started titrating his heparin infusion up due to an increasing amount of fibrin in his circuit. Over the last 4 hours, he developed progressive hypotension, tachycardia, and abdominal distention. You are called to the bedside because the ECMO specialist is having trouble maintaining the flow.
| Vitals | T: 37C; HR: 165 bpm; BP: 60/50 mmHg; RR: 10 breaths/min; O2Sat: 95%; Cerebral NIRS: 50%; CVP: 2 mmHg |
| --- | --- |
| Drips | Fentanyl 2 mcg/kg/hr, Precedex 0.7 mcg/kg/hr, Heparin 45u/kg/hr |
| ECMO Circuit | Flows: 61 mL/kg/min, Adequate RPM, Pin dropped from -60 to -82 and Pout is decreased. |
| Ventilator Settings | PC Mode, PIP 20 cmH2O, PEEP 10 cmH2O, Rate 10, FiO2 0.3, Tidal Volume 4.5mL/kg |
| Physical Exam | Intubated, sedated, tachycardic, decreased breaths sounds, minimal chest rise, abdomen distended and taught, extremities cool distally, capillary refill 4 seconds. |
| Pertinent Labs | Normal electrolytes, Cr 0.6, BUN 25, Hb 5.4 g/dL, aPTT 75s , Anti-Xa: 0.67 IU/mL, INR 2.7, ABG 7.25/50/45/16 |

## Slide 32
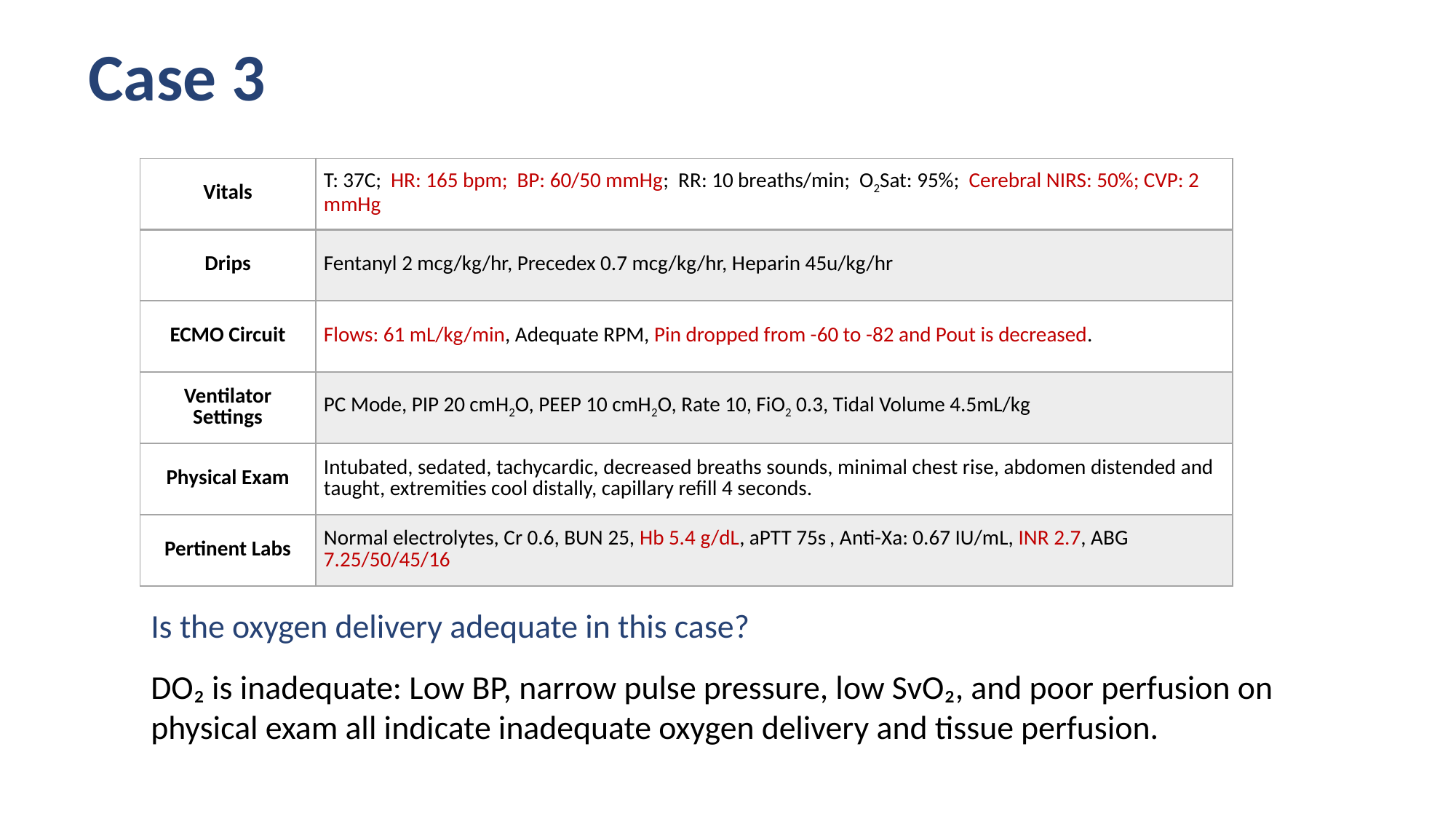

# Case 3
| Vitals | T: 37C; HR: 165 bpm; BP: 60/50 mmHg; RR: 10 breaths/min; O2Sat: 95%; Cerebral NIRS: 50%; CVP: 2 mmHg |
| --- | --- |
| Drips | Fentanyl 2 mcg/kg/hr, Precedex 0.7 mcg/kg/hr, Heparin 45u/kg/hr |
| ECMO Circuit | Flows: 61 mL/kg/min, Adequate RPM, Pin dropped from -60 to -82 and Pout is decreased. |
| Ventilator Settings | PC Mode, PIP 20 cmH2O, PEEP 10 cmH2O, Rate 10, FiO2 0.3, Tidal Volume 4.5mL/kg |
| Physical Exam | Intubated, sedated, tachycardic, decreased breaths sounds, minimal chest rise, abdomen distended and taught, extremities cool distally, capillary refill 4 seconds. |
| Pertinent Labs | Normal electrolytes, Cr 0.6, BUN 25, Hb 5.4 g/dL, aPTT 75s , Anti-Xa: 0.67 IU/mL, INR 2.7, ABG 7.25/50/45/16 |
Is the oxygen delivery adequate in this case?
DO₂ is inadequate: Low BP, narrow pulse pressure, low SvO₂, and poor perfusion on physical exam all indicate inadequate oxygen delivery and tissue perfusion.

## Slide 33
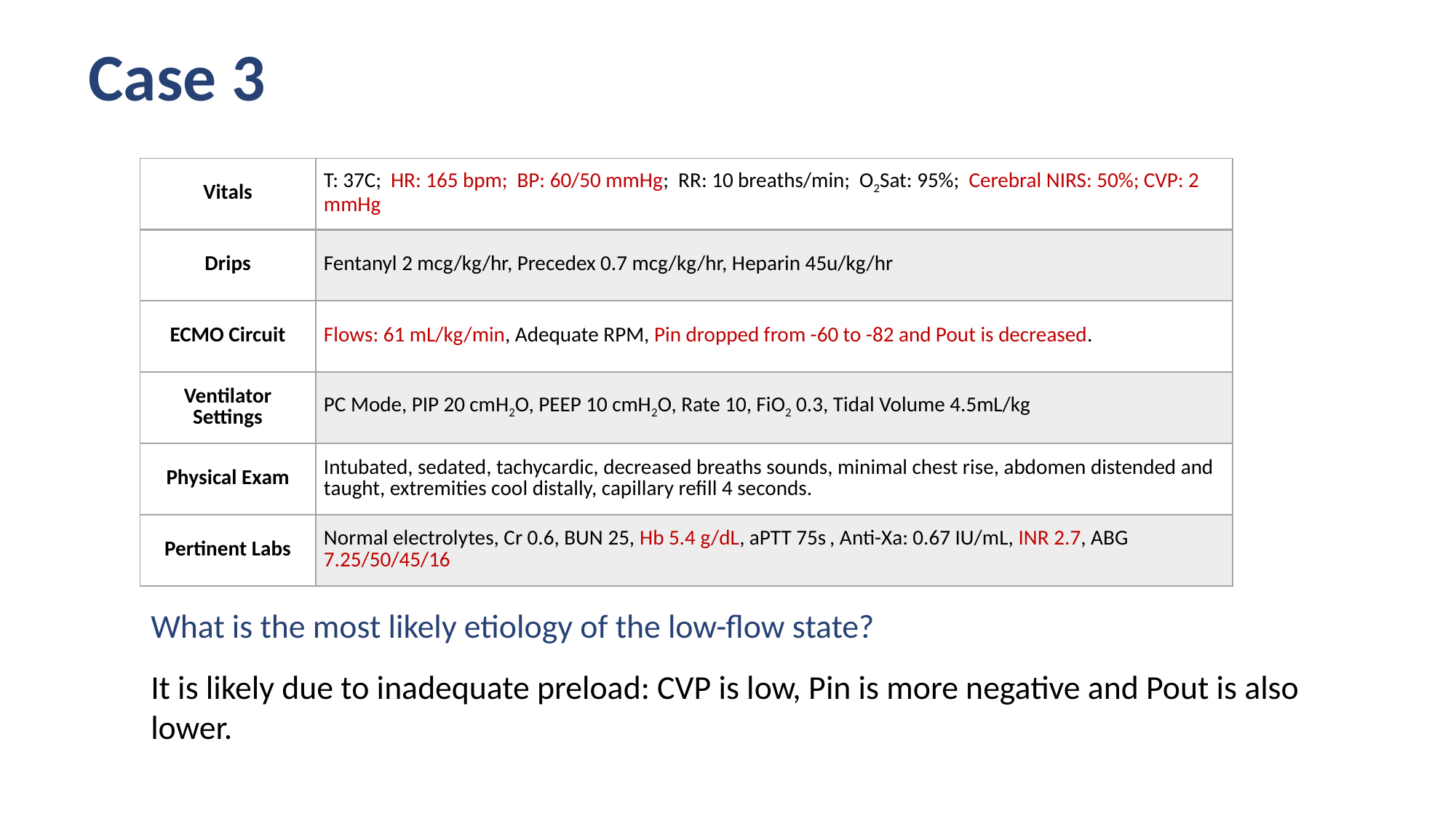

# Case 3
| Vitals | T: 37C; HR: 165 bpm; BP: 60/50 mmHg; RR: 10 breaths/min; O2Sat: 95%; Cerebral NIRS: 50%; CVP: 2 mmHg |
| --- | --- |
| Drips | Fentanyl 2 mcg/kg/hr, Precedex 0.7 mcg/kg/hr, Heparin 45u/kg/hr |
| ECMO Circuit | Flows: 61 mL/kg/min, Adequate RPM, Pin dropped from -60 to -82 and Pout is decreased. |
| Ventilator Settings | PC Mode, PIP 20 cmH2O, PEEP 10 cmH2O, Rate 10, FiO2 0.3, Tidal Volume 4.5mL/kg |
| Physical Exam | Intubated, sedated, tachycardic, decreased breaths sounds, minimal chest rise, abdomen distended and taught, extremities cool distally, capillary refill 4 seconds. |
| Pertinent Labs | Normal electrolytes, Cr 0.6, BUN 25, Hb 5.4 g/dL, aPTT 75s , Anti-Xa: 0.67 IU/mL, INR 2.7, ABG 7.25/50/45/16 |
What is the most likely etiology of the low-flow state?
It is likely due to inadequate preload: CVP is low, Pin is more negative and Pout is also lower.

## Slide 34
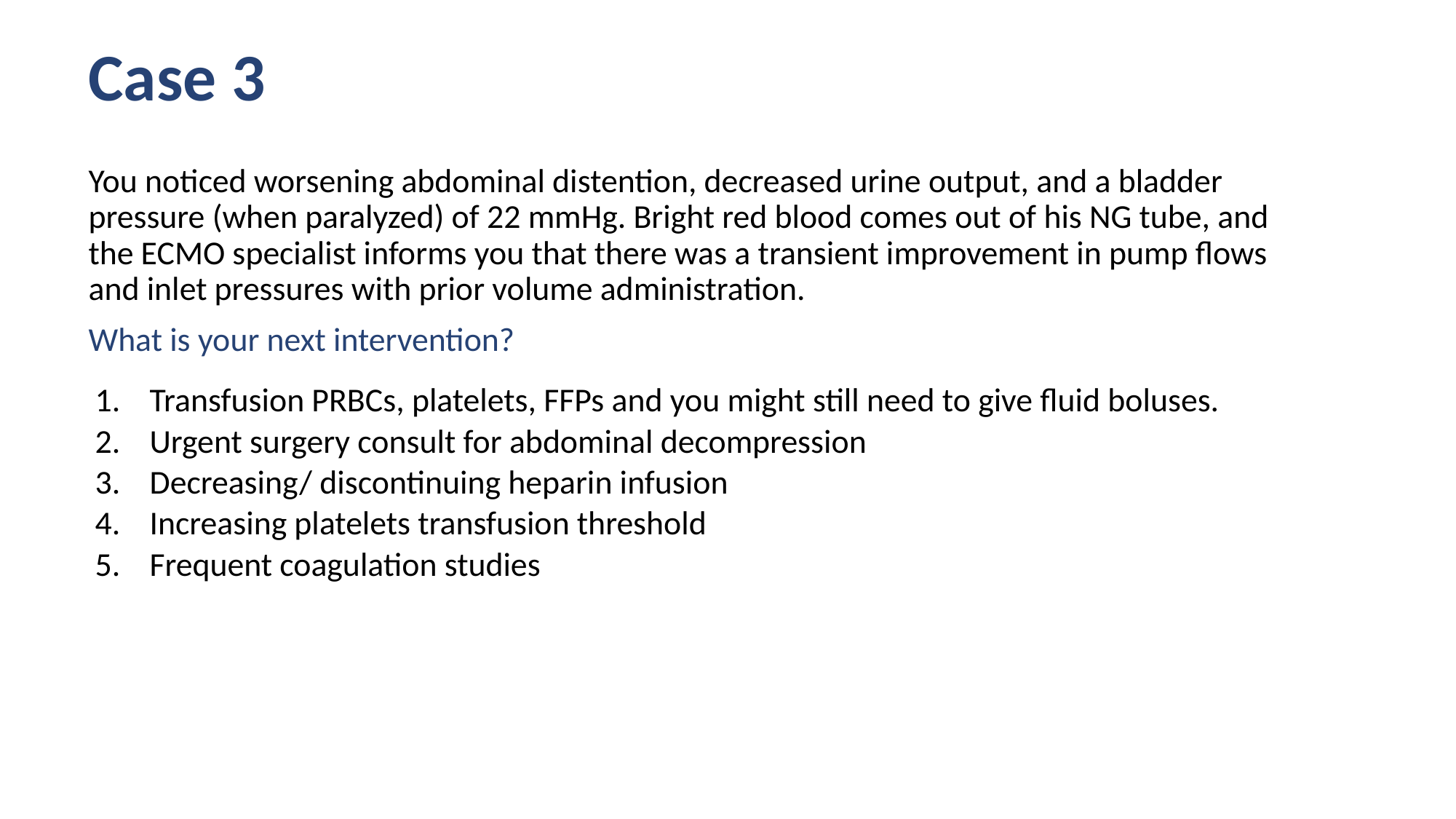

# Case 3
You noticed worsening abdominal distention, decreased urine output, and a bladder pressure (when paralyzed) of 22 mmHg. Bright red blood comes out of his NG tube, and the ECMO specialist informs you that there was a transient improvement in pump flows and inlet pressures with prior volume administration.
What is your next intervention?
Transfusion PRBCs, platelets, FFPs and you might still need to give fluid boluses.
Urgent surgery consult for abdominal decompression
Decreasing/ discontinuing heparin infusion
Increasing platelets transfusion threshold
Frequent coagulation studies

## Slide 35
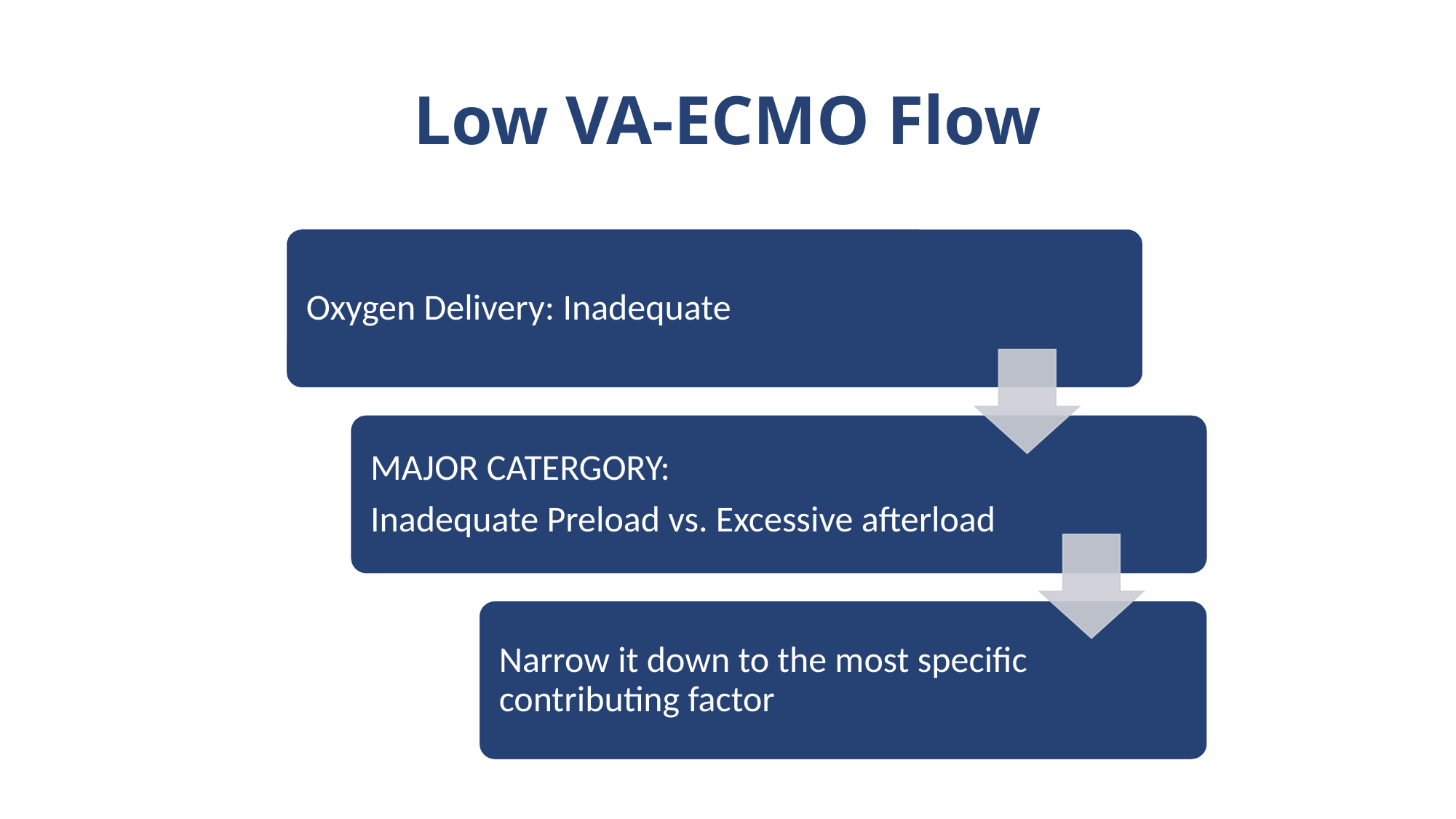

# Low VA-ECMO Flow

## Slide 36
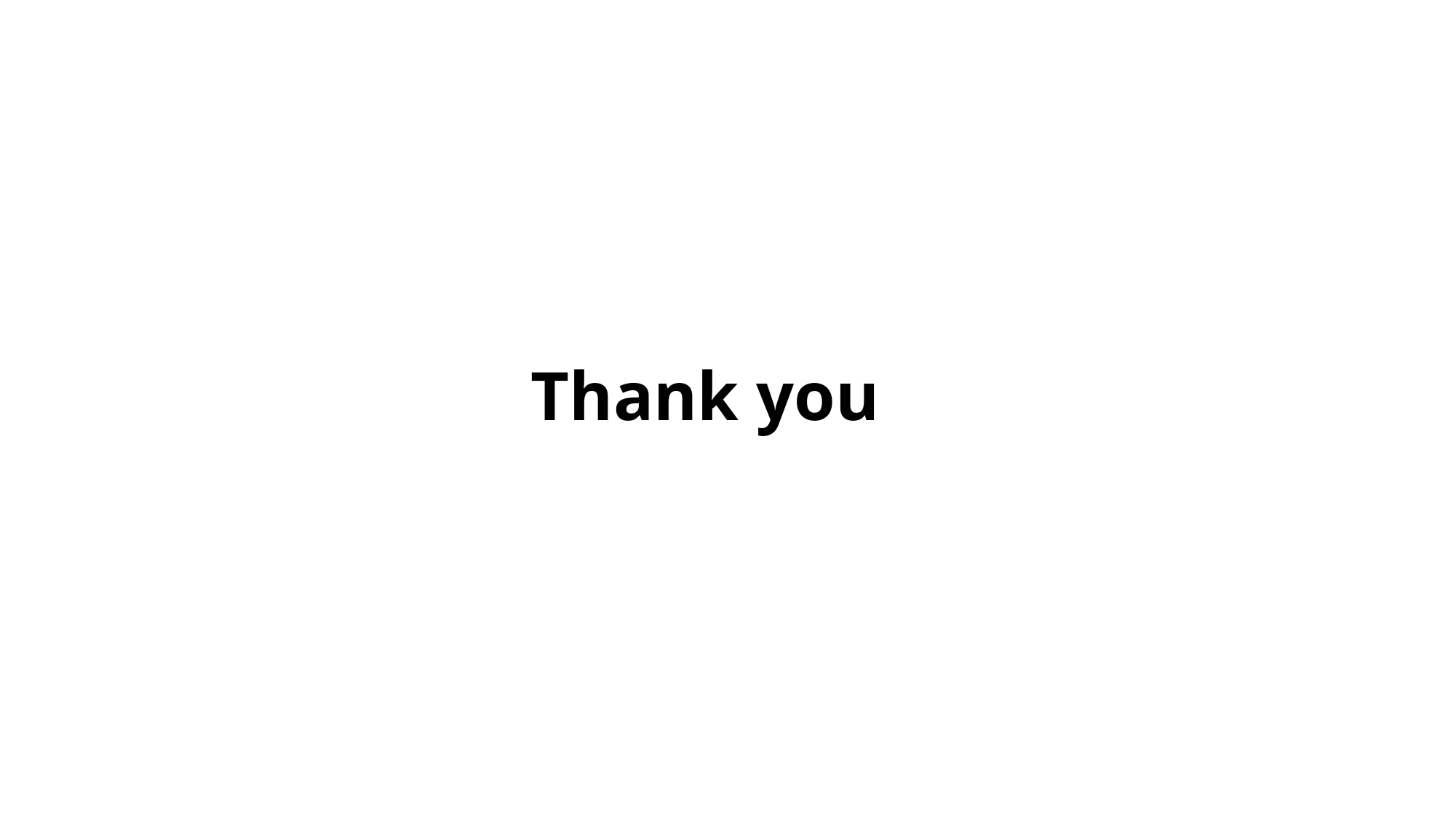

# Thank you

## Slide 37
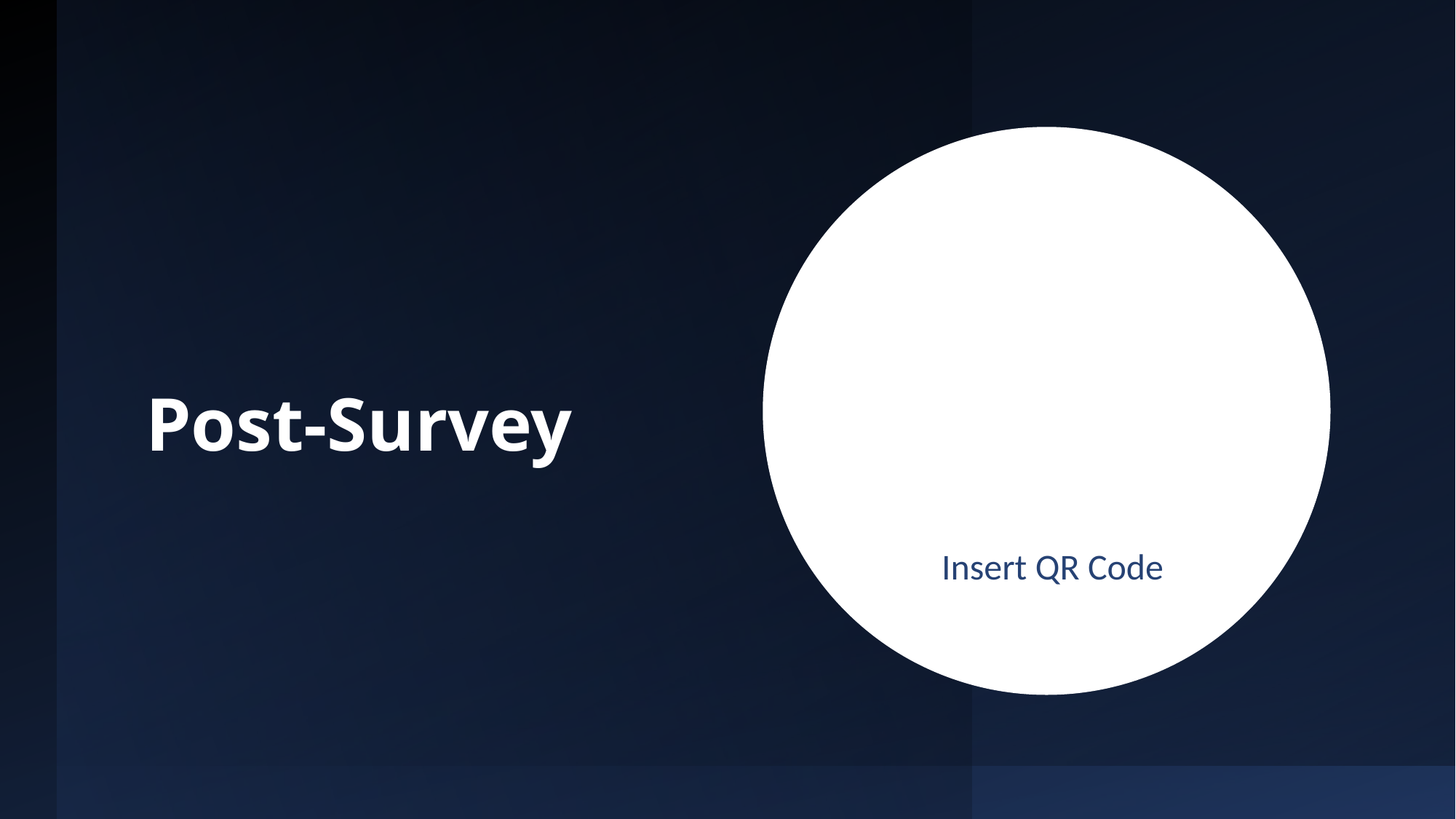

# Post-Survey
Insert QR Code
