# Supplementary material for: Enhancing Pediatric Extracorporeal Membrane Oxygenation Education Through Process-Oriented Guided Inquiry Learning Sessions for Fellows and Advanced Practice Providers
Source: MedEdPORTAL. 2026 May 12;22:11600. doi: 10.15766/mep_2374-8265.11600 (PMC13161199; doi:10.15766/mep_2374-8265.11600)
Supplement: Supplementary file 1 — VA-ECMO Learner Handout.docxVV-ECMO Learner Handout.docxVA-ECMO Facilitator Guide.docxVV-ECMO Facilitator Guide.docxVA-ECMO Slides.pptxVV-ECMO Slides.pptxVA-ECMO Presurvey.docxVV-ECMO Presurvey.docxVA-ECMO Postsurvey.docxVV-ECMO Postsurvey.docx [file mep_2374-8265.11600-s001.zip › F. VV-ECMO Slides.pptx]

## Slide 1
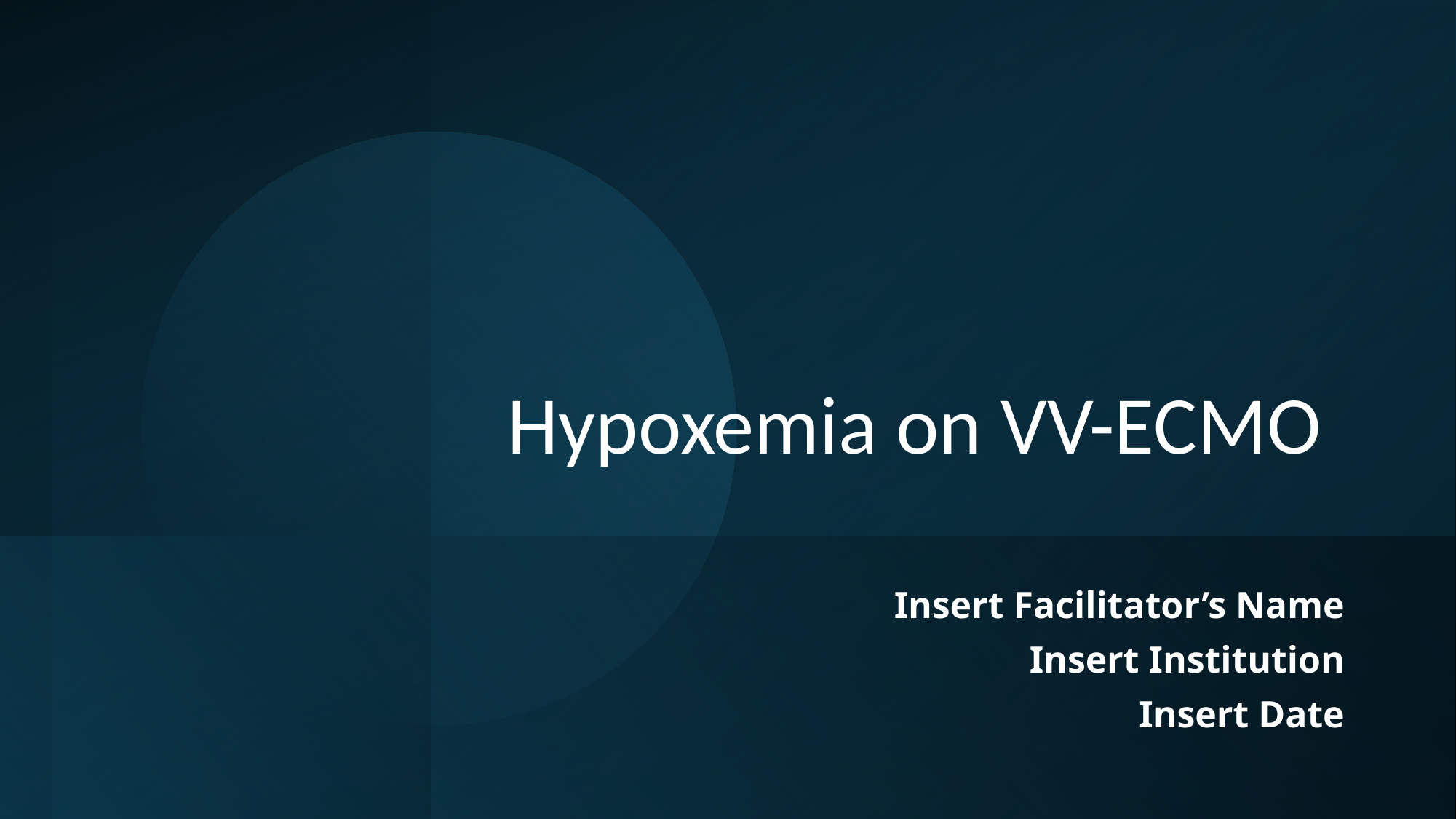

# Hypoxemia on VV-ECMO
Insert Facilitator’s Name
Insert Institution
Insert Date

## Slide 2
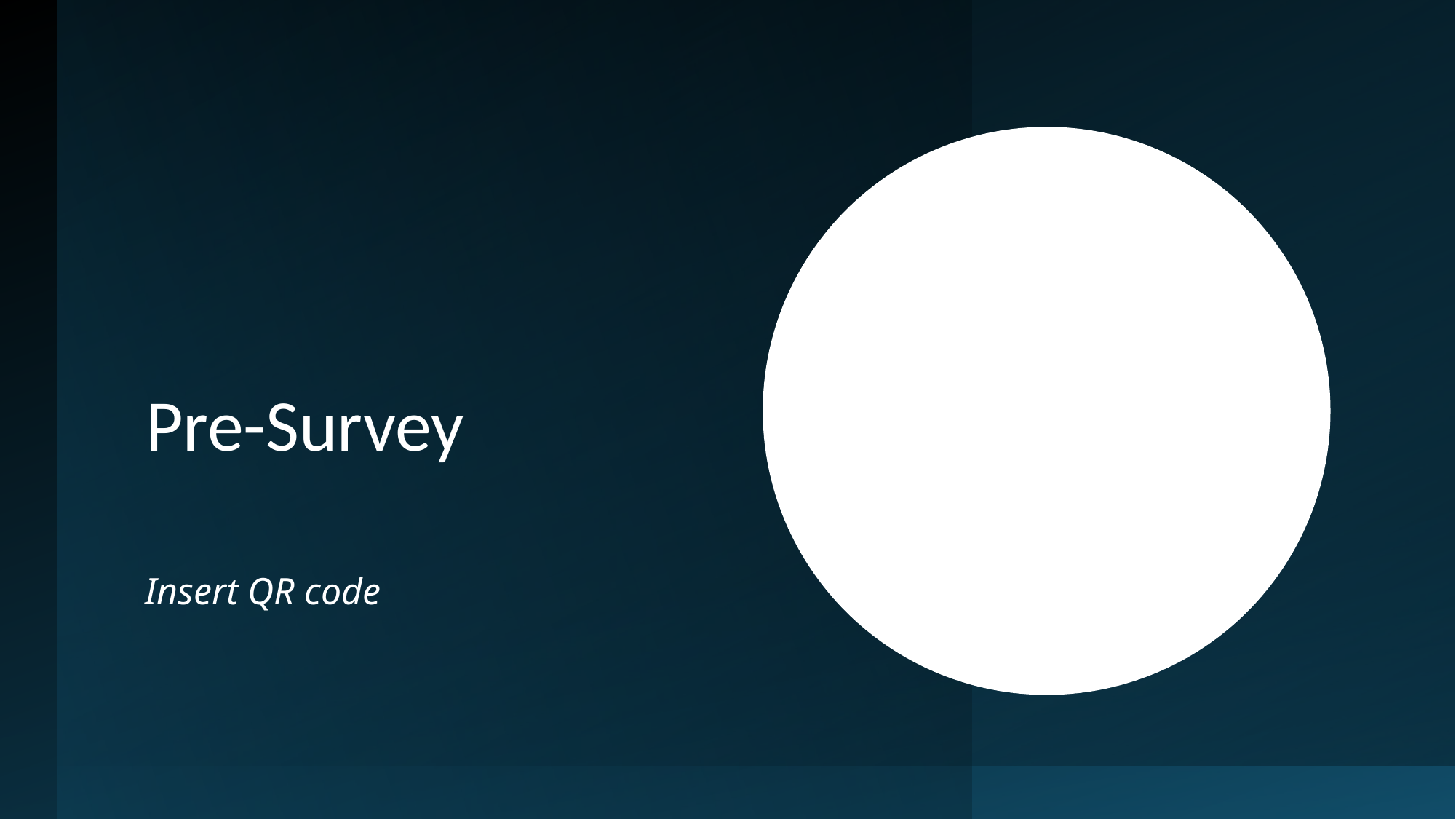

# Pre-Survey
Insert QR code

## Slide 3
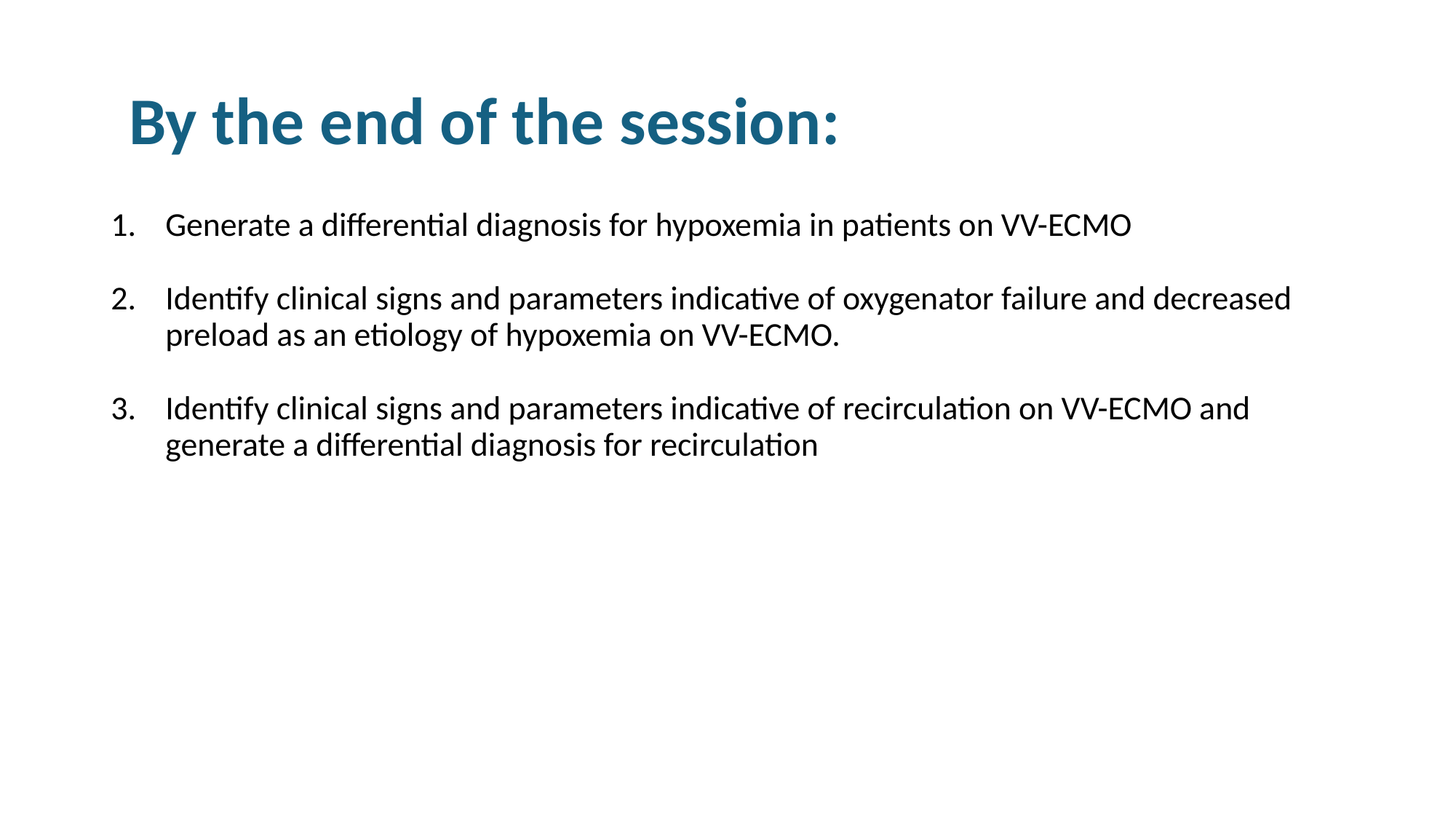

By the end of the session:
Generate a differential diagnosis for hypoxemia in patients on VV-ECMO
Identify clinical signs and parameters indicative of oxygenator failure and decreased preload as an etiology of hypoxemia on VV-ECMO.
Identify clinical signs and parameters indicative of recirculation on VV-ECMO and generate a differential diagnosis for recirculation

## Slide 4
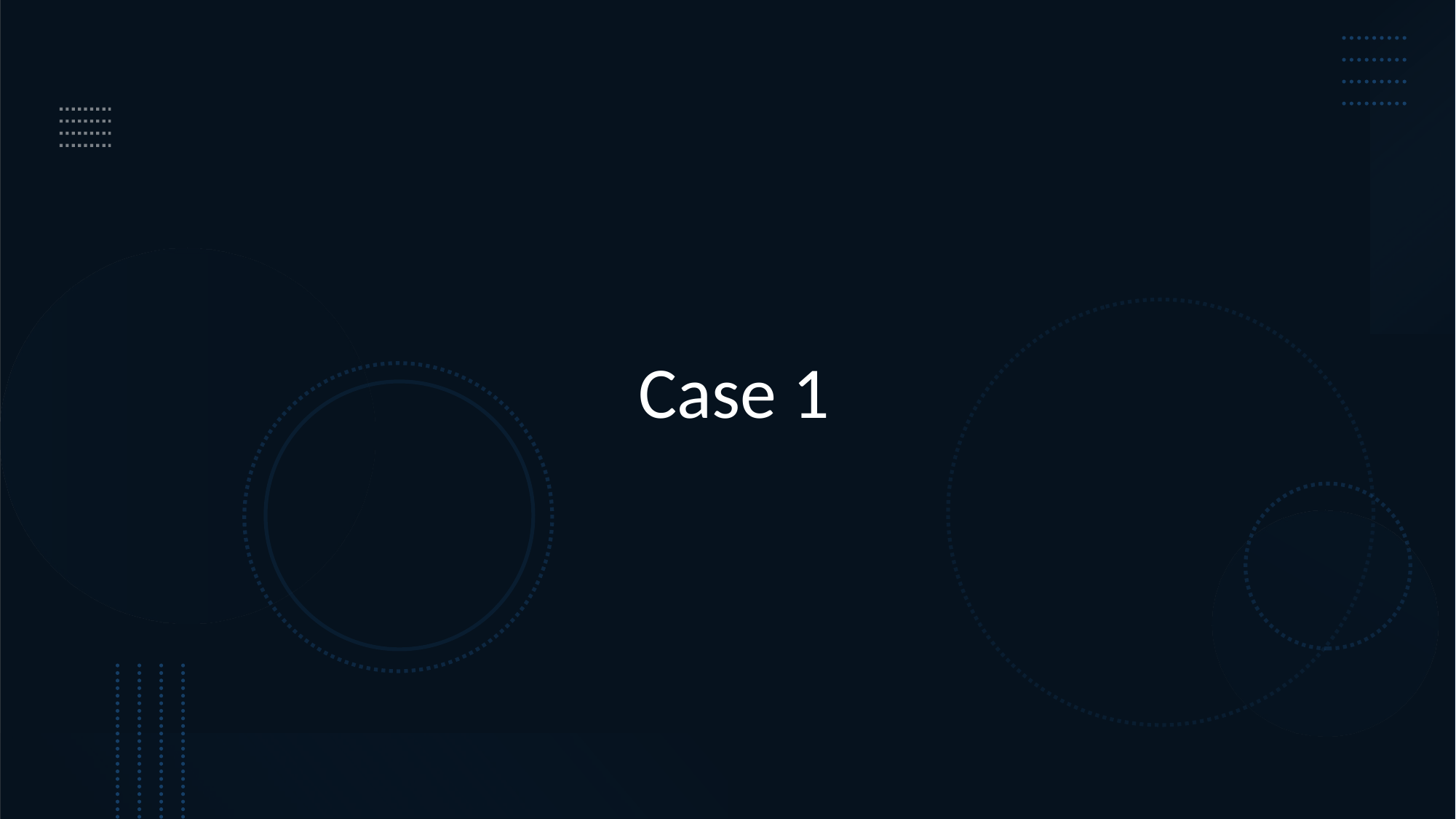

# Case 1

## Slide 5
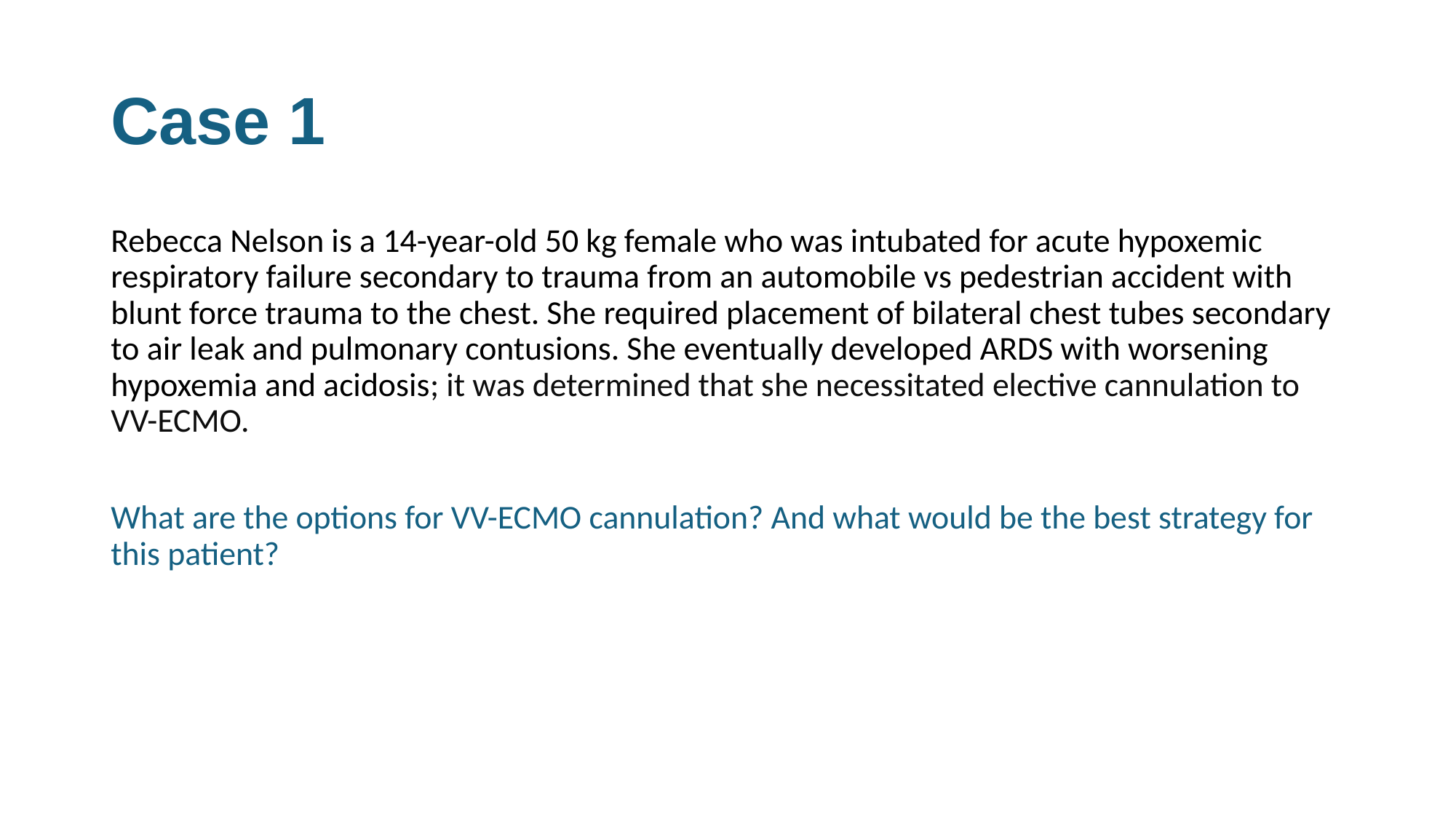

# Case 1
Rebecca Nelson is a 14-year-old 50 kg female who was intubated for acute hypoxemic respiratory failure secondary to trauma from an automobile vs pedestrian accident with blunt force trauma to the chest. She required placement of bilateral chest tubes secondary to air leak and pulmonary contusions. She eventually developed ARDS with worsening hypoxemia and acidosis; it was determined that she necessitated elective cannulation to VV-ECMO.
What are the options for VV-ECMO cannulation? And what would be the best strategy for this patient?

## Slide 6
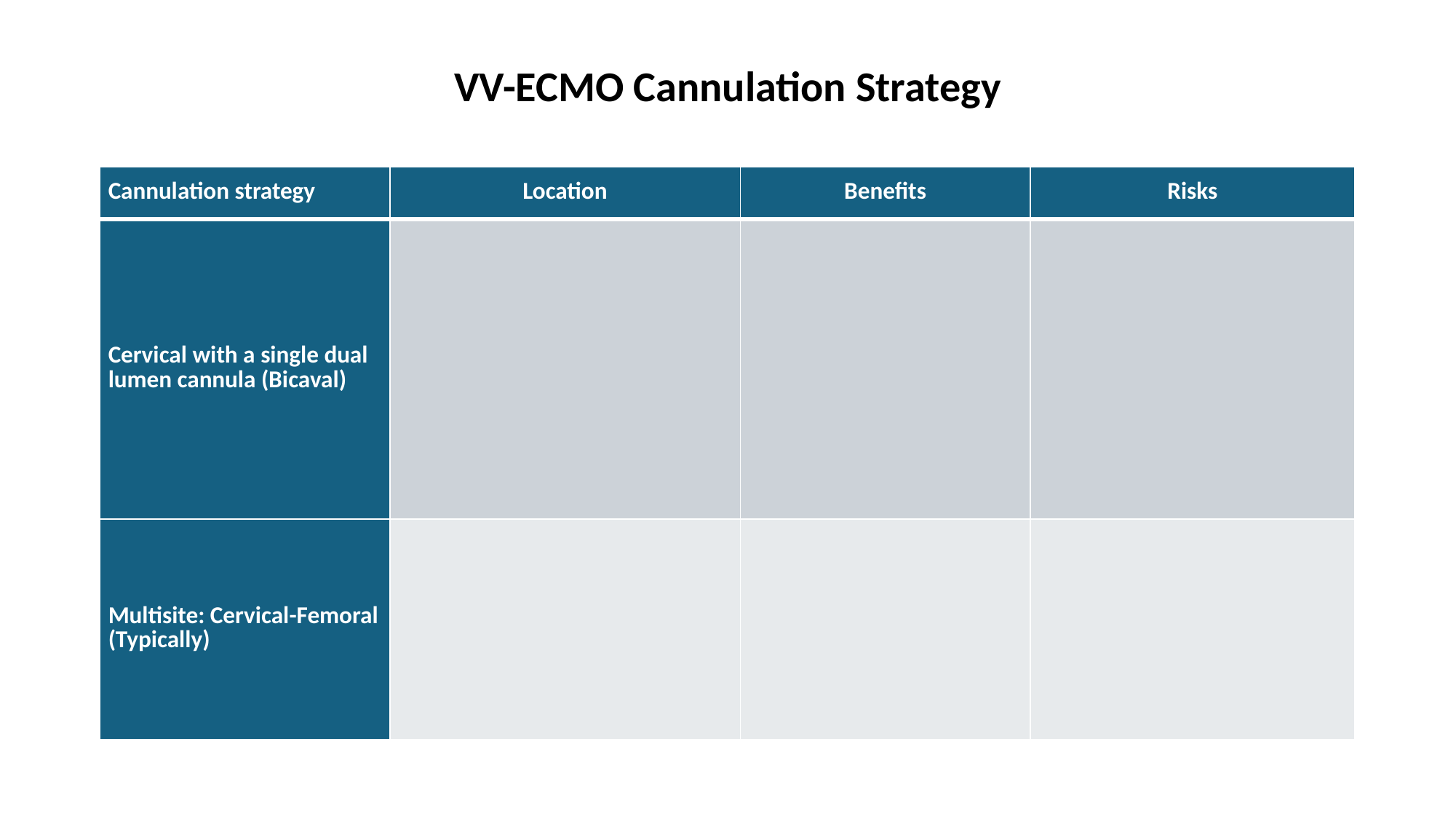

# VV-ECMO Cannulation Strategy
| Cannulation strategy | Location | Benefits | Risks |
| --- | --- | --- | --- |
| Cervical with a single dual lumen cannula (Bicaval) | | | |
| Multisite: Cervical-Femoral (Typically) | | | |

## Slide 7
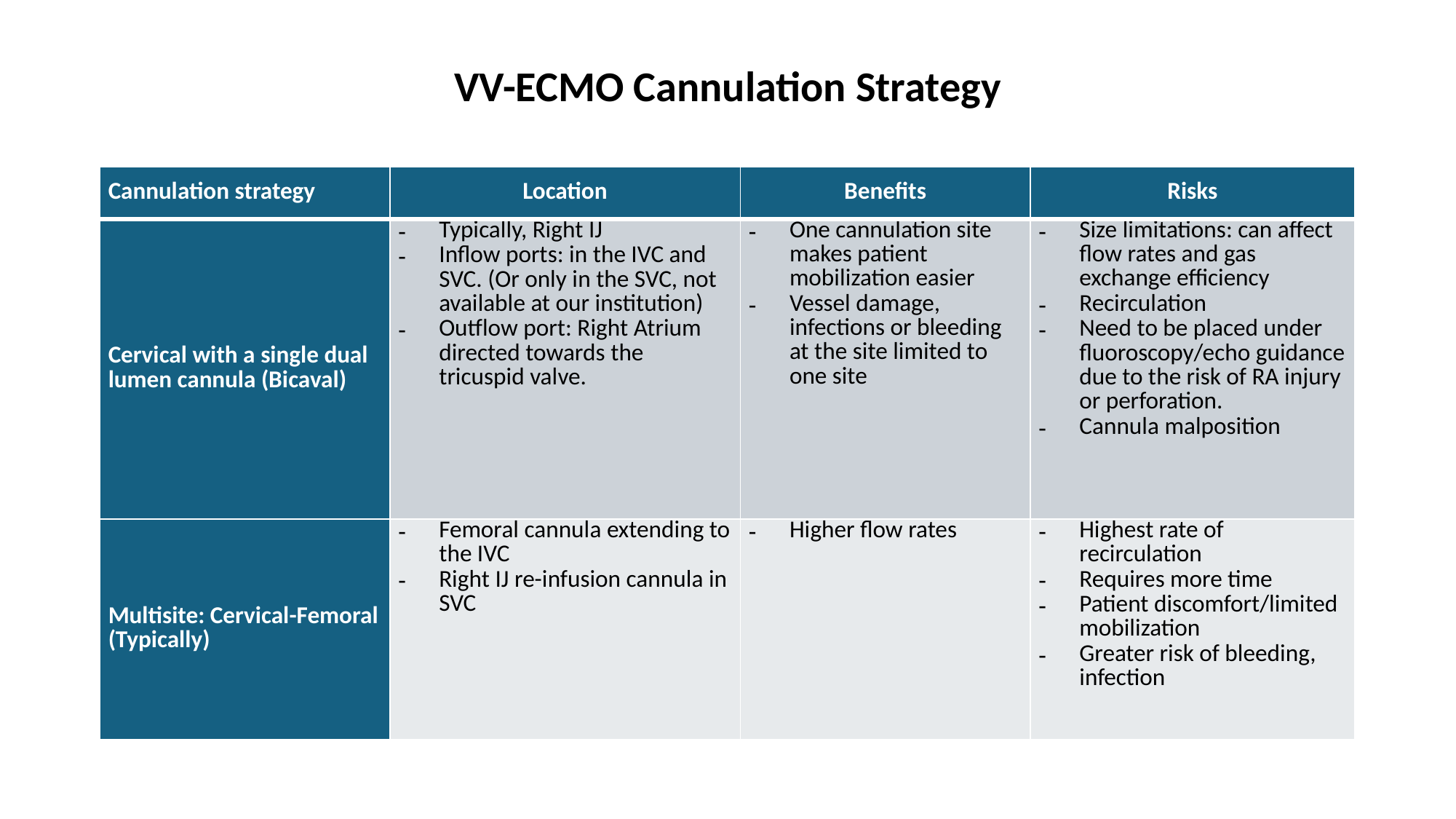

# VV-ECMO Cannulation Strategy
| Cannulation strategy | Location | Benefits | Risks |
| --- | --- | --- | --- |
| Cervical with a single dual lumen cannula (Bicaval) | Typically, Right IJ Inflow ports: in the IVC and SVC. (Or only in the SVC, not available at our institution) Outflow port: Right Atrium directed towards the tricuspid valve. | One cannulation site makes patient mobilization easier Vessel damage, infections or bleeding at the site limited to one site | Size limitations: can affect flow rates and gas exchange efficiency Recirculation Need to be placed under fluoroscopy/echo guidance due to the risk of RA injury or perforation. Cannula malposition |
| Multisite: Cervical-Femoral (Typically) | Femoral cannula extending to the IVC Right IJ re-infusion cannula in SVC | Higher flow rates | Highest rate of recirculation Requires more time Patient discomfort/limited mobilization Greater risk of bleeding, infection |

## Slide 8
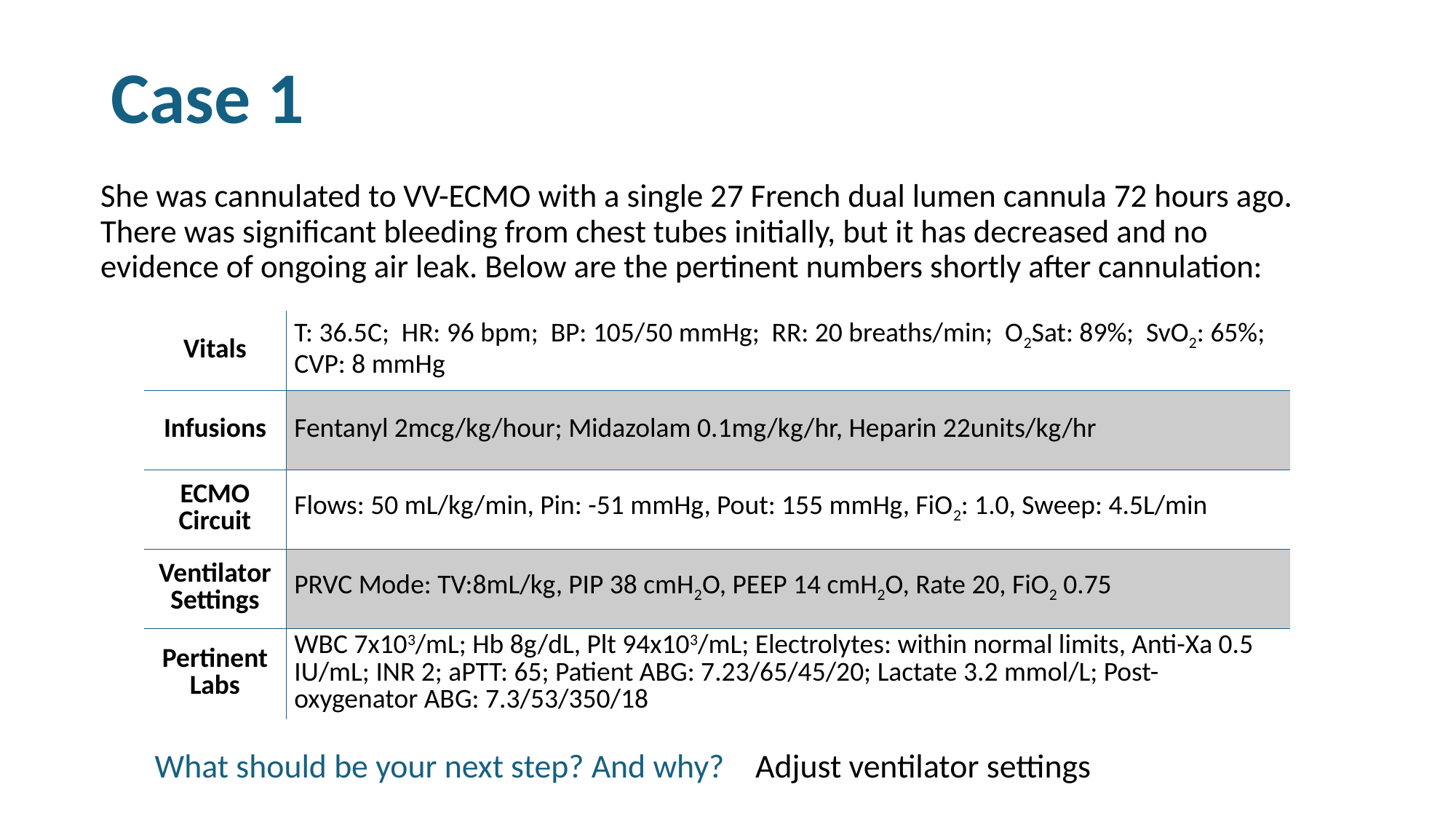

# Case 1
She was cannulated to VV-ECMO with a single 27 French dual lumen cannula 72 hours ago. There was significant bleeding from chest tubes initially, but it has decreased and no evidence of ongoing air leak. Below are the pertinent numbers shortly after cannulation:
| Vitals | T: 36.5C; HR: 96 bpm; BP: 105/50 mmHg; RR: 20 breaths/min; O2Sat: 89%; SvO2: 65%; CVP: 8 mmHg |
| --- | --- |
| Infusions | Fentanyl 2mcg/kg/hour; Midazolam 0.1mg/kg/hr, Heparin 22units/kg/hr |
| ECMO Circuit | Flows: 50 mL/kg/min, Pin: -51 mmHg, Pout: 155 mmHg, FiO2: 1.0, Sweep: 4.5L/min |
| Ventilator Settings | PRVC Mode: TV:8mL/kg, PIP 38 cmH2O, PEEP 14 cmH2O, Rate 20, FiO2 0.75 |
| Pertinent Labs | WBC 7x10^3^/mL; Hb 8g/dL, Plt 94x103/mL; Electrolytes: within normal limits, Anti-Xa 0.5 IU/mL; INR 2; aPTT: 65; Patient ABG: 7.23/65/45/20; Lactate 3.2 mmol/L; Post-oxygenator ABG: 7.3/53/350/18 |
What should be your next step? And why?
Adjust ventilator settings

## Slide 9
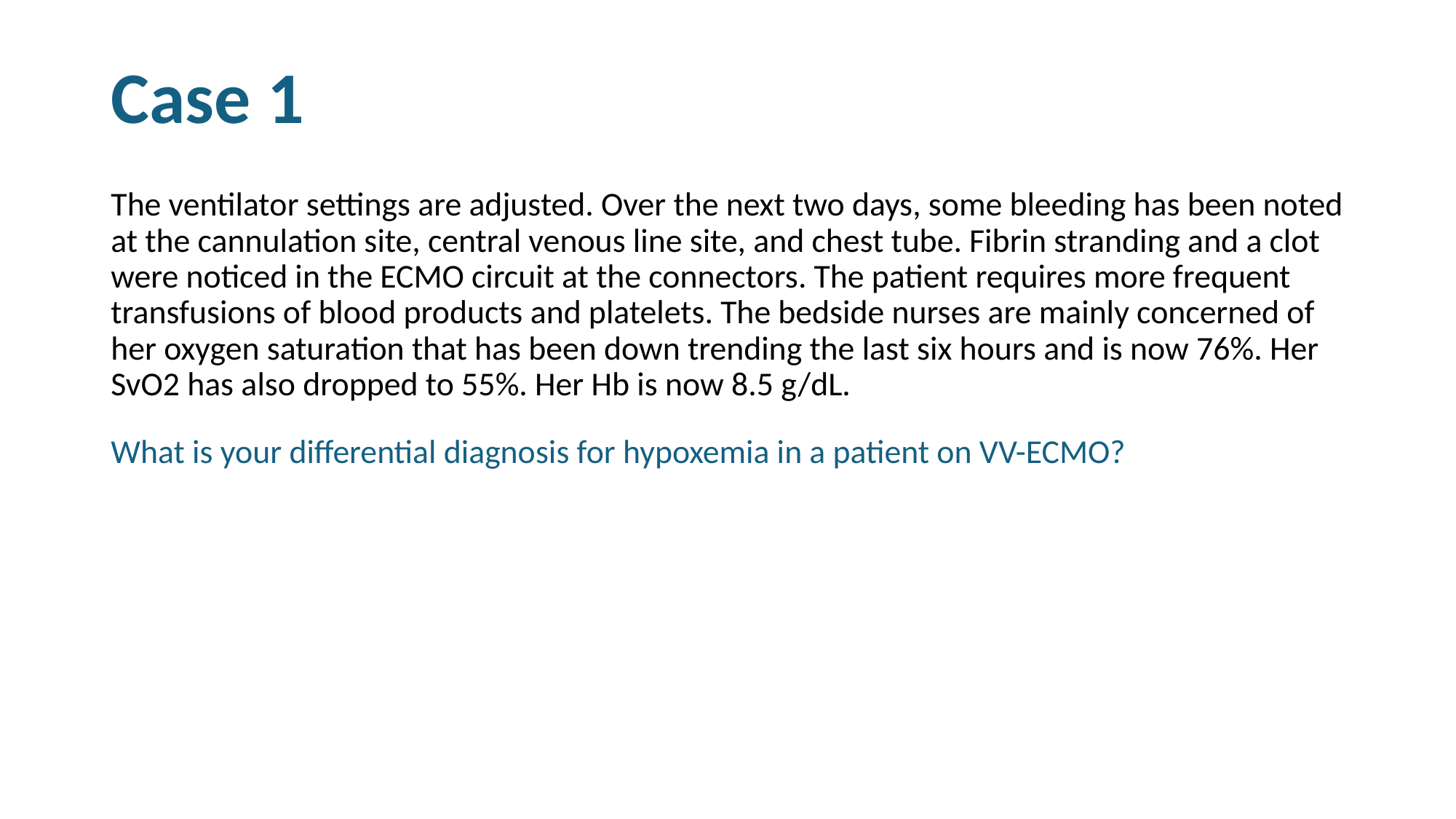

Case 1
The ventilator settings are adjusted. Over the next two days, some bleeding has been noted at the cannulation site, central venous line site, and chest tube. Fibrin stranding and a clot were noticed in the ECMO circuit at the connectors. The patient requires more frequent transfusions of blood products and platelets. The bedside nurses are mainly concerned of her oxygen saturation that has been down trending the last six hours and is now 76%. Her SvO2 has also dropped to 55%. Her Hb is now 8.5 g/dL.
What is your differential diagnosis for hypoxemia in a patient on VV-ECMO?

## Slide 10
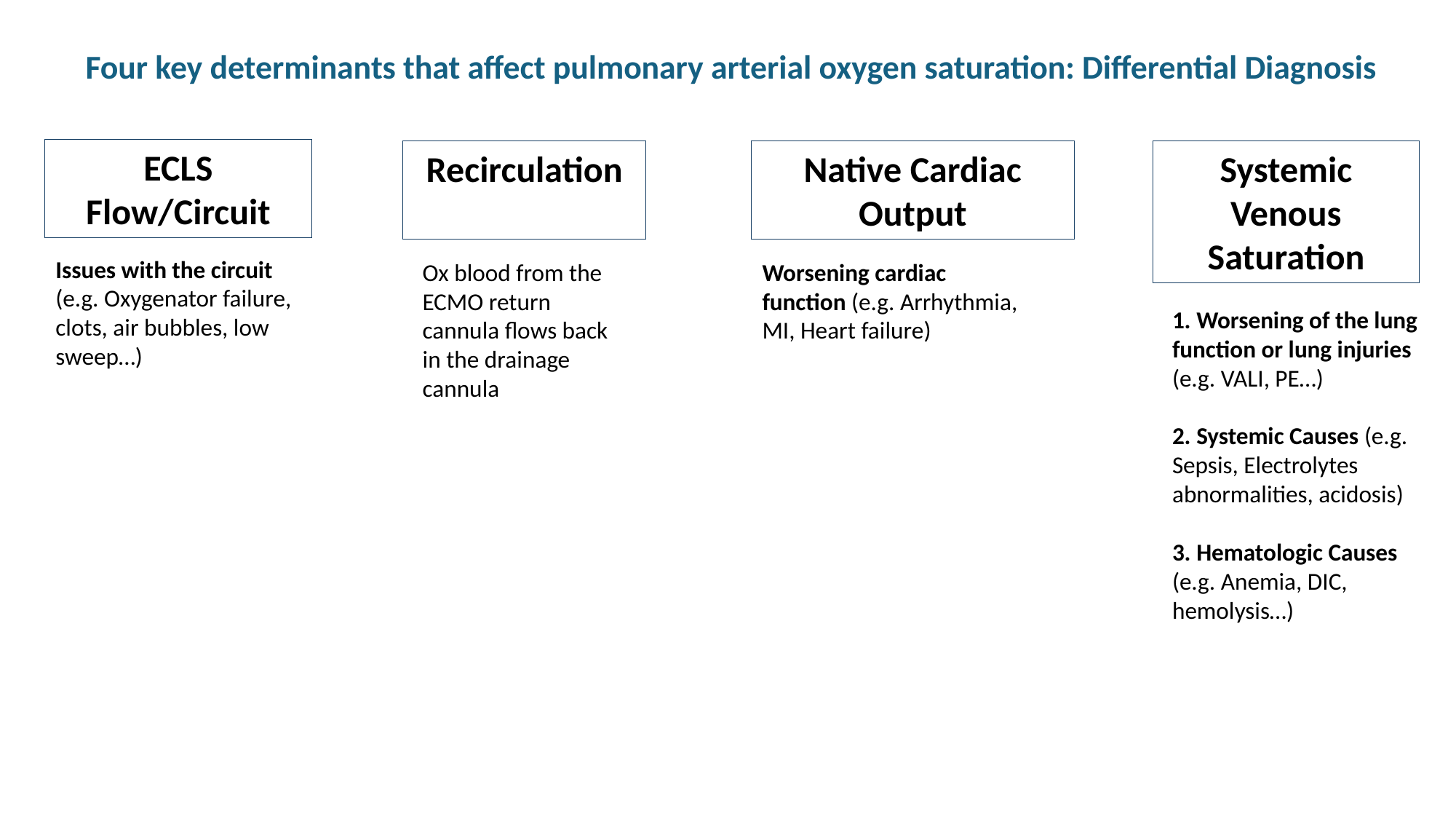

Four key determinants that affect pulmonary arterial oxygen saturation: Differential Diagnosis
ECLS Flow/Circuit
Recirculation
Native Cardiac Output
Systemic Venous Saturation
Issues with the circuit (e.g. Oxygenator failure, clots, air bubbles, low sweep…)
Ox blood from the ECMO return cannula flows back in the drainage cannula
Worsening cardiac function (e.g. Arrhythmia, MI, Heart failure)
1. Worsening of the lung function or lung injuries (e.g. VALI, PE…)
2. Systemic Causes (e.g. Sepsis, Electrolytes abnormalities, acidosis)
3. Hematologic Causes (e.g. Anemia, DIC, hemolysis…)

## Slide 11
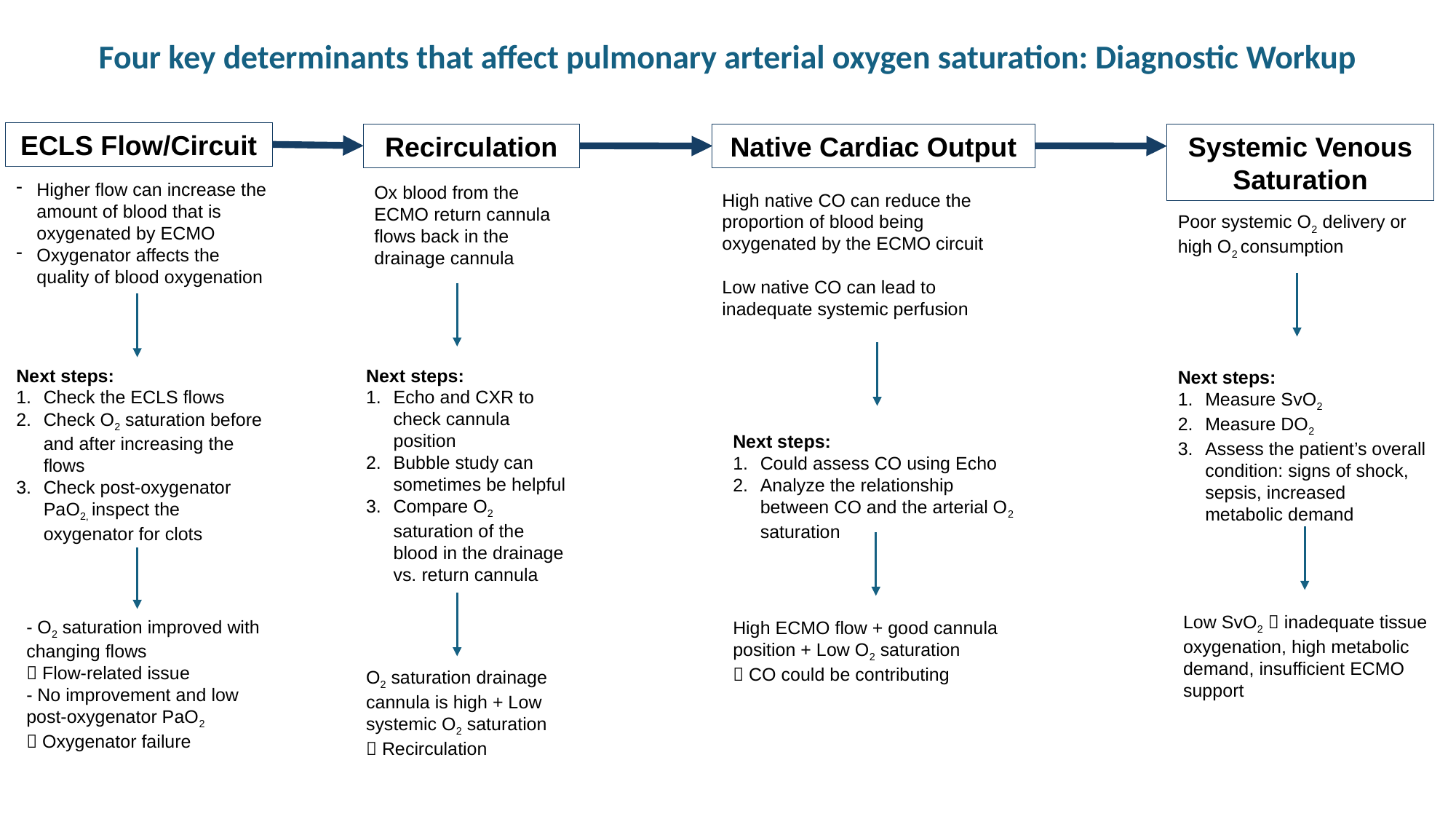

Four key determinants that affect pulmonary arterial oxygen saturation: Diagnostic Workup
ECLS Flow/Circuit
Recirculation
Native Cardiac Output
Systemic Venous Saturation
Higher flow can increase the amount of blood that is oxygenated by ECMO
Oxygenator affects the quality of blood oxygenation
Ox blood from the ECMO return cannula flows back in the drainage cannula
High native CO can reduce the proportion of blood being oxygenated by the ECMO circuit
Low native CO can lead to inadequate systemic perfusion
Poor systemic O2 delivery or high O2 consumption
Next steps:
Check the ECLS flows
Check O2 saturation before and after increasing the flows
Check post-oxygenator PaO2, inspect the oxygenator for clots
Next steps:
Echo and CXR to check cannula position
Bubble study can sometimes be helpful
Compare O2 saturation of the blood in the drainage vs. return cannula
Next steps:
Measure SvO2
Measure DO2
Assess the patient’s overall condition: signs of shock, sepsis, increased metabolic demand
Next steps:
Could assess CO using Echo
Analyze the relationship between CO and the arterial O2 saturation
Low SvO2  inadequate tissue oxygenation, high metabolic demand, insufficient ECMO support
- O2 saturation improved with changing flows
 Flow-related issue
- No improvement and low post-oxygenator PaO2
 Oxygenator failure
High ECMO flow + good cannula position + Low O2 saturation
 CO could be contributing
O2 saturation drainage cannula is high + Low systemic O2 saturation
 Recirculation

## Slide 12
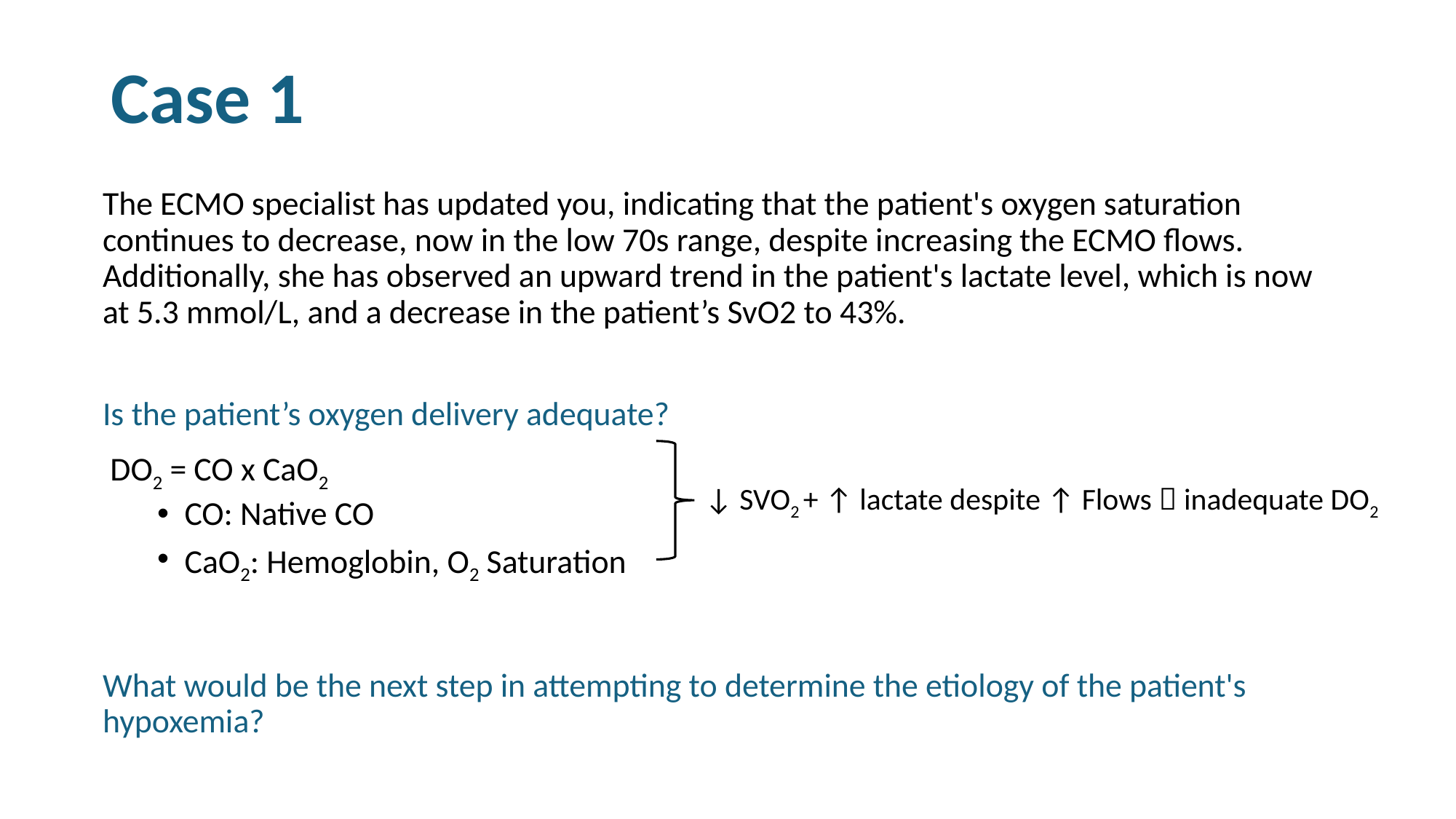

# Case 1
The ECMO specialist has updated you, indicating that the patient's oxygen saturation continues to decrease, now in the low 70s range, despite increasing the ECMO flows. Additionally, she has observed an upward trend in the patient's lactate level, which is now at 5.3 mmol/L, and a decrease in the patient’s SvO2 to 43%.
Is the patient’s oxygen delivery adequate?
 DO2 = CO x CaO2
CO: Native CO
CaO2: Hemoglobin, O2 Saturation
What would be the next step in attempting to determine the etiology of the patient's hypoxemia?
↓ SVO2 + ↑ lactate despite ↑ Flows  inadequate DO2

## Slide 13
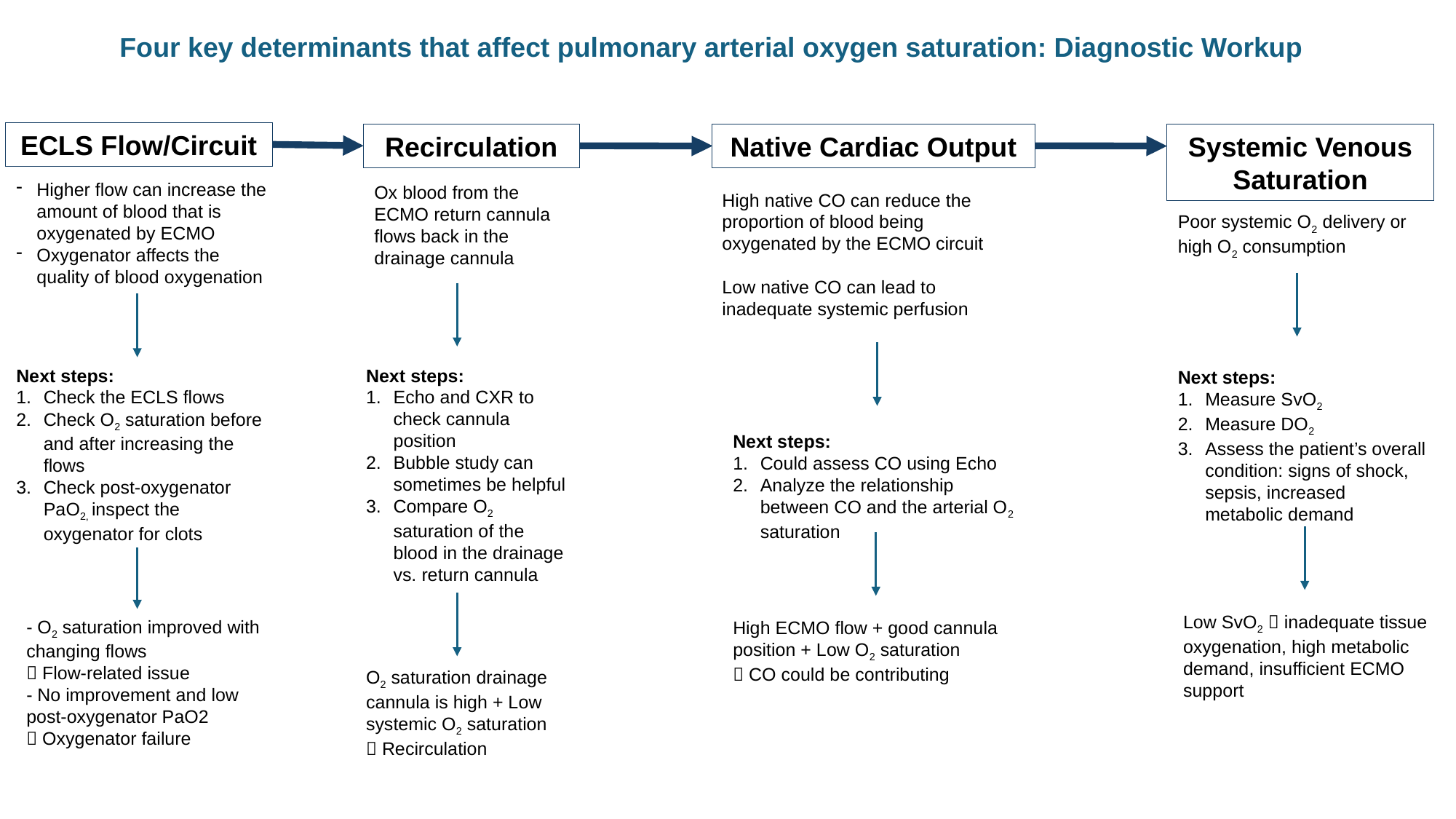

Four key determinants that affect pulmonary arterial oxygen saturation: Diagnostic Workup
ECLS Flow/Circuit
Recirculation
Native Cardiac Output
Systemic Venous Saturation
Higher flow can increase the amount of blood that is oxygenated by ECMO
Oxygenator affects the quality of blood oxygenation
Ox blood from the ECMO return cannula flows back in the drainage cannula
High native CO can reduce the proportion of blood being oxygenated by the ECMO circuit
Low native CO can lead to inadequate systemic perfusion
Poor systemic O2 delivery or high O2 consumption
Next steps:
Check the ECLS flows
Check O2 saturation before and after increasing the flows
Check post-oxygenator PaO2, inspect the oxygenator for clots
Next steps:
Echo and CXR to check cannula position
Bubble study can sometimes be helpful
Compare O2 saturation of the blood in the drainage vs. return cannula
Next steps:
Measure SvO2
Measure DO2
Assess the patient’s overall condition: signs of shock, sepsis, increased metabolic demand
Next steps:
Could assess CO using Echo
Analyze the relationship between CO and the arterial O2 saturation
Low SvO2  inadequate tissue oxygenation, high metabolic demand, insufficient ECMO support
- O2 saturation improved with changing flows
 Flow-related issue
- No improvement and low post-oxygenator PaO2
 Oxygenator failure
High ECMO flow + good cannula position + Low O2 saturation
 CO could be contributing
O2 saturation drainage cannula is high + Low systemic O2 saturation
 Recirculation

## Slide 14
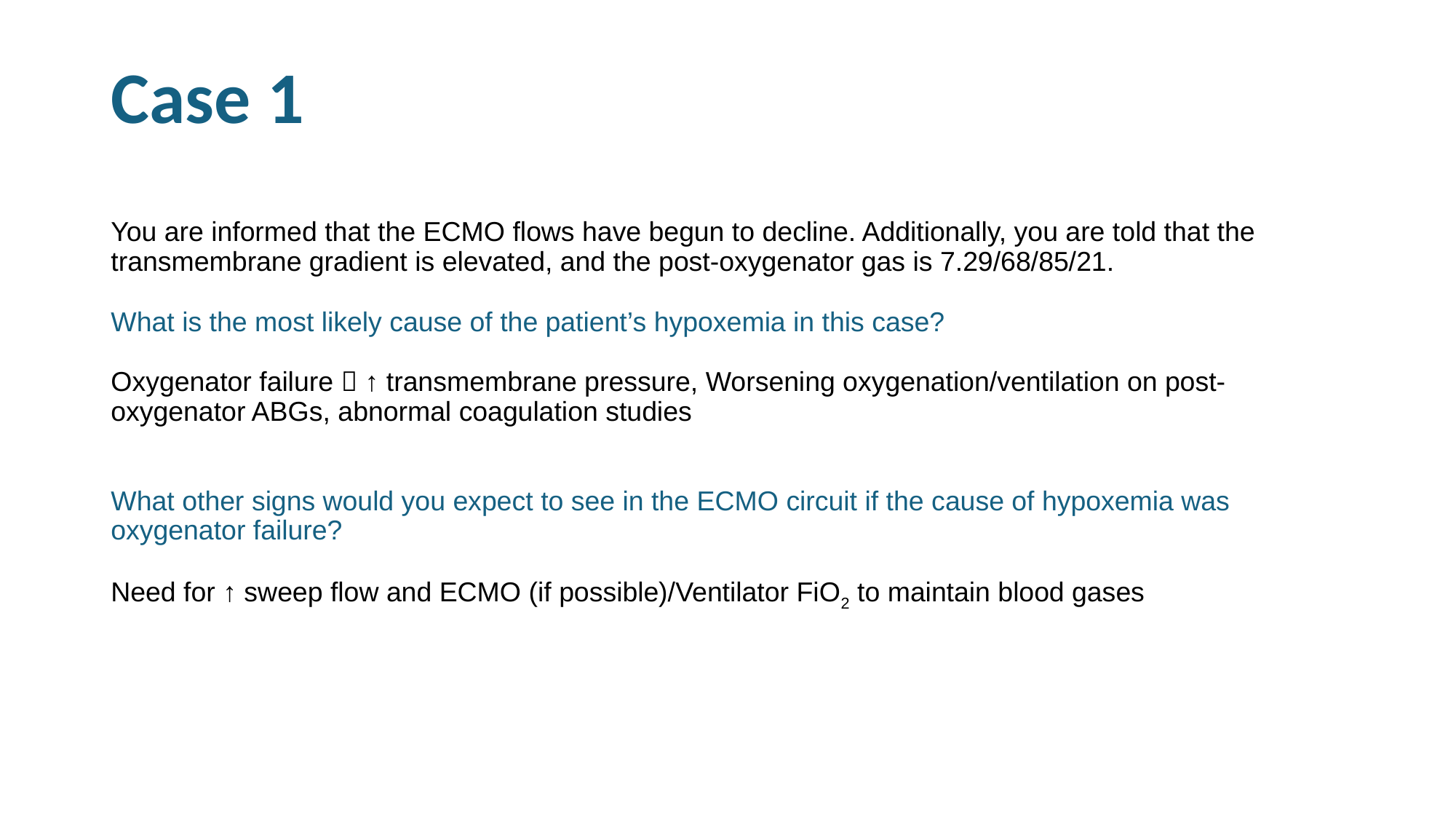

# Case 1
You are informed that the ECMO flows have begun to decline. Additionally, you are told that the transmembrane gradient is elevated, and the post-oxygenator gas is 7.29/68/85/21.
What is the most likely cause of the patient’s hypoxemia in this case?
Oxygenator failure  ↑ transmembrane pressure, Worsening oxygenation/ventilation on post-oxygenator ABGs, abnormal coagulation studies
What other signs would you expect to see in the ECMO circuit if the cause of hypoxemia was oxygenator failure?
Need for ↑ sweep flow and ECMO (if possible)/Ventilator FiO2 to maintain blood gases

## Slide 15
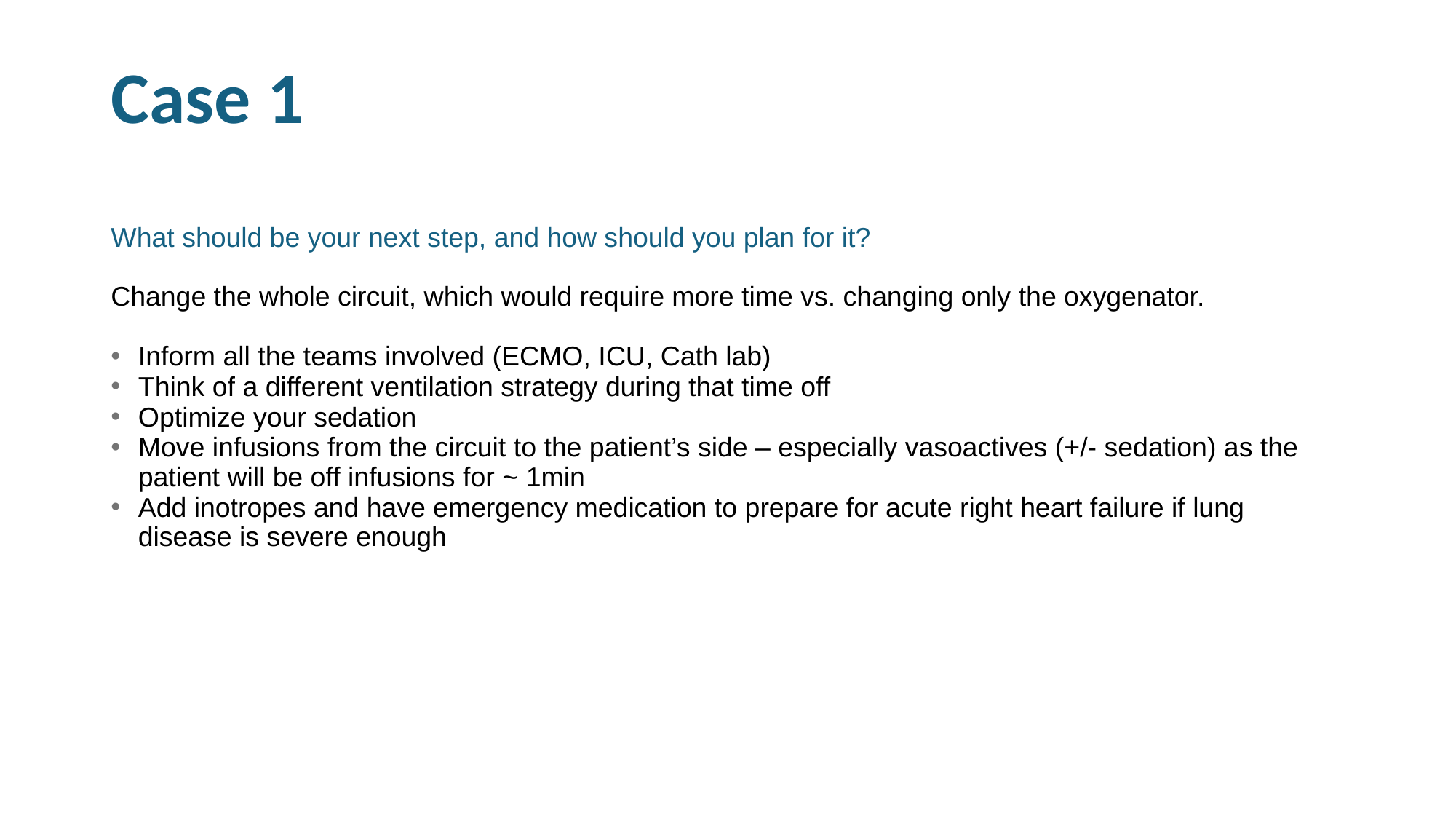

# Case 1
What should be your next step, and how should you plan for it?
Change the whole circuit, which would require more time vs. changing only the oxygenator.
Inform all the teams involved (ECMO, ICU, Cath lab)
Think of a different ventilation strategy during that time off
Optimize your sedation
Move infusions from the circuit to the patient’s side – especially vasoactives (+/- sedation) as the patient will be off infusions for ~ 1min
Add inotropes and have emergency medication to prepare for acute right heart failure if lung disease is severe enough

## Slide 16
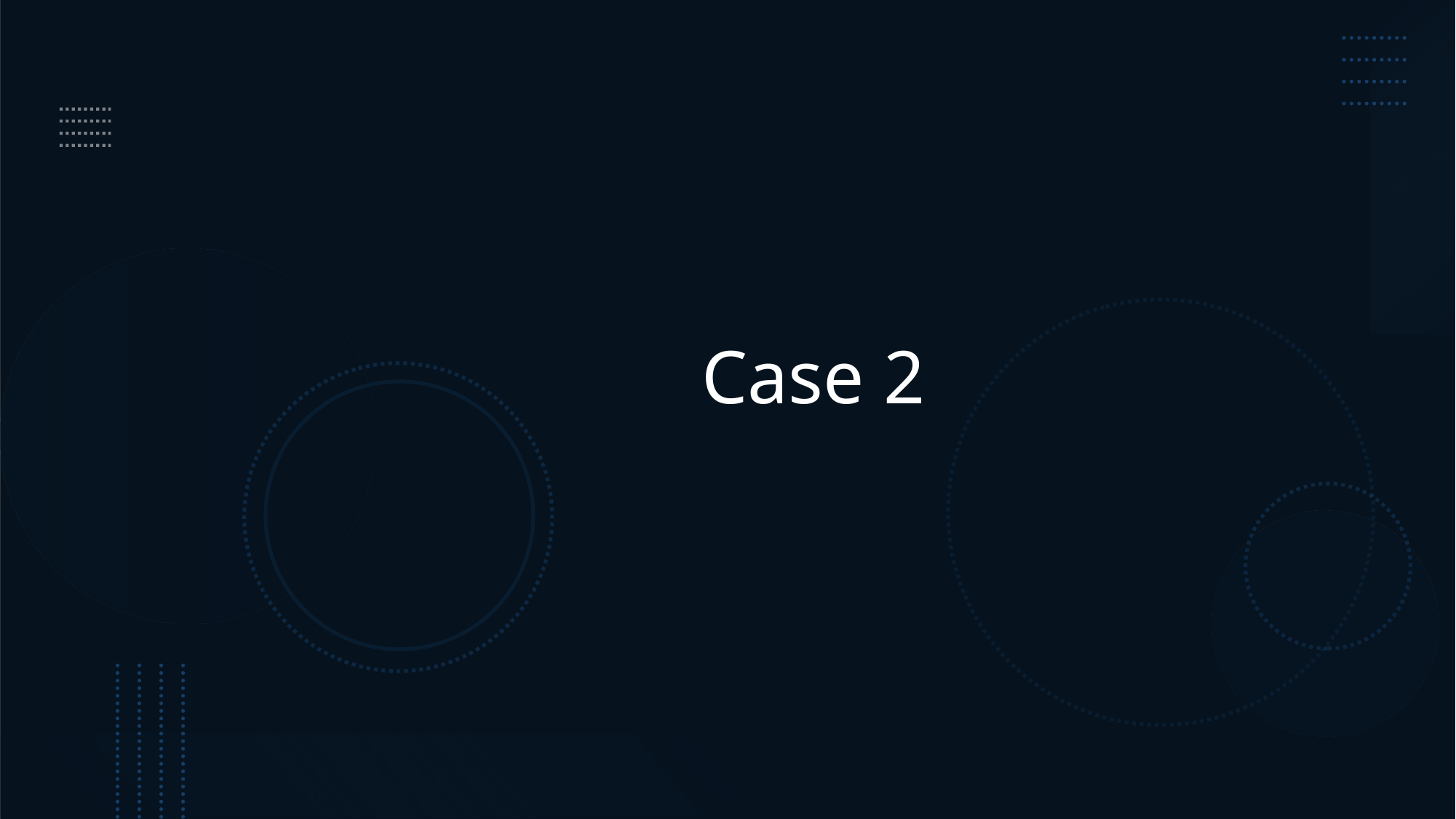

# Case 2

## Slide 17
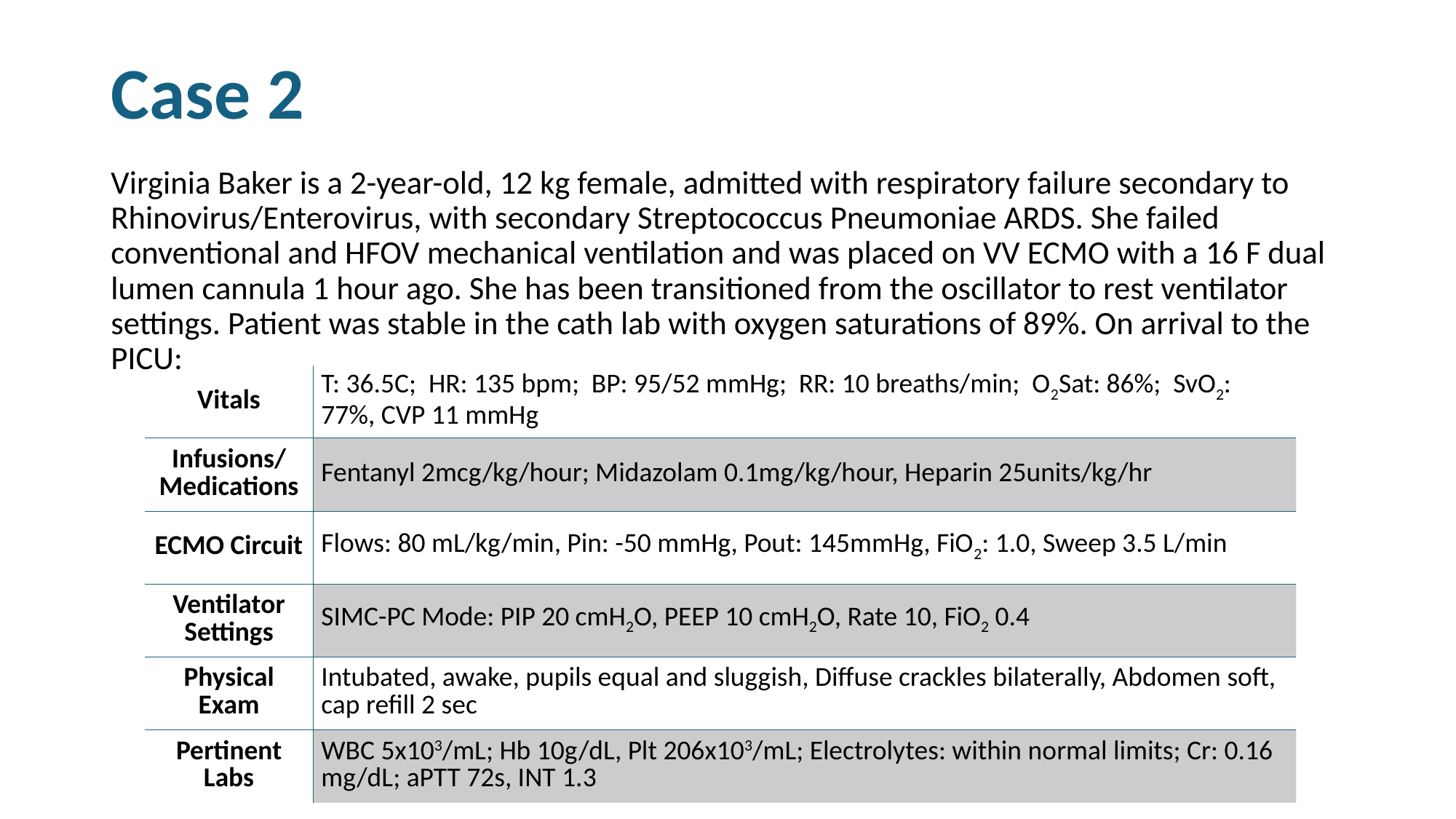

# Case 2
Virginia Baker is a 2-year-old, 12 kg female, admitted with respiratory failure secondary to Rhinovirus/Enterovirus, with secondary Streptococcus Pneumoniae ARDS. She failed conventional and HFOV mechanical ventilation and was placed on VV ECMO with a 16 F dual lumen cannula 1 hour ago. She has been transitioned from the oscillator to rest ventilator settings. Patient was stable in the cath lab with oxygen saturations of 89%. On arrival to the PICU:
| Vitals | T: 36.5C; HR: 135 bpm; BP: 95/52 mmHg; RR: 10 breaths/min; O2Sat: 86%; SvO2: 77%, CVP 11 mmHg |
| --- | --- |
| Infusions/ Medications | Fentanyl 2mcg/kg/hour; Midazolam 0.1mg/kg/hour, Heparin 25units/kg/hr |
| ECMO Circuit | Flows: 80 mL/kg/min, Pin: -50 mmHg, Pout: 145mmHg, FiO2: 1.0, Sweep 3.5 L/min |
| Ventilator Settings | SIMC-PC Mode: PIP 20 cmH2O, PEEP 10 cmH2O, Rate 10, FiO2 0.4 |
| Physical Exam | Intubated, awake, pupils equal and sluggish, Diffuse crackles bilaterally, Abdomen soft, cap refill 2 sec |
| Pertinent Labs | WBC 5x10^3^/mL; Hb 10g/dL, Plt 206x103/mL; Electrolytes: within normal limits; Cr: 0.16 mg/dL; aPTT 72s, INT 1.3 |

## Slide 18
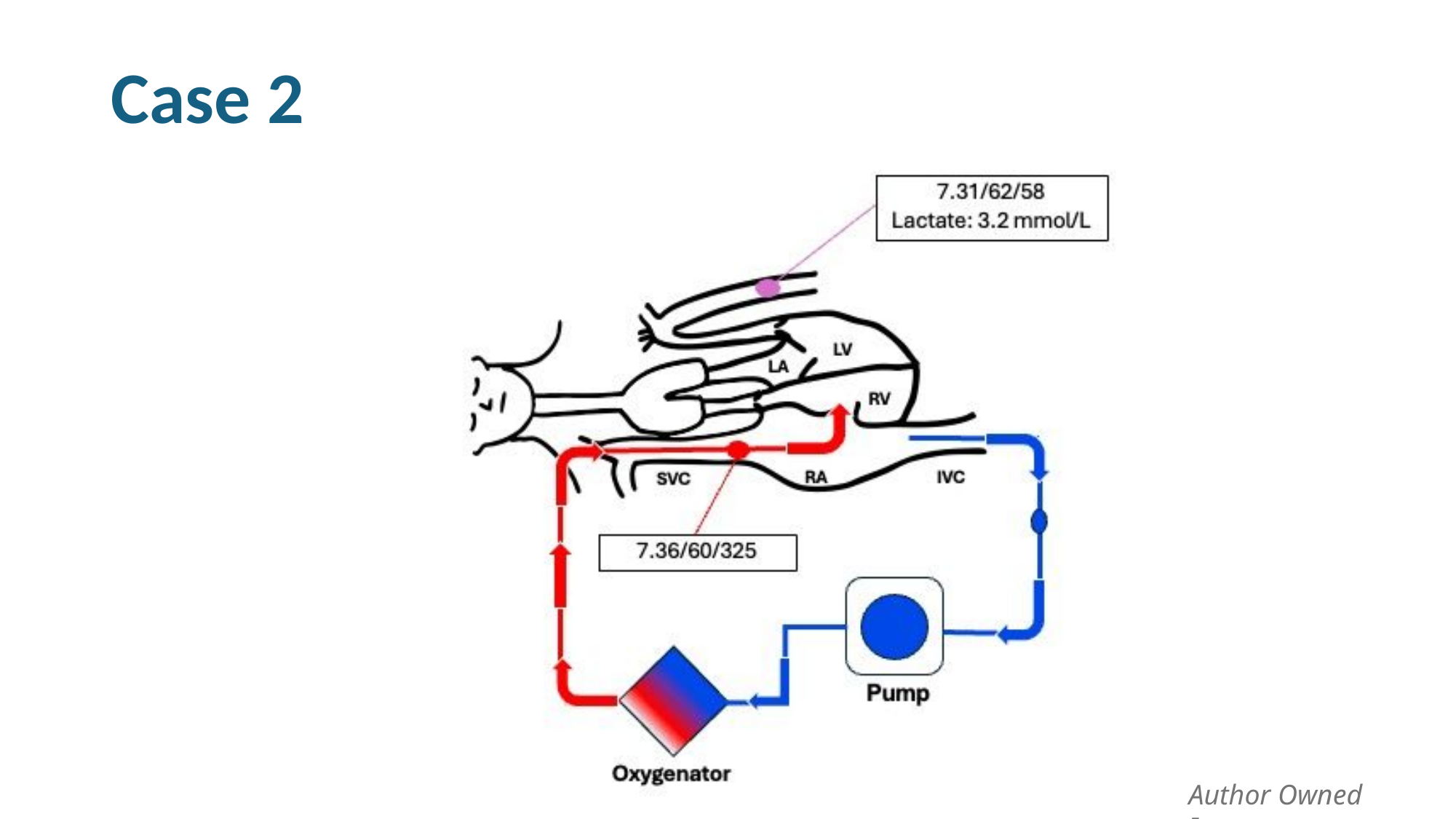

# Case 2
Author Owned Image

## Slide 19
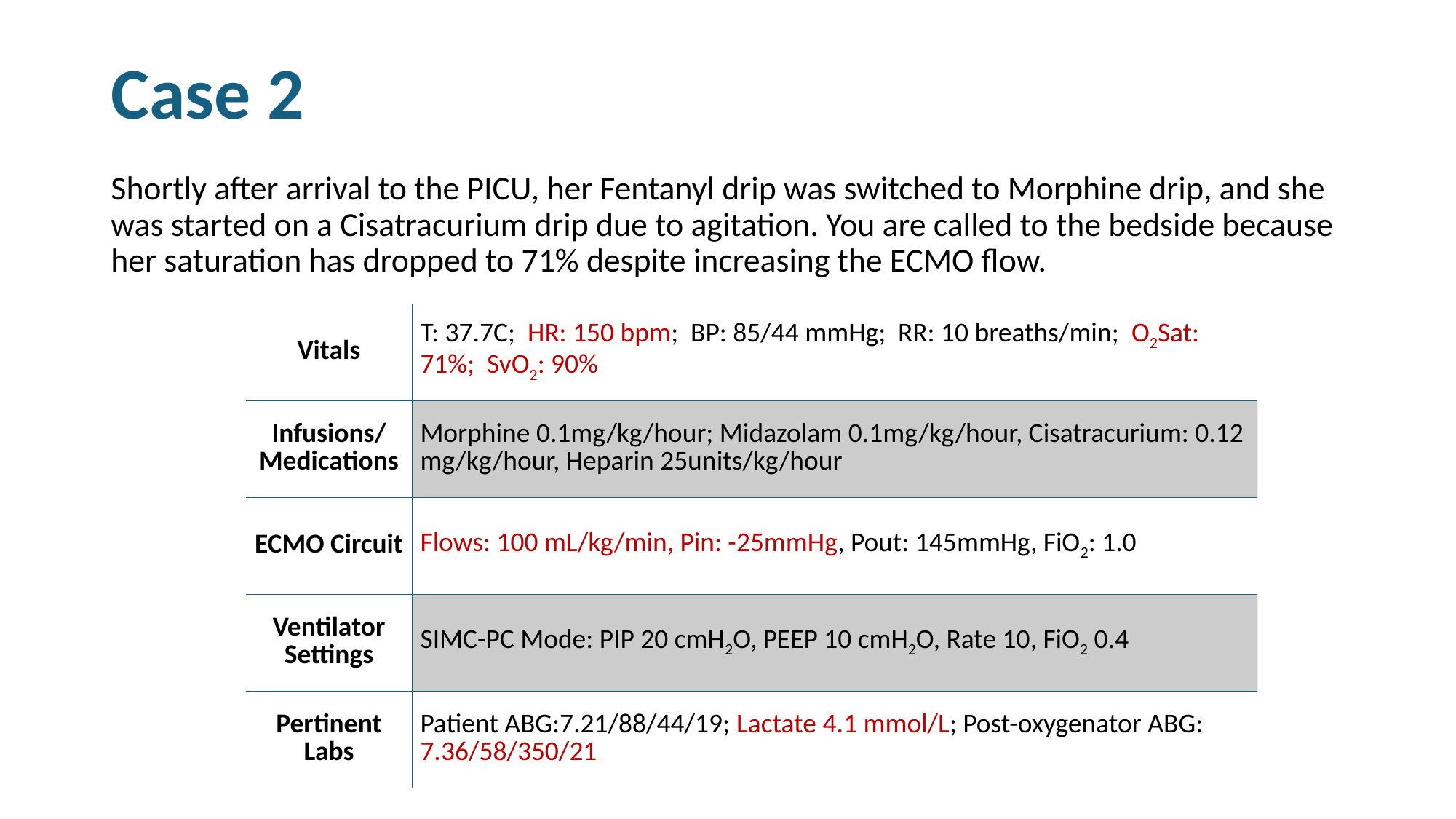

# Case 2
Shortly after arrival to the PICU, her Fentanyl drip was switched to Morphine drip, and she was started on a Cisatracurium drip due to agitation. You are called to the bedside because her saturation has dropped to 71% despite increasing the ECMO flow.
| Vitals | T: 37.7C; HR: 150 bpm; BP: 85/44 mmHg; RR: 10 breaths/min; O2Sat: 71%; SvO2: 90% |
| --- | --- |
| Infusions/ Medications | Morphine 0.1mg/kg/hour; Midazolam 0.1mg/kg/hour, Cisatracurium: 0.12 mg/kg/hour, Heparin 25units/kg/hour |
| ECMO Circuit | Flows: 100 mL/kg/min, Pin: -25mmHg, Pout: 145mmHg, FiO2: 1.0 |
| Ventilator Settings | SIMC-PC Mode: PIP 20 cmH2O, PEEP 10 cmH2O, Rate 10, FiO2 0.4 |
| Pertinent Labs | Patient ABG:7.21/88/44/19; Lactate 4.1 mmol/L; Post-oxygenator ABG: 7.36/58/350/21 |

## Slide 20
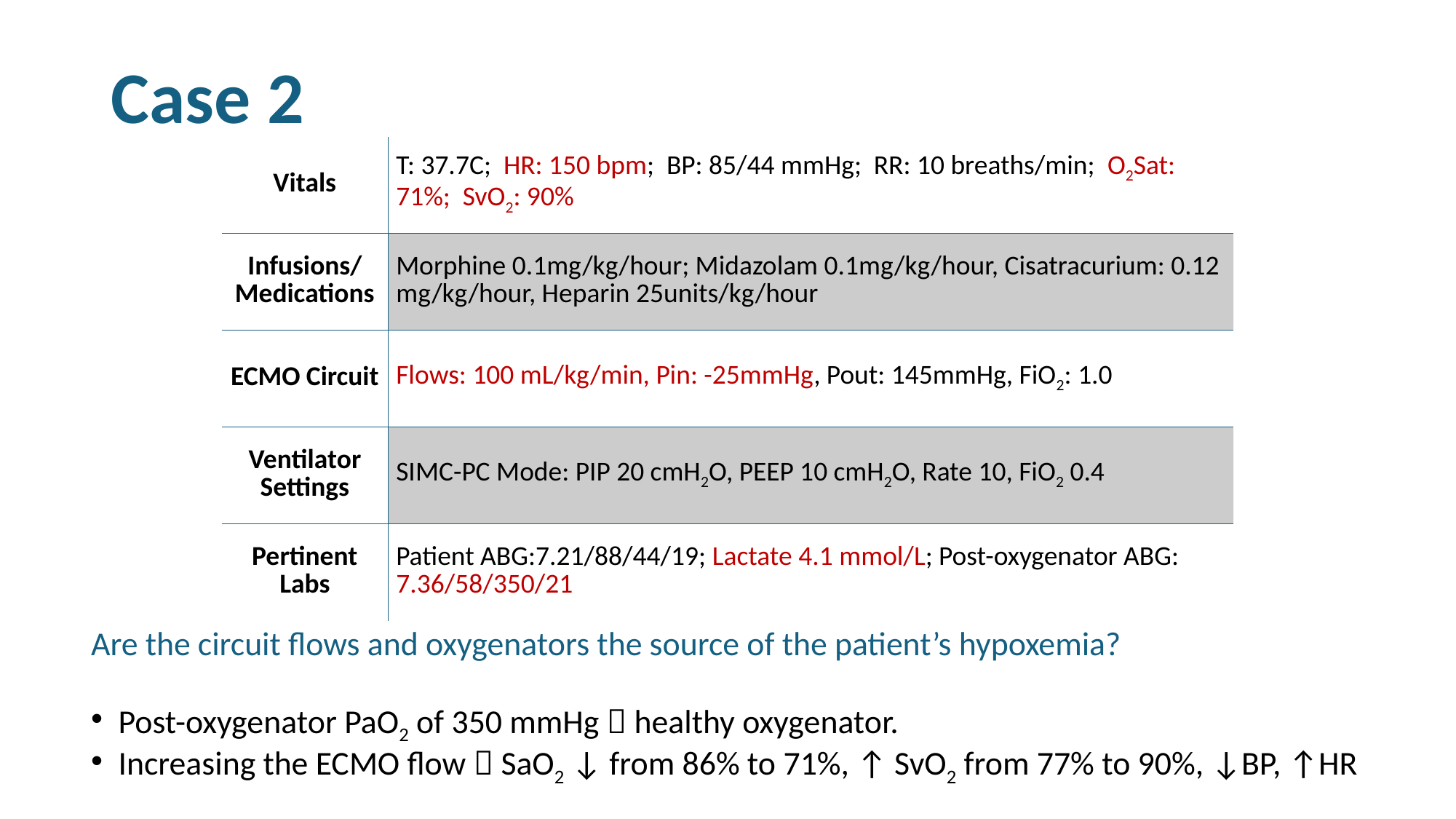

# Case 2
| Vitals | T: 37.7C; HR: 150 bpm; BP: 85/44 mmHg; RR: 10 breaths/min; O2Sat: 71%; SvO2: 90% |
| --- | --- |
| Infusions/ Medications | Morphine 0.1mg/kg/hour; Midazolam 0.1mg/kg/hour, Cisatracurium: 0.12 mg/kg/hour, Heparin 25units/kg/hour |
| ECMO Circuit | Flows: 100 mL/kg/min, Pin: -25mmHg, Pout: 145mmHg, FiO2: 1.0 |
| Ventilator Settings | SIMC-PC Mode: PIP 20 cmH2O, PEEP 10 cmH2O, Rate 10, FiO2 0.4 |
| Pertinent Labs | Patient ABG:7.21/88/44/19; Lactate 4.1 mmol/L; Post-oxygenator ABG: 7.36/58/350/21 |
Are the circuit flows and oxygenators the source of the patient’s hypoxemia?
Post-oxygenator PaO2 of 350 mmHg  healthy oxygenator.
Increasing the ECMO flow  SaO2 ↓ from 86% to 71%, ↑ SvO2 from 77% to 90%, ↓BP, ↑HR

## Slide 21
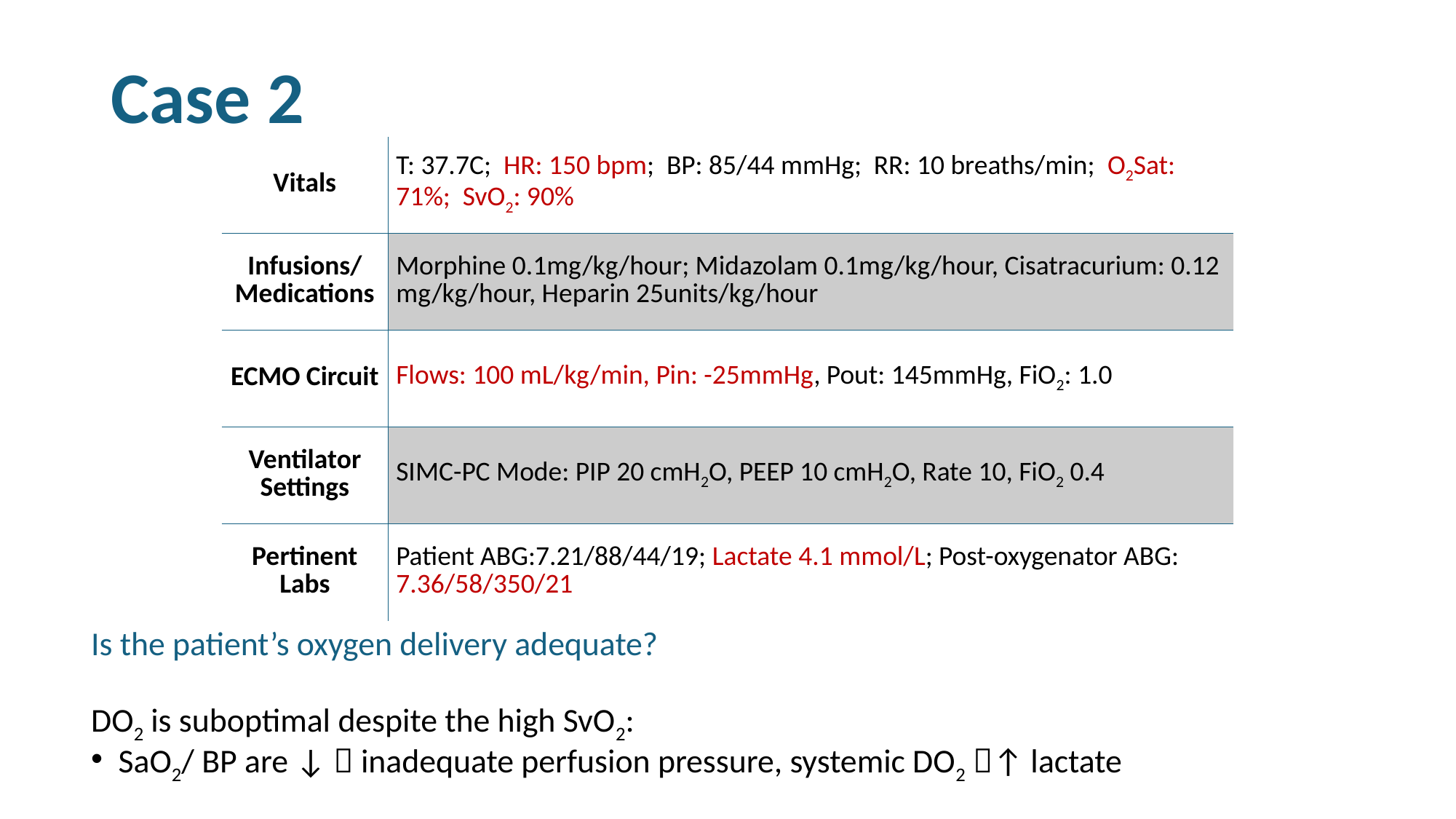

# Case 2
| Vitals | T: 37.7C; HR: 150 bpm; BP: 85/44 mmHg; RR: 10 breaths/min; O2Sat: 71%; SvO2: 90% |
| --- | --- |
| Infusions/ Medications | Morphine 0.1mg/kg/hour; Midazolam 0.1mg/kg/hour, Cisatracurium: 0.12 mg/kg/hour, Heparin 25units/kg/hour |
| ECMO Circuit | Flows: 100 mL/kg/min, Pin: -25mmHg, Pout: 145mmHg, FiO2: 1.0 |
| Ventilator Settings | SIMC-PC Mode: PIP 20 cmH2O, PEEP 10 cmH2O, Rate 10, FiO2 0.4 |
| Pertinent Labs | Patient ABG:7.21/88/44/19; Lactate 4.1 mmol/L; Post-oxygenator ABG: 7.36/58/350/21 |
Is the patient’s oxygen delivery adequate?
DO2 is suboptimal despite the high SvO2:
SaO2/ BP are ↓  inadequate perfusion pressure, systemic DO2 ↑ lactate

## Slide 22
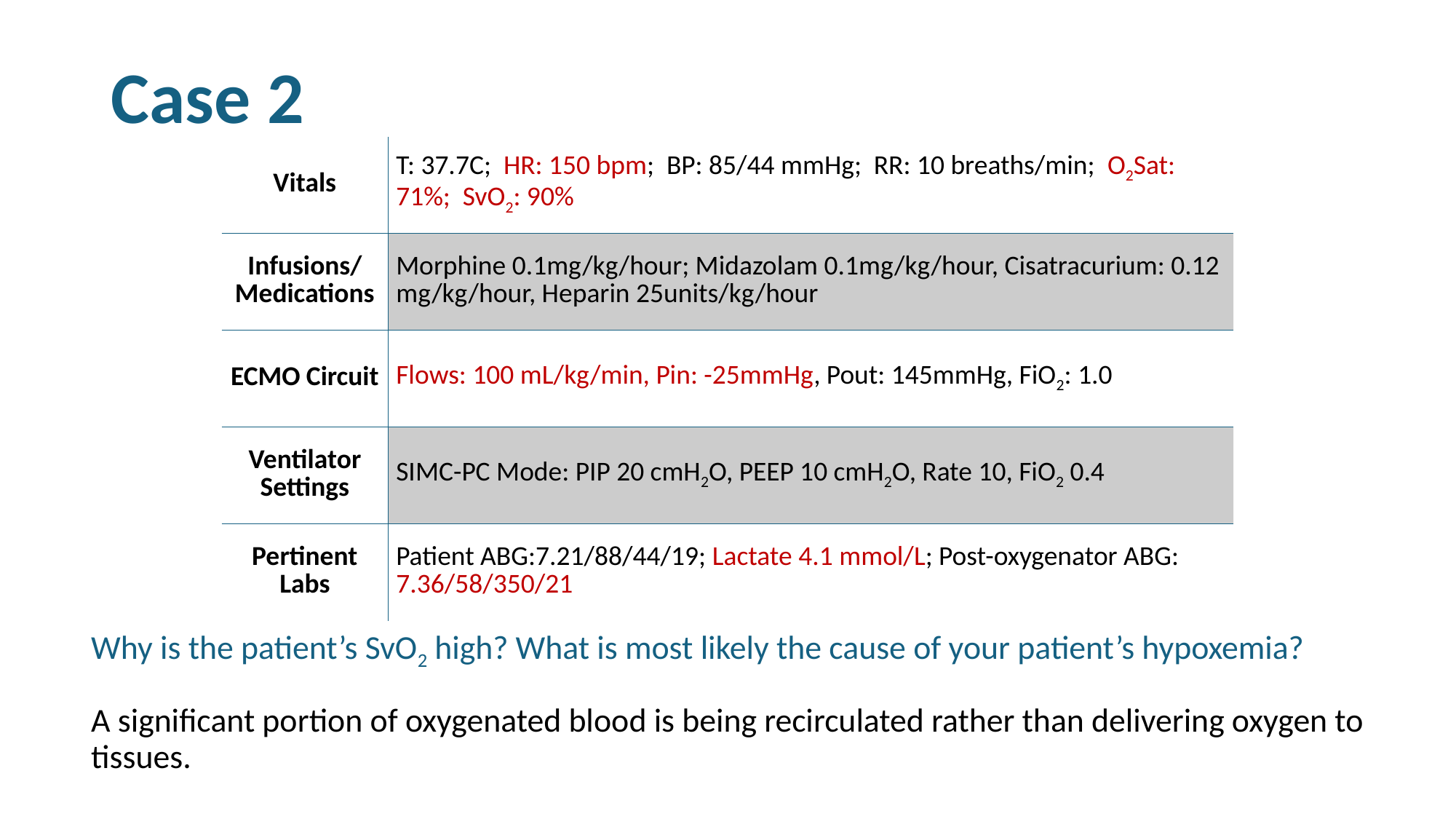

# Case 2
| Vitals | T: 37.7C; HR: 150 bpm; BP: 85/44 mmHg; RR: 10 breaths/min; O2Sat: 71%; SvO2: 90% |
| --- | --- |
| Infusions/ Medications | Morphine 0.1mg/kg/hour; Midazolam 0.1mg/kg/hour, Cisatracurium: 0.12 mg/kg/hour, Heparin 25units/kg/hour |
| ECMO Circuit | Flows: 100 mL/kg/min, Pin: -25mmHg, Pout: 145mmHg, FiO2: 1.0 |
| Ventilator Settings | SIMC-PC Mode: PIP 20 cmH2O, PEEP 10 cmH2O, Rate 10, FiO2 0.4 |
| Pertinent Labs | Patient ABG:7.21/88/44/19; Lactate 4.1 mmol/L; Post-oxygenator ABG: 7.36/58/350/21 |
Why is the patient’s SvO2 high? What is most likely the cause of your patient’s hypoxemia?
A significant portion of oxygenated blood is being recirculated rather than delivering oxygen to tissues.

## Slide 23
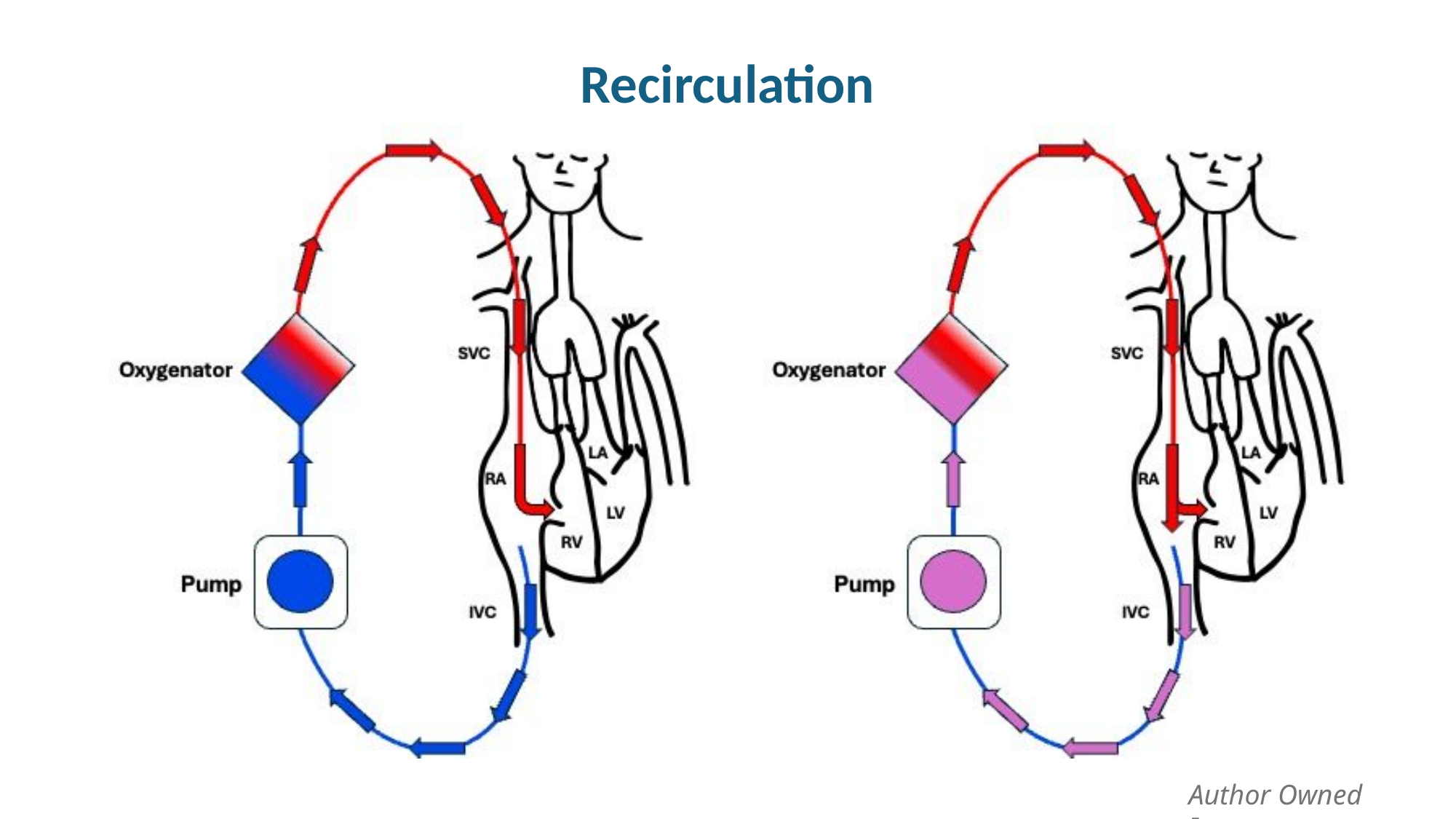

# Recirculation
Author Owned Image

## Slide 24
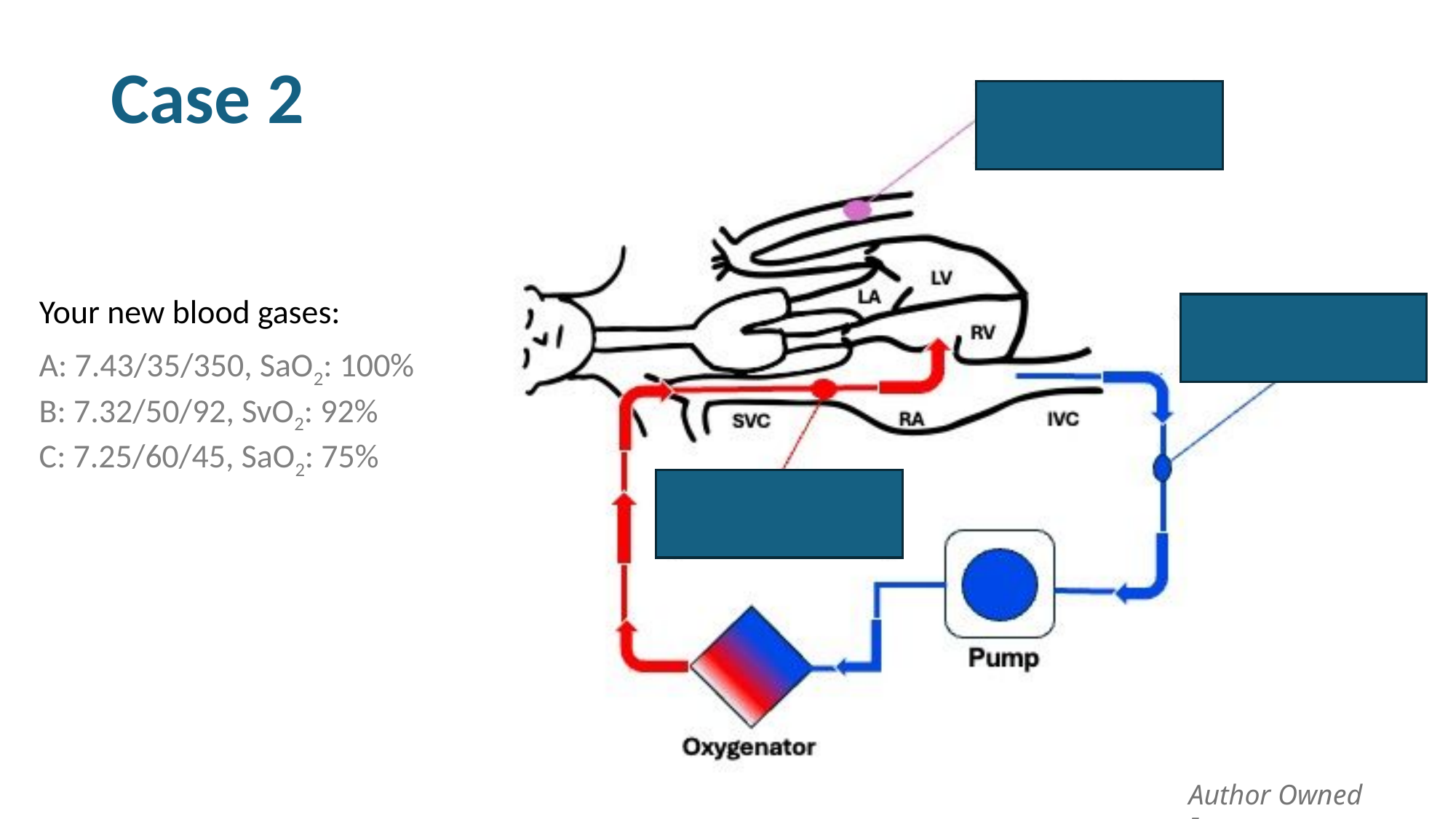

# Case 2
Your new blood gases:
A: 7.43/35/350, SaO2: 100%
B: 7.32/50/92, SvO2: 92%
C: 7.25/60/45, SaO2: 75%
Author Owned Image

## Slide 25
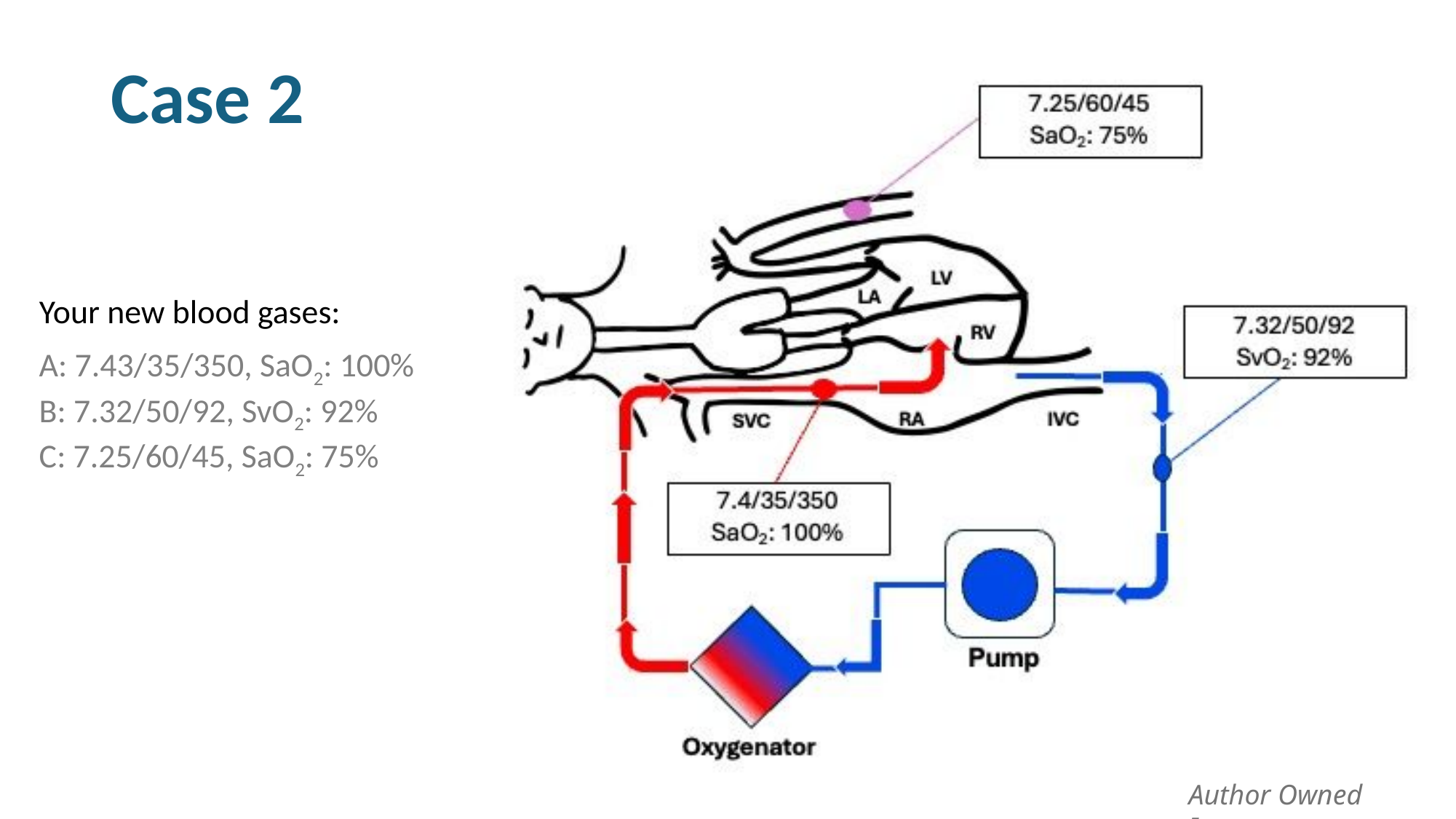

# Case 2
Your new blood gases:
A: 7.43/35/350, SaO2: 100%
B: 7.32/50/92, SvO2: 92%
C: 7.25/60/45, SaO2: 75%
Author Owned Image

## Slide 26
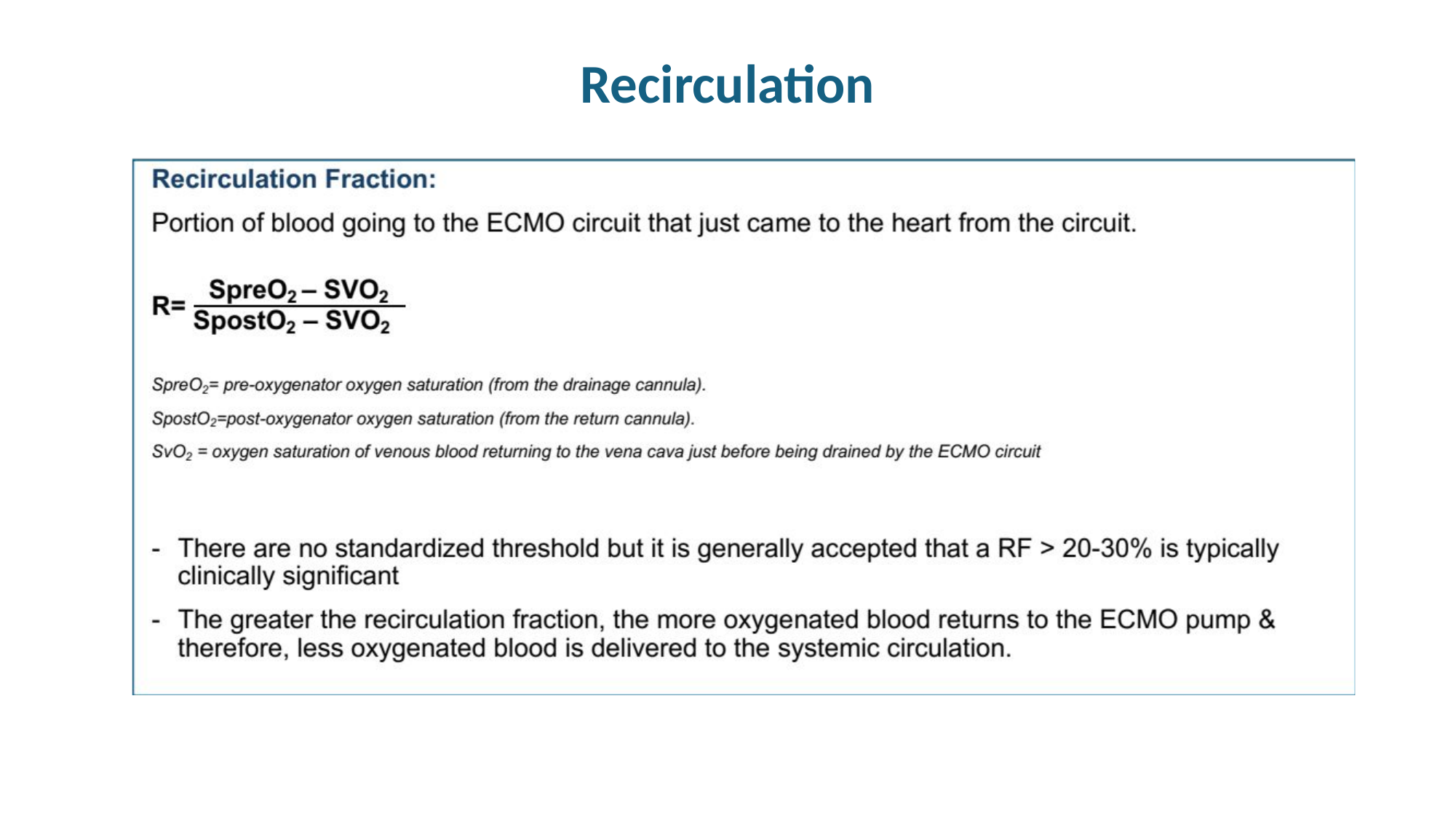

# Recirculation

## Slide 27
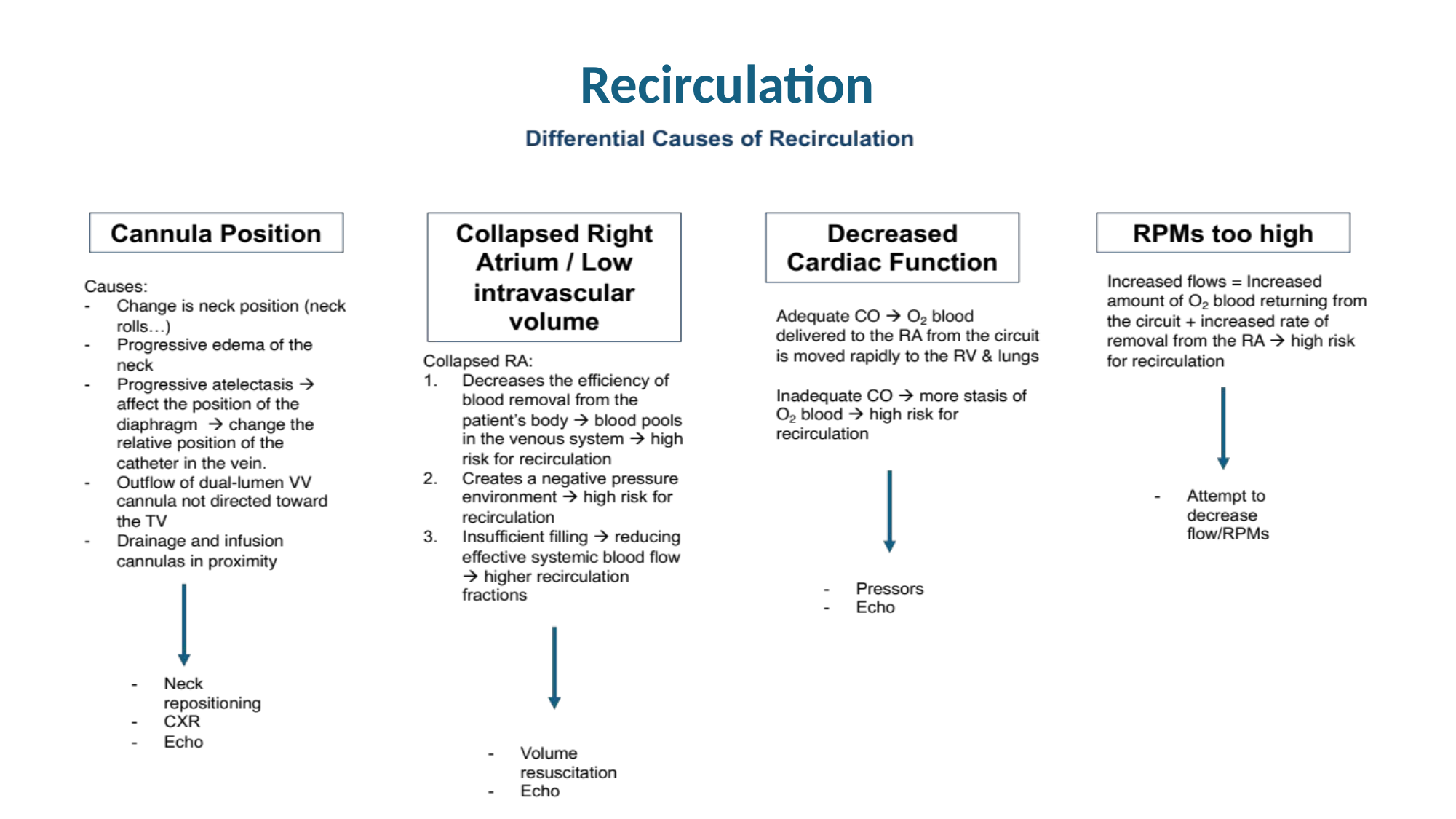

Recirculation

## Slide 28
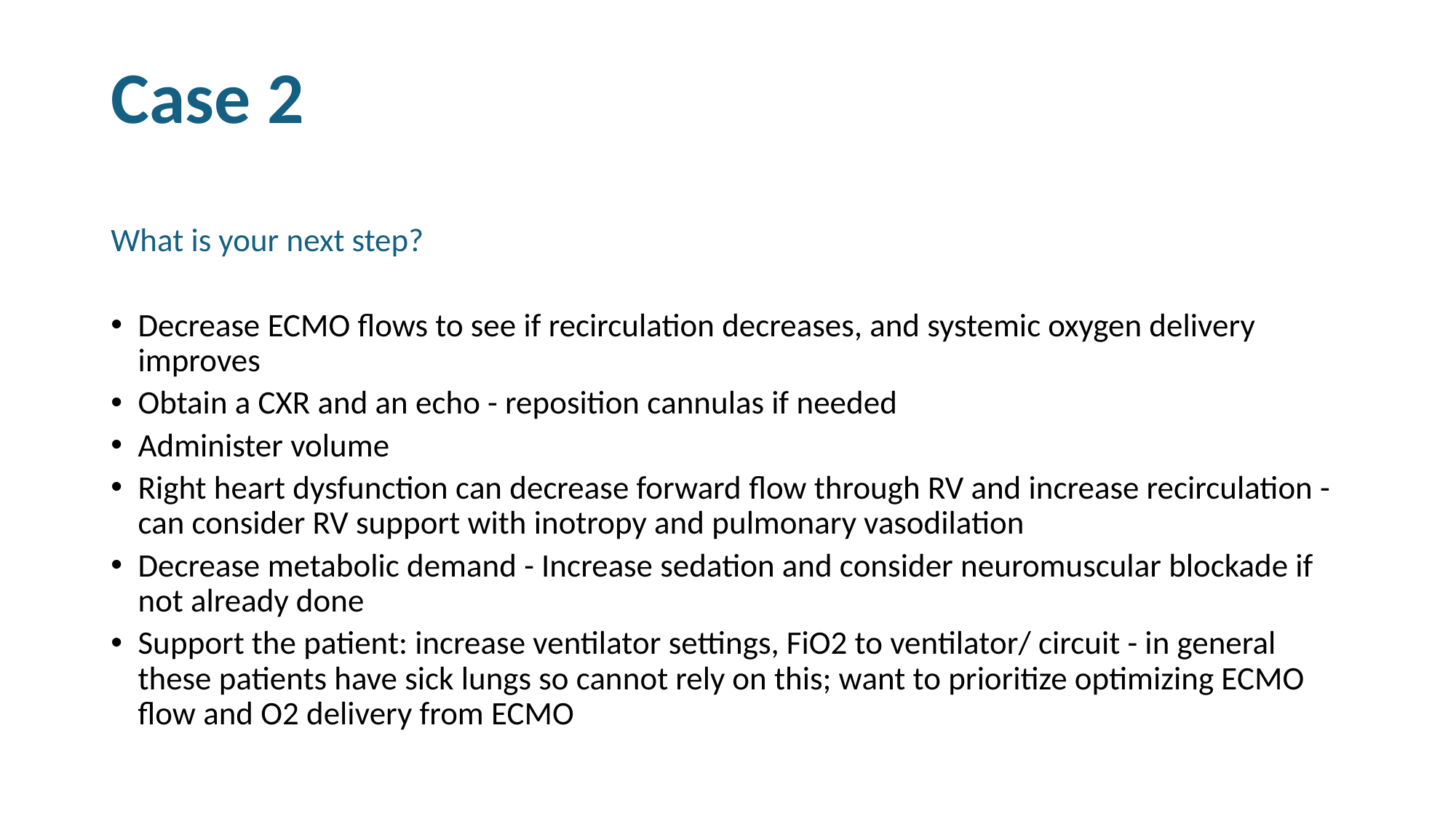

# Case 2
What is your next step?
Decrease ECMO flows to see if recirculation decreases, and systemic oxygen delivery improves
Obtain a CXR and an echo - reposition cannulas if needed
Administer volume
Right heart dysfunction can decrease forward flow through RV and increase recirculation - can consider RV support with inotropy and pulmonary vasodilation
Decrease metabolic demand - Increase sedation and consider neuromuscular blockade if not already done
Support the patient: increase ventilator settings, FiO2 to ventilator/ circuit - in general these patients have sick lungs so cannot rely on this; want to prioritize optimizing ECMO flow and O2 delivery from ECMO

## Slide 29
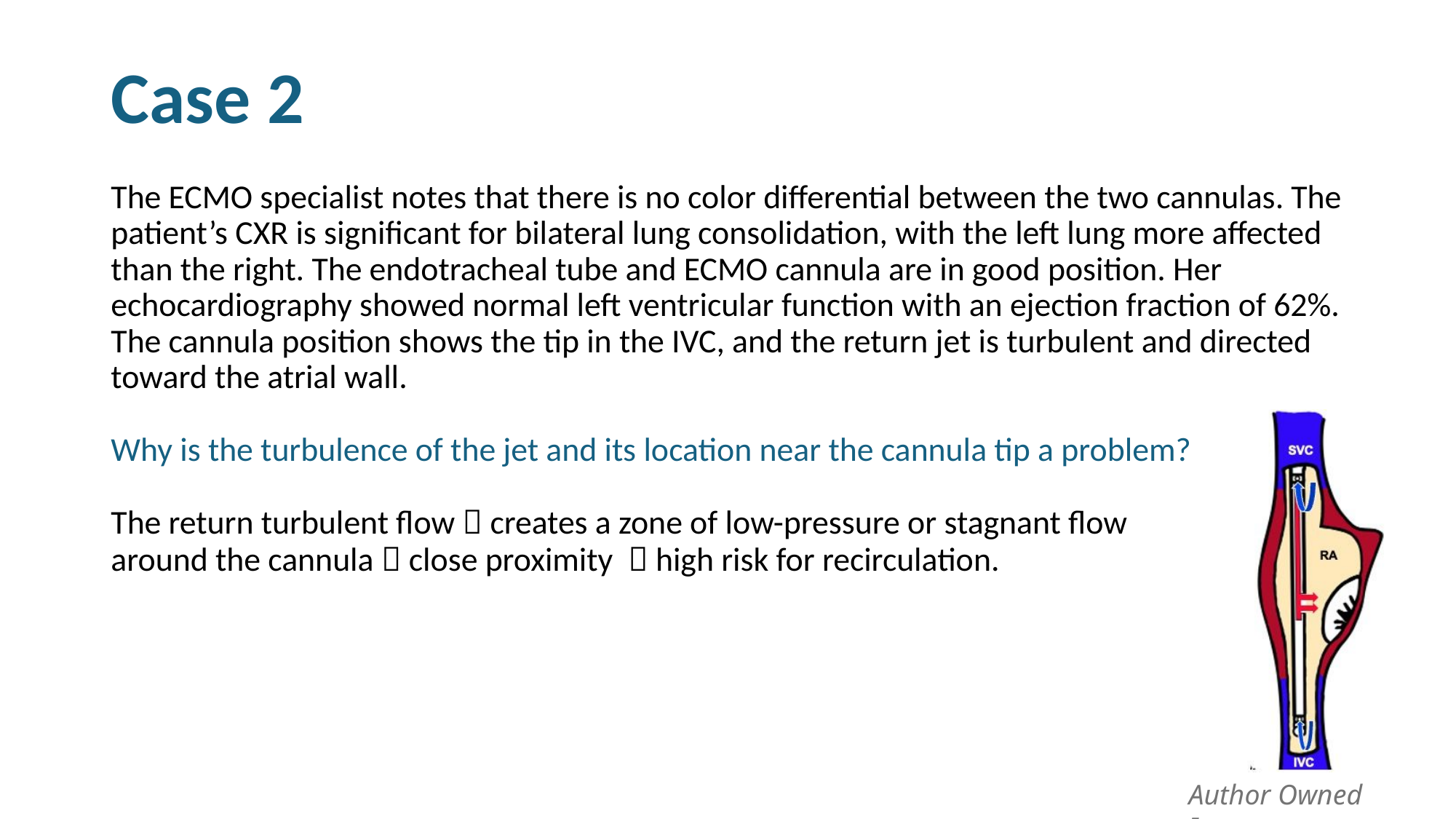

# Case 2
The ECMO specialist notes that there is no color differential between the two cannulas. The patient’s CXR is significant for bilateral lung consolidation, with the left lung more affected than the right. The endotracheal tube and ECMO cannula are in good position. Her echocardiography showed normal left ventricular function with an ejection fraction of 62%. The cannula position shows the tip in the IVC, and the return jet is turbulent and directed toward the atrial wall.
Why is the turbulence of the jet and its location near the cannula tip a problem?
The return turbulent flow  creates a zone of low-pressure or stagnant flow
around the cannula  close proximity  high risk for recirculation.
Author Owned Image

## Slide 30
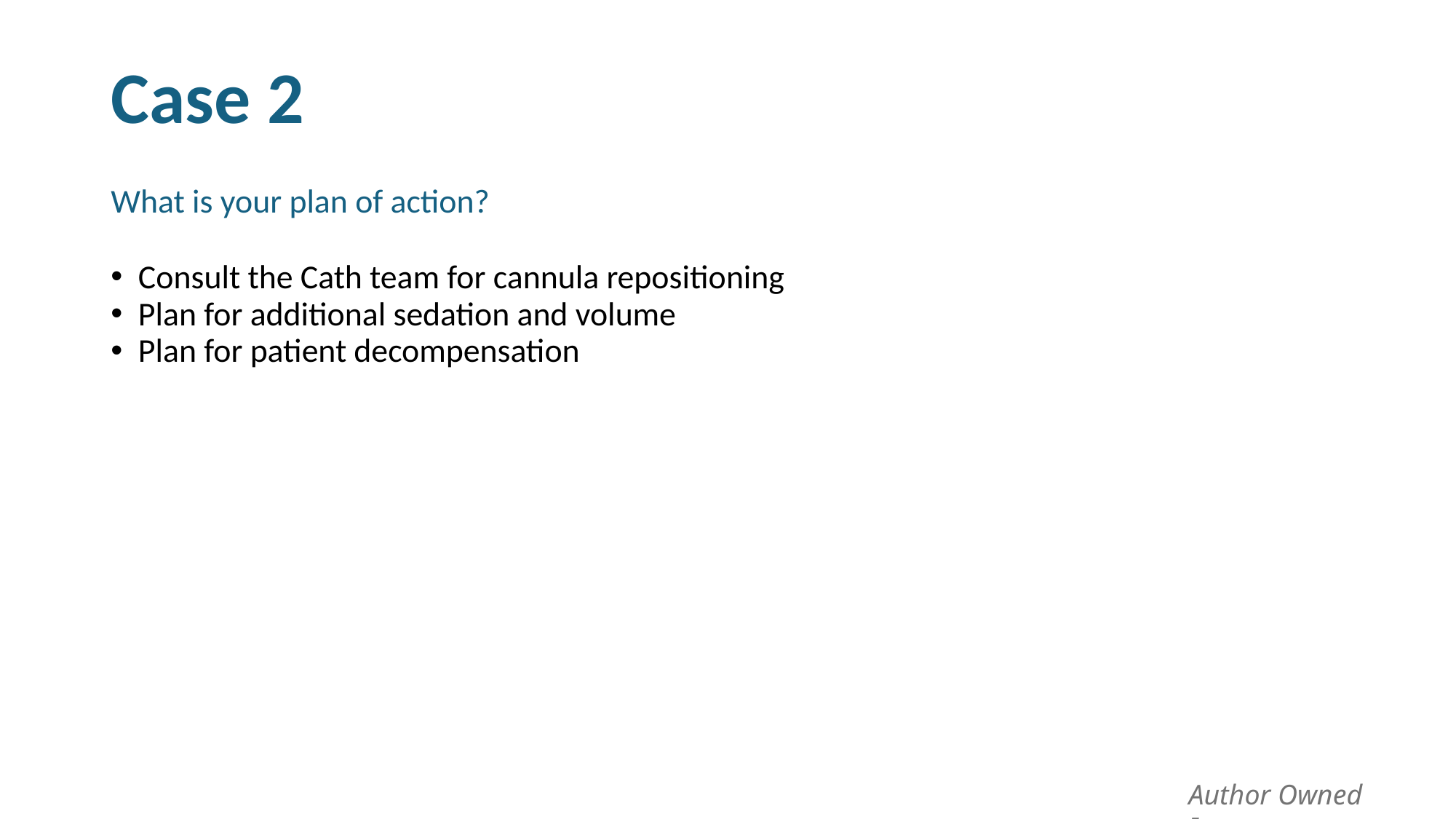

# Case 2
What is your plan of action?
Consult the Cath team for cannula repositioning
Plan for additional sedation and volume
Plan for patient decompensation
Author Owned Image

## Slide 31
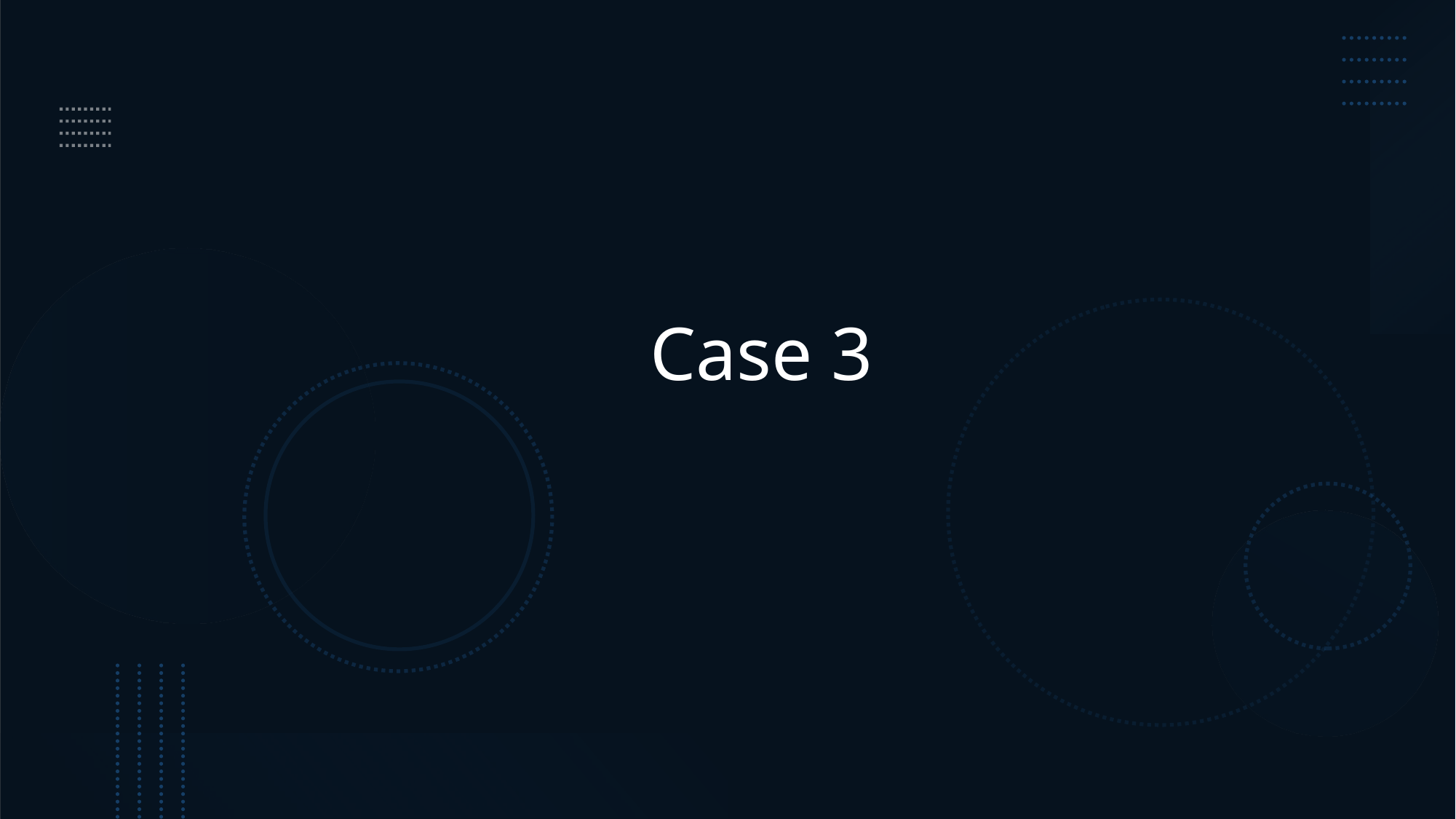

# Case 3

## Slide 32
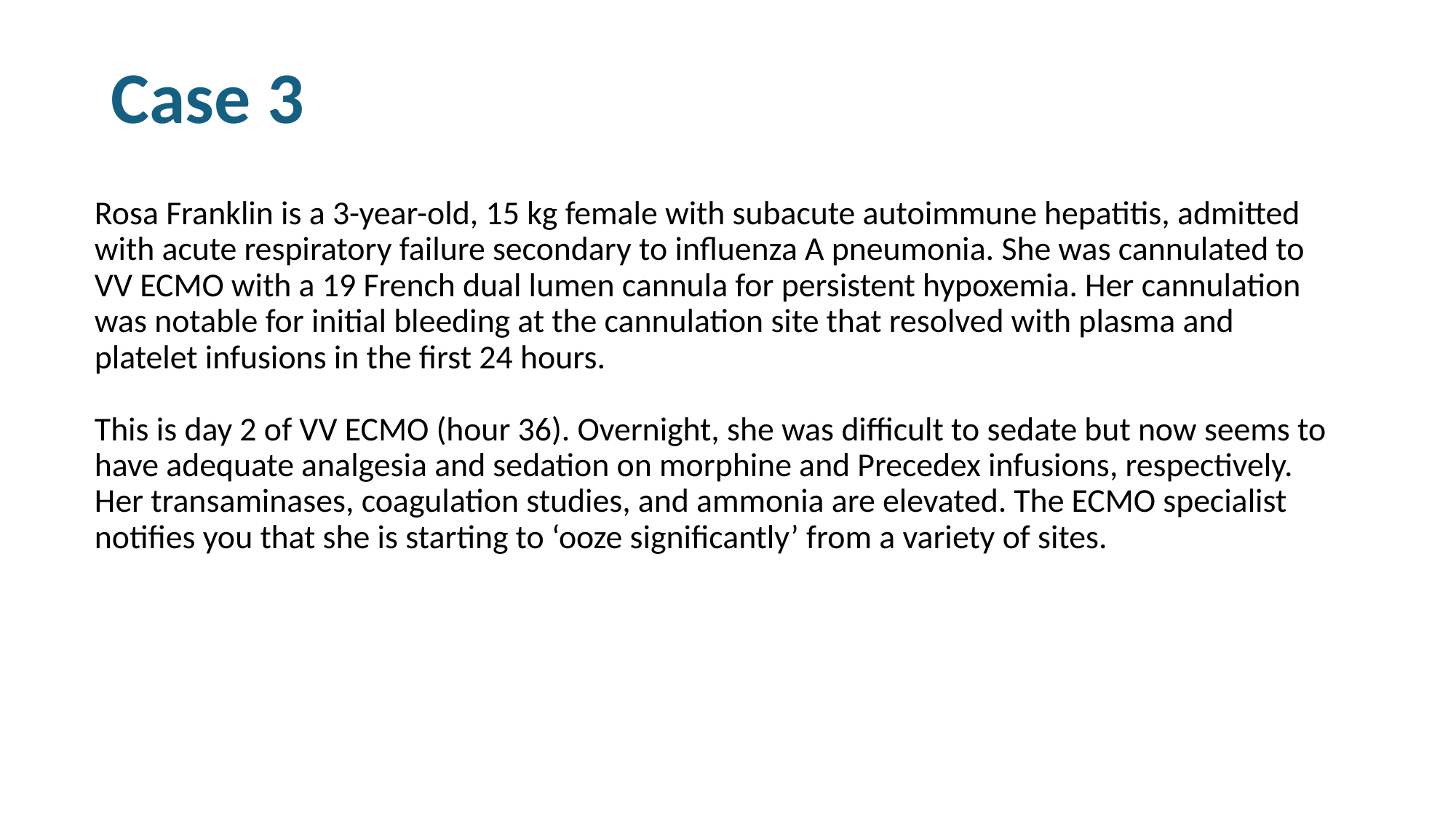

# Case 3
Rosa Franklin is a 3-year-old, 15 kg female with subacute autoimmune hepatitis, admitted with acute respiratory failure secondary to influenza A pneumonia. She was cannulated to VV ECMO with a 19 French dual lumen cannula for persistent hypoxemia. Her cannulation was notable for initial bleeding at the cannulation site that resolved with plasma and platelet infusions in the first 24 hours.
This is day 2 of VV ECMO (hour 36). Overnight, she was difficult to sedate but now seems to have adequate analgesia and sedation on morphine and Precedex infusions, respectively. Her transaminases, coagulation studies, and ammonia are elevated. The ECMO specialist notifies you that she is starting to ‘ooze significantly’ from a variety of sites.

## Slide 33
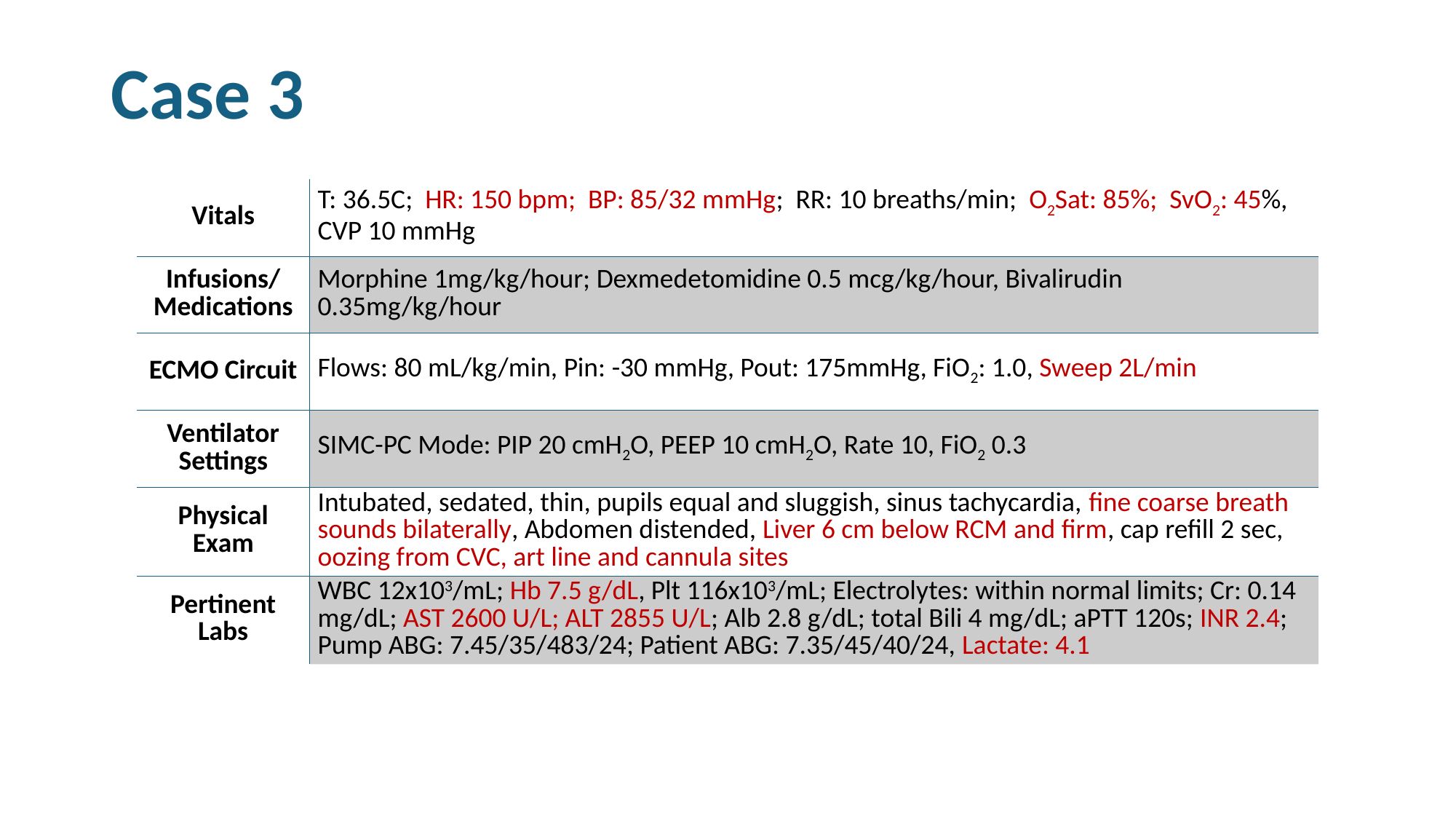

# Case 3
| Vitals | T: 36.5C; HR: 150 bpm; BP: 85/32 mmHg; RR: 10 breaths/min; O2Sat: 85%; SvO2: 45%, CVP 10 mmHg |
| --- | --- |
| Infusions/ Medications | Morphine 1mg/kg/hour; Dexmedetomidine 0.5 mcg/kg/hour, Bivalirudin 0.35mg/kg/hour |
| ECMO Circuit | Flows: 80 mL/kg/min, Pin: -30 mmHg, Pout: 175mmHg, FiO2: 1.0, Sweep 2L/min |
| Ventilator Settings | SIMC-PC Mode: PIP 20 cmH2O, PEEP 10 cmH2O, Rate 10, FiO2 0.3 |
| Physical Exam | Intubated, sedated, thin, pupils equal and sluggish, sinus tachycardia, fine coarse breath sounds bilaterally, Abdomen distended, Liver 6 cm below RCM and firm, cap refill 2 sec, oozing from CVC, art line and cannula sites |
| Pertinent Labs | WBC 12x103/mL; Hb 7.5 g/dL, Plt 116x103/mL; Electrolytes: within normal limits; Cr: 0.14 mg/dL; AST 2600 U/L; ALT 2855 U/L; Alb 2.8 g/dL; total Bili 4 mg/dL; aPTT 120s; INR 2.4; Pump ABG: 7.45/35/483/24; Patient ABG: 7.35/45/40/24, Lactate: 4.1 |

## Slide 34
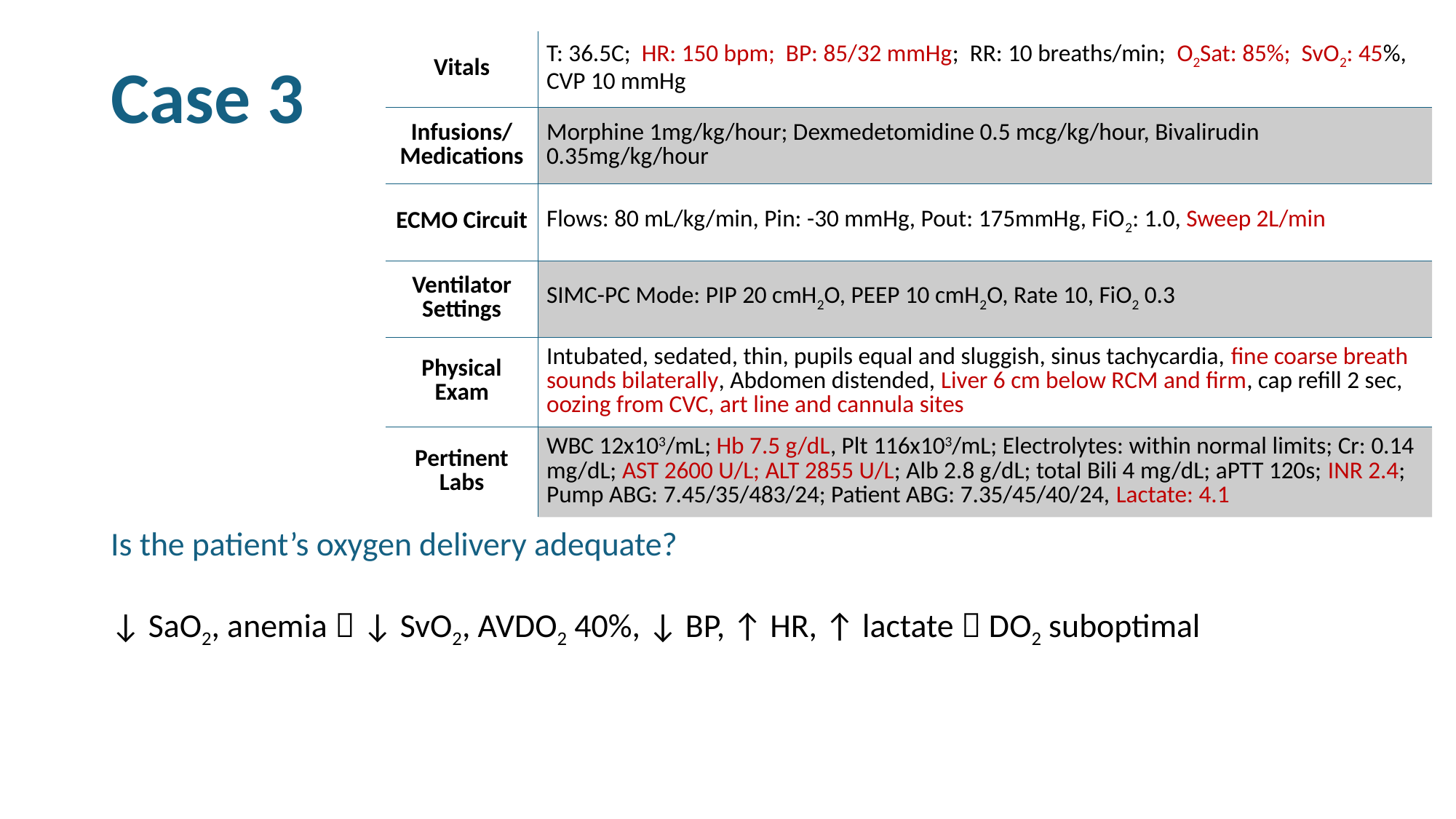

# Case 3
| Vitals | T: 36.5C; HR: 150 bpm; BP: 85/32 mmHg; RR: 10 breaths/min; O2Sat: 85%; SvO2: 45%, CVP 10 mmHg |
| --- | --- |
| Infusions/ Medications | Morphine 1mg/kg/hour; Dexmedetomidine 0.5 mcg/kg/hour, Bivalirudin 0.35mg/kg/hour |
| ECMO Circuit | Flows: 80 mL/kg/min, Pin: -30 mmHg, Pout: 175mmHg, FiO2: 1.0, Sweep 2L/min |
| Ventilator Settings | SIMC-PC Mode: PIP 20 cmH2O, PEEP 10 cmH2O, Rate 10, FiO2 0.3 |
| Physical Exam | Intubated, sedated, thin, pupils equal and sluggish, sinus tachycardia, fine coarse breath sounds bilaterally, Abdomen distended, Liver 6 cm below RCM and firm, cap refill 2 sec, oozing from CVC, art line and cannula sites |
| Pertinent Labs | WBC 12x103/mL; Hb 7.5 g/dL, Plt 116x103/mL; Electrolytes: within normal limits; Cr: 0.14 mg/dL; AST 2600 U/L; ALT 2855 U/L; Alb 2.8 g/dL; total Bili 4 mg/dL; aPTT 120s; INR 2.4; Pump ABG: 7.45/35/483/24; Patient ABG: 7.35/45/40/24, Lactate: 4.1 |
Is the patient’s oxygen delivery adequate?
↓ SaO2, anemia  ↓ SvO2, AVDO2 40%, ↓ BP, ↑ HR, ↑ lactate  DO2 suboptimal

## Slide 35
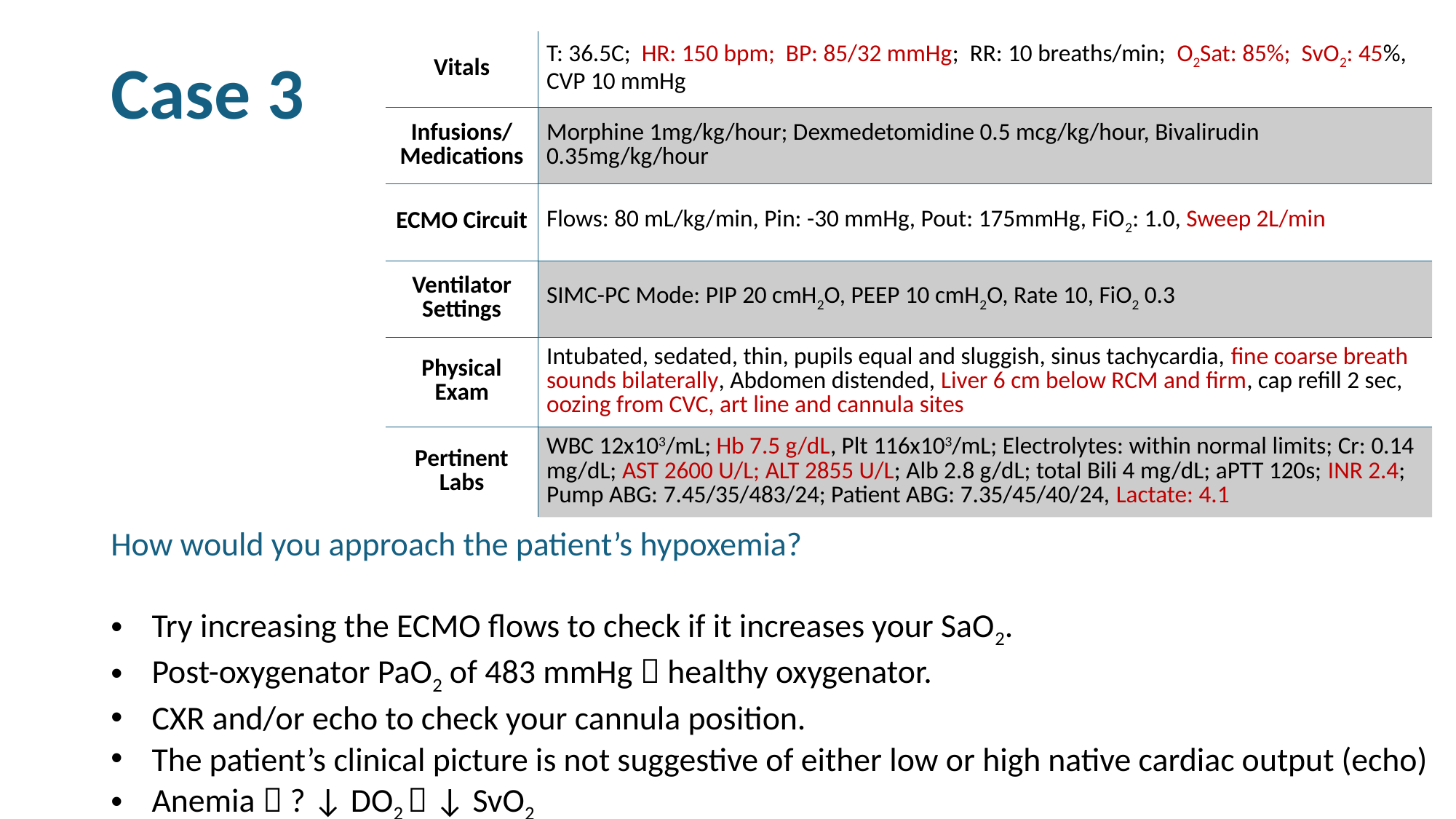

# Case 3
| Vitals | T: 36.5C; HR: 150 bpm; BP: 85/32 mmHg; RR: 10 breaths/min; O2Sat: 85%; SvO2: 45%, CVP 10 mmHg |
| --- | --- |
| Infusions/ Medications | Morphine 1mg/kg/hour; Dexmedetomidine 0.5 mcg/kg/hour, Bivalirudin 0.35mg/kg/hour |
| ECMO Circuit | Flows: 80 mL/kg/min, Pin: -30 mmHg, Pout: 175mmHg, FiO2: 1.0, Sweep 2L/min |
| Ventilator Settings | SIMC-PC Mode: PIP 20 cmH2O, PEEP 10 cmH2O, Rate 10, FiO2 0.3 |
| Physical Exam | Intubated, sedated, thin, pupils equal and sluggish, sinus tachycardia, fine coarse breath sounds bilaterally, Abdomen distended, Liver 6 cm below RCM and firm, cap refill 2 sec, oozing from CVC, art line and cannula sites |
| Pertinent Labs | WBC 12x103/mL; Hb 7.5 g/dL, Plt 116x103/mL; Electrolytes: within normal limits; Cr: 0.14 mg/dL; AST 2600 U/L; ALT 2855 U/L; Alb 2.8 g/dL; total Bili 4 mg/dL; aPTT 120s; INR 2.4; Pump ABG: 7.45/35/483/24; Patient ABG: 7.35/45/40/24, Lactate: 4.1 |
How would you approach the patient’s hypoxemia?
Try increasing the ECMO flows to check if it increases your SaO2.
Post-oxygenator PaO2 of 483 mmHg  healthy oxygenator.
CXR and/or echo to check your cannula position.
The patient’s clinical picture is not suggestive of either low or high native cardiac output (echo)
Anemia  ? ↓ DO2  ↓ SvO2

## Slide 36
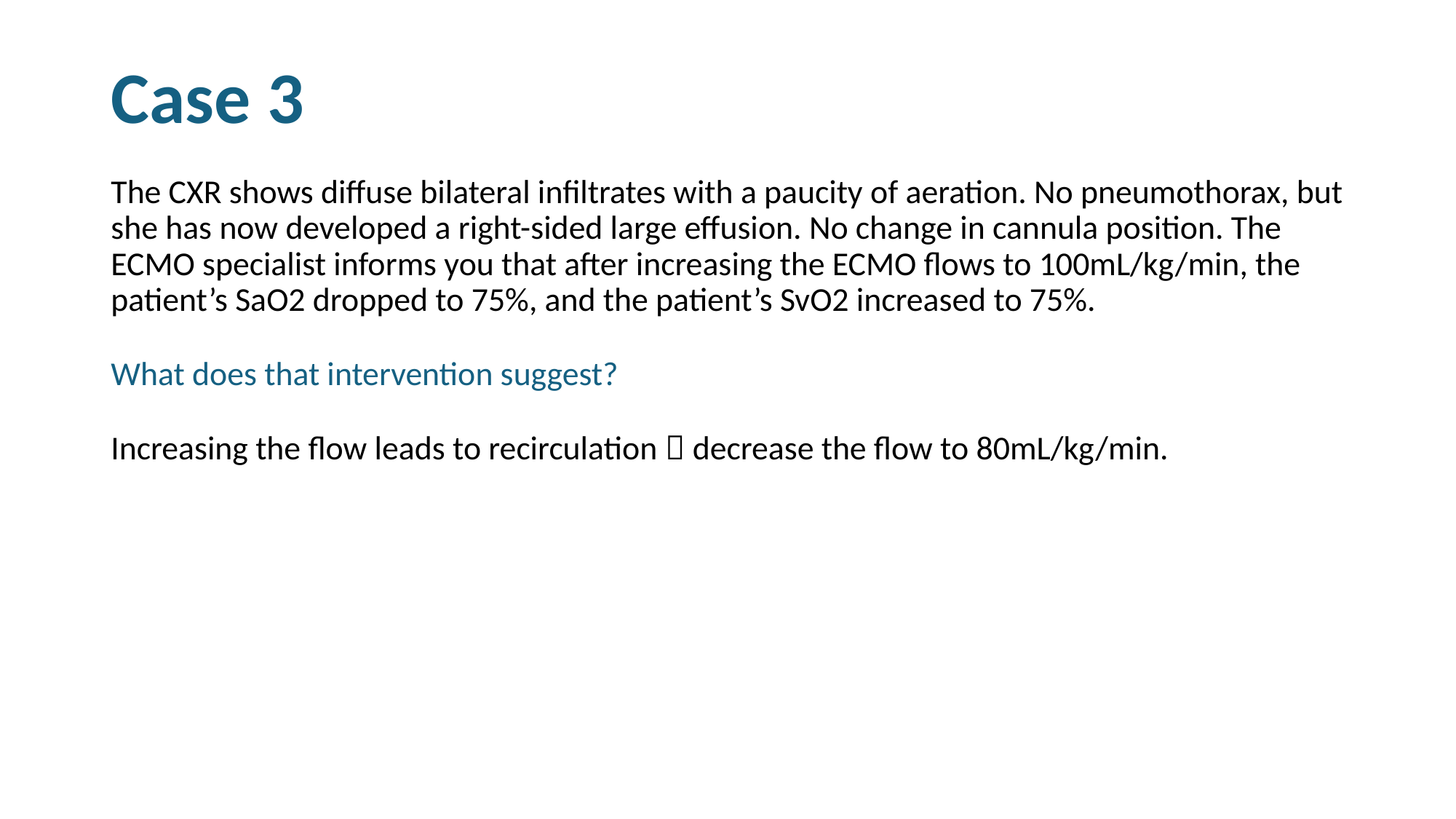

# Case 3
The CXR shows diffuse bilateral infiltrates with a paucity of aeration. No pneumothorax, but she has now developed a right-sided large effusion. No change in cannula position. The ECMO specialist informs you that after increasing the ECMO flows to 100mL/kg/min, the patient’s SaO2 dropped to 75%, and the patient’s SvO2 increased to 75%.
What does that intervention suggest?
Increasing the flow leads to recirculation  decrease the flow to 80mL/kg/min.

## Slide 37
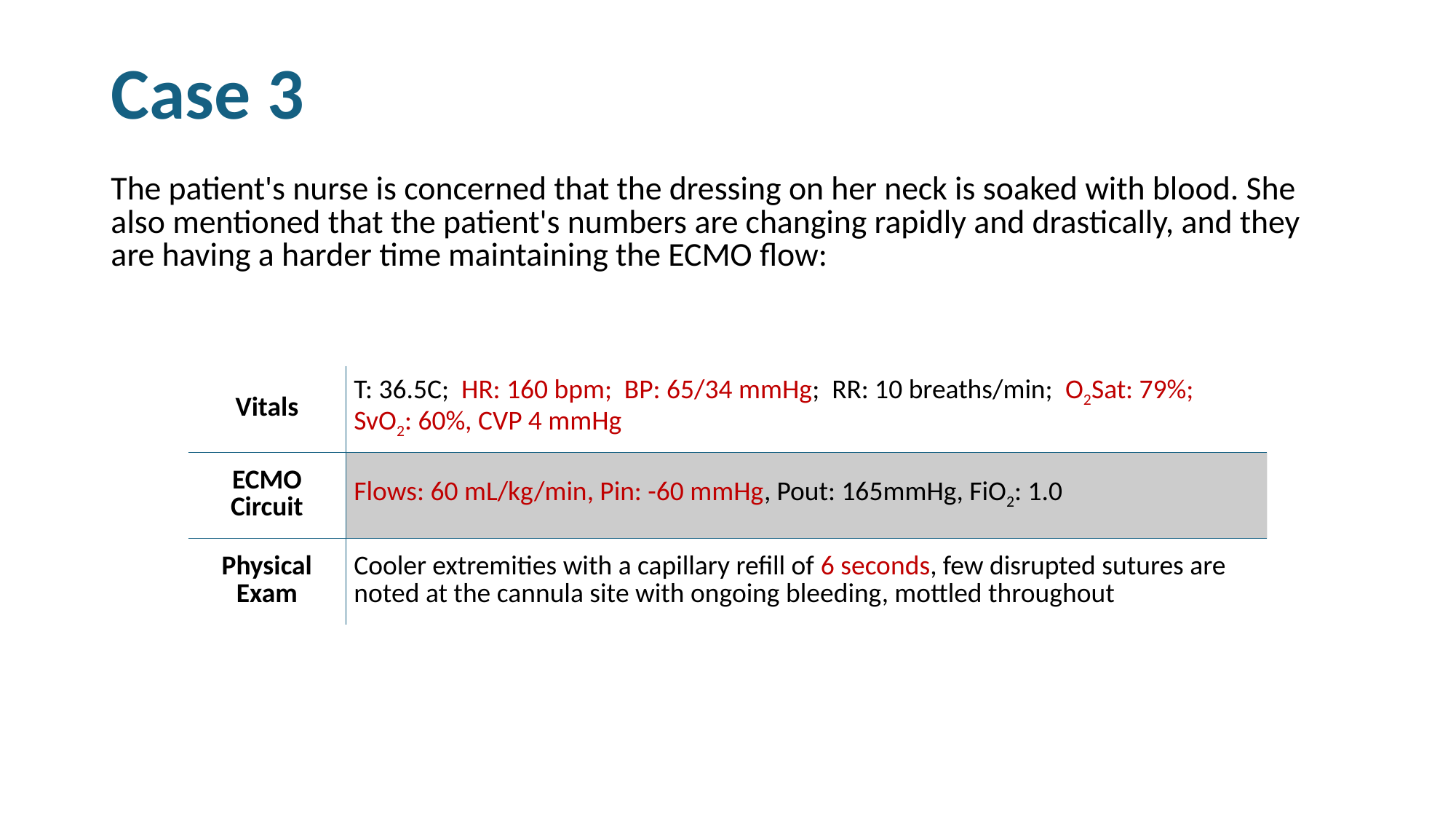

# Case 3
The patient's nurse is concerned that the dressing on her neck is soaked with blood. She also mentioned that the patient's numbers are changing rapidly and drastically, and they are having a harder time maintaining the ECMO flow:
| Vitals | T: 36.5C; HR: 160 bpm; BP: 65/34 mmHg; RR: 10 breaths/min; O2Sat: 79%; SvO2: 60%, CVP 4 mmHg |
| --- | --- |
| ECMO Circuit | Flows: 60 mL/kg/min, Pin: -60 mmHg, Pout: 165mmHg, FiO2: 1.0 |
| Physical Exam | Cooler extremities with a capillary refill of 6 seconds, few disrupted sutures are noted at the cannula site with ongoing bleeding, mottled throughout |

## Slide 38
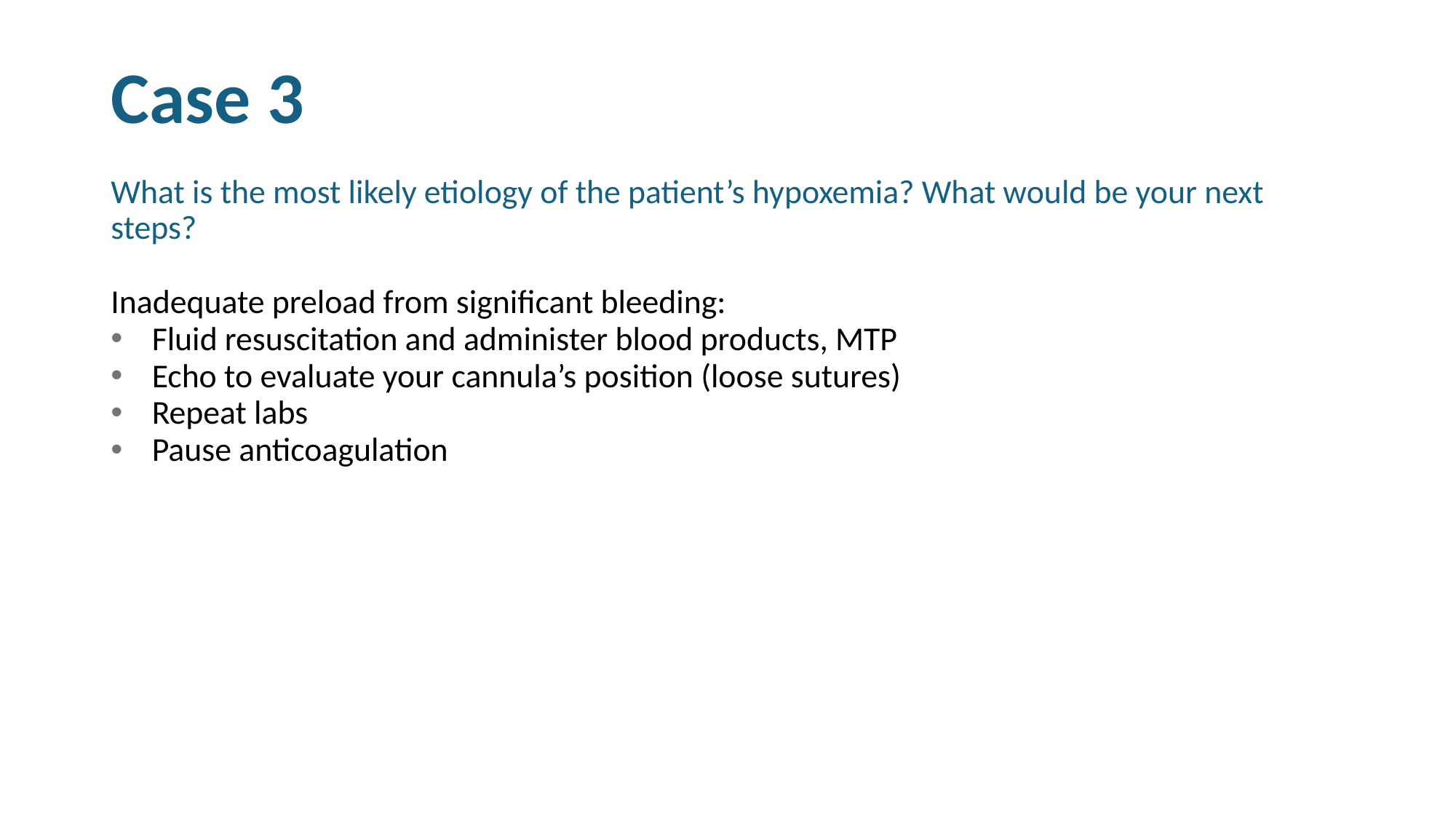

# Case 3
What is the most likely etiology of the patient’s hypoxemia? What would be your next steps?
Inadequate preload from significant bleeding:
Fluid resuscitation and administer blood products, MTP
Echo to evaluate your cannula’s position (loose sutures)
Repeat labs
Pause anticoagulation

## Slide 39
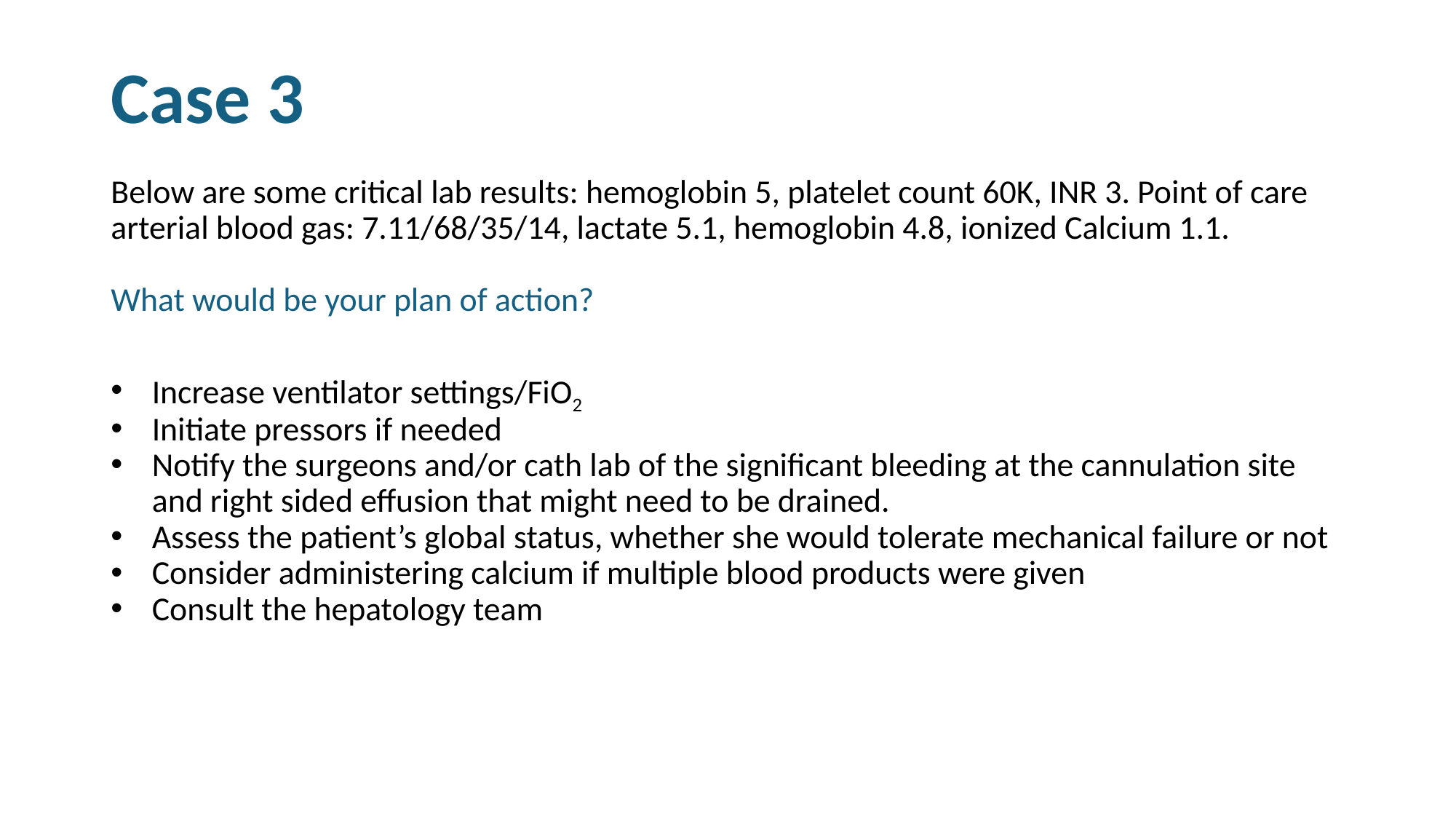

# Case 3
Below are some critical lab results: hemoglobin 5, platelet count 60K, INR 3. Point of care arterial blood gas: 7.11/68/35/14, lactate 5.1, hemoglobin 4.8, ionized Calcium 1.1.
What would be your plan of action?
Increase ventilator settings/FiO2
Initiate pressors if needed
Notify the surgeons and/or cath lab of the significant bleeding at the cannulation site and right sided effusion that might need to be drained.
Assess the patient’s global status, whether she would tolerate mechanical failure or not
Consider administering calcium if multiple blood products were given
Consult the hepatology team

## Slide 40
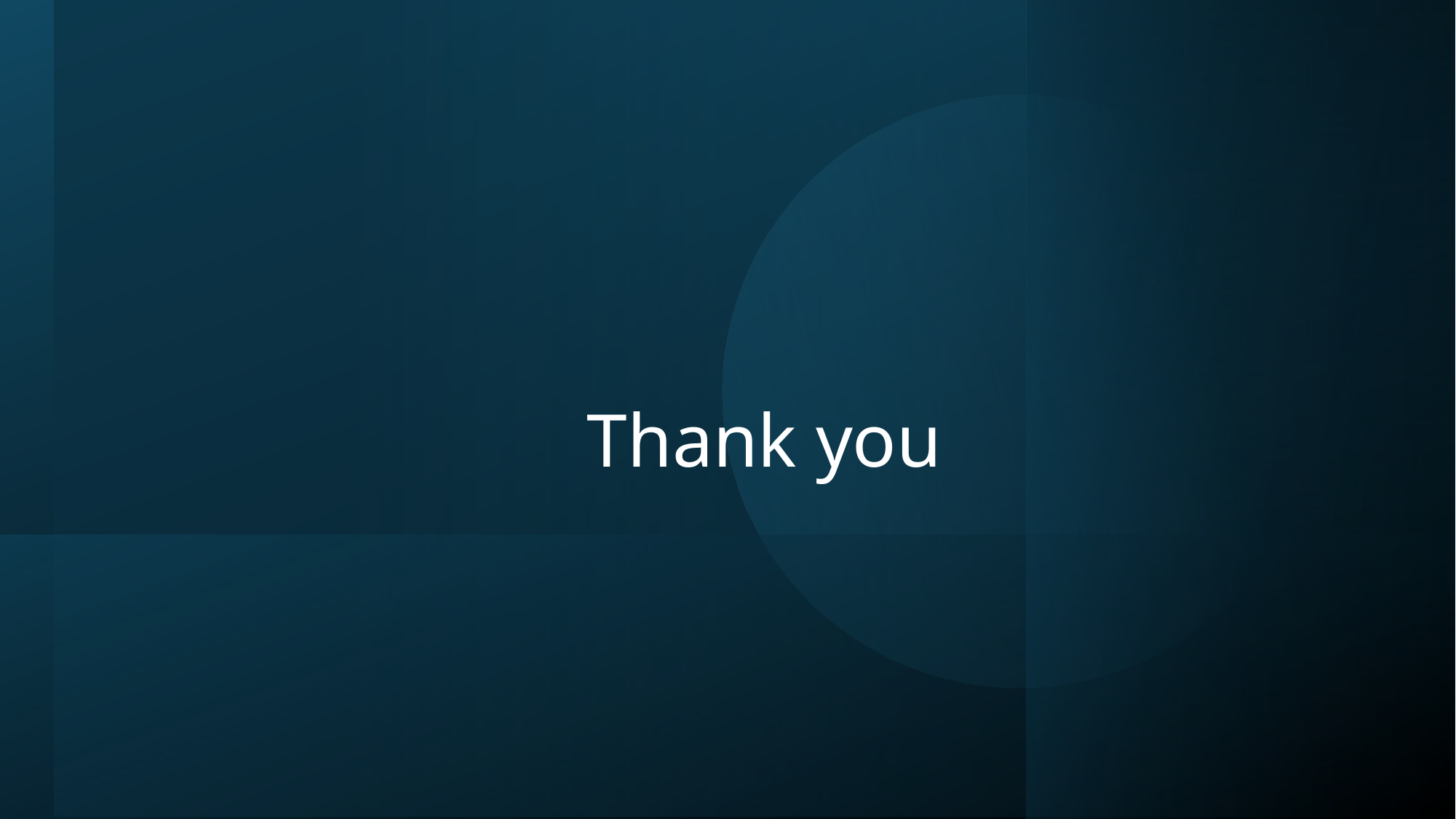

# Thank you

## Slide 41
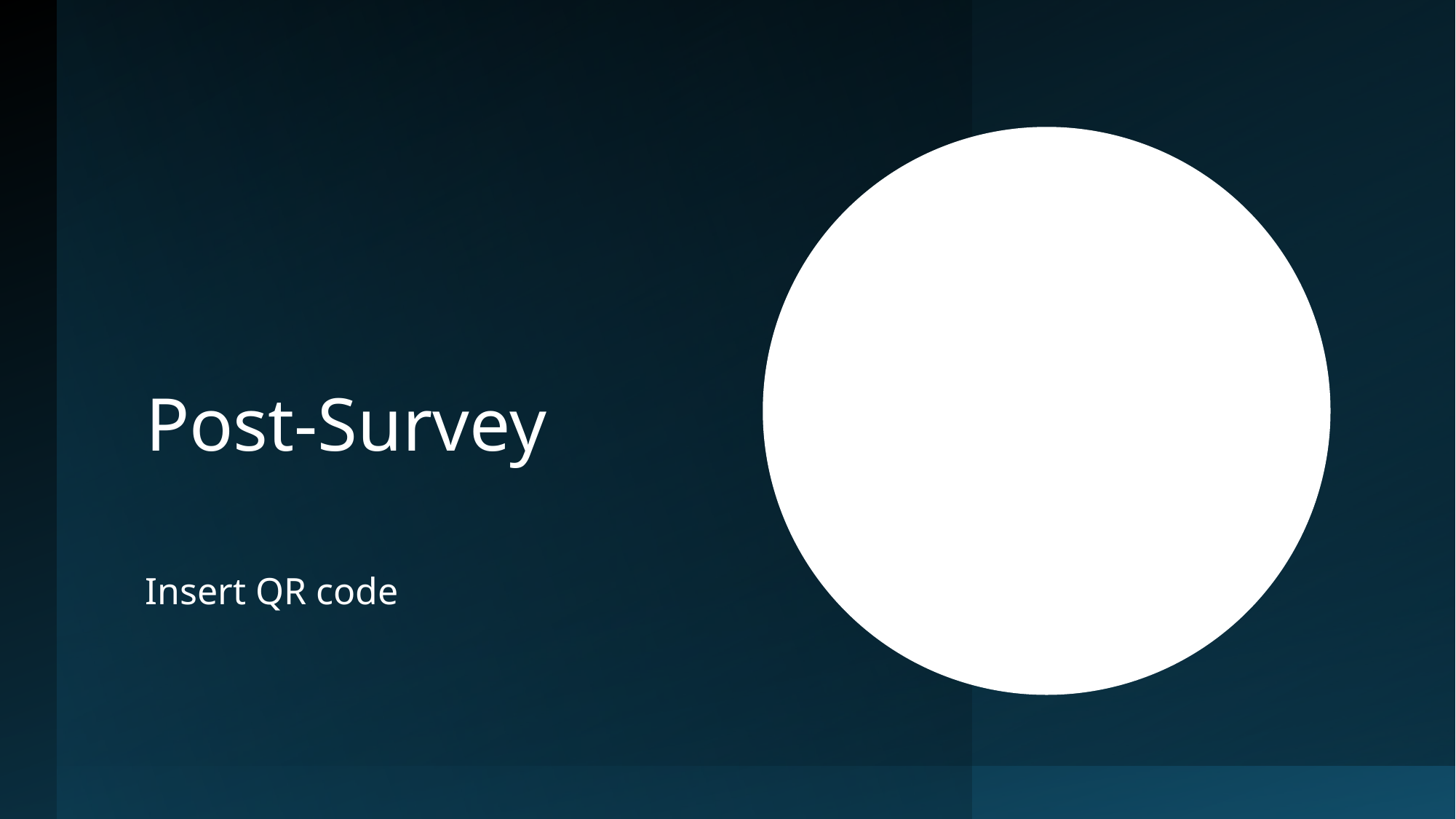

# Post-Survey
Insert QR code
