# Supplementary material for: Enhancing Pediatric Extracorporeal Membrane Oxygenation Education Through Process-Oriented Guided Inquiry Learning Sessions for Fellows and Advanced Practice Providers
Source: MedEdPORTAL. 2026 May 12;22:11600. doi: 10.15766/mep_2374-8265.11600 (PMC13161199; doi:10.15766/mep_2374-8265.11600)
Supplement: Supplementary file 1 — VA-ECMO Learner Handout.docxVV-ECMO Learner Handout.docxVA-ECMO Facilitator Guide.docxVV-ECMO Facilitator Guide.docxVA-ECMO Slides.pptxVV-ECMO Slides.pptxVA-ECMO Presurvey.docxVV-ECMO Presurvey.docxVA-ECMO Postsurvey.docxVV-ECMO Postsurvey.docx [file mep_2374-8265.11600-s001.zip › H. VV-ECMO Presurvey.docx]

**Hypoxemia on VV-ECMO- Pre-Survey**

***This survey is administered to learners before the VV-ECMO session to assess baseline knowledge and confidence.***

***Thank you for completing the survey below before your next ECMO education session.***

1. **Please create a unique identifier using the following format: the first two letters of the high school you attended followed by the first three letters of your favorite color: ______**
2. **Please indicate your role:**

- Pediatric critical care fellow
- Pediatric cardiology fellow
- Pediatric APP
- Pediatric resident
- Attending Physician

1. ***If you are a fellow or a resident:* Please indicate your level: _____**
2. ***Please indicate the extent to which you agree with the following statements:***

| I can generate a differential diagnosis for hypoxemia in patients on VV-ECMO | Strongly agree | Somewhat agree | Neutral | Somewhat disagree | Strongly disagree |
| --- | --- | --- | --- | --- | --- |
| I can identify the clinical signs and parameters that indicate oxygenator failure on VV-ECMO | Strongly agree | Somewhat agree | Neutral | Somewhat disagree | Strongly disagree |
| I can identify the clinical signs and parameters that indicate recirculation on VV-ECMO | Strongly agree | Somewhat agree | Neutral | Somewhat disagree | Strongly disagree |
| I can identify the clinical signs and parameters indicative of decreased preload as an etiology of hypoxemia on VV-ECMO | Strongly agree | Somewhat agree | Neutral | Somewhat disagree | Strongly disagree |

1. **Please answer the following questions to the best of your ability:**
2. ***What are some advantages of V-V ECMO?***

- Lower oxygen saturations
- Normal pulsatile flow to the Kidneys
- Recirculation
- Perfusion of the coronary arteries with oxygenated blood
- Decreased risk of stroke
- b, d, and e
- All of the above
- Unsure

1. ***What monitor (patient and circuit) data is a hallmark of recirculation in VV ECMO? Select all that apply.***
   - ↓SvO_2_
   - ↑ SvO_2_
   - Decreased ECMO flow
   - ↑Patient SaO_2_
   - ↓Patient SaO_2_
   - More positive Pin
   - ↓CVP
   - Hypertension
   - Unsure
2. ***In a patient on VV-ECMO, match the following observations with their most likely underlying cause:***

|  | **ECMO Flow/Circuit Issue** | **Recirculation** | **Low Native Cardiac Output** | **Poor Oxygen Delivery** | **Unsure** |
| --- | --- | --- | --- | --- | --- |
| O_2_ saturation from the drainage cannula is high, but systemic O_2_ is low |  | x |  |  |  |
| Low SvO_2_ in the setting of high metabolic demand state |  |  |  | x |  |
| High ECMO flow, good cannula position and low systemic O_2_ saturation |  |  | x |  |  |
| O_2_ saturation improved with increasing ECMO flows | x |  |  |  |  |
| Low post-oxygenator PaO_2_ | x |  |  |  |  |

**Knowledge Questions Answers:**

1. b,d, and e
2. ↑ SvO_2_ and ↓Patient SaO_2_
3. *Boxes are checked*
