# Supplementary material for: Enhancing Pediatric Extracorporeal Membrane Oxygenation Education Through Process-Oriented Guided Inquiry Learning Sessions for Fellows and Advanced Practice Providers
Source: MedEdPORTAL. 2026 May 12;22:11600. doi: 10.15766/mep_2374-8265.11600 (PMC13161199; doi:10.15766/mep_2374-8265.11600)
Supplement: Supplementary file 1 — VA-ECMO Learner Handout.docxVV-ECMO Learner Handout.docxVA-ECMO Facilitator Guide.docxVV-ECMO Facilitator Guide.docxVA-ECMO Slides.pptxVV-ECMO Slides.pptxVA-ECMO Presurvey.docxVV-ECMO Presurvey.docxVA-ECMO Postsurvey.docxVV-ECMO Postsurvey.docx [file mep_2374-8265.11600-s001.zip › I. VA-ECMO Postsurvey.docx]

**Low Flow on VA-ECMO- Post-Survey**

***This survey is administered to learners after the VA-ECMO session to assess changes in knowledge, confidence, and educational impact.***

***Thank you for attending the ECMO education session. Your feedback and response to this survey will help improve the session’s structure and content.***

1. **Please enter your unique identifier using the following format: the first two letters of the high school you attended followed by the first three letters of your favorite color: ______**
2. **Please indicate your role:**

- Pediatric critical care fellow
- Pediatric cardiology fellow
- Pediatric APP
- Pediatric resident
- Attending Physician

1. ***If you are a fellow or a resident:* Please indicate your level: _____**
2. ***We are hoping to evaluate whether the objectives of this session were met. By the end of the session:***

| When encountering a low flow state on VA-ECMO, I can generate a differential diagnosis | Strongly agree | Somewhat agree | Neutral | Somewhat disagree | Strongly disagree |
| --- | --- | --- | --- | --- | --- |
| I can identify the clinical signs and parameters indicative of elevated afterload on VA-ECMO | Strongly agree | Somewhat agree | Neutral | Somewhat disagree | Strongly disagree |
| I can identify the clinical signs and parameters indicative of low preload on VA-ECMO | Strongly agree | Somewhat agree | Neutral | Somewhat disagree | Strongly disagree |

1. ***Please indicate the extent to which you agree with the following statements related to the content of the session.***

| The content covered during this session was relevant to caring for critically ill children on VA-ECMO | Strongly agree | Somewhat agree | Neutral | Somewhat disagree | Strongly disagree |
| --- | --- | --- | --- | --- | --- |
| The content covered during this session was appropriate for my level of training | Strongly agree | Somewhat agree | Neutral | Somewhat disagree | Strongly disagree |

1. **Please indicate the extent to which you agree with the following statements related to the quality of the session and facilitator performance.**

| The session was well organized and easy to follow | Strongly agree | Somewhat agree | Neutral | Somewhat disagree | Strongly disagree |
| --- | --- | --- | --- | --- | --- |
| The facilitator effectively guided the session and discussion | Strongly agree | Somewhat agree | Neutral | Somewhat disagree | Strongly disagree |
| The facilitator encouraged active participation and engagement | Strongly agree | Somewhat agree | Neutral | Somewhat disagree | Strongly disagree |
| Overall, this was a high-quality educational session | Strongly agree | Somewhat agree | Neutral | Somewhat disagree | Strongly disagree |

1. **Please answer the following questions to the best of your ability:**
2. Which set of parameters is consistent with elevated afterload in a patient with a low flow on VA ECMO?

- ECMO flow rate ↓, the pump speed ↓, Pin, and Pout ↓
- ECMO flow rate ↓, the pump speed ↑, Pin ↑, and Pout ↓
- ECMO flow rate ↓, the pump speed is stable, Pin and Pout ↑
- ECMO flow rate ↓, the pump speed is stable, Pin and Pout are unchanged
- I don’t know

Ari Curry is a 7-month-old girl who was admitted last week to the ICU for AHRF in the setting of ARDS and septic shock. She was on very high settings and eventually cannulated to cervical VA-ECMO after a brief cardiac arrest. Hours after cannulation, she became hypotensive after her bag suction.

| **Vitals** | T: 36.8C; HR: 70 bpm; BP: 60/45 mmHg; RR: 35 breaths/min; O_2_Sat: 92%; SvO_2_ 20%, CVP 15 mmHg |
| --- | --- |
| **Drips** | Fentanyl 2 mcg/kg/hr, Precedex 0.7 mcg/kg/hr, Heparin 25u/kg/hr |
| **ECMO Circuit** | Flows: 58 mL/kg/min, Extreme negative inlet pressure, Decreased Pout |
| **Ventilator Settings** | PIP 35 cmH_2_O, PEEP 10 cmH_2_O, Rate 35, FiO_2_ 0.6, Tidal Volume 8mL/kg |
| **Physical Exam** | Intubated, sedated, tachycardic, Difficult to assess her breath sounds, minimal chest rise, abdomen soft, extremities very cool distally |
| **Pertinent Labs** | Normal electrolytes, Patient ABG 7.19/88/60/18 |

1. Based on the information provided, how would you categorize the patient's oxygen delivery?
   - Adequate
   - Inadequate
   - I don’t know
2. Which variable was most important in your determination of the adequacy of oxygen delivery in this patient?
   - HR
   - BP
   - O2Sat
   - SvO2
   - ABG
   - I don’t know
3. What is the most likely etiology of her low-flow state?
   - Inadequate preload
   - Inadequate RPM
   - Excessive afterload
   - I don’t know
4. **What did you like about this session? _____________**
5. **How could we improve this session? ______________**

***Knowledge Questions Answers:***

*a) ECMO flow rate ↓, the pump speed is stable, and the post membrane pressures ↑*

*b) Inadequate*

*c) SVO2*

*d) Inadequate Preload*
