# Supplementary material for: Enhancing Pediatric Extracorporeal Membrane Oxygenation Education Through Process-Oriented Guided Inquiry Learning Sessions for Fellows and Advanced Practice Providers
Source: MedEdPORTAL. 2026 May 12;22:11600. doi: 10.15766/mep_2374-8265.11600 (PMC13161199; doi:10.15766/mep_2374-8265.11600)
Supplement: Supplementary file 1 — VA-ECMO Learner Handout.docxVV-ECMO Learner Handout.docxVA-ECMO Facilitator Guide.docxVV-ECMO Facilitator Guide.docxVA-ECMO Slides.pptxVV-ECMO Slides.pptxVA-ECMO Presurvey.docxVV-ECMO Presurvey.docxVA-ECMO Postsurvey.docxVV-ECMO Postsurvey.docx [file mep_2374-8265.11600-s001.zip › _Educational Summary Report_11600.pdf]

MedEdPORTAL
THE AAMC JOURNAL OF TEACHING AND LEARNING RESOURCES

Original Publication

Open Access

Enhancing Pediatric Extracorporeal Membrane Oxygenation Education Through Process-Oriented Guided Inquiry Learning Sessions for Fellows and Advanced Practice Providers

Maria Abou Nader, MD*, Matthew K. Leroue, MD, Kevin B. Kilgallon, MD, John S. Kim, MD, Ryan J. Good, MD

*Corresponding author: abounader.maria@gmail.com

Abstract

Introduction: Pediatric fellows and advanced practice providers face challenges in acquiring extracorporeal membrane oxygenation (ECMO) management skills due to low patient volume, high-risk cases, and lack of standardized training guidelines. We sought to develop case-based ECMO education sessions using the Process Oriented Guided Inquiry Learning (POGIL) format and evaluate their impact on participants. **Methods:** We designed two 60-minute, case-based ECMO education sessions: Low Flow on Venoarterial-ECMO (session 1) and Hypoxemia on Venovenous-ECMO (session 2). Participants from 1 institution completed voluntary pre/postsession surveys with knowledge-based questions and 5-point Likert scales (1 = *strongly disagree*; 5 = *strongly agree*) to assess participants' confidence in managing ECMO patients. Comparative analysis was performed on data from participants who completed both surveys. **Results:** Among session 1 participants ($n = 26$), all completed the presession survey, 20 completed the postsession survey, and 12 completed both. Among session 2 participants ($n = 23$), all completed the presession survey, 13 completed the postsession survey, and 13 completed both. Knowledge test scores increased significantly from 46% to 80% ($P < .05$, $n = 12$) after session 1, and from 29% to 86% ($P < .05$, $n = 13$) after session 2. Participants' confidence increased significantly after both sessions. Most participants *strongly agreed* or *somewhat agreed* that the content of both sessions was relevant and appropriate to their level of training. **Discussion:** POGIL could serve as an effective method to address advanced practice provider and fellow knowledge gaps and increase understanding of how to approach challenges for patients receiving ECMO.

Keywords

Pediatric Critical Care Medicine, Extracorporeal Membrane Oxygenation (ECMO), Process-Oriented Guided Inquiry Learning Sessions (POGIL), Case-Based Learning

Educational Objectives

By the end of this activity, learners will be able to:

1. Generate a differential diagnosis for low flow on venoarterial extracorporeal membrane oxygenation (VA-ECMO).
2. Identify the clinical signs and parameters indicative of elevated afterload and low preload on VA-ECMO.
3. Generate a differential diagnosis for hypoxemia in patients on venovenous extracorporeal membrane oxygenation (VV-ECMO).
4. Identify clinical signs and parameters indicative of oxygenator failure on VV-ECMO and decreased preload as an etiology of hypoxemia on VV-ECMO.
5. Identify clinical signs and parameters indicative of recirculation on VV-ECMO and generate a differential diagnosis for recirculation.

Citation:
Nader MA, Leroue MK, Kilgallon KB, Kim JS, Good RJ. Enhancing pediatric extracorporeal membrane oxygenation education through process-oriented guided inquiry learning sessions for fellows and advanced practice providers. *MedEdPORTAL*. 2026;22:11600.
https://doi.org/10.15766/mep_2374-8265.11600

Introduction

Extracorporeal membrane oxygenation (ECMO) is a life-sustaining therapy increasingly used in the pediatric critical care setting.[1-3] Its complexity, high cost, and the potential for life-threatening complications[1,4,5] make education for pediatric fellows and advanced practice providers (APPs) critically important. Effective ECMO management requires a deep understanding of

Copyright © 2026 Nader et al. This is an open-access publication distributed under the terms of the Creative Commons Attribution license.

cardiopulmonary physiology, patient selection, circuit mechanics, and rapid data synthesis skills.

Pediatric fellows and APPs often face a significant gap in confidence, experience, and readiness to manage ECMO independently. A national needs assessment conducted by Farley[6] found that more than 20% of pediatric program directors felt their fellows were not competent to manage ECMO by the time of graduation, and more than one-half reported educational gaps in their training. Furthermore, fellows themselves reported lacking confidence and expressed a desire for more structured supplemental education. As ECMO use continues to rise, especially highlighted during crises like the COVID-19 pandemic,[7,8] the need for robust, standardized training has become more urgent to ensure patient safety and optimal outcomes.

Despite the growing utilization of ECMO, there is still no universal standardized training curriculum for fellows and APPs,[9] and this lack of standardization can lead to variability in training quality and provider preparedness. Current educational strategies described are mostly for ECMO specialists and include a combination of lectures, self-learning, hands-on practice in a wet lab, and simulation.[10] Simulation-based training, particularly high-fidelity simulation, is widely recognized as a cornerstone of effective ECMO education. It allows providers to safely develop technical, cognitive, and behavioral skills without posing risk to patients,[11-13] to practice managing rare but critical emergencies,[14,15] and to enhance teamwork and communication,[14,16] which are essential skills in high-stakes, multidisciplinary ECMO scenarios. A multimodal educational bootcamp that consisted of didactics, hands-on circuit demonstration, and simulation was shown to improve knowledge and learners' confidence in managing patients on ECMO.[17] This model would be an excellent option for educating fellows and APPs, but simulation can be difficult to implement and is not always feasible. In fact, many programs, including community or smaller academic centers, may not have access to high-fidelity simulation equipment, dedicated faculty, or sufficient protected educational time to implement simulation-based ECMO education.

Process Oriented Guided Inquiry Learning (POGIL) is an instructional approach that emphasizes structured, small-group activities to promote active learning, critical thinking, and teamwork. In POGIL, learners work collaboratively to solve guided-inquiry problems, with instructors acting as facilitators. In medical education, POGIL has been associated with improved academic performance, higher course satisfaction, and better long-term retention of material when compared with traditional lectures.[18,19] It also supports the development of essential skills such as problem-solving and teamwork, which are crucial for clinical practice.[18-20]

Although POGIL has not yet been described in the context of ECMO education, it could be an additive or alternative approach for programs lacking simulation resources. We designed 2 ECMO education sessions using the POGIL format, with the dual intent of introducing a more engaging, inquiry-based method and offering a superior alternative to traditional didactic lectures.

Methods

Development

We developed two 60-minute ECMO education sessions utilizing the POGIL format. The first session focused on the topic of Low Flow on Venoarterial-ECMO (VA-ECMO). While bleeding and thrombosis are well-described as the most common complications associated with VA-ECMO, both can contribute to the development of a low blood-flow state, making this a critical area for focused education. The second session addressed the topic of Hypoxemia on Venovenous-ECMO (VV-ECMO), since low blood-oxygen levels represent one of the most frequently encountered challenges in patients supported on VV-ECMO.[1]

Each session was structured around 3 clinical cases. Patient histories and -clinical data were adapted from the Extracorporeal Life Support Organization's published neonatal and pediatric ECMO simulation scenarios,[21] with modifications made to better fit the educational context and specific case scenarios. We chose the POGIL format because it fosters active engagement, critical thinking, and team-based problem solving. Those are all essential skills for managing the complexities of ECMO, which requires the integration of evolving physiologic data and device parameters in real time. POGIL is highly effective as it supports learners in constructing their own understanding through guided inquiry and encouraging deeper processing of information and retention of concepts.[18-20] This approach was also designed to demonstrate how traditional simulation-based education can be translated into a more interactive, case-based learning format, particularly in programs without access to a simulation center. For each session, the first case introduced a structured framework for evaluating the clinical problem, which learners then applied to the subsequent 2 cases. All cases were then reviewed by 2 pediatric intensivists, including 1 with expertise in medical education, and 2 pediatric cardiac intensivists and ECMO experts to ensure clinical accuracy and logical case progression.

Copyright © 2026 Nader et al. This is an open-access publication distributed under the terms of the Creative Commons Attribution license.

Learners

Both sessions were delivered to pediatric critical care fellows at all levels at Children's Hospital Colorado, as part of their scheduled fellow conferences. The first session, Low Flow on VA-ECMO, was also presented to pediatric cardiology fellows at all levels at Children's Hospital Colorado, during their designated conference time. Pediatric critical care APPs at Children's Hospital Colorado were invited to attend both sessions, when available. Learners were expected to have a foundational understanding of ECMO, including how the circuit functions, its core components, and the physiologic principles involved.

Implementation

During the 2023–2024 and 2024–2025 academic years, we implemented the educational sessions during prescheduled fellow conferences. The VA-ECMO session was delivered twice to pediatric critical care fellows in February 2024, and June 2025, and once to pediatric cardiology fellows in April 2025. The VV-ECMO session was conducted in June 2024 and April 2025. Given that the POGIL approach emphasizes active, collaborative, and in-person engagement, participants were strongly encouraged to attend the sessions in person. All sessions took place in the designated conference rooms where weekly fellow conferences are typically held. A virtual attendance option was available for those unable to participate in person. For each session, a question sheet (Appendix A and Appendix B) was distributed to the learners, while the facilitator had an answer key (Appendix C and Appendix D) and a PowerPoint presentation (Appendix E and Appendix F). The slides included the session's questions as well as key figures and tables relevant to the cases. The facilitator's role was to guide learners through structured, inquiry-based ECMO cases, helping them stay on track, encouraging participation from all attendees, and prompting critical thinking through guiding questions included in the materials, rather than directly providing answers. The facilitator also ensured that learners understood the clinical reasoning framework presented in the initial case and supported them in applying that reasoning to subsequent ECMO scenarios. This study was reviewed by the Colorado Multiple Institutional Review Board (COMIRB) and was determined to be exempt from IRB review (Category 1; COMIRB no. 23-2585).

Assessment

To evaluate the impact of our education sessions, we collected quantitative and qualitative data through presession surveys (Appendix G and Appendix H) and postsession surveys (Appendix I and Appendix J) for each session. The survey was distributed via email, with 1 reminder email sent prior to each session and an additional reminder email sent afterward. To encourage further participation, a QR code linking to the surveys was also displayed at the beginning and end of each session. Participants were allotted five minutes during the session to complete the survey if they had not already done so.

These surveys included confidence-related questions rated on a 5-point Likert scale (1 = *strongly disagree*; 5 = *strongly agree*) as well as de novo knowledge-based questions. The knowledge questions for session 1 were case-based and closely resembled the clinical scenarios discussed during the session. They followed the same diagnostic framework introduced in the initial case. In contrast, the knowledge questions for session 2 focused on fundamental concepts and tested essential baseline information relevant to the topic. Knowledge scores were calculated as the percentage of correct answers among participants.

To gather qualitative feedback, we included open-ended questions at the end of the postsession surveys. While all surveys were deidentified to protect participant privacy, each participant was asked to create a unique code at the beginning of the survey so we could link their pre- and postsession responses. Comparative analysis was performed on data from participants who completed both surveys.

Results

Among the 26 participants in session 1, including 15 pediatric critical care fellows, 5 pediatric cardiology fellows, 5 pediatric APPs, and 1 attending physician, 26 learners completed the presession survey, 20 completed the postsession survey, and 12 completed both. Of those who completed both surveys, 67% were pediatric critical care fellows. Among these 12 learners, there was a statistically significant increase in confidence regarding the session objectives, including confidence in generating a differential diagnosis ($P < .05$), identifying clinical signs and parameters indicative of elevated afterload on VA-ECMO ($P < .05$), and recognizing signs of low preload on VA-ECMO ($P < .05$) (Table). Knowledge test scores also improved significantly, rising from a score of 46% presession to 80% postsession ($P < .05$). Nearly all participants *somewhat agreed* or *strongly agreed* that the content was relevant to the care of critically ill children on VA-ECMO and appropriate for their level of training.

Session 2 had 23 participants: 14 pediatric critical care fellows, 7 pediatric APPs, and 2 attending physicians. Presession surveys were completed by all participants, with 13 completing both the presession and postsession surveys. Postsession surveys showed a significant improvement in learners' confidence,

Copyright © 2026 Nader et al. This is an open-access publication distributed under the terms of the Creative Commons Attribution license.

Table. Pre- and Postsession Confidence Ratings of Educational Objectives in Enhancing Pediatric ECMO Education Through Process Oriented Guided Inquiry

Objectives	Presession Score *Mdn* (IQR)[a]	Postsession Score *Mdn* (IQR)[a]	*P*
**VA-ECMO Session Objectives (*n* = 12)**			
Generating a differential diagnosis for low flow on VA-ECMO	3 (2–4)	4 (4–4.25)	.0002
Identifying signs indicative of elevated afterload on VA-ECMO	3 (1–4)	4 (4–4.25)	<.0001
Identifying signs indicative of low preload on VA-ECMO	3 (1.75–4)	4 (4–5)	<.0001
**VV-ECMO Session Objectives (*n* = 13)**			
Generating a differential diagnosis for hypoxemia on VV-ECMO	4 (3–4)	5 (5–5)	.014
Identifying signs indicative of oxygenator failure	4 (3–4)	5 (5–5)	.012
Identifying signs indicative of recirculation	4 (3–4)	5 (5–5)	.016
Identifying signs indicative of decreased preload as a cause of hypoxemia	4 (2–4)	5 (5–5)	.0039

Abbreviations: VA-ECMO, venoarterial extracorporeal membrane oxygenation; VV-ECMO, venovenous extracorporeal membrane oxygenation.
[a]Pediatric fellows and advanced practice providers rated their confidence on a 5-point Likert scale (1 = *strongly disagree* to 5 = *strongly agree*).

particularly in the ability to formulate a differential diagnosis for hypoxemia in patients on VV-ECMO ($P < .05$), and in identifying key clinical signs suggesting oxygenator failure ($P < .05$), recirculation ($P < .05$), or decreased preload as a cause of hypoxemia ($P < .05$) (Table). There was a significant increase in the knowledge scores following the session, increasing from 29% presession to 86% postsession ($P < .05$). All respondents agreed that the session content was applicable to the management of critically ill pediatric patients on ECMO and suitable for their level of expertise.

Participants provided highly positive feedback in the open-ended questions of the survey, emphasizing the interactive, case-based format and clinical relevance. One participant noted, “It was very well organized to walk learners through the different etiologies of low flow states and work through problems in a stepwise fashion.” Another participant shared that “I liked the case-based presentation—very educational.” Suggestions included adding more cases and slowing the initial didactic portion. Overall, the session was described as “super useful,” “well-organized,” and “engaging,” and was considered to promote “excellent discussions.”

Discussion

We successfully demonstrated that the POGIL method can be an effective and practical alternative to traditional lectures or simulation-based training for ECMO education. The sessions enabled an interactive, collaborative learning environment in which participants, primarily pediatric cardiology and critical care fellows, as well as pediatric critical care APPs, reported increased confidence in identifying and managing 2 important ECMO-related issues. The motivation for this project arose from feedback indicating that trainees often felt underprepared to manage patients on ECMO and were seeking more structured, applicable educational experiences. At the time of project initiation, our institution did not have an established ECMO simulation program, though one is currently under development. This made it necessary to design educational experiences beyond traditional didactics, which are often less engaging and less effective for complex clinical topics such as ECMO-related ones.[22,23]

To our knowledge, POGIL had not been previously applied to ECMO education, making this an innovative and novel use of this method. We chose this approach precisely because of its structured yet flexible format, which allows learners to engage in critical thinking, pattern recognition, and collaborative problem-solving. While POGIL is not well described in the literature regarding critical care education, its emphasis on guided inquiry and teamwork is ideal to bridge the gap between passive learning and high-fidelity simulation. This approach allowed us to create a meaningful and interactive experience using only case-based materials while still reinforcing key clinical concepts. Furthermore, the virtual format expanded access, enabling participation in the educational sessions not only by our trainees but also by fellows at another institution, thereby broadening the educational impact.

Our evaluation focused on Level 1 and Level 2 of the Kirkpatrick curriculum development model for assessing educational effectiveness. At Level 1 (Reaction), participants reported high satisfaction with the sessions. They found the format engaging, clinically relevant, and well-suited to their level of training. One of the most powerful aspects of the experience was the collaborative learning that occurred between senior and junior fellows, APPs, and other learners. The sessions promoted open discussion and shared problem-solving in a low-pressure

Copyright © 2026 Nader et al. This is an open-access publication distributed under the terms of the Creative Commons Attribution license.

environment, unlike high-fidelity simulations, which can be intimidating or logistically demanding. This psychologically safe atmosphere allowed for more effective learning, particularly in a topic area where comfort and confidence are essential.

At Level 2 (Learning), learners showed measurable improvements in knowledge and confidence. Knowledge assessment scores improved between presession and postsession, and learners expressed increased confidence in generating differential diagnoses for low-flow states on VA-ECMO and hypoxemia on VV-ECMO. The structured framework used in the sessions, including diagnostic algorithms, was frequently cited in participant feedback as especially useful and memorable. Several participants reported applying this framework directly in clinical settings, suggesting that the learning translated beyond the session itself and into real-world application.

Creation of this curriculum was an interesting journey, and we embraced the feedback we received to improve subsequent sessions. We started each session by discussing cannulation strategies, and as mentioned, the first case was designed to introduce a framework or approach to the topic at hand. One piece of feedback we received was to slow down and spend more time explaining these initial concepts. In response, we dedicated more time in the following sessions to cover these concepts in detail. As a result, we noticed that learners were more engaged and were able to troubleshoot the subsequent cases much more efficiently.

Despite these encouraging results, there are limitations to consider. Our sample size was small and limited to a single institution, which may affect the generalizability of findings. Furthermore, we assessed only Kirkpatrick Levels 1 and 2 for evaluating educational effectiveness. We did not evaluate long-term behavior change (Level 3) or patient outcomes (Level 4), both of which are essential to fully understanding the educational impact. Survey response rates posed another limitation. Although we sent the surveys by email and provided QR codes in the session slides to facilitate survey completion, not all learners participated. Some may have needed more than the 5 minutes allotted, and others may have experienced survey fatigue. Additionally, some fellows may have attended the same session more than once, which could have influenced outcomes, although repetition is known to reinforce learning,[24] particularly in complex domains like ECMO. Lastly, an additional question assessing session and facilitator satisfaction was added to the survey instrument after initial data collection (included in Appendix I and Appendix J). Therefore, no data for this item is available in the present analysis.

In summary, our findings suggest that POGIL is a promising, low-resource strategy for ECMO education that can be implemented without the need for high-fidelity simulation. This format can be integrated easily into existing educational time blocks, as it requires only 1 facilitator and minimal materials. Based on the success of this initiative, we are now developing additional POGIL sessions covering other ECMO-related and critical care topics. Future directions include assessing long-term knowledge retention, evaluating behavioral change in clinical practice, and exploring whether this model can be scaled across institutions to support broader improvements in ECMO education.

Appendices

A. VA-ECMO Learner Handout.docx
B. VV-ECMO Learner Handout.docx
C. VA-ECMO Facilitator Guide.docx
D. VV-ECMO Facilitator Guide.docx
E. VA-ECMO Slides.pptx
F. VV-ECMO Slides.pptx
G. VA-ECMO Presurvey.docx
H. VV-ECMO Presurvey.docx
I. VA-ECMO Postsurvey.docx
J. VV-ECMO Postsurvey.docx

All appendices are peer reviewed as integral parts of the Original Publication.

Maria Abou Nader, MD: Pediatric Critical Care Medicine Fellow, Division of Pediatric Critical Care, University of Colorado School of Medicine, Children's Hospital Colorado; ORCID: https://orcid.org/0009-0003-7522-675X

Matthew K. Leroue, MD: Assistant Professor of Pediatrics, Division of Pediatric Critical Care, University of Colorado School of Medicine, Children's Hospital Colorado

Kevin B. Kilgallon, MD: Assistant Professor of Pediatrics, Division of Pediatric Cardiac Critical Care, Case Western University School of Medicine, Rainbow Babies and Children's Hospital

John S. Kim, MD: Associate Professor of Pediatrics, Division of Cardiology, University of Colorado School of Medicine; Children's Hospital of Colorado Heart Institute

Ryan J. Good, MD: Associate Professor of Pediatrics, Division of Pediatric Critical Care, University of Colorado School of Medicine, Children's Hospital Colorado

Copyright © 2026 Nader et al. This is an open-access publication distributed under the terms of the Creative Commons Attribution license.

Disclosures
None to report.

Funding/Support
None to report.

Ethical Approval
The University of Colorado Institutional Review Board reviewed this project.

References

1. Barbaro RP, Brodie D, MacLaren G. Bridging the gap between intensivists and primary care clinicians in extracorporeal membrane oxygenation for respiratory failure in children: a review. *JAMA Pediatr*. 2021;175(5):510-517. https://doi.org/10.1001/jamapediatrics.2020.5921
2. Cashen K, Regling K, Saini A. Extracorporeal membrane oxygenation in critically ill children. *Pediatr Clin North Am*. 2022;69(3):425-440. https://doi.org/10.1016/j.pcl.2022.01.008
3. Valencia E, Nasr VG. Updates in pediatric extracorporeal membrane oxygenation. *J Cardiothorac Vasc Anesth*. 2020;34(5):1309-1323. https://doi.org/10.1053/j.jvca.2019.09.006
4. Fernando SM, Qureshi D, Tanuseputro P, et al. Long-term survival and costs following extracorporeal membrane oxygenation in critically ill children-a population-based cohort study. *Crit Care*. 2020;24(1):131. https://doi.org/10.1186/s13054-020-02844-3
5. Teijeiro-Paradis R, Gannon WD, Fan E. Complications associated with venovenous extracorporeal membrane oxygenation-What can go wrong? *Crit Care Med*. 2022;50(12):1809-1818. https://doi.org/10.1097/CCM.0000000000005673
6. Farley L. *A Characterization of ECMO Training Practices in Pediatric Fellowships: Are We Doing Enough?* Master's thesis. Ohio State University; 2022. Accessed April 3, 2026. https://etd.ohiolink.edu/acprod/odb_etd/ws/send_file/send?accession=osu1651754260741694
7. Watanabe A, Yasuhara J, Karube T, et al. Extracorporeal membrane oxygenation in children with COVID-19: A systematic review and meta-analysis. *Pediatr Crit Care Med*. 2023;24(5):406-416. https://doi.org/10.1097/PCC.0000000000003113
8. Bembea MM, Loftis LL, Thiagarajan RR, et al. Extracorporeal membrane oxygenation characteristics and outcomes in children and adolescents with COVID-19 or Multisystem Inflammatory Syndrome admitted to U.S. ICUs. *Pediatr Crit Care Med*. 2023;24(5):356-371. https://doi.org/10.1097/PCC.0000000000003212
9. Crannell WC, Zakhary B, Hamilton H, Brasel K, Zonies D. Design of an entrustable professional activity for adult extracorporeal membrane oxygenation. *Surg Open Sci*. 2019;2(1):42-45. https://doi.org/10.1016/j.sopen.2019.09.001
10. Weems MF, Friedlich PS, Nelson LP, et al. The role of extracorporeal membrane oxygenation simulation training at extracorporeal life support organization centers in the United States. *Simul Healthc*. 2017;12(4):233-239. https://doi.org/10.1097/SIH.0000000000000243
11. Park Y, Hocutt G, Wetzel E, et al. A multimodal educational boot camp for training fellows in pediatric extracorporeal membrane oxygenation (ECMO). *MedEdPORTAL*. 2024;20:11455. https://doi.org/10.15766/mep_2374-8265.11455
12. Sawyer T, Stavroudis TA, Ades A, et al. Simulation in neonatal-perinatal medicine fellowship programs. *Am J Perinatol*. 2020;37(12):1258-1263. https://doi.org/10.1055/s-0039-1693465
13. Henricksen JW, Troy L, Siefkes H. Pediatric critical care medicine fellowship simulation use survey. *Pediatr Crit Care Med*. 2020;21(10):e908-e914. https://doi.org/10.1097/PCC.0000000000002343
14. Brown PJP. Process-oriented guided-inquiry learning in an introductory anatomy and physiology course with a diverse student population. *Adv Physiol Educ*. 2010;34(3):150-155. https://doi.org/10.1152/advan.00055.2010
15. Walker L, Warfa AM. Process oriented guided inquiry learning (POGIL(R)) marginally effects student achievement measures but substantially increases the odds of passing a course. *PLoS One*. 2017;12(10):e0186203. https://doi.org/10.1371/journal.pone.0186203
16. Vanags T, Pammer K, Brinker J. Process-oriented guided-inquiry learning improves long-term retention of information. *Adv Physiol Educ*. 2013;37(3):233-341. https://doi.org/10.1152/advan.00104.2012
17. Costa ML, van Rensburg L, Rushton N. Does teaching style matter? A randomised trial of group discussion versus lectures in orthopaedic undergraduate teaching. *Med Educ*. 2007;41(2):214-217. https://doi.org/10.1111/j.1365-2929.2006.02677.x
18. Miller CJ, McNear J, Metz MJ. A comparison of traditional and engaging lecture methods in a large, professional-level course. *Adv Physiol Educ*. 2013;37(4):347-355. https://doi.org/10.1152/advan.00050.2013
19. Johnston L, Su L, Allan C. *Neonatal and Pediatric ECMO Simulation Scenarios—Prepared Jointly by ELSO and IPSS*. Extracorporeal Life Support Organization (ELSO) and the International Pediatric Simulation Society (IPSS); 2023.
20. Kirkpatrick JD, Kirkpatrick WK. *Kirkpatrick's Four Levels of Training Evaluation: A New World*. ATD Press; 2016.
21. O'Donoghue D, Davison G, Hanna LJ, McNaughten B, Stevenson M, Thompson A. Calibration of confidence and assessed clinical skills competence in undergraduate paediatric OSCE scenarios: a mixed methods study. *BMC Med Educ*. 2018;18(1):211. https://doi.org/10.1186/s12909-018-1318-8
22. Garg A, Arora A, Hand IL. Pediatric resident attitudes and knowledge of critical congenital heart disease screening. *Pediatr Cardiol*. 2016;37(6):1137-1140. https://doi.org/10.1007/s00246-016-1407-6

Copyright © 2026 Nader et al. This is an open-access publication distributed under the terms of the Creative Commons Attribution license.

23. Augustin M. How to learn effectively in medical school: test yourself, learn actively, and repeat in intervals. *Yale J Biol Med*. 2014;87(2):207-212.

24. Price DW, Wang T, O'Neill TR, et al. The effect of spaced repetition on learning and knowledge transfer in a large cohort of practicing physicians. *Acad Med*. 2025;100(1):94-102. https://doi.org/10.1097/ACM.0000000000005856

Received: August 27, 2025
Accepted: March 25, 2026
Published: May 12, 2026

Copyright © 2026 Nader et al. This is an open-access publication distributed under the terms of the Creative Commons Attribution license.
